# Characterisation of N-glycans in the epithelial-like tissue of the rat cochlea

**DOI:** 10.1038/s41598-018-38079-0

**Published:** 2019-02-07

**Authors:** Yoriko Nonomura, Seishiro Sawamura, Ken Hanzawa, Takashi Nishikaze, Sadanori Sekiya, Taiga Higuchi, Fumiaki Nin, Satoru Uetsuka, Hidenori Inohara, Shujiro Okuda, Eiji Miyoshi, Arata Horii, Sugata Takahashi, Shunji Natsuka, Hiroshi Hibino

**Affiliations:** 1 Department of Molecular Physiology, Niigata University School of Medicine, Niigata, Japan; 2 Department of Otorhinolaryngology–Head and Neck Surgery, Niigata University School of Medicine, Niigata, Japan; 3 Department of Biology, Faculty of Science, Niigata University, Niigata, Japan; 4 Koichi Tanaka Mass Spectrometry Research Laboratory, Shimadzu Corporation, Kyoto, Japan; 5 Center for Transdisciplinary Research, Niigata University, Niigata, Japan; 6 Department of Otorhinolaryngology–Head and Neck Surgery, Graduate School of Medicine, Osaka University, Osaka, Japan; 7 Bioinformatics Laboratory, Niigata University School of Medicine, Niigata, Japan; 8 Division of Health Sciences, Graduate School of Medicine, Osaka University, Osaka, Japan; 9 AMED-CREST, AMED, Niigata, Japan

## Abstract

Membrane proteins (such as ion channels, transporters, and receptors) and secreted proteins are essential for cellular activities. N-linked glycosylation is involved in stability and function of these proteins and occurs at Asn residues. In several organs, profiles of N-glycans have been determined by comprehensive analyses. Nevertheless, the cochlea of the mammalian inner ear, a tiny organ mediating hearing, has yet to be examined. Here, we focused on the stria vascularis, an epithelial-like tissue in the cochlea, and characterised N-glycans by liquid chromatography with mass spectrometry. This hypervascular tissue not only expresses several ion transporters and channels to control the electrochemical balance in the cochlea but also harbours different transporters and receptors that maintain structure and activity of the organ. Seventy-nine N-linked glycans were identified in the rat stria vascularis. Among these, in 55 glycans, the complete structures were determined; in the other 24 species, partial glycosidic linkage patterns and full profiles of the monosaccharide composition were identified. In the process of characterisation, several sialylated glycans were subjected sequentially to two different alkylamidation reactions; this derivatisation helped to distinguish α2,3-linkage and α2,6-linkage sialyl isomers with mass spectrometry. These data should accelerate elucidation of the molecular architecture of the cochlea.

## Introduction

Cellular and tissue functions are precisely and dynamically controlled by a variety of membrane-integral proteins such as receptors, ion channels, and transporters. More than 50% of these proteins are glycosylated^1^. This post-transcriptional modification also occurs in the majority of secreted proteins that mediate the cross-talk among cells^2^. Glycosylation affects Asn residues (N-glycans) or Ser/Thr residues (O-glycans). Recent studies highlighted roles of different N-glycans not only in the processes related to protein stability and trafficking but also in the modulation of actions of membrane proteins^3,4^. Therefore, characterisation of glycan types expressed in each tissue or organ is crucial for elucidation of molecular architectures underlying vital phenomena in various organisms.

**Figure 1:**
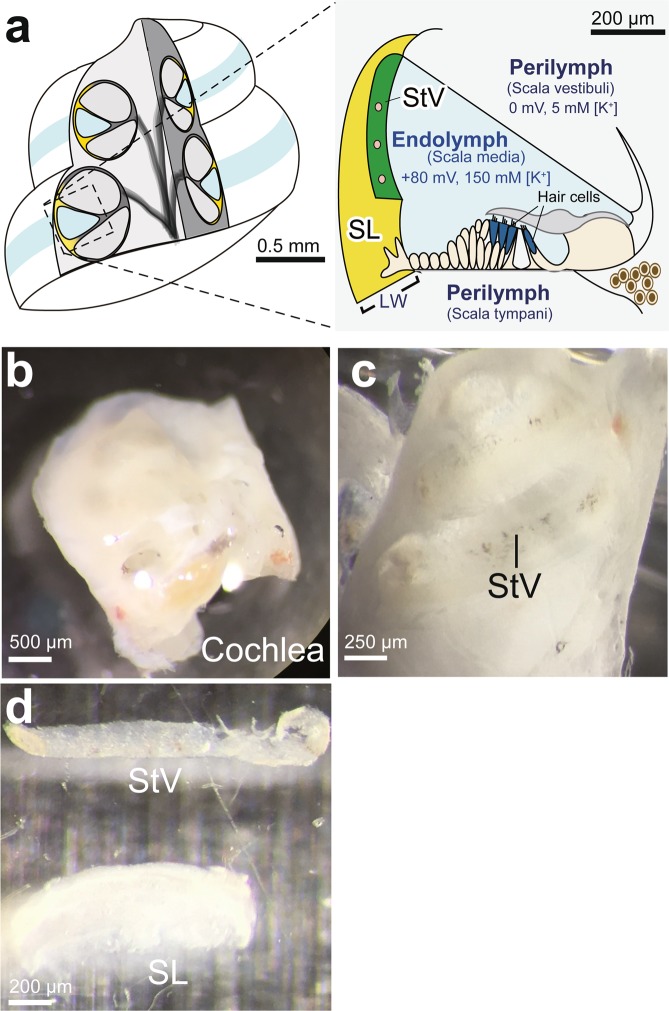
Isolation of the stria vascularis. (**a**) Structure of the cochlea. An overview image and cross-section of this organ are shown in *left* and *right panels*, respectively. In the latter, electrochemical properties of endolymph and perilymph are indicated. StV; stria vascularis, SL; spiral ligament, LW; lateral cochlear wall. (**b**–**d**) Dissociation of StV. The cochlea was dissected from the temporal bone (**b**) and divided sagittally into two parts along the cochlear axis (**c**). The StV was identified by brown pigmentation of the intermediate cells. Then, as shown in (**d**), the StV was carefully peeled away from the SL with a fine needle (see Methods).

Although structures of N-glycans in serum and several organs including the brain, lungs, and kidneys have been comprehensively analysed^5–8^, those in the cochlea of the mammalian inner ear, a small organ of a few millimetres in size, have not yet been sufficiently profiled. In the cochlea, the stria vascularis, an epithelial-like tissue composed of marginal, intermediate, and basal cells, contains numerous capillaries; therefore, it carries a variety of substances including hormones, metabolites, glucose, and even externally applied drugs, from blood to itself and other tissues^9–11^. These actions are likely to be mediated by a considerable number of organic transporters; besides, receptors for growth factors and hormones are expressed in strial cells^12–15^. Strial K^+^ channels and K^+^ uptake transporters maintain a high [K^+^] of 150 mM and a highly positive potential of +80 mV in an extracellular fluid, endolymph; these electrochemical milieus contribute to the maintenance of hearing (Fig. 1a)^16–18^. Other endolymphatic properties such as volume, osmolarity, and pH may be balanced by a variety of ion channels and transporters in the stria^12,19,20^. In addition, marginal cells seem to secrete a few protein types that can be involved in the development of the cochlea^21,22^. Overall, it is plausible that the strial membrane protein networks described above are collectively crucial for cochlear function. In the present study, we focused on the stria vascularis and profiled the structures of its N-glycans, which potentially regulate activities of the membrane and secreted proteins. Our method combining three high performance liquid chromatography (HPLC) types and different modes of multi-stage mass spectrometry (MS^n^) identified 79 different N-glycan species and characterised their structures.

## Results

### Verification of purification of the stria vascularis dissociated from the cochlea

**Figure 2:**
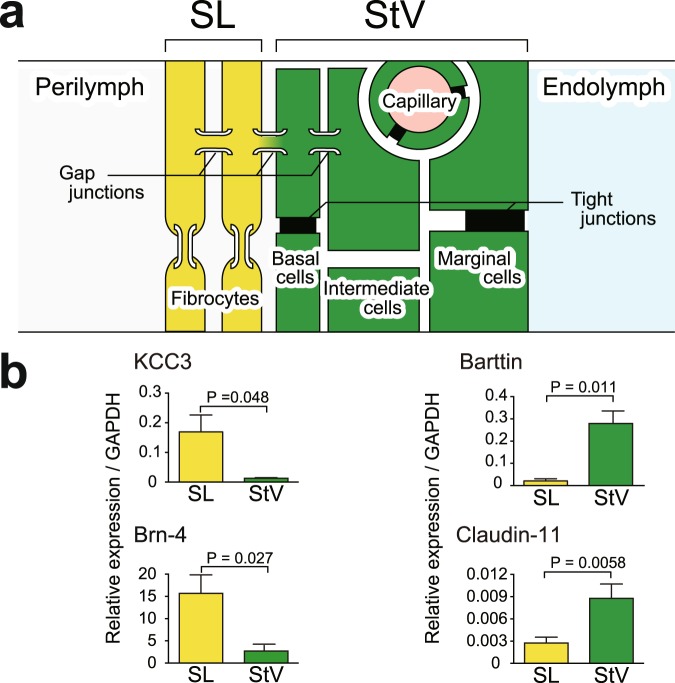
Verification of purity of the stria vascularis in the samples. (**a**) Cellular components of the lateral wall. The stria vascularis (StV) is composed of marginal, intermediate, and basal cells, whereas the spiral ligament (SL) consists of fibrocytes. This scheme was adapted from our earlier work^57^. (**b**) Assessment of purity of the StV in the obtained samples. qPCR analyses were performed separately on the StV (*green bars*) and SL (*yellow bars*) isolated from the lateral cochlear wall. In these assays, the primers for genes encoding K^+^,Cl^−^-cotransporter type 3 (KCC3) and Brn-4, which are exclusively expressed in SL, and those for barttin and claudin 11, which are specific to StV, were used. Quantitative data (n = 3) were collected with reference to an internal gene, glyceraldehyde 3-phosphate dehydrogenase (*GAPDH*); averages, standard errors, and *P* values determined by two-tailed Student’s *t* test are also shown.

The stria vascularis is enriched with capillaries and is tightly attached to a neighbouring tissue, the spiral ligament, in the lateral cochlear wall (Fig. 1a). We first perfused rats systemically with saline to remove blood from the stria vascularis and then carefully isolated this tissue from the lateral wall (Fig. 1b–d; see also ‘Methods’). Nevertheless, we were concerned about contamination with the ligament in the samples. We therefore decided to evaluate the purity of the samples. Total-RNA samples extracted from the isolated stria vascularis and spiral ligament from 8–10 cochleae (4–5 rats) were independently analysed by quantitative PCR (qPCR) with primers for the four genes that encode the proteins specific to each tissue (Fig. 2). This series of assays was repeated three times with different batches of cochleae. The stria vascularis is separated from endolymph and the spiral ligament by marginal and basal cells, respectively (Fig. 2a). Our qPCR assays (Fig. 2b) showed that mRNA of Cl^−^channel β-subunit barttin (Bsnd), which is exclusively expressed in marginal cells^23^, was abundant in the samples of the stria but was only moderately detectable in those of the ligament. Similar results were obtained with the primers specific for claudin 11 (Cldn11), a component of tight junction strands in basal cells^24,25^. We next performed assays with primers for a K^+^,Cl^−^-cotransporter KCC3 (slc12a6) and transcription factor Brn-4 (pou3f4), both of which are present in the ligament but absent in the stria vascularis^26,27^. The amounts of mRNAs of the two genes were negligible in the samples of the stria but well detectable in those of the ligament. These tendencies were observed in all the three series of experiments (Fig. 2b). The observations described above confirmed not only the purity of the strial samples but also the reliability of our isolation technique.

### Detection and characterisation of glycans in the stria vascularis

**Figure 3:**
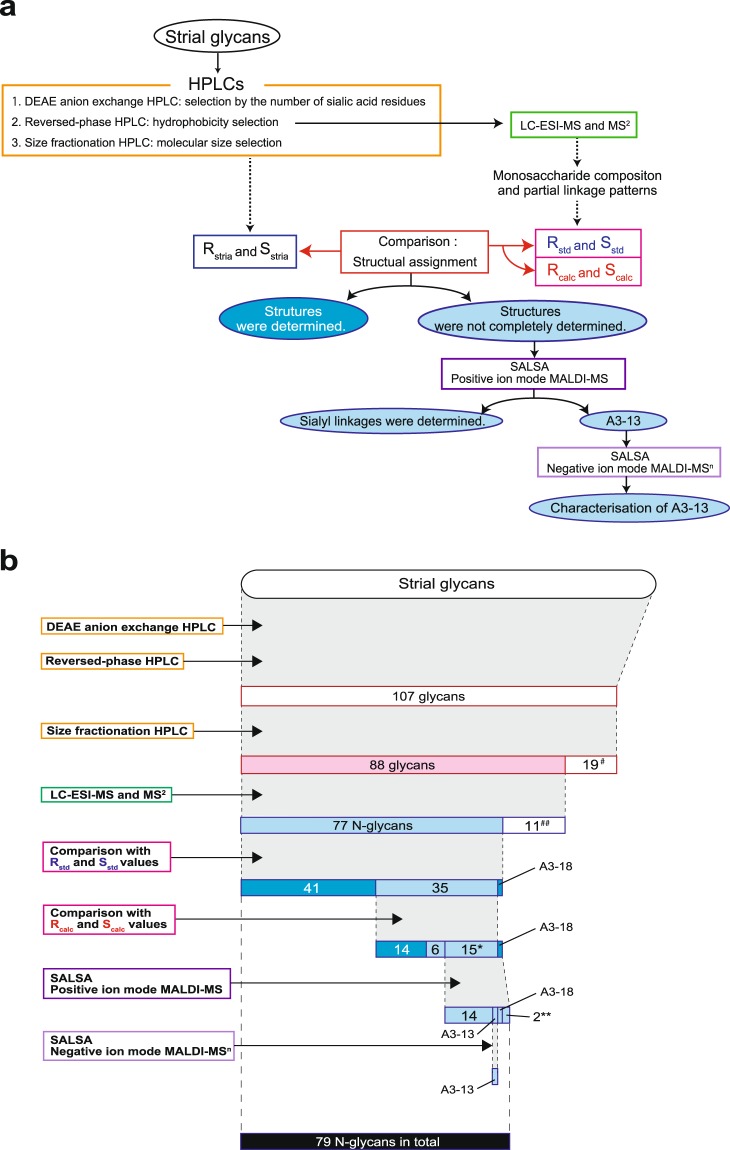
The workflow for characterisation of strial glycans. (**a**) An overview flowchart of the experiments conducted in this study. As the first step, glycans obtained from the stria vascularis were sequentially fractionated by three different HPLC types described in the *orange box* (*upper left*). The elution times recorded in reversed-phase and size fractionation HPLCs represent R and S values (R_stria_ and S_stria_), respectively^29,32^ (*blue box*). In parallel, the original samples extracted via reversed-phase HPLC were analysed by positive ion mode LC-ESI-MS and MS^2^ (*green box* in the *upper right*); the spectra provided monosaccharide composition and linkage patterns. On the basis of these data, of the HPLC analyses of 194 standard glycans^7,28^, and of the empirical additivity rule^7^, we predicted set(s) of R_std_ and S_std_ values or those of R_calc_ and S_calc_ values for each glycan (*pink box*). A comparison between this information and the values of R_stria_ and S_stria_ determined the structures of some glycan species (the *deep-blue*-filled *ellipse*). The other glycans whose structures were not completely determined by this procedure (the *pale-blue*-filled *ellipse*) were next subjected to SALSA and positive ion mode MALDI-MS (*dark-purple box*). This experiment revealed the sialyl linkage patterns of the majority of the analysed glycans (the *pale-blue*-filled *ellipse*). Nevertheless, characterisation of the sialyl linkage in A3-13 required a series of more complicated analyses consisting of SALSA and negative ion mode MALDI-MS^n^ (*pale-purple box*). DEAE: diethylaminoethyl, HPLC: high performance liquid chromatography, LC-ESI: liquid chromatography with electrospray ionisation, MS: mass spectrometry, R_std_: R value of standard glycans, S_std_: S value of standard glycans, R_calc_: calculated R value, S_calc_: calculated S value, SALSA: sialic-acid-linkage–specific alkylamidation, MALDI: matrix-assisted laser desorption/ionisation. (**b**) The numbers of strial glycans extracted or characterised by each series of the methods described in (**a**). The methods are shown on the *left side*. Of 107 glycans collected by reversed-phase HPLC, each of the 19 species marked by a *single hash tag* (^#^) was detected as a fraction whose peak area was less than 2% of that of the N-5 fraction in the subsequent HPLC chromatogram, and therefore each was excluded from further assays (see *text*). Glycans indicated by a *double hash tag* (^##^) were likely to be O-linked-type or non-specific moieties (see *text*). Groups highlighted in *deep blue* have glycans whose structures were determined perfectly, whereas groups coloured with *pale blue* consist of glycans whose partial linkages were assigned temporarily or those whose linkages were not completely clarified after the analyses. In summary, the structures of 79 strial N-glycans in total were profiled, as indicated by the *black bar*. Details of the experiments for A3-13 and A3-18 species are described in the *main text*. *Sialylated species that have glycosidic linkages inaccessible to the analyses using R and S values. **Species that were initially identified as a single sialylated glycan but later found to have an additional sialyl linkage isomer.

**Figure 4:**
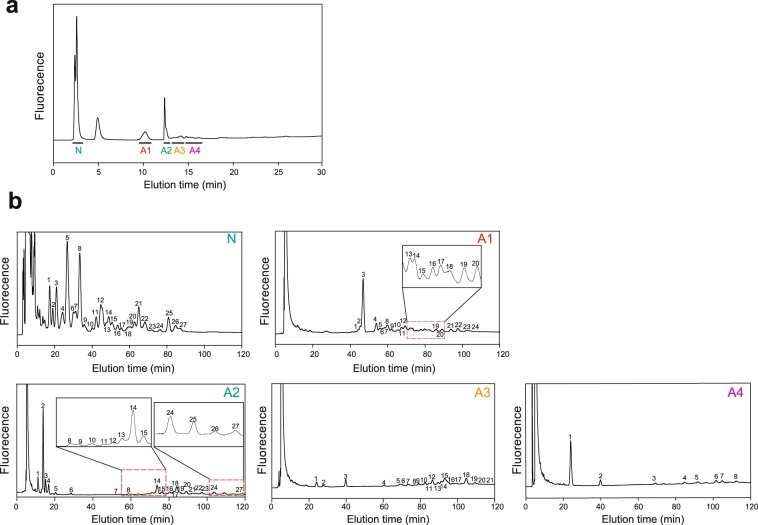
Separation and identification of N-glycans from the stria vascularis. (**a**) An elution profile of diethylaminoethyl (DEAE) anion exchange HPLC. Samples extracted from the stria vascularis were analysed by chromatography. Peaks of compounds without sialylation (class N) and with a modification consisting of one to four sialic acid residues (classes A1 to A4) were detected. A signal between N and A1 peaks derived from non-glycan fluorescent materials^7,28^. (**b**) An elution profile of reversed-phase HPLC. We analysed the fractions isolated by DEAE chromatography [N and A1–A4; see (**a**)]. The numbers point to significant peaks (for the criterion, see Results). The traces marked by *dashed red boxes* in *panels A1* and *A2* are expanded in *insets*. Numerous peaks within the initial ~10 min were likely to contain chemical reagents including pyridylamine and its derivatives^28^.

We next characterised the profile of glycans in the stria vascularis. The strial tissues dissociated from 102 cochleae (51 rats) were combined into one batch. The sample was lyophilised and chemically treated in accordance with the process described in the ‘Methods’ section. The following workflow was carried out by multiple methods as shown in Fig. 3a, and the numbers of strial glycans extracted or characterised by each method are illustrated in Fig. 3b. In this series of experiments, crude pyridylaminated (PA)-glycans from the samples were initially subjected to diethylaminoethyl (DEAE) anion exchange HPLC (Fig. 3a). This method fractionates N-glycans in accordance with the number of the attached sialic acid residues. As shown in Fig. 4a, in the stria vascularis, non-sialic glycans (N) were the most abundant. Smaller amounts of the mono- and di-sialic glycans (classes A1 and A2, respectively) were also detected in the samples. Careful observations unveiled a weak but significant signal of tri- and tetra-sialylated classes (A3 and A4, respectively). A peak between N and A1 signals stemmed primarily from non-glycan materials that persisted during sample processing^7,28^. Then, the five fractions (N, A1–A4) were separately collected and assayed by reversed-phase HPLC, which evaluates hydrophobicity of samples (Figs 3 and 4b). Numerous peaks were obtained in this assay; in this context, the eluates within the initial ~10 min were likely to contain chemical reagents including pyridylamine and its derivatives^28^, and therefore they were omitted for further analyses. We measured the areas of all the individual peaks and found that the fifth fraction in the non-sialic class (N-5) was the most abundant species. On the basis of this observation, a fraction whose area exceeded 2% of that of the N-5 fraction was assumed to be significant. According to this criterion, we re-evaluated all the data obtained by reversed-phase HPLC (Fig. 3a). Consequently, 27 fractions from class N, 24 fractions from class A1, 27 fractions from class A2, 21 fractions from class A3, and eight fractions from the A4 class were collected as presented in Fig. 4b (a total of 107 fractions; see also Fig. 3b). Then, each of these 107 fractions was subjected one by one to size fractionation HPLC, which analyses molecular size of the samples (Fig. 3a; for raw chromatograms, see Supplementary Fig. S1). Each fraction separated with this procedure should be composed of a sole glycan type. Areas of the individual peaks were again determined; the most abundant was a species that was derived from the N-5 fraction extracted by reversed-phase HPLC (N-5 as is). In the products of the size fractionation HPLC, the fraction whose area exceeded 2% of that of the N-5 fraction was assumed to be significant. Of note, in some cases, from the fraction separated by the reversed-phase HPLC, multiple peaks were obtained in the analysis of the size fractionation HPLC; these products were sub-numbered with reference to their elution time (Table 1 and Supplementary Fig. S1 and Tables S1 and S2). As a consequence, at least 88 glycan species were isolated from the strial samples (Fig. 3b and Supplementary Fig. S1).

**Table 1:** Structures of N-glycans in the stria vascularis.

Fraction No.		R_stria_	S_stria_	R_std_ or *R*_*calc*_	S_std_ or *S*_*calc*_	Relative amount	Assigned structure
N	1	31.757	8.37	**32.7**	**8.4**	27.1	Man_8_GlcNAc_2_:(M8)
	2	33.304	7.51	**34.4**	**7.5**	12.8	Man_7_GlcNAc_2_:(M7)
	3	35.137	9.05	**36.4**	**9.1**	32.3	Man_9_GlcNAc_2_:(M9)
	4-1	38.140	7.46	**39.4**	**7.5**	10.4	Man_7_GlcNAc_2_:(M7)
	4-2	38.140	8.21	**38.7**	**8.2**	3.0	Man_8_GlcNAc_2_:(M8)
	5	40.152	6.64	**41.2**	**6.6**	100.0	Man_6_GlcNAc_2_:(M6)
	6-1	42.858	3.15	**43.1**	**3.2**	7.7	Man_2_GlcNAc_2_:(M2)
	6-2	42.858	6.02	**44.5**	**6.1**	4.0	GlcNAcMan_5_GlcNAc_2_:(GnM5)
	7-1	43.755	4.11	**44.5**	**4.1**	9.0	Man_3_GlcNAc_2_:(M3)
	7-2	43.755	5.01	**43.7**	**5.0**	5.2	Man_4_GlcNAc_2_:(M4)
	8-1	45.266	5.80	**46.2**	**5.8**	67.2	Man_5_GlcNAc_2_:(M5)
	8-2	45.266	7.30	**47.0**	**7.3**	4.2	Man_7_GlcNAc_2_:(M7)
	8-3	45.266	9.67	**47.0**	**9.8**	2.9	GluMan_9_GlcNAc_2_:(G1M9)
	10	49.780	6.48			2.0	Ac-Man_9_GlcNAc_2_:(Ac-M9)
	11-1	51.191	4.87	**52.3**	**4.9**	2.4	GlcNAc_2_Man_3_GlcNAc_2_:(AG12)
	12-1	52.739	3.55	*51.5*	*3.4*	8.0	Man_2_FucGlcNAc_2_:(M2F6)
	12-2	53.507	4.45	**54.0**	**4.4**	10.0	Man_3_FucGlcNAc_2_:(M3F6)
	12-3	53.507	5.61	**53.7**	**5.6**	11.5	GlcNAc_2_Man_4_GlcNAc_2_:(GnM4Bs)
	14-2	55.595	6.31	**56.3**	**6.4**	7.7	Gal_2_GlcNAc_2_Man_3_GlcNAc_2_:(BI)
	15	56.807	4.89			3.7	Ac-Man_6_GlcNAc_2_:(Ac-M6)
	16-1	58.699	5.62			2.1	Ac-Man_5_GlcNAc_2_:(Ac-M5)
	16-2	58.699	6.48			2.0	GalGlcNAcHexNAc_3_Man_3_GlcNAc_2_:(Hex_4_HexNAc_6)_
	17	59.934	5.12	**60.3**	**5.1**	2.2	GlcNAc_2_Man_3_FucGlcNAc_2_:(AG12F6)
	20	64.501	5.17	**64.7**	**5.2**	3.9	GlcNAc_2_Man_3_FucGlcNAc_2_:(AG2BsF6)
	21-1	66.000	5.16	**66.6**	**5.2**	17.8	GlcNAc_3_Man_3_GlcNAc_2_:(AG12Bs)
	21-2	66.000	7.39			2.3	Gal_2_GlcNAc_2_HexNAc_2_Man_3_FucGlcNAc_2_:(Hex_5_HexNAc_6_dHex)
	22-1	68.153	5.80	*70.2*	*5.8*	5.6	GalGlcNAc_3_Man_3_GlcNAc_2_:(BIBs-G2)
	22-2	68.153	6.69			3.4	GalGlcNAc_4_Man_3_FucGlcNAc_2_:(TEF6-3G)
	25	76.047	5.38	**76.1**	**5.4**	12.8	GlcNAc_3_Man_3_FucGlcNAc_2_:(AG12BsF6)
	26	78.263	5.97	**78.4**	**6.0**	6.9	GalGlcNAc_3_Man_3_FucGlcNAc_2_:(BIBsF6-G1)
	27	79.883	6.65	**80.0**	**6.7**	2.6	Gal_2_GlcNAc_3_Man_3_FucGlcNAc_2_:(BIBsF6)
A1	2	53.324	5.83	**55.3**	**6.0**	6.5	NeuAcGalGlcNAcMan_3_GlcNAc_2_:(6N-MO1)
	3-1	54.312	6.47	**56.2**	**6.6**	26.9	NeuAcGalGlcNAcMan_4_GlcNAc_2_:(6N-LnM4)
	3-2	54.312	7.24	**56.6**	**7.4**	55.8	NeuAcGalGlcNAcMan_5_GlcNAc_2_:(6N-LnM5)
	4-1	58.874	6.03	*61.2*	*5.9*	3.0	NeuAcGalGlcNAcMan_4_GlcNAc_2_:(3N-LnM4)
	4-2	58.874	6.74	**60.7**	**6.9**	9.0	NeuAcGalGlcNAcMan_5_GlcNAc_2_:(3N-LnM5)
	5	59.998	6.05	**61.7**	**6.1**	4.1	NeuAcGalGlcNAc_2_Man_3_GlcNAc_2_:(06N-BI-G2)
	6	61.064	8.07	*62.6*	*8.4*	2.4	NeuAcGal_3_GlcNAc_3_Man_3_FucGlcNAc_2_:(06N-TRF6)
	8-1	62.732	6.06	*62.9*	*6.1*	4.6	NeuAcGalGlcNAcMan_3_FucGlcNAc_2_:(6N-MO1F6)
	8-2	62.732	6.71	*64.9*	*6.5*	9.9	NeuAcGalGlcNAcMan_4_FucGlcNAc_2_:(6N-LnM4F6)
	8-3	62.732	7.42	**64.4**	**7.5**	3.1	NeuAcGalGlcNAcMan_5_FucGlcNAc_2_:(6N-LnM5F6)
	9	64.148	5.96	**67.2**	**5.7**	2.8	NeuAcGalGlcNAc_2_Man_3_GlcNAc_2_:(03N-BI-G2)
	11	67.625	5.89	*69.4*	*5.9*	2.5	NeuAcGalNAcGlcNAc_2_Man_3_FucGlcNAc_2_: [06N-BI(mLdn1)F6-G2]
	12	68.806	6.28	*71.0*	*6.4*	5.9	NeuAcGalGlcNAc_2_Man_3_FucGlcNAc_2_:(06N-BIF6-G2)
	13	70.422	6.93	**72.5**	**7.1**	4.0	NeuAcGal_2_GlcNAc_2_Man_3_FucGlcNAc_2_:(06N-BIF6)
	14-1	71.278	6.30	*74.4*	*6.4*	2.2	NeuAcGalGlcNAc_3_Man_3_GlcNAc_2_:(06N-BIBs-G2)
	14-2	71.278	7.61	**72.2**	**7.6**	2.7	NeuAcGal_3_GlcNAc_2_Man_3_FucGlcNAc_2_:(06N-Gaβ_4_2-BIF6)
	17-1	75.313	6.02	*76.6*	*5.9*	2.2	NeuAcGalGlcNAcMan_3_FucGlcNAc_2_:(6N-MO2F6)
	17-2	75.313	6.49	**76.4**	**6.6**	2.8	NeuAcGal_2_GlcNAc_2_Man_3_FucGlcNAc_2_:(30N-BIF6)
	18	76.811	5.57	**77.7**	**5.6**	2.9	NeuAcGalGlcNAcMan_3_FucGlcNAc_2_:(3N-MO2F6)
	19	79.040	6.08	*78.9*	*6.2*	3.4	NeuAcGalGlcNAc_3_Man_3_GlcNAc_2_:(60-BIBs-G1)
	20	81.076	6.55	**82.7**	**6.6**	3.8	NeuAcGalGlcNAc_3_Man_3_FucGlcNAc_2_:(06N-BIBsF6-G2)
	21	84.022	7.10	**86.2**	**7.2**	3.4	NeuAcGal_2_GlcNAc_3_Man_3_FucGlcNA_2_:(06N-BIBsF6)
	22	86.338	6.29	*89.1*	*6.5*	5.9	NeuAcGalGlcNAc_3_Man_3_FucGlcNAc_2_:(06-BIBsF6-G2)
A2	14-1	71.510	7.11	**75.2**	**7.3**	21.9	NeuAc_2_Gal_2_GlcNAc_2_Man_3_GlcNAc_2_:(66N-BI)
	14-2	71.510	7.83			2.1	NeuAc_2_Gal_3_GlcNAc_3_Man_3_FucGlcNAc_2_:(dN-TRF6)
	15	73.106	6.68	**76.5**	**6.9**	4.8	NeuAc_2_Gal_2_GlcNAc_2_Man_3_GlcNAc_2_:(36N-BI)
	16-1	75.619	8.04			2.2	NeuAc_2_Gal_3_GlcNAc_4_Man_3_FucGlcNAc_2_:(dN-TEF6-1G or dN-TRBsF6)
	16-2	75.619	8.61			3.4	NeuAc_2_Gal_4_GlcNAc_4_Man_3_FucGlcNAc_2_:(dN-TEF6)
	17	76.989	6.89	*79.8*	*7.1*	8.1	NeuAc_2_GalGalNAcGlcNAc_2_Man_3_FucGlcNAc_2_: [66N-BI(mLdn1)F6]
	18	77.890	7.32	**81.4**	**7.5**	19.2	NeuAc_2_Gal_2_GlcNAc_2_Man_3_FucGlcNAc_2_:(66N-BIF6)
	19	79.027	6.60	*82.0*	*6.8*	2.7	NeuAc_2_Gal_2_GlcNAc_2_Man_3_FucGlcNAc_2_: [66N-BI(dLdn1,2)F6]
	20	80.969	6.90	**84.2**	**7.1**	8.5	NeuAc_2_Gal_2_GlcNAc_2_Man_3_FucGlcNAc_2_:(36N-BIF6)
	23	85.992	6.54	**89.0**	**6.7**	5.0	NeuAc_2_Gal_2_GlcNAc_2_Man_3_FucGlcNAc_2_:(33N-BIF6)
	24	90.046	7.46			6.4	NeuAc_2_Gal_2_GlcNAc_3_Man_3_FucGlcNAc_2_:(dN-BIBsF6 or dN-TRF6-1G)
	25	93.339	6.92			5.1	NeuAc_2_Gal_2_GlcNAc_3_Man_3_FucGlcNAc_2_:(dN-BIBsF6 or dN-TRF6-1G)
	27	99.235	6.50			2.1	NeuAc_2_Gal_2_GlcNAc_2_Man_3_FucGlcNAc_2_:(dN-BIBsF6 or dN-TRF6-1G)
A3	10	77.082	8.11			2.9	NeuAc_3_Gal_3_GlcNAc_3_Man_3_FucGlcNAc_2_:(trN-TRF6)
	12	79.491	7.81			5.2	NeuAc_3_Gal_3_GlcNAc_3_Man_3_FucGlcNAc_2_:(trN-TRF6)
	13	81.419	5.59			2.8	SO_3_^−^-NeuAcGalNAc_2_GlcNAc_2_Man_3_FucGlcNAc_2_: [SO_3_^−^6N-BI(dLdn1,2)F6]
	14	82.500	7.40			2.9	NeuAc_3_Gal_3_GlcNAc_3_Man_3_FucGlcNAc_2_:(trN-TRF6)
	15-1	83.595	7.93			3.1	NeuAc_3_Gal_3_GlcNAc_3_Man_3_GlcNAc_2_:(trN-TR)
	15-2-1	83.595	8.52			1.2	NeuAc_3_Gal_3_GlcNAc_4_Man_3_FucGlcNAc_2_:(trN-TRBsF6 or trN-TEF6-1G)
	15-2-2	83.595	8.52			2.7	NeuAc_3_Gal_3_GlcNAc_4_Man_3_FucGlcNAc_2_:(trN-TRBsF6 or trN-TEF6-1G)
	15-3-1	83.595	8.95			1.5	NeuAc_3_Gal_4_GlcNAc_4_Man_3_FucGlcNAc_2_:(trN-TEF6)
	15-3-2	83.595	8.95			1.6	NeuAc_3_Gal_4_GlcNAc_4_Man_3_FucGlcNA_2_:(trN-TEF6)
	18	90.797	8.05			7.6	NeuAc_3_Gal_3_GlcNAc_3_Man_3_FucGlcNAc_2_:(trN-TRF6)
A4	6	88.863	8.88			2.1	NeuAc_4_Gal_4_GlcNAc_4_Man_3_FucGlcNAc_2_:(teN-TEF6)
	7	90.864	8.54			2.2	NeuAc_4_Gal_4_GlcNAc_4_Man_3_FucGlcNAc_2_:(teN-TEF6)

Possible structures and names of individual 79 N-glycans identified in the present study are listed with the R and S values of the strial samples (R_stria_ and S_stria_), the R and S values of the standard glycans (R_std_ and S_std_: Bold) or those calculated based on empirical additivity rule and MS data of the strial glycan (R_calc_ and S_calc_: Italic) and relative amounts [toward the amount of N-5 (Man_6_GlcNAc_2_, i.e. M6)]. Following data are described in Supplementary Table S2; peak areas of the glycan fractions obtained by size fractionation HPLC, total peak intensity of A3-15-2-1, A3-15-2-2, A3-15-3-1, and A3-15-3-2 in positive ion mode MALDI-QIT-TOF-MS analysis, the *m/z* values detected in LC-ESI-MS spectrum, the *m/z* values described in mass databases, the ion types detected in LC-ESI-MS analysis and symbolic images of the glycan structures.

All the 88 glycans were subjected to liquid chromatography with positive ion mode electrospray ionisation mass spectrometry (LC-ESI-MS) and MS^2^ analyses (Fig. 3). In the former, because the original samples extracted through reversed-phase HPLC were analysed, multiple peaks that showed different glycan types were detected in the spectra in many cases (Supplementary Fig. S2). Elution profiles of the products in the size fractionation HPLC enabled us to roughly predict the molecular mass of each eluted glycan^29^. On the basis of this information, among the fractions obtained in the MS analysis, the ones that represented the aforementioned 88 glycans were selected and then examined with MS^2^ (Supplementary Fig. S2). This series of experiments revealed that in 75 glycans, the reducing ends consist of Man_3_GlcNAc_2_, the core structure of N-glycans (Table 1 and Supplementary Tables S1 and S2)^30^. We additionally identified Man_2_GlcNAc_2_ and its mono-fucosylated derivative, both of which are also categorised into the N-linked type (Table 1 and Supplementary Tables S1 and S2)^30,31^. These 77 N-glycans were subjected to further analyses (Fig. 3b). On the other hand, 11 glycans, which did not contain Man_3_GlcNAc_2_, Man_2_GlcNAc_2_, or its mono-fucosylated derivative, were characterised as follows (Supplementary Fig. S2 and Table S3). Four glycans (A2-1, A2-3, A3-3, and A4-1) were found to bear reducing ends composed of N-acetylgalactosamine and therefore they manifested themselves as the O-linked type. Five species (A2-2-1, A2-2-2, A2-4, A2-5, and A4-2) were likely to be degradation products of some original glycans. As for each remainder (N-11-2 and N-14-1), the molecular mass predicted from the signals of the size fractionation HPLC could not be explained by the MS data.

In general, when a glycan is analysed with LC-ESI-MS and MS^2^, the spectra provide monosaccharide composition and partial glycosidic linkage patterns. This information, on some occasions, allows us to predict multiple structure types of the glycan. Next, to determine the detailed profiles of the 77 N-linked glycans obtained from the stria vascularis, we attempted to use their R and S values^29,32^ (Fig. 3a). These indexes stem from measured elution time of each glycan in reversed-phase HPLC and that in size fractionation HPLC, respectively^29,32^ (see ‘Methods’). In addition, 194 standard glycans, which are available from commercial or other resources, have been examined by the two HPLC types, and their R and S values (R_std_ and S_std_) can be obtained from the literature^7,28^ (Supplementary Table S4). With these values, we proceeded to the following four steps for each of the 77 N-glycans from the stria vascularis. Firstly, among the 194 standard glycans, species that have structure(s) identical to a predicted one or multiple structure(s) of a strial glycan were identified. Secondly, the R and S values obtained from the HPLCs’ data of the strial glycan (R_stria_ and S_stria_; see above) were compared to set(s) of the R_std_ and S_std_ values of the standard glycan(s) assigned at the first step. Thirdly, error factors (error R_std_ and error S_std_) between the values of the strial glycan and those of each of the corresponding standard glycans were calculated by means of the formulas:

1 $${\rm{error}}\,{{\rm{R}}}_{{\rm{std}}}=|\frac{{{\rm{R}}}_{{\rm{stria}}}-{{\rm{R}}}_{{\rm{std}}}}{{{\rm{R}}}_{{\rm{std}}}}|\times 100$$

and

2 $${\rm{error}}\,{{\rm{S}}}_{{\rm{std}}}=|\frac{{{\rm{S}}}_{{\rm{stria}}}-{{\rm{S}}}_{{\rm{std}}}}{{{\rm{S}}}_{{\rm{std}}}}|\times 100$$

Finally, we assigned the standard glycan, which provided both error factors of <5, to the strial glycan. In accordance with these criteria, the structures of 42 strial glycans were determined, as presented in Table 1 and Supplementary Tables S1 and S2 (see also Fig. 3b and Supplementary Table S5). Nevertheless, among these glycans, the assignment of A3-18 [NeuAc_3_Gal_3_GlcNAc_3_Man_3_FucGlcNAc_2_ (333N-TRF6)] may not be correct because the information on standard glycans that have more than two sialyl residues is limited.

Next, the rest of the strial N-linked glycans (35 species; see Fig. 3b) were analysed via the following four steps (Fig. 3a). Firstly, even for each of these glycans, the composition and partial linkage patterns were determined by the LC-ESI-MS and MS^2^ analyses (Supplementary Fig. S2); on the basis of these data, we extracted one or multiple constituent(s) that could also be identified in the library of 194 standard glycans (see Supplementary Table S4). Secondly, R_std_ and S_std_ values of these assigned constituent(s) were combined with ‘partial elution times’ of the remaining residues. These indexes are the values that are derived and obtained from the types and linkage patterns of the residues in accordance with the ‘empirical additivity rule’^7^. Collectively, in these two processes, we could not only predict structure(s) of a strial glycan but also calculate the R and S values (i.e. R_calc_ and S_calc_, respectively). Furthermore, in some cases, multiple linkage patterns were predictable for one strial glycan; therefore, different sets of R_calc_ and S_calc_ values were available. Thirdly, error factors (error R_calc_ and error S_calc_) between the R and S values of the strial glycan (i.e. aforementioned R_stria_ and S_stria_ values) and each set of the R_calc_ and S_calc_ values were obtained via the following equations:

3 $${\rm{error}}\,{{\rm{R}}}_{{\rm{calc}}}=|\frac{{{\rm{R}}}_{{\rm{stria}}}-{{\rm{R}}}_{{\rm{calc}}}}{{{\rm{R}}}_{{\rm{calc}}}}|\times 100$$

and

4 $${\rm{error}}\,{{\rm{S}}}_{{\rm{calc}}}=|\frac{{{\rm{S}}}_{{\rm{stria}}}-{{\rm{S}}}_{{\rm{calc}}}}{{{\rm{S}}}_{{\rm{calc}}}}|\times 100$$

Finally, we concluded that a candidate that showed two error factors of <5 corresponds to the strial glycan. This series of assays determined the structures of 14 strial glycans (Fig. 3b, Table 1, and Supplementary Tables S1, S2 and S5).

After that, 21 strial glycans remained to be characterised, due to a lack of partial elution times of the constituents (Fig. 3b, Table 1, and Supplementary Tables S1 and S2). In this context, although it was clear that some non-reducing ends in N-16-2 and N-21-2 consist of N-acetylhexosamines, whether each of these ends is GlcNAc or GalNAc was uncertain. In N-22-2, localisation of galactose at the non-reducing end could not be determined. Each of N-10, N-15, and N-16-1 species had a mannose residue that was acetylated. This modification is ordinarily never detected in organisms; in the present study, it was likely to artificially take place in the re-N-acetylation process of the liberated strial glycans (see ‘Methods’). Because neither R and S values of an acetylated standard glycan nor the empirical additivity rule for acetylation are available, the structures of the three glycans mentioned above were elusive. Each of the other 15 strial glycans (A2-14-2, A2-16-1, A2-16-2, A2-24, A2-25, A2-27, all the A4 species, and A3 species except A3-18), all of which are sialylated, also contains the linkage(s) whose elution times are unknown. Nevertheless, these glycans and A3-18 species, which were, as mentioned above, assigned to NeuAc_3_Gal_3_GlcNAc_3_Man_3_FucGlcNAc_2_ (333N-TRF6), were next subjected to profiling of sialyl linkage patterns (Fig. 3a). At the first step, original reversed-phase HPLC fractions that contained these 16 glycans were alkylamidated with isopropylamine (iPA) and thereafter with methylamine (MA). In this process, iPA binds specifically to α2,6-linked sialic acid, whereas MA is attached to α2,3-linked sialic acid^33^. After that, the samples were analysed by matrix-assisted laser desorption/ionisation quadrupole ion trap time-of-flight mass spectrometry (MALDI-QIT-TOF MS) in positive ion mode; derivatisation with iPA and MA increases the molecular mass of a glycan by 41.06 Da and 13.03 Da, respectively, as compared to the mass determined by LC-ESI-MS^33^. The combination of this sialic-acid-linkage–specific alkylamidation (SALSA) and mass spectrometry revealed that A3-15-2 species, which was separated as a sole glycan via the three different HPLCs, was composed of two glycan types: one that contains two α2,3-linked sialic acid residues and an α2,6-linked sialic acid residue (i.e. A3-15-2-1), and the other that has an α2,3-linked sialic acid residue and two α2,6-linked sialic acid residues (i.e. A3-15-2-2; Fig. 3b, Table 1, and Supplementary Fig. S3 and Tables S1, S2 and S6). This was also the case for A3-15-3 species, which are referred to as A3-15-3-1 and A3-15-3-2 (Table 1 and Supplementary Fig. S3 and Tables S1, S2 and S6). Because of detection of these two additional species, 79 N-linked glycan types in total were listed in the library of the stria vascularis (Fig. 3b, Table 1, and Supplementary Tables S1 and S2). Furthermore, the method using SALSA and positive ion mode MALDI-QIT-TOF MS indicated that A3-18 species had an α2,3-linked sialic acid residue and two α2,6-linked sialic acid residues although all the three linkages were shown by the analyses with R_std_ and S_std_ values to be the α2,3-type [i.e. NeuAc_3_Gal_3_GlcNAc_3_Man_3_FucGlcNAc_2_ (333N-TRF6); Supplementary Fig. S3 and Table S4]. This observation also indicates that the initially determined structure was invalid; in this context, the linkage pattern between three antenna structures (each of which contains α2,3-linked or α2,6-linked sialic acid) and the core structure became uncertain (Table 1 and Supplementary Tables S1 and S2). Finally, characterised structures of the other glycans are illustrated in Table 1 and Supplementary Tables S1 and S2 (see also Supplementary Fig. S3 and Table S6).

In summary, in the stria vascularis, we identified the complete structures of 55 N-linked glycans and assigned partial structures (along with full profiles of the monosaccharide composition) to the other 24 species (Fig. 3b, Table 1, and Supplementary Tables S1 and S2). As for A3-13 species that belongs to the latter category, the molecular mass determined by positive ion mode MALDI-QIT-TOF MS after SALSA differed by 1095.49 Da from the molecular mass indicated by the combination of LC-ESI-MS and MS^2^ (Supplementary Figs S2–S4 and Table S6); this marked inconsistency cannot be explained by any effects of alkylamidation of the three sialic acid residues. Accordingly, the derivatised sample was next profiled by negative ion mode MALDI-TOF-MS^n^, as described below (also see Fig. 3).

### Examples of structural analysis of strial N-glycans

**Figure 5:**
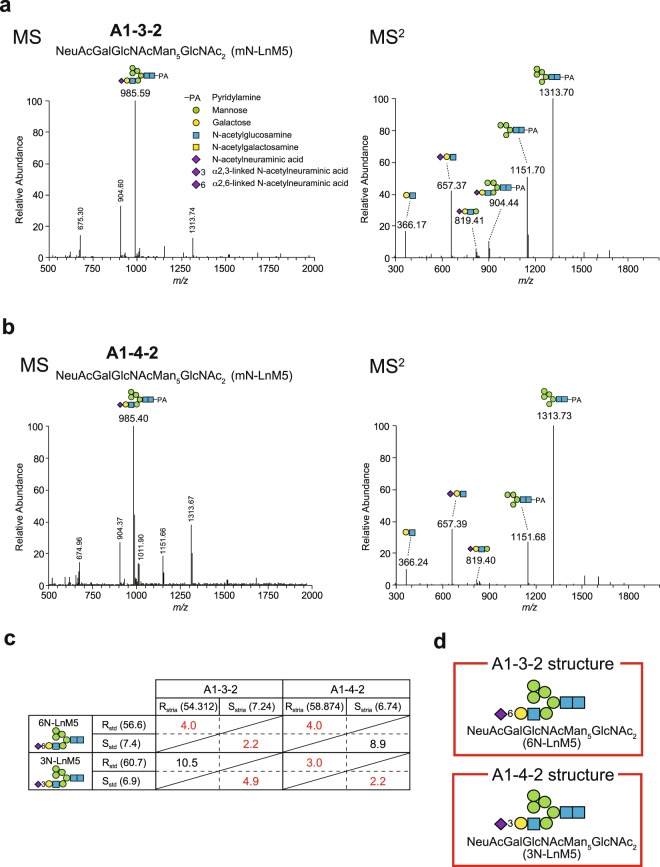
Structural analyses of A1-3-2 and A1-4-2 glycans. (**a**,**b**) *Left panels* illustrate full-scan mass spectra (*m/z* range: 500–2000) with A1-3 and A1-4 fractions eluted via reversed-phase HPLC (see Fig. 4b). *Right panels* depict signals of A1-3-2 and A1-4-2 analysed in MS^2^ mode. In these and subsequent spectra, symbolic notation above the peaks indicates the composition and linkage patterns of the products. Annotated structure of the glycans NeuAcGalGlcNAcMan_5_GlcNAc_2_ (mN-LnM5) is also shown in the *left panels*. (**c**) Comparison of R and S values obtained in the analyses of the strial glycans with three different HPLCs (R_stria_ and S_stria_ for A1-3-2 and A1-4-2 species) to those of standard glycans (R_std_ and S_std_) of 6N-LnM5 and 3N-LnM5 (Supplementary Table S4). These R and S values are indicated in parentheses. Shown in the *table* are error R_std_ and S_std_ factors in each pair. Procedures to obtain all the values and factors are described in the *main text*. The results highlighted in *red* satisfy the criterion of the glycan assignment (<5; see *text*). (**d**) Determined structures of A1-3-2 and A1-4-2 glycans. PA: pyridylamine.

In some cases, analysis of glycans with R and S values reveals the linkage patterns that cannot be addressed with mass spectrometry alone. Representative cases are A1-3-2 and A1-4-2 species, which were characterised with LC-ESI-MS and MS^2^ analyses and the R_std_ and S_std_ values (Fig. 5 and see Fig. 3). A difference in structure between these two strial N-glycans was limited to the linkage pattern of a single sialic acid residue (Table 1 and Supplementary Tables S1 and S2). In the MS spectrum, the A1-3-2 species was detected as a peak at *m/z* 985.59 [M + 2 H]^2+^ (Fig. 5a). This glycan was separated by MS^2^ into six remarkable fragments; HexHexNAc (*m/z* 366.17 [M + H]^+^), NeuAcHexHexNAc (*m/z* 657.37 [M + H]^+^), NeuAcHex_2_HexNAc (*m/z* 819.41 [M + H]^+^), NeuAcHex_5_HexNAc_3_-PA (m/z 904.44 [M + 2H]^2+^), Hex_4_HexNAc-PA (*m/z* 1151.70 [M + H]^+^), and Hex_5_HexNAc_2_-PA (*m/z* 1313.70 [M + H]^+^; Fig. 5a and Supplementary Table S7). These observations implied that species A1-3-2 is NeuAcGalGlcNAcMan_5_GlcNAc_2_ (mN-LnM5). At this stage, it remained uncertain whether the glycan is 3N-LnM5 that has an α2,3-linked sialic acid residue or 6N-LnM5 that contains an α2,6-linked sialic acid residue. Such prediction was also the case for A1-4-2 species (Fig. 5b). R_std_ and S_std_ values of 3N-LnM5 are 60.7 and 6.9, respectively, whereas those of 6N-LnM5 are 56.6 and 7.4, respectively (Supplementary Table S4). Each set of these values was compared to R_stria_ and S_stria_ values of the two strial glycans (Fig. 5c). Calculation of error R_std_ and S_std_ values using equations (1) and (2) and the aforementioned criterion revealed that A1-3-2 and A1-4-2 species are 6N-LnM5 and 3N-LnM5, respectively, as shown in Fig. 5c,d (see also Table 1 and Supplementary Tables S1 and S2).

**Figure 6:**
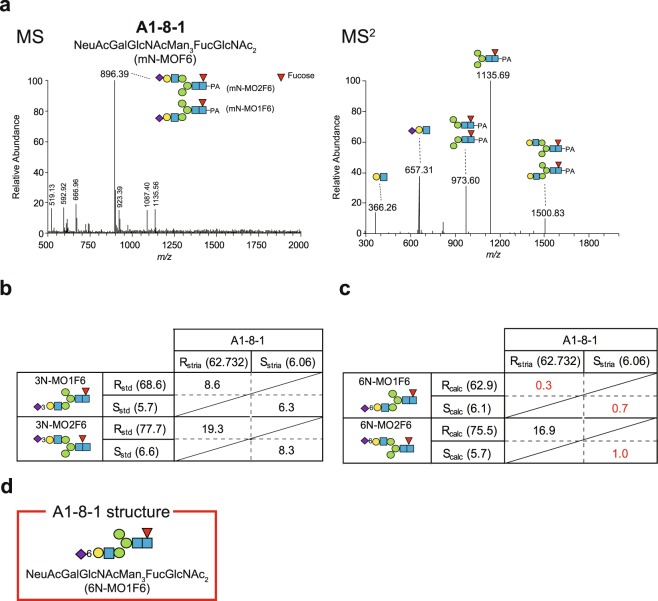
Determination of A1-8-1 glycan structure. (**a**) Mass spectra of A1-8-1 species. The profile of the A1-8 fraction analysed by mass spectrometry (MS) is shown in the *left panel*. The analysed fraction was isolated by reversed-phase HPLC (see Fig. 4b). Strial glycan A1-8-1 was detected as the signal at *m/z* 896.59 [M + 2 H]^2+^, and this fragment was next assayed with MS^2^ as displayed in the *right panel*. In both *panels*, monosaccharide composition and possible glycosidic linkage patterns for each product and annotated structure of A1-8-1 [NeuAcGalGlcNAcMan_3_FucGlcNAc_2_ (mN-MOF6)] are illustrated with symbols. (**b**,**c**) Characterisation of the structure of the A1-8-1 glycan. R and S values obtained through the analysis of the strial glycans with three different HPLCs (R_stria_ and S_stria_) were compared with R and S values of standard glycans (R_std_ and S_std_) 3N-MO1F6 and 3N-MO2F6 (Supplementary Table S4). (**b**) as well as calculated R and S values of 6N-MO1F6 and 6N-MO2F6 (R_calc_ and S_calc_) (**c**). The R and S values are shown in parentheses. Error R_std_ and S_std_ factors and error R_calc_ and S_calc_ factors in each pair were obtained, and the results are depicted in the *tables* in (**b**) and (**c**). Procedures for obtaining all the values and factors are described in the *main text*. The results indicated in *red* meet the criterion of the glycan assignment (<5; see *text*). (**d**) The candidate structure for A1-8-1 species. PA: pyridylamine.

The next example is a strial glycan whose structure was characterised with the R_calc_ and S_calc_ values (Fig. 6). As illustrated in Fig. 6a, the A1-8-1 species was detected as a marked signal at *m/z* 896.39 [M + 2 H]^2+^ in the LC-ESI-MS spectra. Analysis of this fraction in MS^2^ mode detected five significant peaks, each of which corresponded to HexHexNAc (*m/z* 366.26 [M + H]^+^), NeuAcHexHexNAc (*m/z* 657.31 [M + H]^+^), Hex_2_HexNAc_2_dHex-PA (*m/z* 973.60 [M + H]^+^), Hex_3_HexNAc_2_dHex-PA (*m/z* 1135.69 [M + H]^+^), or Hex_4_HexNAc_3_dHex-PA (*m/z* 1500.83 [M + H]^+^; Fig. 6a and Supplementary Table S7). Accordingly, fraction A1-8-1 was likely to be made up of NeuAcGalGlcNAcMan_3_FucGlcNAc_2_, i.e. mN-MOF6. Regarding the structure of this glycan, four candidates can be proposed because isomers of NeuAcGalGlcNAcMan_3_GlcNAc_2_ (MOF6) exist (MO1F6 and MO2F6; Supplementary Table S4) and a galactose residue in each isomer can bind to a sialic acid with α2,3-linkage (3N-MO1F6 or 3N-MO2F6) or with α2,6-linkage (6N-MO1F6 or 6N-MO2F6). For 3N-MO1F6 and 3N-MO2F6, R_std_ and S_std_ values were available (Supplementary Table S4). Comparison of each pair of these values with R_stria_ and S_stria_ values of A1-8-1 indicated that none of the sets of error R_std_ and S_std_ values satisfied the criterion ‘<5’ as depicted in Fig. 6b [see equations (1) and (2)]. To assign the strial glycan to 6N-MO1F6 or 6N-MO2F6, we obtained their R_calc_ and S_calc_ values via equations (3) and (4) because of a lack of their R_std_ and S_std_ values (for details, see Fig. 6c). On the basis of calculated error R_calc_ and S_calc_ values and the aforementioned criterion, the A1-8-1 species is potentially 6N-MO1F6 but not 6N-MO2F6 (Fig. 6c,d).

**Figure 7:**
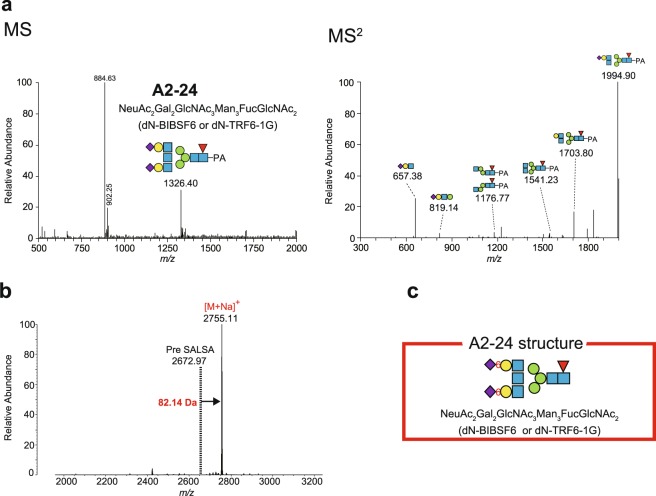
Characterisation of A2-24 glycan structure with sialic acid derivatisation. (**a**) Mass spectra of A2-24 species. The *left panel* shows the result of LC-ESI mass spectrometry (MS) analysis of the A2-24 fraction isolated by reversed-phase HPLC (see Fig. 4b). The product detected as a peak at *m/z* 1326.12 [M + 2 H]^2+^ was subjected to MS^2^ analysis and separated into several fragments as illustrated in the *right panel*. Constituents and possible linkage patterns for some products are also illustrated with symbols. NeuAc_2_Gal_2_GlcNAc_3_Man_3_FucGlcNAc_2_ (dN-BIBsF6 or dN-TRF6-1G) is annotated structure of strial glycan A2-24 (*left panel*). Note that the peak at *m/z* 884.63 in the *left panel* represents tri-protonated form [M + 3 H]^3+^ of the A2-24 glycan. (**b**) A full-scan positive ion mode MALDI-QIT-TOF mass spectrum (*m/z* > 2000) of the A2-24 fraction with sialic-acid-linkage–specific alkylamidation (SALSA). The strong signal at *m/z* 2755.11 [M + Na]^+^ represents the derivatised A2-24 species. The *dotted line* denotes the predicted position of the non-alkylamidated A2-24 glycan in sodiated form [M + Na]^+^ on the basis of its calculated *m/z* value (2672.97 marked by ‘Pre SALSA’). The difference in molecular mass between these two signals is shown (82.14 Da). (**c**) Assigned structure of the A2-24 strial glycan. Note that the linkage patterns among three N-acetylglucosamine residues in antenna structures and two mannose residues in the core structure have yet to be determined. PA: pyridylamine.

In the experiment shown in Fig. 7, SALSA derivatisation was necessary to clarify the sialyl linkage (see Fig. 3). Species A2-24, which was initially found to possess two sialic acid residues in the HPLC assays (Fig. 4), appeared as a signal at *m/z* 1326.40 [M + 2 H]^2+^ in the LC-ESI-MS spectra (Fig. 7a). This parent ion was next separated by MS^2^ analysis into six major moieties, which were detected as the signals at *m/z* 657.38 [M + H]^+^ (NeuAcHexHexNAc), *m/z* 819.14 [M + H]^+^ (NeuAcHex_2_HexNAc), *m/z* 1176.77 [M + H]^+^ (Hex_2_HexNAc_3_dHex-PA), *m/z* 1541.23 [M + H]^+^ (Hex_3_HexNAc_5_dHex-PA), *m/z* 1703.80 [M + H]^+^ (Hex_4_HexNAc_4_dHex-PA), and *m/z* 1994.90 [M + H]^+^ (NeuAcHex_4_HexNAc_4_dHex-PA; Fig. 7a and Supplementary Table S7). These observations predicted NeuAc_2_Gal_2_GlcNAc_3_Man_3_FucGlcNAc_2_ (dN-BIBsF6 or dN-TRF6-1G) as the structure of the A2-24 glycan; in this situation, the linkage pattern between three N-acetylglucosamine residues in antenna structures and two mannose residues in the core structure remained obscure (Fig. 7a and Supplementary Table S7). Moreover, each of the two sialic acid residues can bind to any one of the two galactose residues in the antenna structures. These unresolved issues stem from unavailability of R_calc_ and S_calc_ values owing to a lack of partial elution times related to the N-acetylglucosamines and sialic acid residues. Therefore, the linkage patterns of sialic acid residues were analysed with the combination of SALSA and positive ion mode MALDI-QIT-TOF MS (see Fig. 3). The spectrum showed a clear-cut peak at *m/z* 2755.11 [M + Na]^+^ (Fig. 7b; and see Supplementary Fig. S3 and Table S6). The monoisotopic *m/z* [M + Na]^+^ value of the A2-24 species without SALSA was calculated on the basis of the data from the LC-ESI-MS and MS^2^ analyses and was found to be 2672.97 (Fig. 7b). The mass difference (82.14 Da) between these two fractions matched molecular mass of two iPAs (single iPA; 41.06 Da). Accordingly, it was concluded that the glycan carries two α2,6-linked sialic acid residues (Fig. 7c, Table 1, and Supplementary Tables S1 and S2; see also Supplementary Table S6).

**Figure 8:**
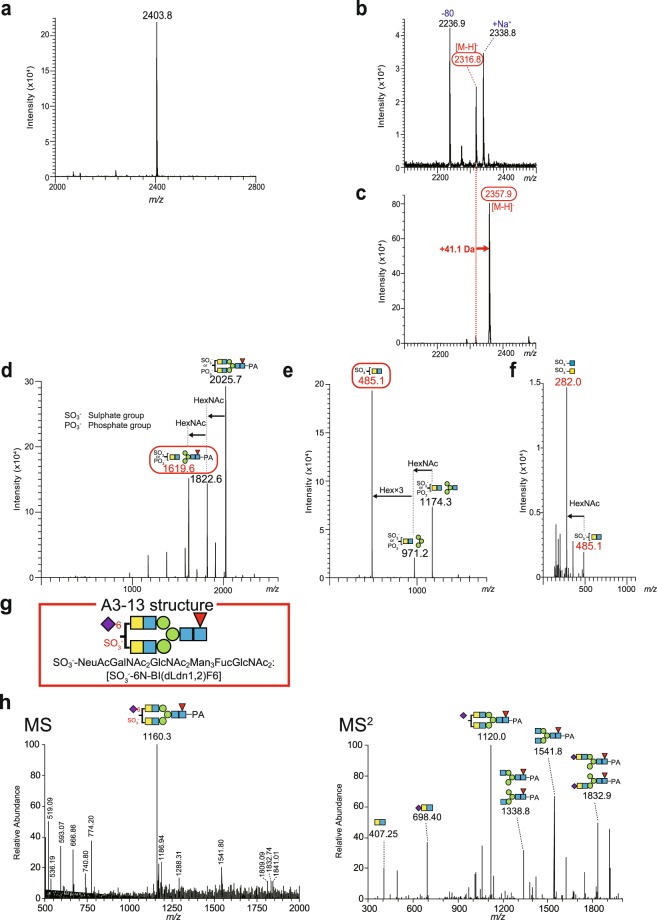
Analysis of A3-13 glycan structure with SALSA and multiple mass spectrometry modes. (**a**) A positive ion mode MALDI-QIT-TOF mass spectrum of A3-13 species. The sample analysed in this experiment was obtained by reversed-phase HPLC and derivatised with SALSA (see Supplementary Fig. S3). (**b**,**c**) Negative ion mode MALDI-QIT-TOF MS spectra of the reversed-phase HPLC fraction containing A3-13 species. The data on the samples without and with SALSA are displayed in panels b and c, respectively. In (**b**), the peaks at *m/z* 2316.8 [M-H]^−^ and at *m/z* 2338.8 [M-H]^−^ represent a deprotonated ion of the A3-13 strial glycan and its Na^+^-bound form, respectively, whereas the moiety (of the former) deficient in an acidic functional group is observed as a signal at *m/z* 2236.9 [M-H]^−^. Note that the deprotonated glycan (**b**) shifted by 41.1 Da with SALSA (**c**). (**d**) An MS^2^ spectrum of SALSA-derivatised A3-13 glycan isolated by the MS assay (a peak at *m/z* 2357.9 [M-H]^−^ in (**c**). A signal at *m/z* 2025.7 [M-H]^−^ should result from neutral loss of a single isopropylamine (iPA)-bound sialic acid in the parent ion. The lack of one and two HexNac residues likely produced two fractions at *m/z* 1822.6 [M-H]^−^ and 1619.6 [M-H]^−^, respectively. (**e**) Data from the MS^3^ analysis of the product at *m/z* 1619.6 [M-H]^−^ in MS^2^ mode. The signal at *m/z* 1174.3 [M-H]^−^ stems from loss of the pyridylamine (PA)-bound GlcNAc and Fuc in the parent ion. The difference in *m/z* between this signal and other two peaks (*m/z* 971.2 [M-H]^−^ and *m/z* 485.1 [M-H]^−^) can be explained by a loss of HexNAc or a loss of both HexNAc and three Hex residues, respectively, as shown in the panel. As described in the *main text*, the fraction at *m/z* 485.1 [M-H]^−^ consists of GalNAcGlcNAc modified by a sulphate group. (**f**) The MS^4^ spectrum obtained from the parent ion detected as a signal at *m/z* 485.1 [M-H]^−^ in MS^3^ mode. Difference in *m/z* value between the parent ion and the product at *m/z* 282.0 [M-H]^−^ corresponds to the *m/z* value of a HexNAc residue. Composition of the moiety should be sulphated GalNAc or GlcNAc (see *symbolic notations*). (**g**) Possible structure of A3-13. This glycan has two GalNAc residues; however, which one is attached with a sialic acid or a sulphate group remains to be determined. (**h**) Mass spectra of A3-13 species (see also Supplementary Fig. S2). The *left panel* shows the result of LC-ESI MS analysis with the A3-13 fraction isolated through reversed-phase HPLC (see Fig. 4b). The fragment at *m/z* 1160.3 [M + 2 H]^2+^ was next subjected to a surgical MS^2^ assay, and the result is shown in *right panel*. Constituents and possible linkage patterns described above several peaks in both panels are based on the conclusion that A3-13 strial glycan is sulphated NeuAcGalNAc_2_GlcNAc_2_Man_3_FucGlcNAc_2_ [SO_3_^−^-6N-BI(dLdn1,2)F6] (see **g**). PA: pyridylamine.

The final case is species A3-13 whose characterisation required more complicated analyses (Fig. 8; see also Fig. 3). This strial glycan, which was originally found to have three sialic acid residues by the analyses with three different HPLC types (see Fig. 4a), appeared as a peak at *m/z* 1160.29 [M + 3 H]^3+^ in the LC-ESI-MS spectra (Supplementary Fig. S4). Subsequent MS^2^ analysis detected signals at *m/z* 844.17 and *m/z* 1120.01 and predicted that both of these molecular fragments contained a pseudo-Lewis X structure, i.e. GalNAc(GlcNAc)Fuc (Supplementary Table S7). These results suggested that the A3-13 glycan is NeuAc_3_GalNAc_2_GalGlcNAc_3_Fuc_2_Man_3_FucGlcNAc_2_ [trN-d(LdnF)-mLn-M3F6] whose molecular mass is 3477.30 Da. On the other hand, when the sample separated by reversed-phase HPLC was processed with SALSA and assayed with positive ion mode MALDI-QIT-TOF MS, the A3-13 species was detected as a strong peak at *m/z* 2403.8 [M-H + 2Na]^+^ (Fig. 8a). This observation indicated that molecular mass of this glycan is 2381.8 Da. The difference between the mass values obtained in these two series of analyses was not explained by SALSA derivatisation. To more precisely profile the A3-13 glycan, we carried out the following experiments. Firstly, the corresponding fraction isolated via reversed-phase HPLC was subjected to negative ion mode MALDI-QIT-TOF MS without SALSA. This analysis identified a signal at *m/z* 2316.8 [M-H]^−^ (Fig. 8b). Then, the original sample was treated with SALSA and assayed by the same MS type, visualizing a peak at *m/z* 2357.9 [M-H]^−^ (Fig. 8c). The difference of 41.1 Da in molecular mass between these two deprotonated ions [M-H]^−^ is identical to molecular mass of one iPA (41.06 Da). Accordingly, species A3-13 is likely to contain an α2,6-linked sialic acid residue. Of note, in the MS spectrum of the A3-13 sample without SALSA (Fig. 8b), two other peaks at *m/z* 2338.8 [M-H]^−^ and at *m/z* 2236.9 [M-H]^−^ were detected. The former is likely to represent addition of Na^+^ to the original glycan observed as the peak at *m/z* 2316.8 [M-H]^−^. This result indicates that the A3-13 species remained to be deprotonated even when an acidic functional group included in this glycan was neutralised by Na^+^ binding; in other words, this glycan has multiple acidic functional groups. In addition, even though a sialic acid in the A3-13 species was neutralised by SALSA, this glycan remained to be a deprotonated form [M-H]^−^ in negative ion mode MALDI-QIT-TOF MS (Fig. 8c). Taken together, the A3-13 species is likely to harbour at least one acidic functional group that is different from carboxyl group of the sialic acid. In this context, in the MS spectrum obtained from the sample without SALSA (Fig. 8b), the peak at *m/z* 2236.9 [M-H]^−^ was smaller by approximately 80 Da than the native signal of A3-13 species (*m/z* 2316.8 [M-H]^−^) and the difference is consistent with molecular mass of a sulphate group or a phosphate group. Accordingly, it is plausible that this strial glycan consists of NeuAcGalNAc_2_GlcNAc_2_Man_3_FucGlcNAc_2_ [6N-BI(dLdn1,2)F6)] (molecular mass: 2237.86) to which either acidic functional group is attached. Furthermore, with MS^2^ mode we analysed the SALSA-treated fraction, which was detected as a product at *m/z* 2357.9 [M-H]^−^ in negative ion mode MALDI-QIT-TOF MS (Fig. 8c; and see Fig. 3). As shown in Fig. 8d, three marked peaks were detected; the one at *m/z* 2025.7 [M-H]^−^ should result from neutral loss of single iPA-bound sialic acid in the parent ion. Additional lack of one and two HexNAc residue(s) (203.08 Da for one) is likely to result in the other two peaks at *m/z* 1822.6 [M-H]^−^ and *m/z* 1619.6 [M-H]^−^, respectively. The latter seems to represent a glycan of GalNAcGlcNAcMan_3_FucGlcNAc_2_ (LdnM3F6) that is sulphated or phosphorylated (Supplementary Table S7). Analysis of this fraction with MS^3^ assay produced multiple signals (Fig. 8e); difference in molecular mass between a fragment at *m/z* 1174.3 [M-H]^−^ and the parent ion (*m/z* 1619.6 [M-H]^−^) was likely due to loss of the PA-bound GlcNAc and Fuc at the reducing end. Moreover, it seems probable that the fragment at *m/z* 971.2 [M-H]^−^ was produced by deletion of one HexNAc residue (203.08 Da) from the glycan detected at *m/z* 1174.3 [M-H]^−^ whereas the moiety at *m/z* 485.1 [M-H]^−^ resulted from additional loss of three Hex residues (Fig. 8e). The value of *m/z* 485.1 [M-H]^−^ can be accounted for by molecular mass of GalNAcGlcNAc (406.16 Da) that is modified by a sulphate group or a phosphate group (~80 Da; Fig. 8e). According to the literature^34^, Gal and HexNAc in N-glycans can be sulphated but not phosphorylated (but see ‘Discussion’). Therefore, we concluded that the functional group in GalNAcGlcNAc is likely to be a sulphate group. Next, the aforementioned fraction with *m/z* 485.1 [M-H]^−^ (GalNAcGlcNAc) was analysed with MS^4^ and a peak at *m/z* 282.0 [M-H]^−^ was detected in the spectrum (Fig. 8f). This *m/z* value seems to represent a combination of the mass of a sulphate group (79.96 Da) and that of GalNAc; alternatively, it may denote GlcNAc (203.08 Da; Supplementary Table S7). It is only logical that a sulphate group is attached to the non-reducing terminus of a glycan^35^; this modification should be the case for the strial A3-13 glycan comprising NeuAcGalNAc_2_GlcNAc_2_Man_3_FucGlcNAc_2_ [6N-BI(dLdn1,2)F6] (Fig. 8g). This conclusion means no inconsistency for the signal detected at *m/z* 1160.29 in ESI-MS and MS^2^ spectra (Fig. 8h and also see Supplementary Fig. S2) when this fraction served as a doubly protonated ion [M + 2 H]^2+^ instead of a triply protonated form [M + 3 H]^3+^ that was initially predicted.

### Profiles of strial N-glycans

**Figure 9:**
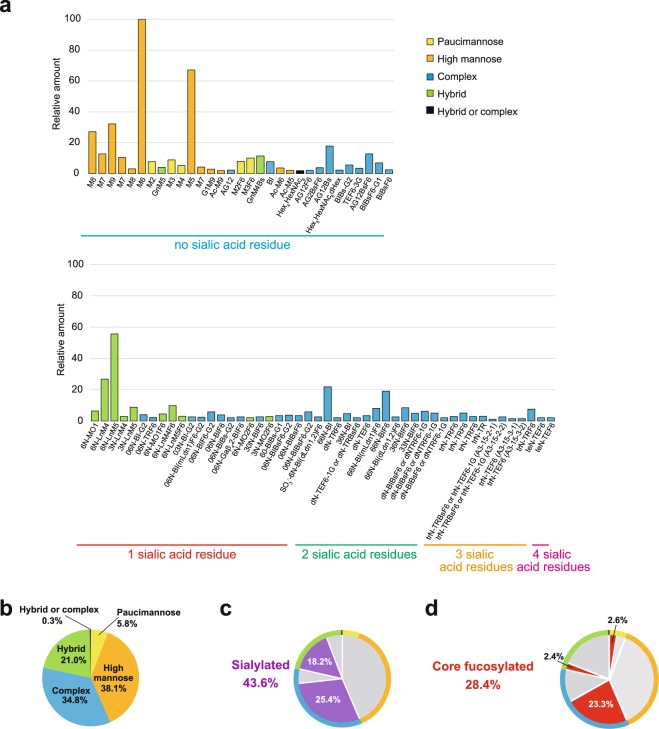
A profile of N-glycans in stria vascularis. Strial 79 N-glycan types determined by the workflow shown in Fig. 3 are listed in (**a**). In this *panel*, the amount of each glycan was normalised to that of N-5 (Man_6_GlcNAc_2_, i.e. M6), which is the most abundant among all the glycan species. *Yellow*, *orange*, *blue*, and *green bars* indicate paucimannose, high-mannose, complex, and hybrid type glycans. Structure of the glycan marked by *black bar* belongs to either complex or hybrid type (see *text*). Data on these 79 glycans are derived from Table 1 and Supplementary Tables S1 and S2. The numbers of sialic acid residues attached to glycans are also shown below the names. Pie graphs in (**b**–**d**) describe populations of paucimannose (*yellow*), high-mannose (*orange*), complex (*blue*), and hybrid (*green*), and hybrid or complex type (*black*) glycans (**b**) and proportions of sialylated (*purple*) and core-fucosylated (*red*) glycans (**c** and **d**, respectively). For comparison, the proportion of each of the four glycan types (see **b**) is also shown at the edges of graphs (**c** and **d**).

We finally categorised all the 79 strial N-linked glycans in accordance with their structural profiles as follows. Major vertebrate N-glycans can be categorised into three groups, i.e. high-mannose, complex, and hybrid types, all of which have a common core glycan chain of Man_3_GlcNAc_2_. Each type has a different additional sequence as follows^30^. Firstly, in the high-mannose type, the core is coupled to chains of various lengths composed purely of mannose residues. Secondly, the complex type harbours ‘antennae’ that are initiated by N-acetylglucosaminyltransferases and contain a different combination of other monosaccharides such as N-acetylglucosamine, galactose, fucose, N-acetylgalactosamine, and sialic acid^30^. Thirdly, in the hybrid type, the chain(s) of only mannose(s) (i.e. high-mannose-type residues) and the complex-type ‘antennae’ are both bound to the core. In addition to these three groups, some strial glycans belong to paucimannose species (Man_1–4_GlcNAc_2_Fuc_0–1_), which does not contain more than four mannoses and any antenna structures (Table 1 and Supplementary Tables S1 and S2)^30,31^. This type is common in invertebrates and plants but was recently detected in vertebrates as well^31,36,37^. The 79 strial N-glycans were subjected to this categorisation, and we present in Fig. 9a their amounts normalised to the amount of fraction N-5 (Man_6_GlcNAc_2,_ i.e. M6). On the basis of this characterisation, we obtained the proportion of each of the four structural types among the 79 glycans. As illustrated in Fig. 9b, the high-mannose type was the most abundant and accounted for 38.1% of the total amount of strial glycans. Complex and hybrid types represented 34.8% and 21.0%, respectively. The least abundant variety was the paucimannose type, which represented 5.8% of the total. As mentioned above, in N-16-2 (GalGlcNAcHexNAc_3_Man_3_GlcNAc_2_), it was unclear whether each of the three HexNAc residues that constitute some non-reducing ends is GlcNAc or GalNAc (Table 1 and Supplementary Tables S1 and S2). Therefore, we could not conclude whether this glycan belongs to the hybrid or complex type. On the other hand, it seems probable that N-21-2 belongs to the complex type, owing to the identified monosaccharide composition at the non-reducing ends (see Table 1 and Supplementary Tables S1 and S2). Sialylation can occur in the hybrid or complex type but not in the high-mannose or paucimannose type^38^. In the stria vascularis, 43.6% of the total amount of N-glycans had single or multiple sialic acid residues; complex and hybrid types constituted 25.4% and 18.2%, respectively (Fig. 9c). Finally, core fucosylation was detected in 28.4% of the total amount of N-glycans, i.e. 2.6%, 23.3%, and 2.4% were the paucimannose type, complex type, and hybrid type of glycans, respectively (Fig. 9d).

## Discussion

The cochlear stria vascularis, which is essential for hearing, has affinity for some lectin types such as concanavalin A, ulex europaeus agglutinin I, and wheat germ agglutinin^39–41^. Although these observations indicate that this tissue expresses different glycan types, the detailed profile remains unclear. In the present study, a combination of different analytical approaches detected 79 N-linked glycans in the stria vascularis and determined their relative amount (Table 1, Fig. 9a, and Supplementary Tables S1 and S2). In this process, we identified the complete structures of 55 strial glycans and determined partial glycosidic linkage patterns and full monosaccharide composition in the other 24 glycans (Fig. 3b). To our knowledge, such detailed analysis of sugar chains in the stria has not been carried out to date.

The method composed of three HPLC types and LC-ESI-MS is relatively simple and is also used for different samples to analyse their glycans^7,28^. Nonetheless, this approach seems to have limitations; in particular, it cannot perfectly distinguish sialyl linkage isomers in some cases. In the present study, we additionally treated a few samples with SALSA and clearly distinguished α2,3-linked and α2,6-linked sialic acid residues by mass spectrometry (Figs 3, 7 and 8). Accordingly, the workflow combining this derivatisation and MS^n^ as described in Fig. 3 is an effective and sensitive procedure for characterisation of glycan structures.

A possible application of the glycan library obtained here is to complement other studies as follows. Mumps virus infects the stria vascularis and causes deafness^42^. *In vitro* experiments show that neuraminidase in the virus strongly binds unbranched-type NeuAc-Gal-Glucose or NeuAc-Gal-GlcNAc that have the α2,3 sialyl linkage in glycans^43^. Nevertheless, whether proteins in the stria vascularis harbour either sugar chain has remained elusive. In our list of 79 strial glycans (Table 1 and Supplementary Tables S1 and S2), the latter binding site is included in glycan A1-18-1. This evidence reinforces the current theory about the route of mumps infection and may contribute to elucidation of the pathological process of the deafness. Further comparison of the strial glycan structures shown in this study with different experimental results may expand the repertoire of uses of the library.

In the analysis of glycan A3-13, we concluded that the functional group in GalNAcGlcNAc is a sulphate group (Figs 3 and 8; Supplementary Fig. S2). Nevertheless, a possibility of phosphorylation instead of the sulphation cannot be ruled out completely. In O-glycans, LacdiNAc, which is composed of two HexNAc residues, can be phosphorylated^44,45^. The same modification may be identified in HexNAc of N-glycans by further study.

Although the methodologies shown in this work are effective at analysing glycan structures, they have several limitations. The first issue is related to liberation of sugar chains from glycoproteins (see ‘Methods’). Anhydrous hydrazine employed in our experiments can extract more divergent glycans regardless of their structures and protein types, as compared to enzymes including peptide N-glycosidase F. Nevertheless, the hydrazinolysis can cleave acyl groups from monosaccharides constituting glycans. Therefore, we re-*N*-acetylated the samples with acetic anhydride^46^. These procedures and modifications also affect all the sialic acid residues and prevent us from determining individual types such as N-acetyl, N-glycolyl, and O-acetyl derivatives.

The second limitation lies in the procedure for purification of strial glycans. Trifluoroacetic acid is commonly used to elute acidic glycans from a graphite carbon cartridge loaded with samples^47^. The reagent is strongly acidic. If the eluates will be next concentrated for some reason, then increased acidification may degrade sugar chains. In this study, the amount of tissues of the stria vascularis was expected to be small (see Fig. 1d), and therefore the samples were processed in a vacuum concentrator. To avoid excess acidification, we added no trifluoroacetic acid but instead ammonium acetate to elution buffer (final concentration: 50 mM), as described in the literature^7^ (see ‘Methods’). It is noteworthy that the increased salt concentration accelerates a release of numerous glycans from the cartridge but may be ineffective for collection of some highly acidic types.

Thirdly, because 194 standard glycans we used are primarily of the human or zebrafish type, extrapolation of these glycan data to *rat* strial glycans should be addressed carefully. Moreover, as presented in Fig. 3b, the structures of 24 strial N-glycans could not be completely determined by means of our procedures. To completely resolve these issues, analyses of the samples treated multiple times with different exoglycosidases by mass spectrometry or HPLC^48,49^ may be useful. This series of assays would require a considerable amount of a sample, and therefore this series was not incorporated here into the workflow for analysis of glycans from the stria vascularis, a tiny tissue (see Fig. 1d). In this context, further experiments may be necessary to validate our procedures.

## Methods

### Isolation of the stria vascularis

All the animal experiments were conducted in compliance with the protocol reviewed by the Institutional Animal Care and Use Committee, were approved by the President of Niigata University (Niigata Univ. Res. 215-2), and were compliant with the ARRIVE guidelines^50^. Male BN/SsNSIc rats (7 weeks old, 140–180 g; SLC, Hamamatsu, Japan) were used (64 rats total). All the animals were anaesthetised by intraperitoneal injection of sodium pentobarbital (0.5 ml per rat; Nembutal; Abbott, IL, USA). After deep anaesthesia was confirmed by the toe pinch, corneal reflexes, and respiratory rate, in each rat, the heart was surgically exposed and cold saline (100 ml) was infused systemically through the left ventricle (drainage; right atrium). Then, the cochleae were dissected from the temporal bone (Fig. 1b) and washed in the chilled standard solution consisting of NaCl, 140 mM; KCl, 5 mM; HEPES, 10 mM (pH 7.4); D-glucose, 10 mM; MgCl_2_, 1 mM; CaCl_2_, 1 mM; and cOmplete Protease Inhibitor Cocktail (1 tablet per 50 ml; Roche, Basel, Switzerland).

The stria vascularis and spiral ligament were isolated under a stereo-microscope as follows. Firstly, the cochlea was sagittally divided using a sharp scalpel, and the bony lateral wall, which contains the stria vascularis and spiral ligament, was manually separated from the organ of Corti and cochlear axis with fine tweezers (Fig. 1c). Secondly, the lateral cochlear wall consisting of the stria and ligament was detached from the bony wall, and the stria, which is identified by brown pigmentation of intermediate cells^51^ (Fig. 1c), was carefully peeled away from the ligament with a 27-gauge needle (Fig. 1d). Thirdly, the isolated tissues were gently washed with the standard solution. Five to eight pieces of the stria vascularis and ligament (length: 200–1300 μm) were obtained from one cochlea. Samples of respective tissues from 8–10 cochleae were collected in 1.5 ml microtubes and centrifuged at 11432 × *g* for 1 min at 4 °C (KITMAN-18; TOMY, Tokyo, Japan). After removal of the supernatant, the samples were frozen in liquid nitrogen and stored at −80 °C until use.

### qPCR analyses

Total-RNA samples were extracted from the stria vascularis and spiral ligament isolated from 8–10 cochleae using NucleoSpin RNA XS (TaKaRa Bio, Otsu, Japan). Concentration and quality of RNA were estimated on a 2100 BioAnalyzer (Agilent, Santa Clara, CA, USA); samples whose RNA integrity number exceeded 7.0 were admitted to subsequent experiments. The RNA was processed with Moloney Murine Leukaemia Virus (M-MULV) reverse transcriptase (Invitrogen, Carlsbad, CA). Real-time qPCR was carried out on a LightCycler Nano System (Roche). Expression levels of transcripts of four genes, i.e. *Slc12a6* (GenBank accession No. NM_001109630), *Pou3f4* (NM_017252), *Bsnd* (NM_138979), and *Cldn11* (NM_053457), which encode KCC3, Brn-4, barttin, and claudin 11, respectively, were compared between the stria vascularis and spiral ligament. The primer sequences have been described in our earlier work^12^. All the experiments were conducted in duplicate. Quantitative data from PCR with each set of primers were obtained with reference to an internal control gene: glyceraldehyde 3-phosphate dehydrogenase (*Gapdh*; GenBank accession No. NM_017008). The cDNA template resulting from 5 ng of total RNA was used for the assessment of expression of *Slc12a6*, *Pou3f4*, *Bsnd*, and *Cldn11* and that from 1 ng of total RNA was tested for *Gapdh*. This series of assays was carried out three times with different batches of cochlea samples: 26 cochleae from 13 rats were analysed in total. Each gene expression level was displayed as mean ± SE (n = 3), and differences between the stria vascularis and spiral ligament were evaluated by two-tailed Student’s *t* test.

### Preparation of pyridylaminated glycans from the stria vascularis

N-glycans were liberated from the strial glycoproteins by hydrazinolysis as described previously^29^. Pieces of strial tissues that were excised from 102 cochleae (51 rats) and stored separately in 14 tubes were combined in one tube. Next, the samples were lyophilised and heated at 100 °C for 10 h with 1 ml of anhydrous hydrazine (Tokyo Chemical Industry, Tokyo, Japan). After removal of this reagent by repeated evaporation, the glycans were re-*N*-acetylated with acetic anhydride in a saturated sodium bicarbonate solution and subsequently desalted by passing them through a Dowex 50Wx2 (H^+^) cation exchanger (Dow Chemicals, Midland, MI, USA). The samples were again lyophilised and then heated at 90 °C for 60 min with 20 µl of a pyridylamination reagent; this reagent was prepared by dissolving 2-aminopyridine (276 mg) in acetic acid (100 µl). Finally, they were heated at 80 °C for 35 min with 70 µl of a reducing reagent, which was a mixture of dimethylamine borate (50 mg), acetic acid (20 µl), and double-distilled water (12.5 µl). In this process, the glycans were tagged with a fluorophore, 2-aminopyridine^52,53^.

Pyridylaminated (PA)-glycans from the reaction mixture were purified as described elsewhere^7,54^. The reaction mixture was diluted with 0.15 ml of water and extracted twice using 1 ml of phenol:chloroform (1:1 v/v) to remove the excess reagents and contaminants. The water layer that contained the PA-glycans was purified by gel filtration on a column (1.5 × 18 cm, TSK-gel Toyopearl HW-40F, Tosoh, Tokyo, Japan) equilibrated with 10 mM ammonium acetate pH 6.0. After loading of the sample, the eluate between 10 and 25 ml was collected as the PA-glycan fraction. The PA-glycans were further purified using a graphite carbon cartridge (GL-Pak Carbograph 300 mg; GL Sciences Ltd, Tokyo, Japan). The salt concentration of the glycan mixture was adjusted to 50 mM with ammonium acetate, pH 6.0, and the mixture was loaded onto the cartridge. After a wash with 5 ml of 50 mM ammonium acetate, pH 6.0, the glycans were eluted with 5 ml of 60% acetonitrile in the ammonium acetate buffer. The eluate was concentrated by means of a vacuum concentrator and dried by lyophilisation.

Sample preparation described above was performed in accordance with MIRAGE guidelines^55,56^.

### HPLC

Three types of HPLC were carried out using a Waters Alliance Waters 2695 separation module and W2475 fluorescence detector (Waters, Milfold, MA) in accordance with MIRAGE guidelines^55,56^; the detailed information including the parameter settings are described below.

Firstly, the lyophilised strial PA glycans were dissolved in ultra-pure water and analysed at a flow rate of 1.0 ml/min by anion exchange HPLC combined with a TSKgel DEAE-5PW column (0.75 × 7.5 cm; Tosoh, Tokyo, Japan). The column was equilibrated with 0.7 mM aqueous ammonia, pH 9.0. After the samples were injected into the column, the concentration of ammonium acetate was increased linearly to 0.2 M between the time points 5 and 20 min, and then, was further elevated to 0.5 M during the following 10 min. The PA-glycans were detected on a fluorescence spectrophotometer at an excitation wavelength of 310 nm and an emission wavelength of 380 nm. By these procedures, the N-glycans were separated in accordance with the number of attached sialic acid residues, i.e. into fractions N, A1, A2, A3, and A4 (see Fig. 3a and the ‘Results’ section).

Secondly, each fraction (N, A1–A4) was subjected to reversed-phase HPLC, which involved a Cosmosil 5C18-P column (0.2 × 25 cm; Nacalai Tesque, Kyoto, Japan). The flow rate of the samples was 0.2 ml/min. The column was equilibrated with 100 mM triethylamine acetate, pH 4.0, containing 0.075% of 1-butanol. After injection of the sample, the 1-butanol concentration was increased linearly from 0.075% to 0.5% during 105 min. Negative charges of sialic acid residues interfere with separation of samples by reversed-phase HPLC; therefore, in our preparation, they were neutralised by triethylamine, an ion-pairing reagent that has both a positive charge and a hydrophobic moiety. This treatment sharpens the elution profile of acidic N-glycans^7^. The PA-glycans were detected at an excitation wavelength of 315 nm and an emission wavelength of 400 nm. Areas of all the individual peaks were measured separately^32^. The most abundant species was N-5. A peak whose area exceeded 2% of that of N-5 was considered significant; thus, 107 fractions were subjected to the next analysis.

Thirdly, the samples were processed by size fractionation HPLC involving a TSK gel Amide 80 column (0.46 × 7.5 cm; Tosoh, Tokyo, Japan) at a flow rate of 0.5 ml/min. The column was equilibrated with 50 mM ammonium formate, pH 4.4, containing 80% acetonitrile. After a sample was injected, acetonitrile concentration was decreased linearly from 80% to 65% during 5 min and then from 65% to 55% over the next 5 min, and finally from 55% to 30% during the subsequent 25 min. PA-glycans were detected using a fluorescence spectrophotometer at an excitation wavelength of 315 nm and an emission wavelength of 400 nm. The peak area of each glycan obtained by this HPLC was determined as follows. When multiple subfractions were extracted from a fraction of the reversed-phase HPLC, an area proportion of each subfraction was calculated. The area of the original fraction was multiplied by each proportion to determine areas of the subfractions. A glycan whose relative amount exceeded 2% of that of N-5 was considered significant; 88 species that satisfied this criterion were subjected to LC-ESI-MS and MS^2^ (see Fig. 3, Table 1, and Supplementary Tables S1, S2 and S3).

A glycan’s elution time obtained in reversed-phase HPLC was converted to R_stria_ by means of the reversed-phase scale, and that obtained in size fractionation HPLC was converted to S_stria_ with the glucose unit, as described elsewhere^29,32^.

### LC-ESI-MS and MS^2^ analysis

Mass-spectrometric analysis of PA-glycans was performed by positive-ion mode ESI-MS on an LTQ XL linear ion trap mass spectrometer coupled to a Dionex U3000 HPLC system (Thermo Scientific, San Jose, CA). The fractions isolated through reversed-phase HPLC were trapped on a Hypercarb guard cartridge column (1 × 10 mm; Thermo Scientific, Waltham, MA) for enrichment and separation from contaminants. The samples were eluted with 0.1% (v/v) formic acid in water and 0.5% formic acid in acetonitrile. The flow rate was 50 µl/min, and the gradient conditions were varied for different samples. MS^2^ analyses of PA-glycans were carried out by collision-induced dissociation in a data-dependent mode or with manually selected parent ion isolation. The peak intensities were extracted in the Mass+ + software ver. 2 (Shimadzu, Kyoto, Japan). All the MS and MS^2^ spectra are displayed in Supplementary Fig. S2.

Sample preparation and mass-spectrometric analyses described above were performed in accordance with MIRAGE guidelines^55,56^. Details of the HPLC, MS, and MS^2^ settings are given in Supplementary Table S8.

### SALSA and multiple MALDI-QIT-TOF mass spectrometry (MS^n^) analysis

Strial PA-glycan fractions obtained by reversed-phase HPLC were desalted by means of StageTip Carbon composed of Empore SPE carbon disks (Sigma-Aldrich, St. Louis, MO)^33^. The solvent was removed *in vacuo*. The glycans included in dried samples were derivatised in a linkage-specific manner in alkylamidation solutions containing iPA and MA^33^. The samples were dried and re-dissolved in 10 μl of ultra-pure and sterile water. After that, 1 μl of this solution was deposited on a 700 μm μ Focus MALDI plate (Hudson Surface Technology Inc., Old Tappan, NJ) and then mixed with 1 μl of a matrix solution. Composition of this solution is described in Supplementary Table S9. The plate was place on a 75 °C heat block for 1 min to accelerate solvent evaporation. On-plate PA-glycans were analysed by MALDI-QIT-TOF MS (AXIMA-Resonance, Shimadzu/Kratos, Manchester, UK) in positive or negative ion mode. MS^n^ analyses were conducted by collision-induced dissociation with manually selected parent ion isolation. The peak intensities were extracted using Shimadzu Biotech Launchpad ver. 2.9.3 (Kratos Analytical Ltd., Manchester, UK). Of note, the A3-15-2 glycan that was detected as a sole type by HPLC analyses was found to be composed of two species, i.e. A3-15-2-1 and A3-15-2-2, by the procedure combining SALSA and positive ion mode MALDI-MS (Table 1 and Supplementary Fig. S3 and Tables S1, S2 and S6). Relative amounts of each of these two species were calculated via the following three steps. Firstly, as usually detected in the results of mass spectrometry experiments, careful observation revealed that the SALSA derivatised glycan in positive ion mode MALDI-QIT-TOF MS was composed of multiple isotopic peaks; the peaks within 6 Da from the *m/z* value of the onset of the initial peak were taken as significant signals. All the data points in these significant peaks were analysed, and a total peak intensity was obtained (see ‘MALDI-MS total peak intensity’ in Supplementary Table S2). Secondly, using this value, the proportion of each glycan (i.e. A3-15-2-1 or A3-15-2-2) in the parent fraction, A3-15-2 species, was calculated. Finally, the relative amount of the glycan was determined by multiplying the proportion by the parent’s amount normalised to N-5. With the same procedure, the relative amounts of A3-15-3-1 and A3-15-3-2 species were calculated (Table 1 and Supplementary Tables S1 and S2). Finally, to one glycan (A3-13), MS^n^ analyses were then applied (Fig. 8 and Supplementary Fig. S3 and Table S6).

Sample preparation and mass spectrometry described above were carried out in accordance with MIRAGE guidelines^55,56^. Information on the MS and MS^n^ settings is shown in Supplementary Table S9.

## Supplementary information

Supplementary Materials

## Data Availability

All the data generated or analysed during this study are included in this published article or available from the corresponding author on reasonable request.
