# Supplementary material for: Characterisation of N-glycans in the epithelial-like tissue of the rat cochlea
Source: Sci Rep. 2019 Feb 7;9:1551. doi: 10.1038/s41598-018-38079-0 (PMC6367448; doi:10.1038/s41598-018-38079-0)
Supplement: Supplementary file 1 — Supplementary Materials [file 41598_2018_38079_MOESM1_ESM.pdf]

Supplementary Materials

Characterisation of N-glycans in the epithelial-like tissue of the rat cochlea

Yoriko Nonomura[1,2], Seishiro Sawamura[1], Ken Hanzawa[3], Takashi Nishikaze[4], Sadanori Sekiya[4], Taiga Higuchi[1], Fumiaki Nin[1,5], Satoru Uetsuka[6], Hidenori Inohara[6], Shujiro Okuda[7], Eiji Miyoshi[8], Arata Horii[2], Sugata Takahashi[2], Shunji Natsuka[3] & Hiroshi Hibino[1,5,9,*]

[1]Department of Molecular Physiology, Niigata University School of Medicine

[2]Department of Otorhinolaryngology–Head and Neck Surgery, Niigata University School of Medicine

[3]Department of Biology, Faculty of Science, Niigata University

[4]Koichi Tanaka Mass Spectrometry Research Laboratory, Shimadzu Corporation

[5]Center for Transdisciplinary Research, Niigata University

[6]Department of Otorhinolaryngology–Head and Neck Surgery, Graduate School of Medicine, Osaka University

[7]Bioinformatics Laboratory, Niigata University School of Medicine

[8]Division of Health Sciences, Graduate School of Medicine, Osaka University

[9]AMED-CREST, AMED, Niigata, Japan

[*]Corresponding author: *H Hibino*, Department of Molecular Physiology, Niigata University School of Medicine, 1-757 Asahimachi-dori, Chuo-ku, Niigata, Niigata 951-8510, Japan. Email: hibinoh@med.niigata-u.ac.jp, Tel.: +81 25-227-2071, Fax: +81 25-227-0460.

List of Supplementary Materials

・Supplementary Figure S1 **Chromatograms of size fractionation HPLC**

・Supplementary Figure S2 **LC-ESI-MS and MS^2 spectra of 88 strial glycans**

・Supplementary Figure S3 **Positive ion mode MALDI-QIT-TOF-MS spectra of SALSA-derivatised glycans**

・Supplementary Figure S4 **LC-ESI-MS and MS^2 spectra of A3-13 species**

・Supplementary Table S1 **Rules of abbreviations that represent the glycan structures**

・Supplementary Table S2 **Dataset of N-glycans obtained from the stria vascularis**

・Supplementary Table S3 **A list of species other than N-glycans**

・Supplementary Table S4 **Structures and R and S values of 194 standard glycans**

・Supplementary Table S5 **A list of error factors**

・Supplementary Table S6 **The *m/z* values for SALSA-derivatised glycans in positive ion mode MALDI-QIT-TOF-MS**

・Supplementary Table S7 **A list of N-glycan databases**

・Supplementary Table S8 **Parameter settings for LC-ESI-MS and MS^2 analyses**

・Supplementary Table S9 **Parameter settings for MALDI-QIT-TOF-MS^n analyses**

Legends for Supplementary Materials

Supplementary Figure S1. Chromatograms of size fractionation HPLC.

Shown are the results of analyses of 107 pyridylaminated strial glycan fractions, which were obtained via reversed-phase HPLC and size fractionation HPLC. The separated fraction whose peak area exceeded 2% of that of N-5 was assumed to be significant. The significant signals are labelled by the fraction numbers (88 species in total; see **Fig. 3**, **Table 1** and **Supplementary Table S1** and **S2**). In some chromatograms, multiple significant peaks were detected; these products are sub-numbered with reference to their elution times.

Supplementary Figure S2. LC-ESI-MS and MS^2 spectra of 88 strial glycans.

Upper panels illustrate full-scan LC-ESI-MS spectra (*m/z* range 500–2000) of 88 glycan fractions obtained by three different HPLCs (see **Fig. 3**). Retention times (RT) of the analysed glycans in the liquid chromatography coupled with mass spectrometry are also shown. The *m/z* values of the fractions subjected to MS^2 analyses are presented above the peaks. *Lower panels* display MS^2 spectra of the parent fractions. All the major peaks in the LC-ESI-MS and MS^2 spectra were annotated with the corresponding fragments. As for N-11-2 and N-14-1, the molecular mass predicted from the signals of the size fractionation HPLC could not be accounted for by the MS data. Therefore, these two fractions

were no longer analysed in MS^2 mode. Symbols: a filled *green circle*, mannose; filled *blue square*, N-acetylglucosamine; filled *yellow circle*, galactose; filled *yellow square*, N-acetylgalactosamine; filled *grey square*, N-acetylhexosamine (N-acetylglucosamine or N-acetylgalactosamine); *red* filled *triangle*, fucose; *blue* filled *circle*, glucose; *purple* filled *diamond*, sialic acid; Ac, acetyl group; SO_3^-, sulphate group; PA, pyridylamine.

Supplementary Figure S3. Positive ion mode MALDI-QIT-TOF MS spectra of SALSA-derivatised glycans.

Displayed are the SALSA-derivatised strial glycans in high-mass mode (*m/z* > 2000; see **Fig. 3**). Analysed glycan species as well as monoisotopic *m/z* values for major peaks are shown (see also **Supplementary Fig. S2** and **Table S2**). In the A3-15-2 fraction, two sialyl linkage isomers were identified; the same was true for fraction A3-15-3; the glycans were sub-numbered with reference to their *m/z* values (see *inset* in A3-15 spectrum).

Supplementary Figure S4. LC-ESI-MS and MS^2 spectra of A3-13 species.

The *left panel* illustrates a full-scan mass spectrum (*m/z* range 500–2000), with the A3-13 fraction eluted via reversed-phase HPLC. The *right panel* presents signals of the N-glycan analysed in MS^2

mode. These data are derived from the spectrum shown in **Supplementary Figure S2**. Symbolic notations above the peaks indicate the composition and linkage patterns of the products. Annotated structure of the glycan $NeuAc_3GalNAc_2GalGlcNAc_3Fuc_2Man_3FucGlcNAc_2$ [trN-d(LdnF)-mLn-M3F6] based on the MS and MS^2 data was found to be inconsistent with findings of the analysis of SALSA-derivatised A3-13 by positive-ion mode MALDI-QIT-TOF MS (see **Fig. 8**).

Supplementary Table S1. Rules of abbreviations that represent the glycan structures.

Details of the abbreviations used in this study are shown with graphical examples. Symbols: a filled *green circle*, mannose; filled *blue square*, N-acetylglucosamine; filled *yellow circle*, galactose; filled *yellow square*, N-acetylgalactosamine; *red* filled *triangle*, fucose; *blue* filled *circle*, glucose; *purple* filled *diamond*, sialic acid. The number between two sugar residues indicates a linkage type between these monosaccharides: the *black* letter, 'β' between two galactose residues shows a β1,4-linked form and the *black* letters, '3' and '6' between a sialic acid and a galactose moiety represent α2,3-bound and α2,6-bound forms, respectively.

Supplementary Table S2. Dataset of N-glycans obtained from the stria vascularis. The panel includes additional data of the strial N-glycans described in **Table 1**. Possible structures and names of

individual 79 N-glycans identified in the present study are listed with the R and S values of the strial samples (R_{stria} and S_{stria}), the R and S values of the standard glycans (R_{std} and S_{std}: *blue*) or those calculated based on empirical additivity rule and MS data of the strial glycan (R_{calc} and S_{calc}: *red*), areas of the fractions obtained by size fractionation HPLC, and relative amounts [toward the amount of N-5(M6B)]. For A3-15-2-1, A3-15-2-2, A3-15-3-1, and A3-15-3-2, total peak intensity of each glycan in positive ion mode MALDI-QIT-TOF-MS analysis was calculated for determination of the relative amount of these four glycans. Observed *m/z* values represent the *m/z* values detected in LC-ESI-MS spectrum (**Supplementary Fig. S2**) and calculated *m/z* values are the values described in mass databases (**Supplementary Table S7**). All the molecular ions were $[M+H]^+$, $[M+2H]^{2+}$, or $[M+3H]^{3+}$. Symbols: a filled *green circle*, mannose; filled *blue square*, N-acetylglucosamine; filled *yellow circle*, galactose; filled *yellow square*, N-acetylgalactosamine; filled *grey square*, N-acetylhexosamine (N-acetylglucosamine or N-acetylgalactosamine); *red* filled *triangle*, fucose; *blue* filled *circle*, glucose; *purple* filled *diamond*, sialic acid; Ac, acetyl group; SO_3^-, sulphate group. The number between two sugar residues indicates a linkage type of these monosaccharides: the *black* letter, 'β' between two galactose residues shows a β1,4-linked form and the *black* letters, '3' and '6' between a sialic acid and a galactose or a N-acetylgalactosamine moiety, represent α2,3-bound and α2,6-bound forms that were determined by only R and S values, respectively, whereas the numbers highlighted by

red indicate the sialyl isomers distinguished by means of SALSA and MALDI-MS analysis.

Supplementary Table S3. A list of species other than N-glycans.

Possible structures and names of 11 non-N-linked glycans identified in the present study. R and S values of the strial samples (R_{stria} and S_{stria}), peak areas of the fractions obtained by size fractionation HPLC, and relative amounts toward the amount of N-5 (M6) are described for each glycan fraction. Observed *m/z* values on the basis of mass spectrometry data and calculated *m/z* values in accordance with the mass database (see **Supplementary Table S7**) are also shown. All the molecular ions are $[M+H]^+$. In the cases of N-11-2 and N-14-1, the molecular mass predicted from the signals of size fractionation HPLC could not be explained by the MS data. Therefore, for these fractions, neither the *m/z* value nor assigned structure were displayed. Symbols: filled *blue square*, N-acetylglucosamine; filled *yellow circle*, galactose; filled *yellow square*, N-acetylgalactosamine; filled *grey square*, N-acetylhexosamine (N-acetylglucosamine or N-acetylgalactosamine); *grey* filled *circle*, hexose (mannose or galactose); *purple* filled *diamond*, sialic acid. The number between two sugar residues shows a linkage type between these monosaccharides. The *black* letters, ‘3’ and ‘6’ between a sialic acid and a galactose or a N-acetylgalactosamine moiety, represent α2,3-bound and α2,6-bound forms that were determined only by R and S values, respectively.

Supplementary Table S4. Structures and R and S values of 194 standard glycans.

Information on the 194 standard glycans used for characterisation of strial N-glycans is presented. R and S values (R_{std} and S_{std}) were measured in our earlier studies [Natsuka *et al*. (2014) *PLoS One*. **9**, e102219 and Hanzawa *et al*. (2017) *Glycobiology.* **27**, 228-245]. Monosaccharide symbols: an open *circle*, galactose; filled *grey circle*, mannose; filled *black circle*, glucose; open *square*, N-acetylgalactosamine; filled *black square*, N-acetylglucosamine; *grey* filled *triangle*, fucose; open star, xylose; *grey* filled *diamond*, sialic acid; *black* half-filled *diamond*, glucuronic acid; PA, pyridylamine. All the displayed data were obtained with permission from the literature [Table S1 from '*Improved method for drawing of a glycan map, and the first page of glycan atlas, which is a compilation of glycan maps for a whole organism.*' (Natsuka *et al*. [2014] *PLoS One*. **9**, e102219 under the Creative Commons Attribution licence) and Table 1 from '*Structures and developmental alterations of N-glycans of zebrafish embryos.*' (Hanzawa *et al*. [2017] *Glycobiology.* **27**, 228-245) with permission of Oxford University Press (https://academic.oup.com/glycob/article/27/3/228/2656954?searchresult=1)].

Supplementary Table S5. A list of error factors.

Values of error factors (error R_{std} and S_{std} highlighted in *blue* and error R_{calc} and S_{calc} indicated by *red*) used for determination of structures of 55 strial N-glycans (see **Fig. 3**) are shown. In this context, the related R and S values are also provided.

Supplementary Table S6. The *m/z* values for SALSA-derivatised glycans in positive ion mode MALDI-QIT-TOF-MS.

For each species, the displayed multiple compositions and their calculated *m/z* values were obtained from the data on LC-ESI-MS and MS^2 spectra shown in **Supplementary Figure S2** (see also **Supplementary Table S7**). The observed *m/z* value was derived from positive ion mode MALDI-QIT-TOF-MS spectra of SALSA-derivatised samples described in **Supplementary Figure S3**. The candidate whose calculated *m/z* value matches the observed *m/z* value is expected to represent the virtual composition of the glycan and is marked by *red*. Note that all the molecular ions were either $[M+H]^+$ or $[M-H+2Na]^+$.

Supplementary Table S7. A list of N-glycan databases.

Shown are the MS number and *m/z* values for different glycans of asialo, monosialo, disialo, trisialo, and tetrasialo species in the forms of $[M+H]^+$, $[M+Na]^+$, $[M+2H]^{2+}$, and $[M+3H]^{3+}$ ions. The values of

B-type glycosidic fragments are also described. Composition: PA, pyridylaminated; M3PA, $Man_3GlcNAc_2$-PA; Hex, hexose; HexNAc, N-acetylhexosamine; dHex, deoxy-hexose; Pen, pentose; NANA, N-acetylneuraminic acid. Modification: Me, methylation; Ac, acetylation; HPO_3, phosphorylation; SO_3, sulphation.

Supplementary Table S8. Parameter settings for LC-ESI-MS and MS^2 analyses.

Information on the HPLC as well as the LC-ESI-MS and MS^2 analyses is displayed.

Supplementary Table S9. Parameter settings for MALDI-QIT-TOF-MS^n analyses.

Information on the positive and negative ion mode MALDI-QIT-TOF MS and MS^n assays is presented.

Supplementary Figure S1-1

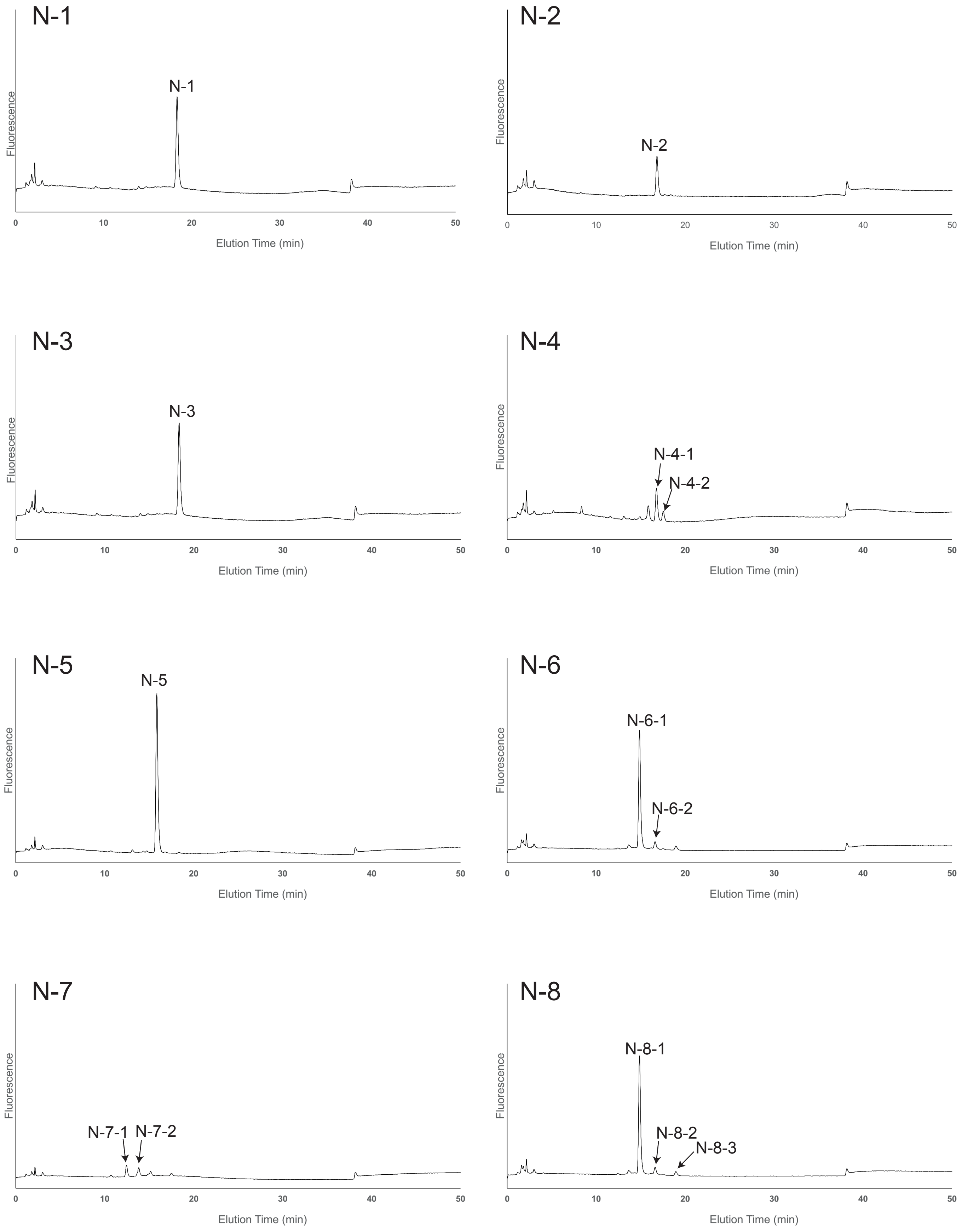

Supplementary Figure S1-2

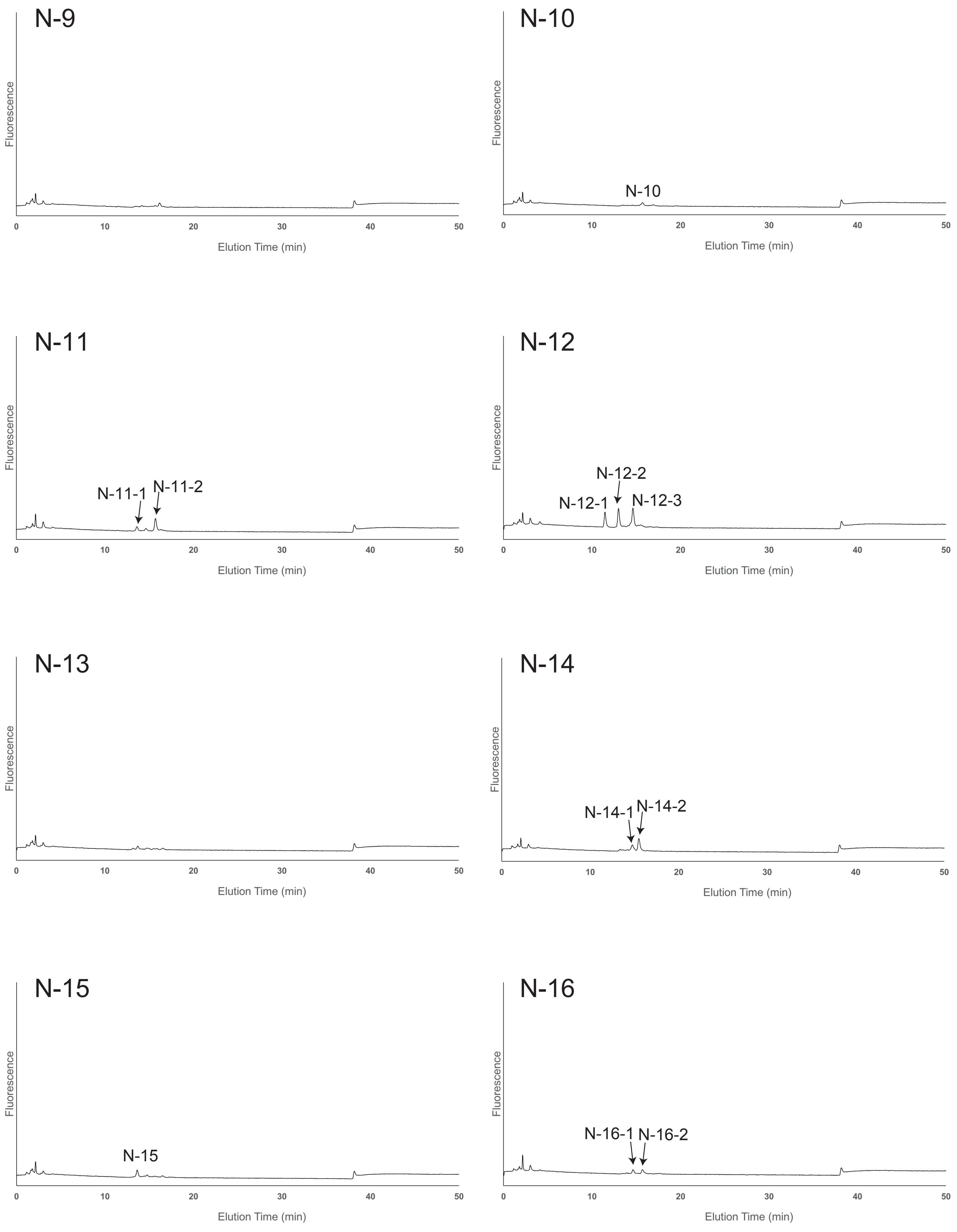

Supplementary Figure S1-3

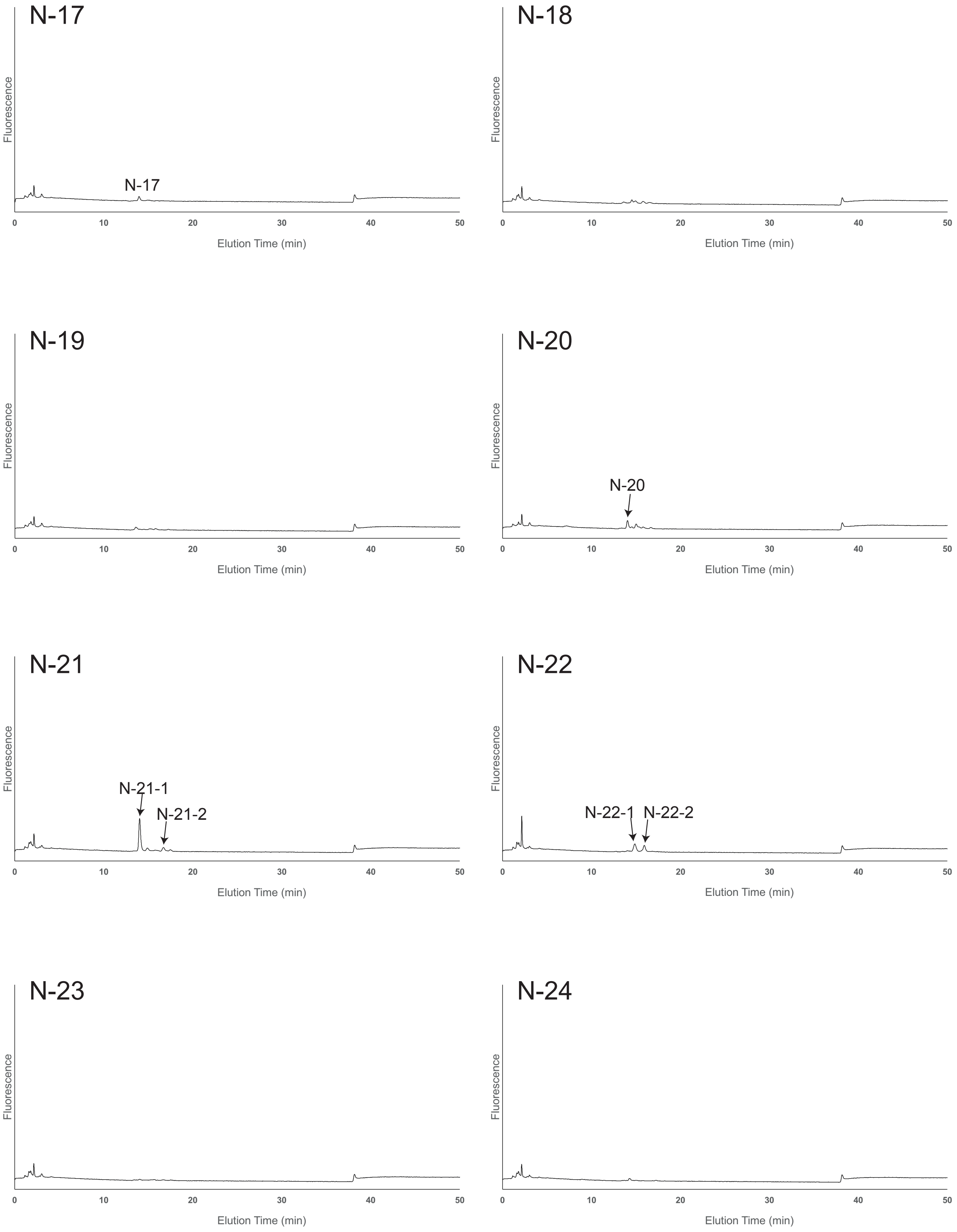

Supplementary Figure S1-4

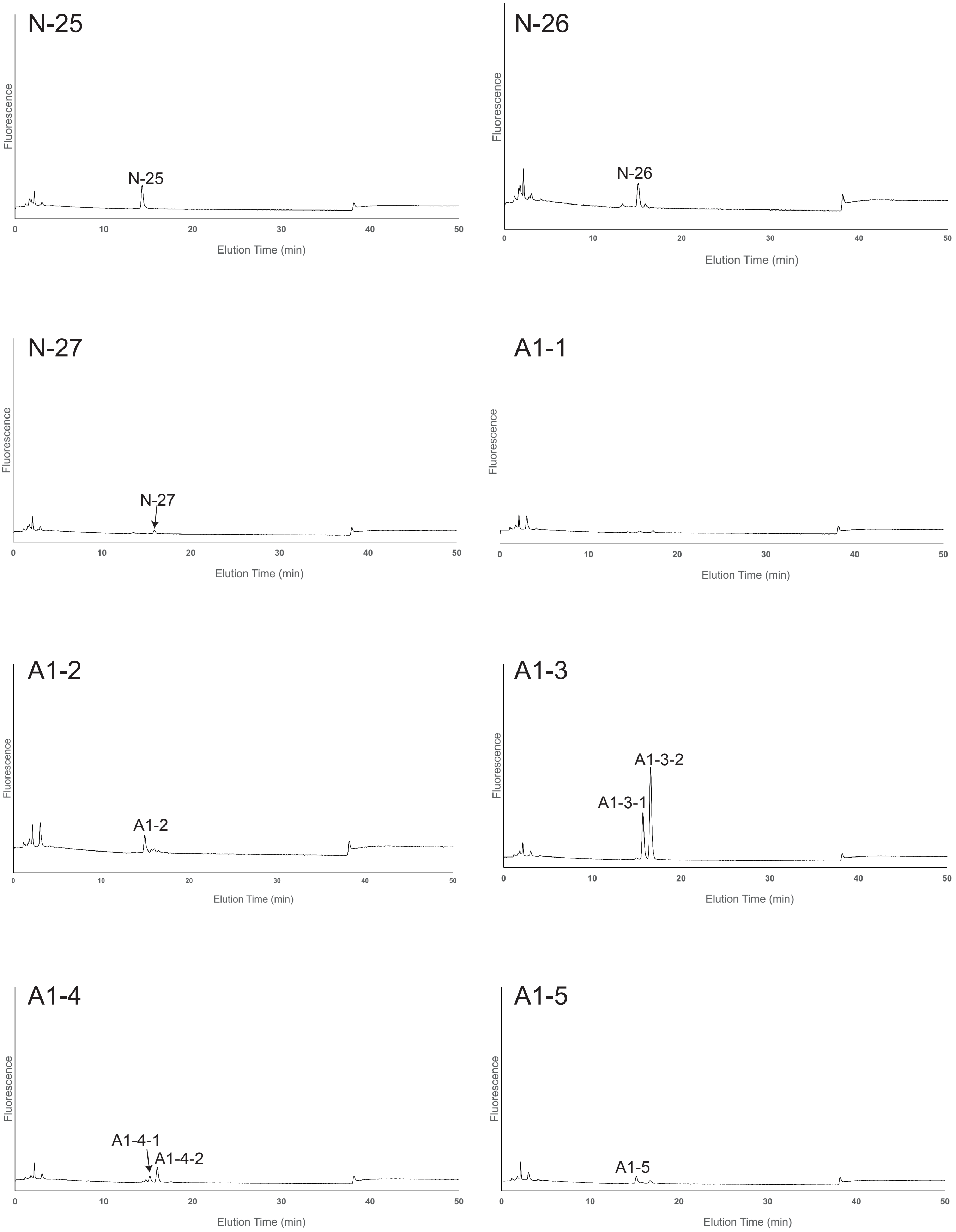

Supplementary Figure S1-5

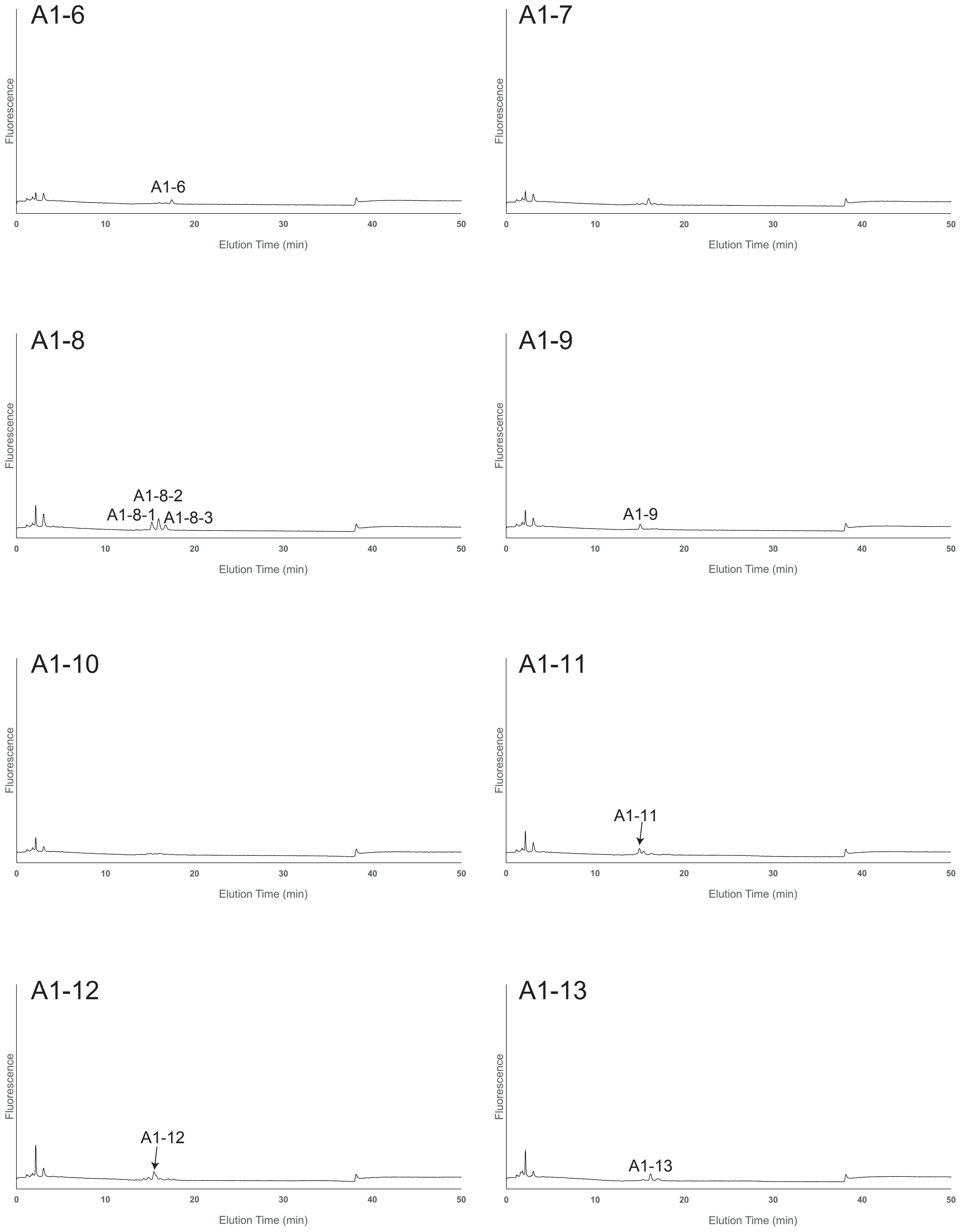

Supplementary Figure S1-6

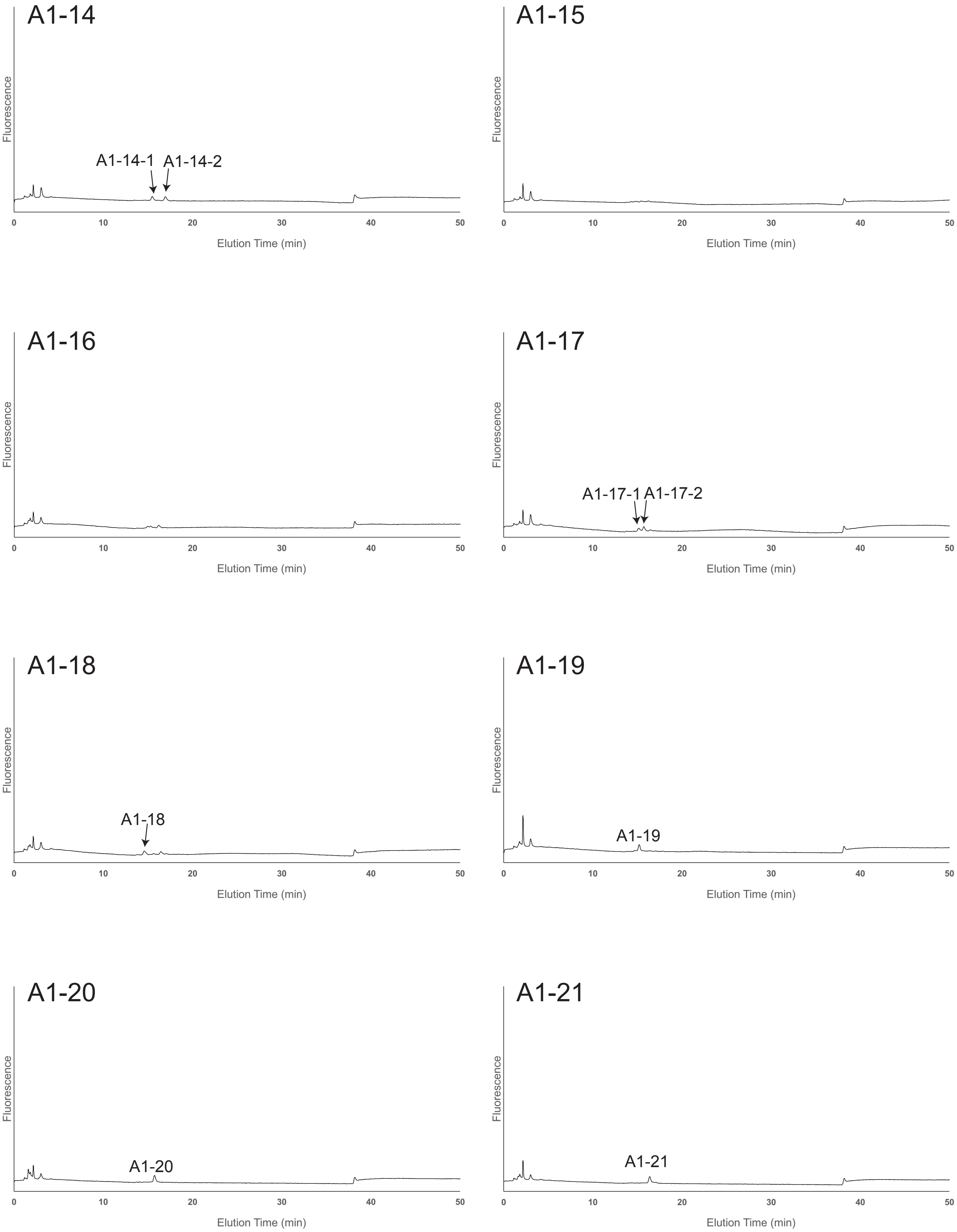

Supplementary Figure S1-7

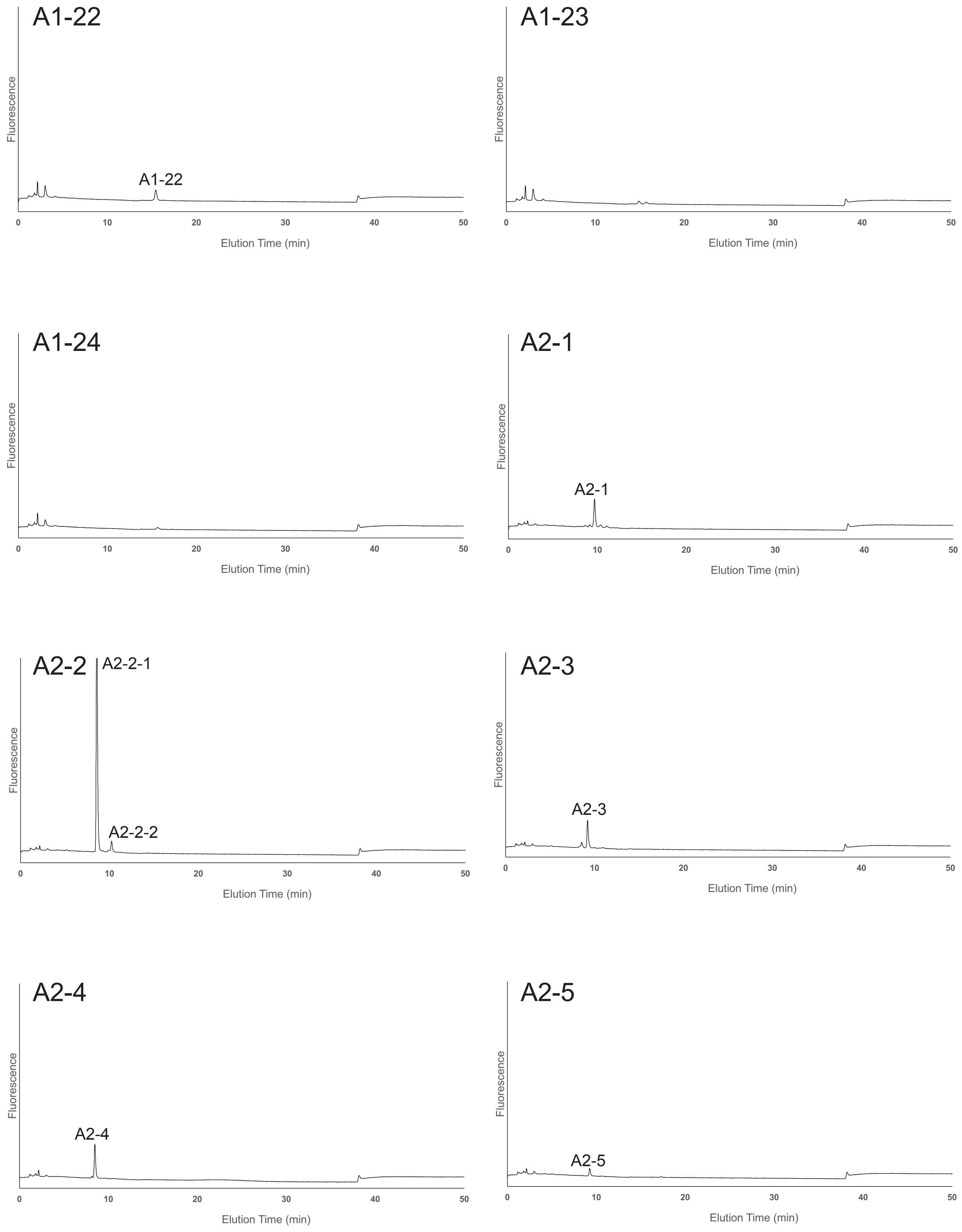

Supplementary Figure S1-8

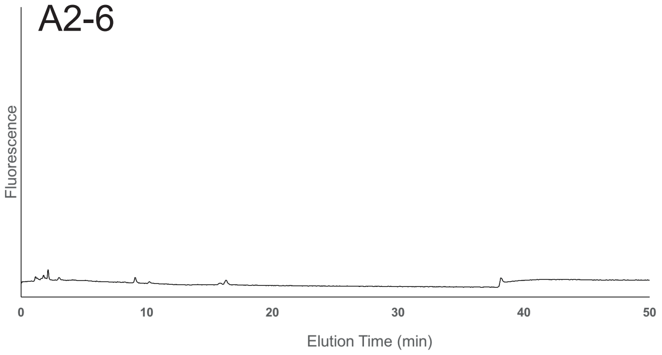

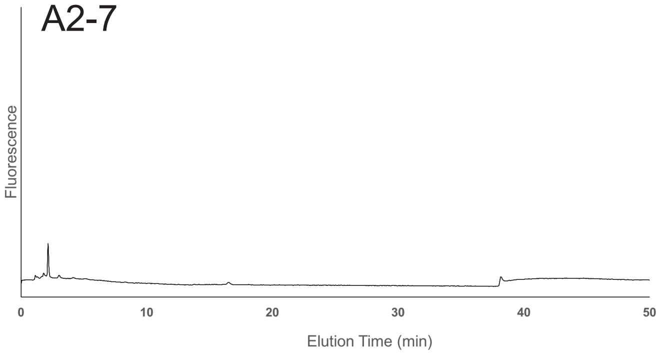

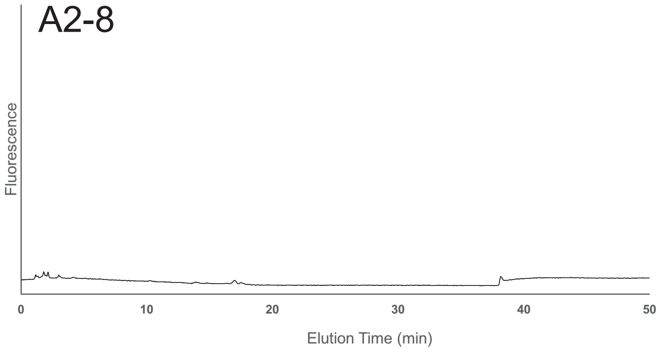

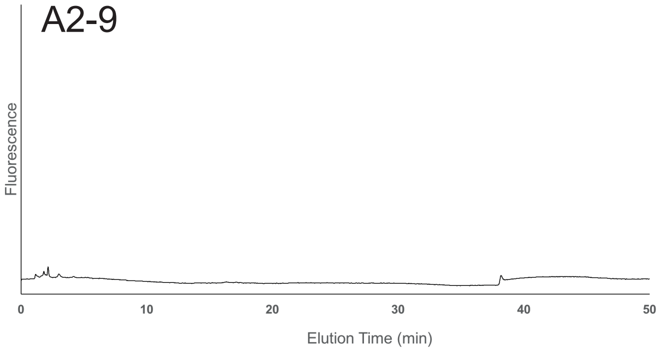

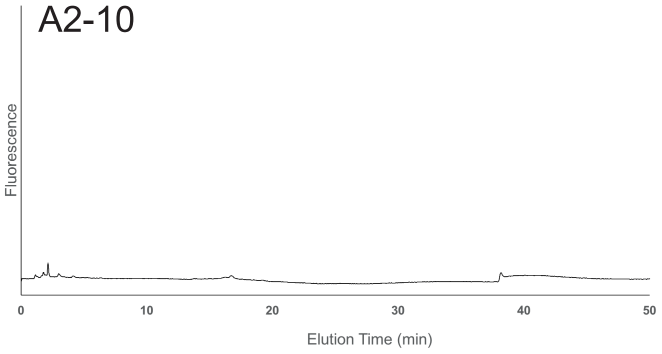

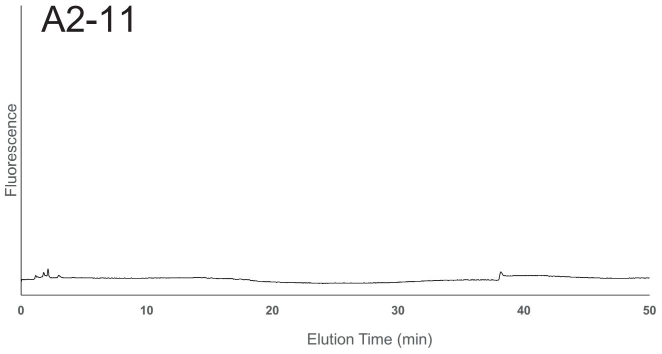

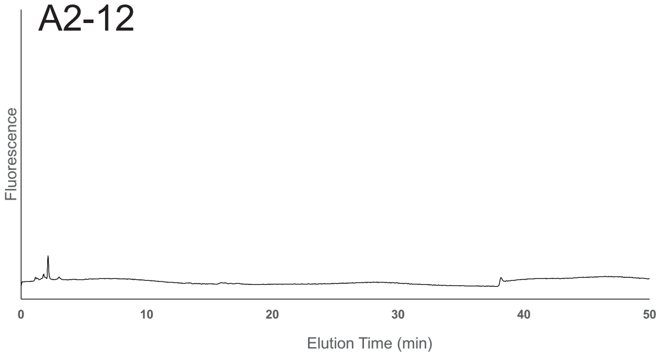

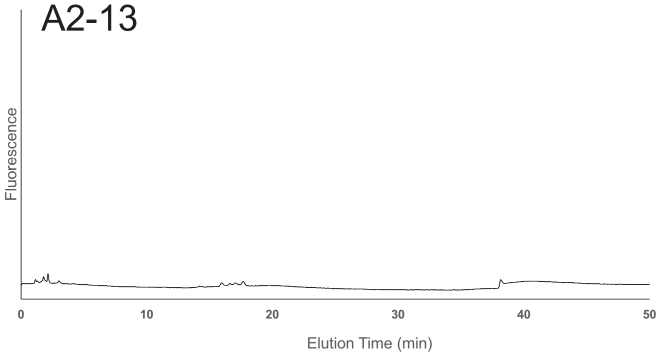

Supplementary Figure S1-9

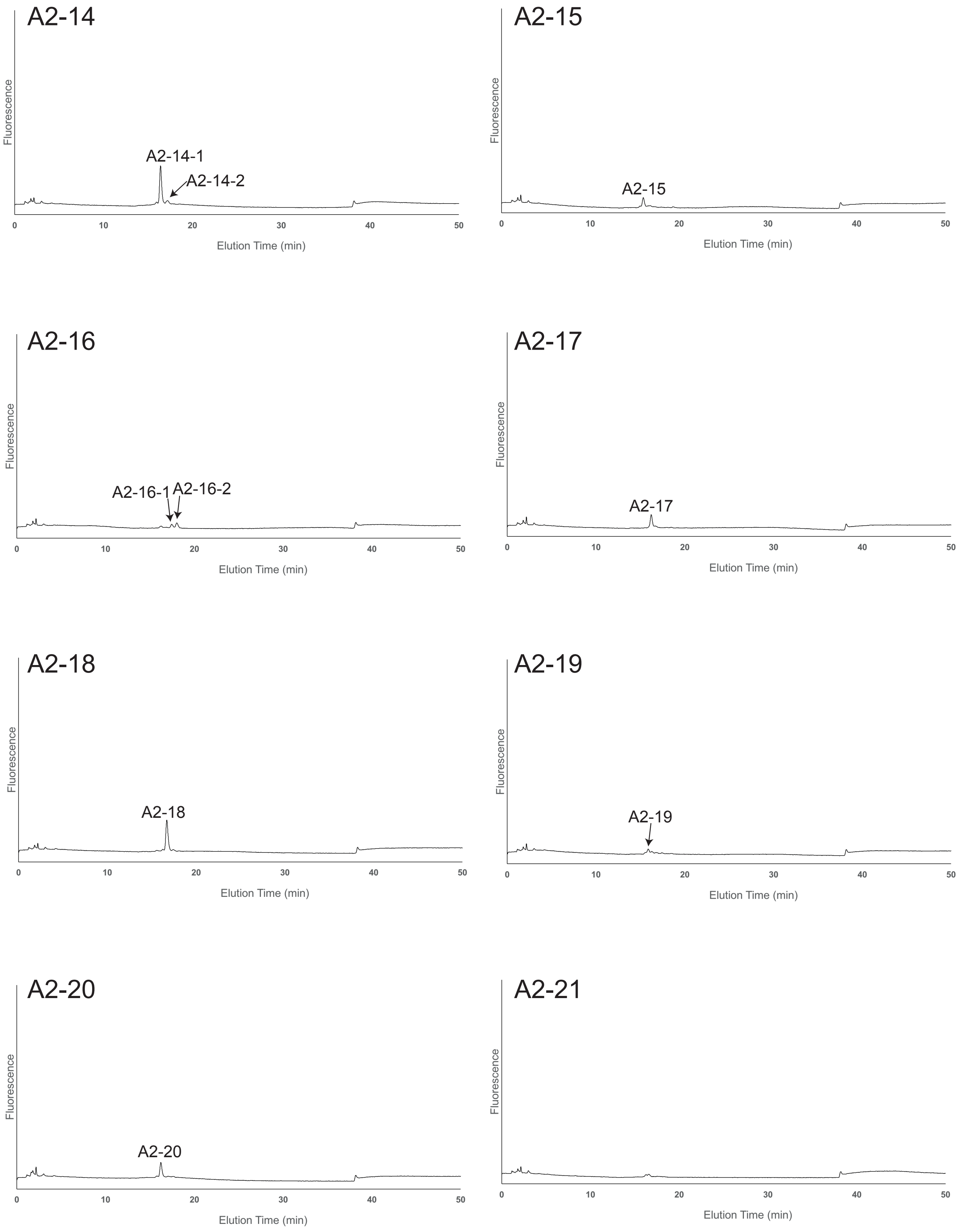

Supplementary Figure S1-10

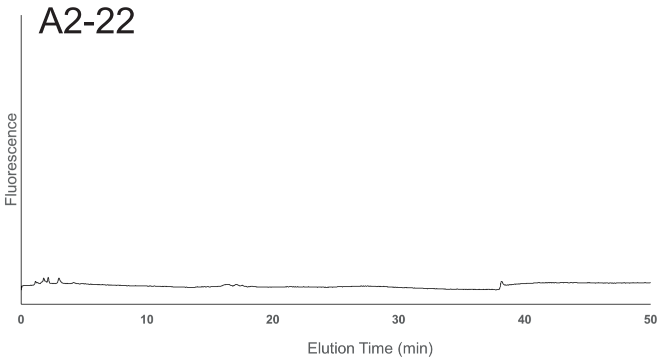

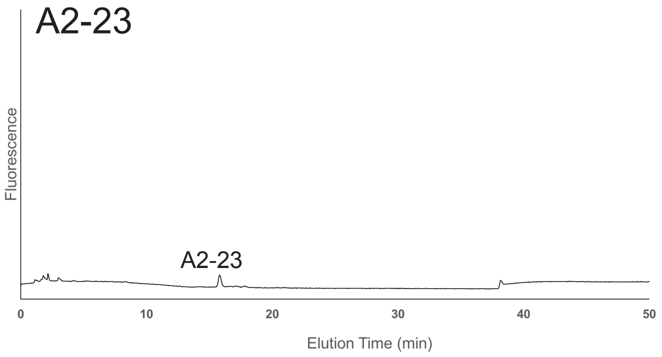

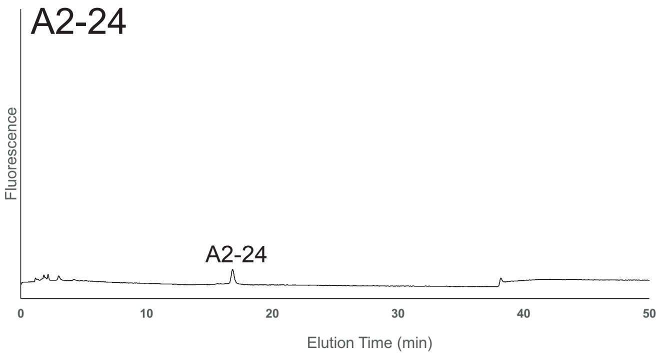

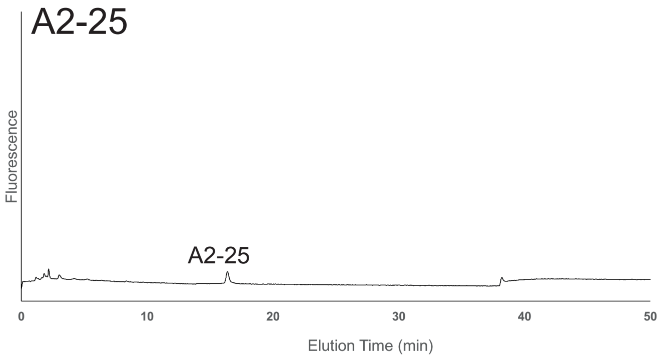

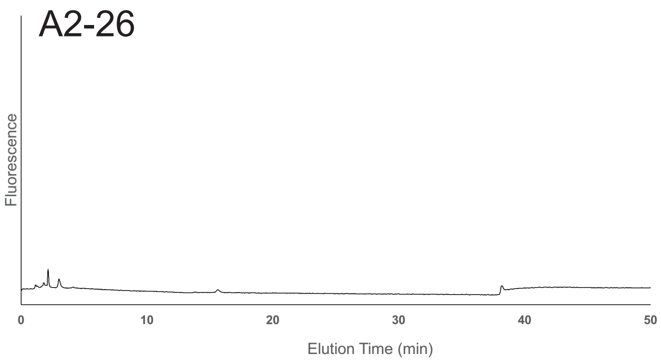

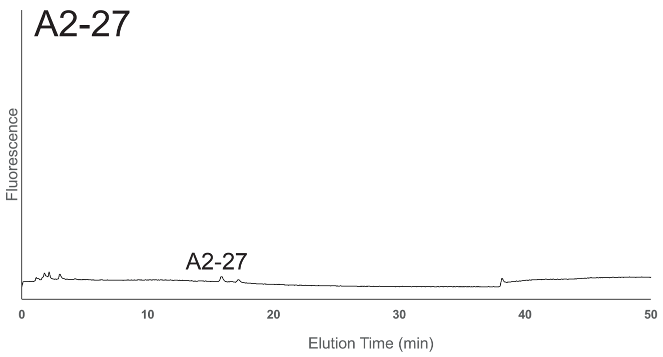

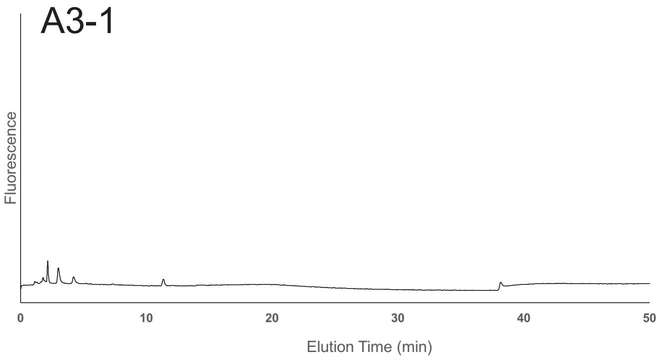

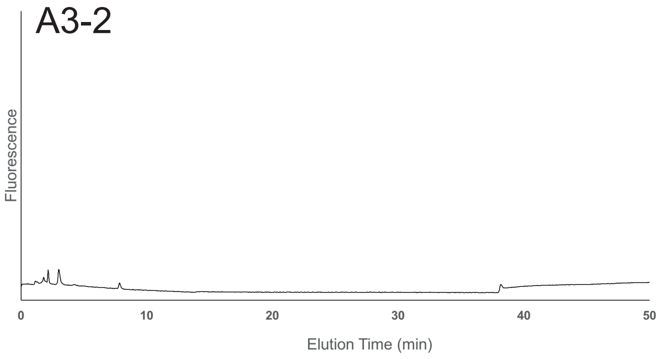

Supplementary Figure S1-11

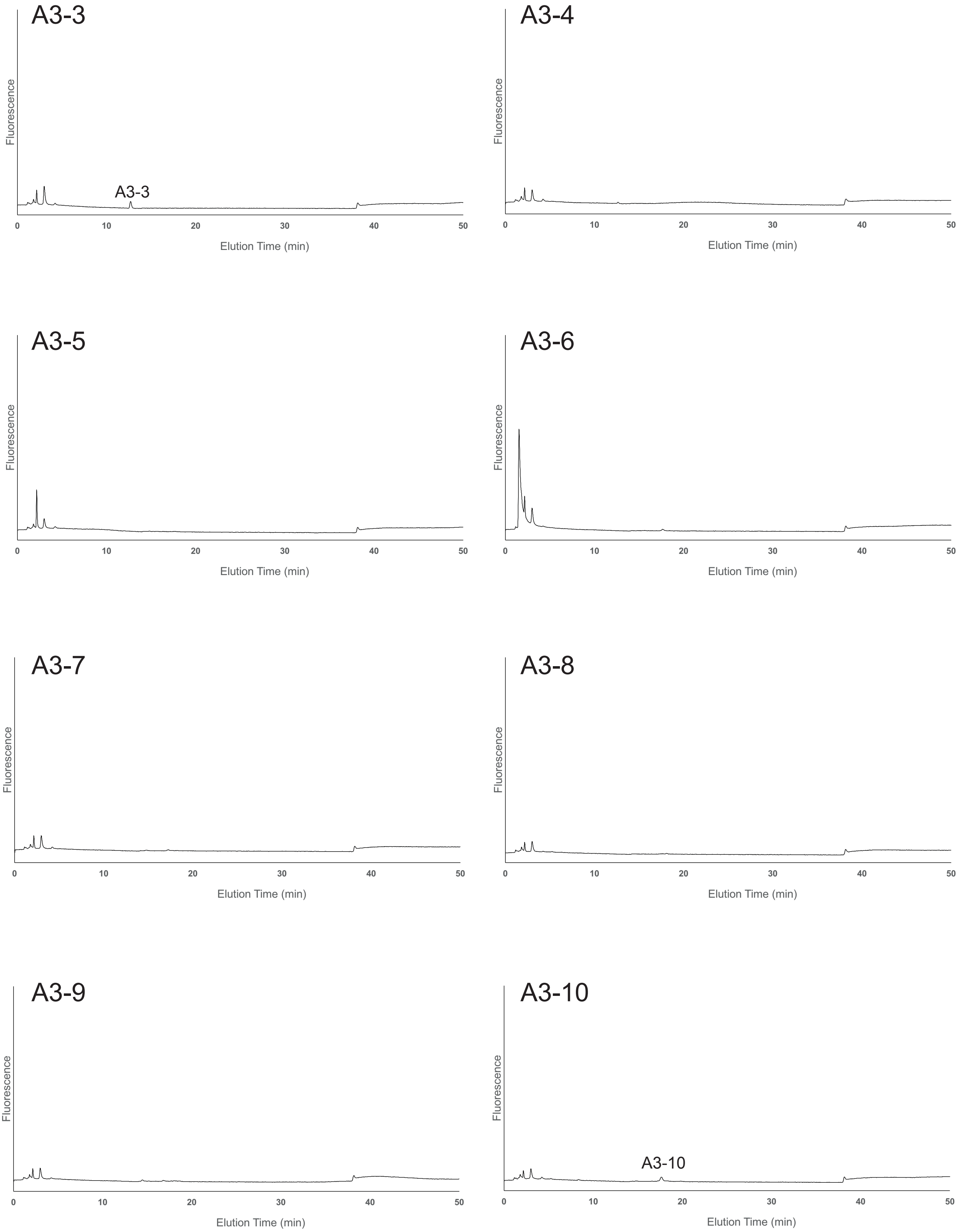

Supplementary Figure S1-12

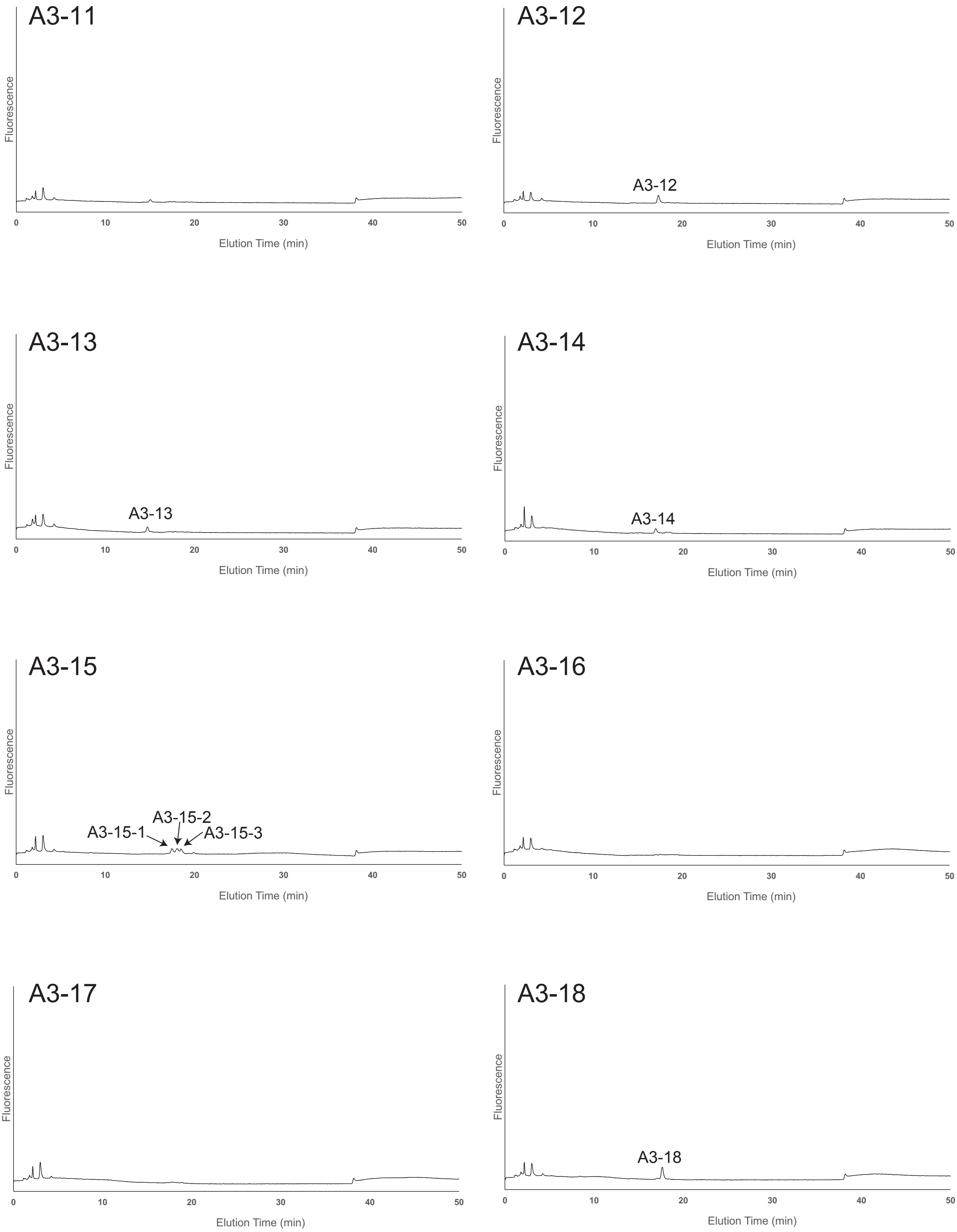

Supplementary Figure S1-13

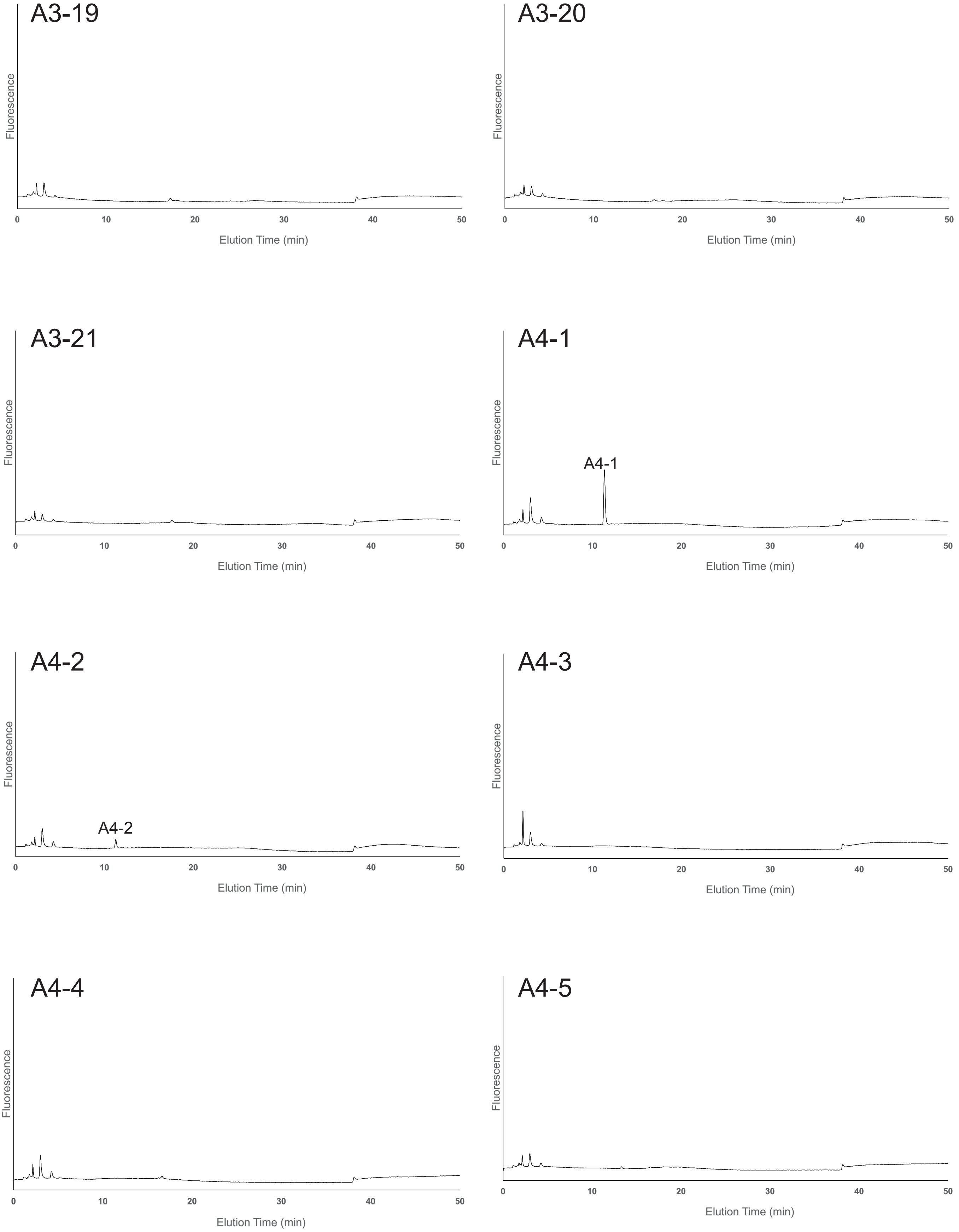

Supplementary Figure S1-14

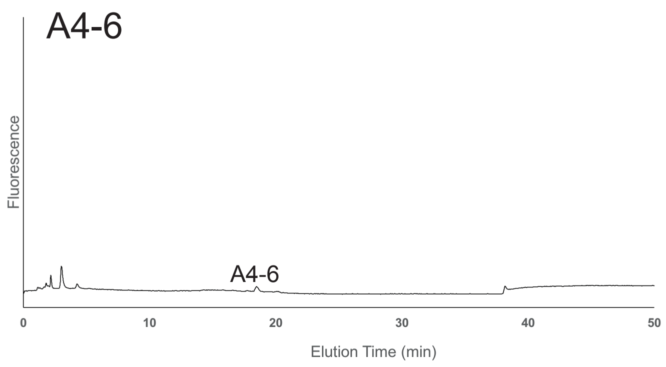

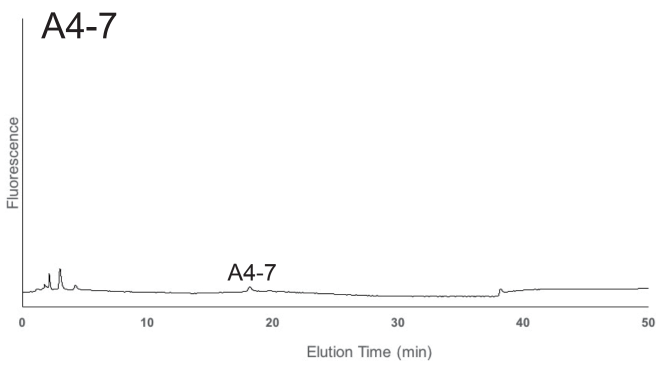

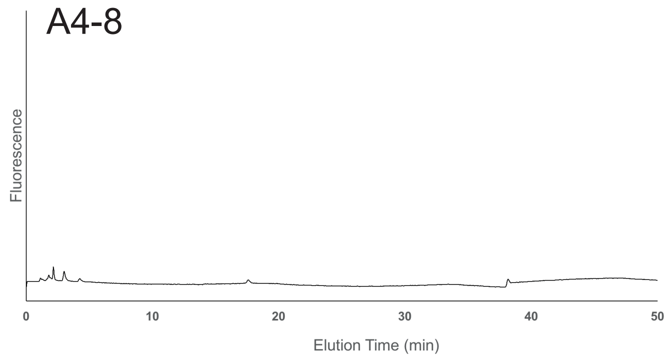

Supplementary Figure S2-1

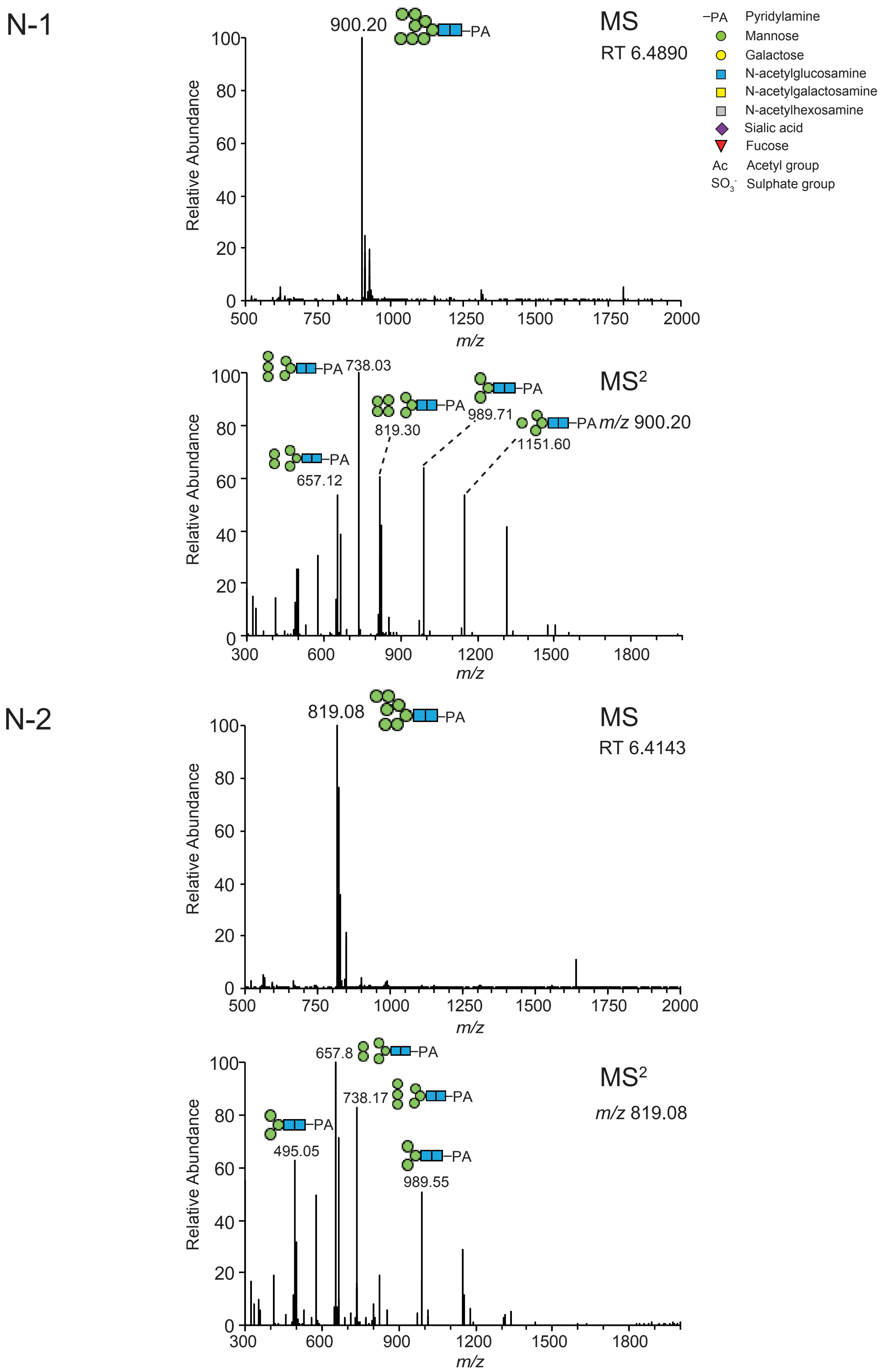

Supplementary Figure S2-2

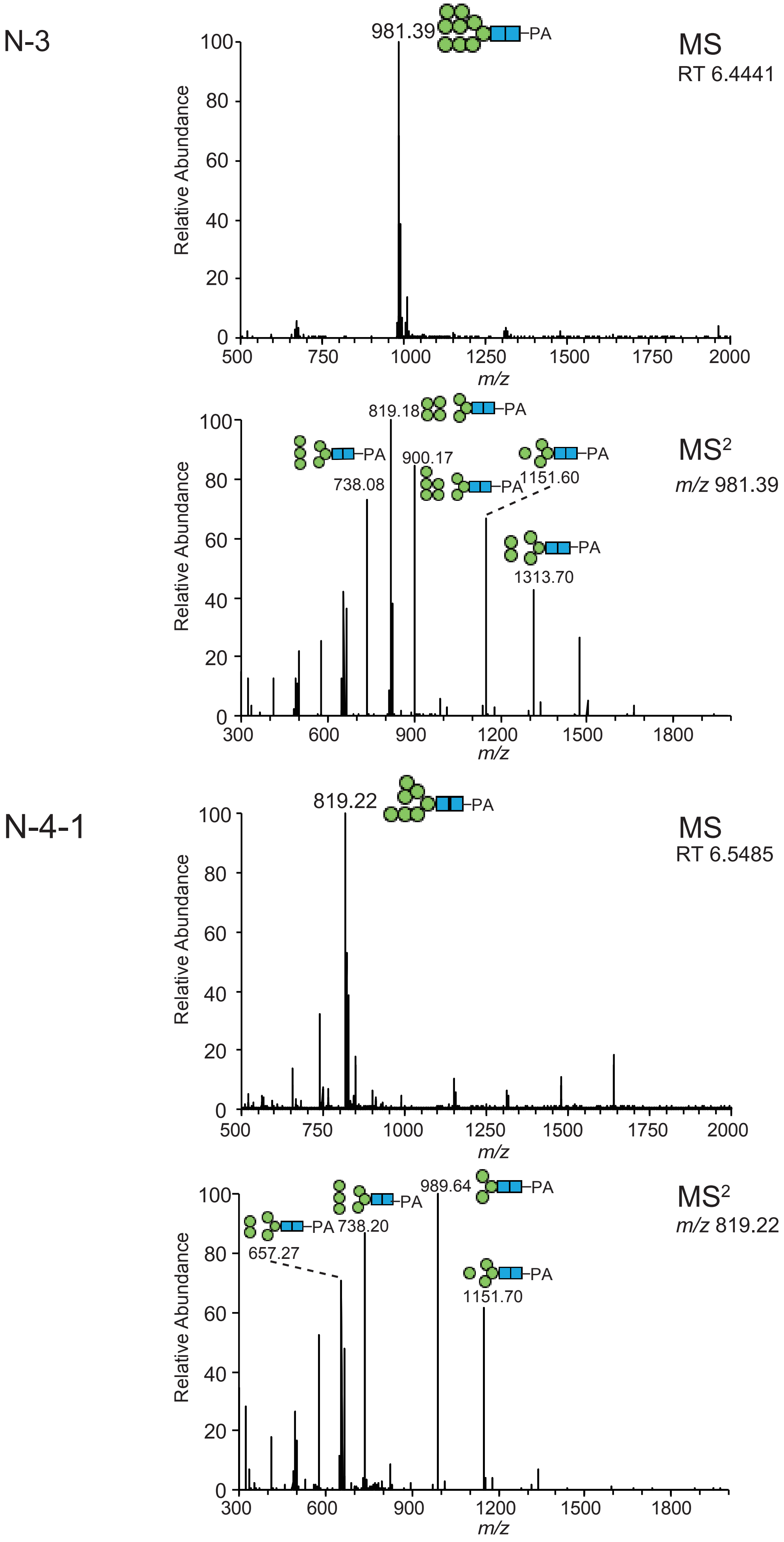

Supplementalry Figure S2-3

N-4-2

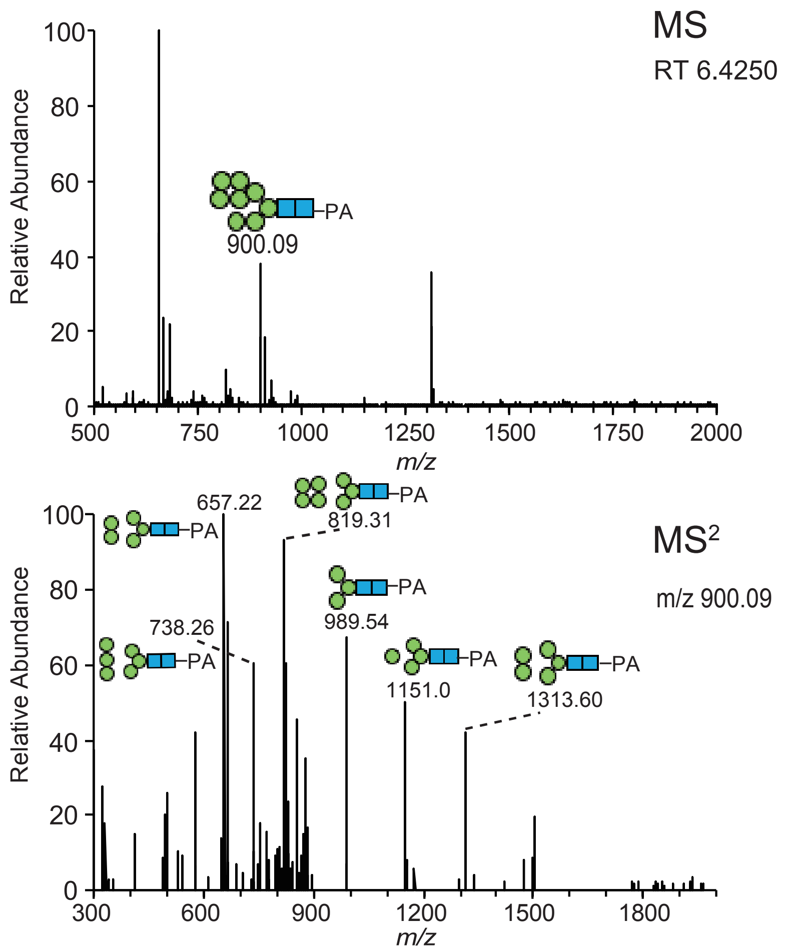

N-5

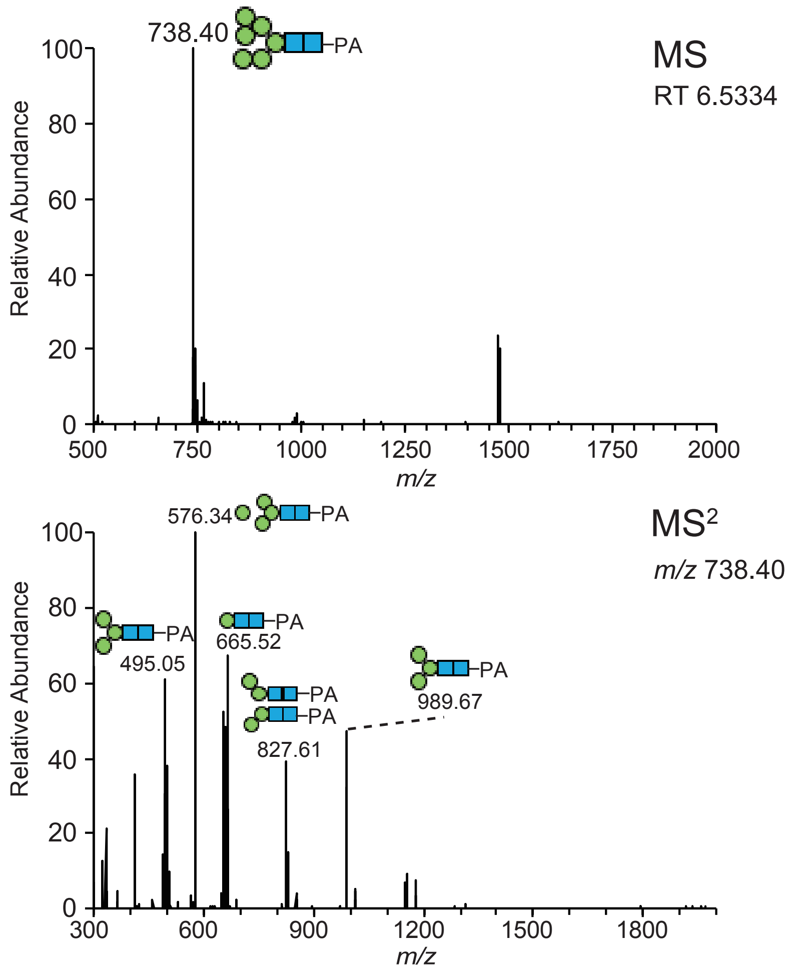

Supplementary Figure S2-4

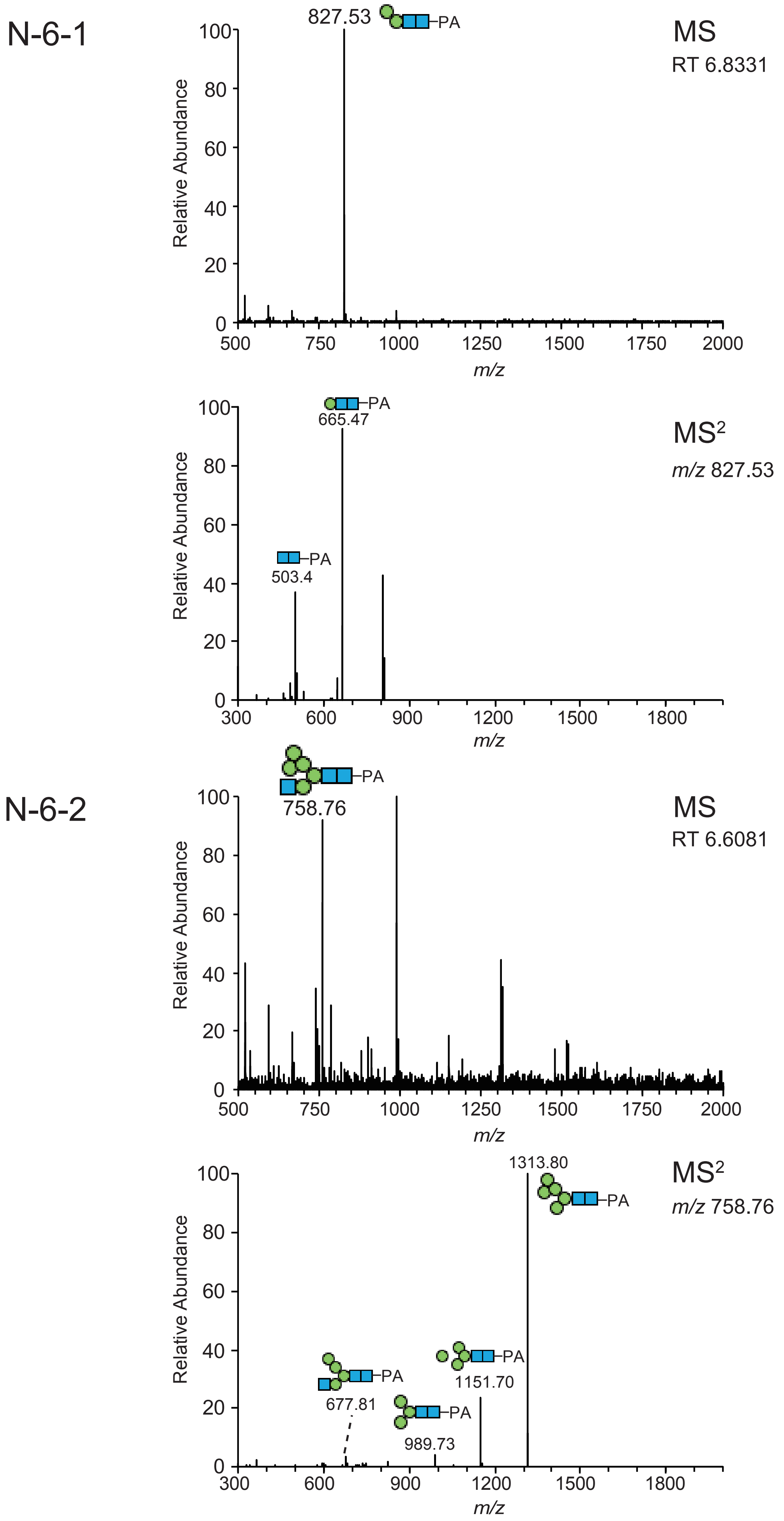

Supplementary Figure S2-5

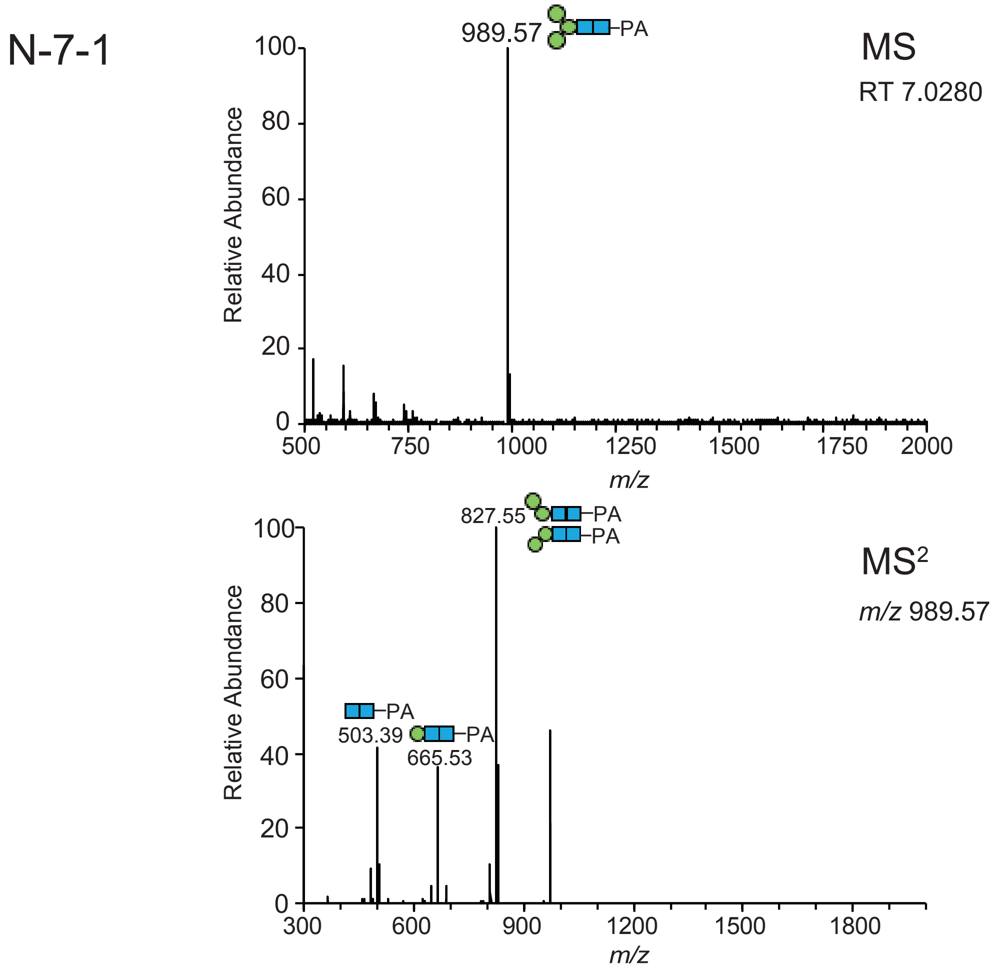

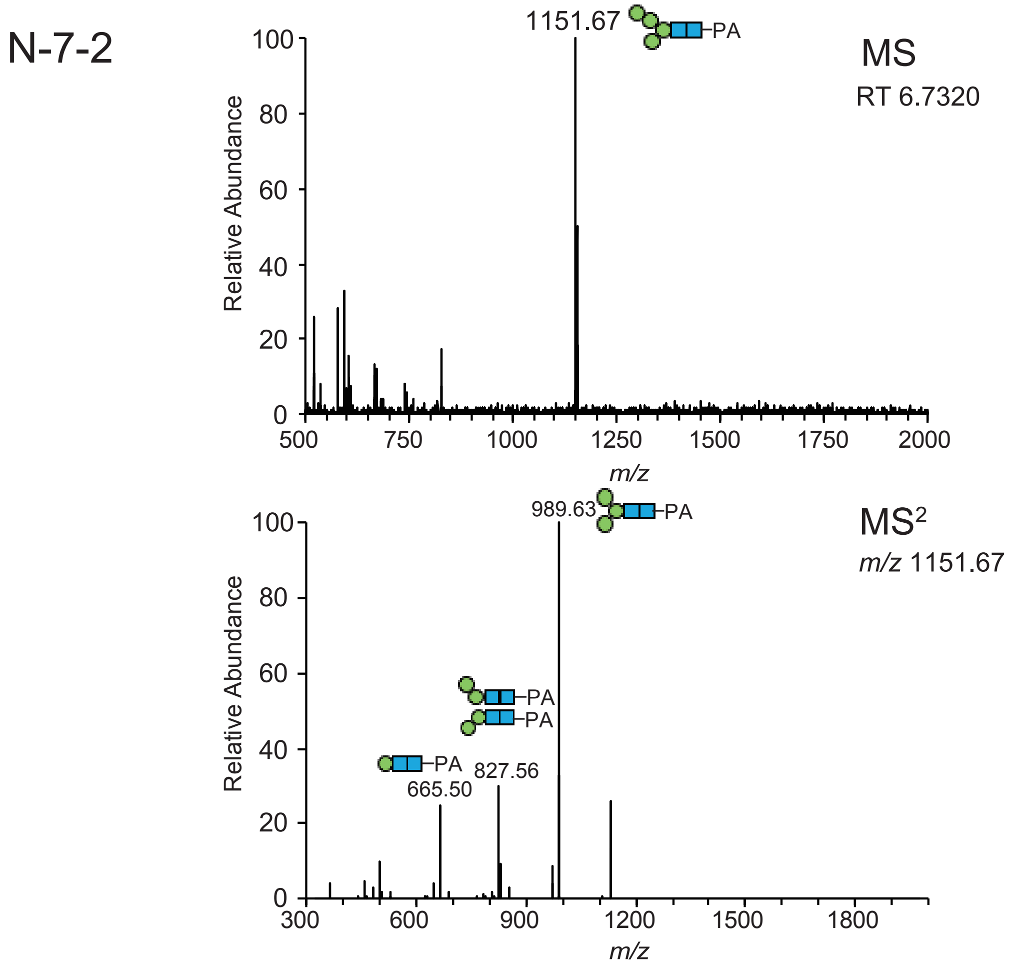

Supplementary Figure S2-6

N-8-1

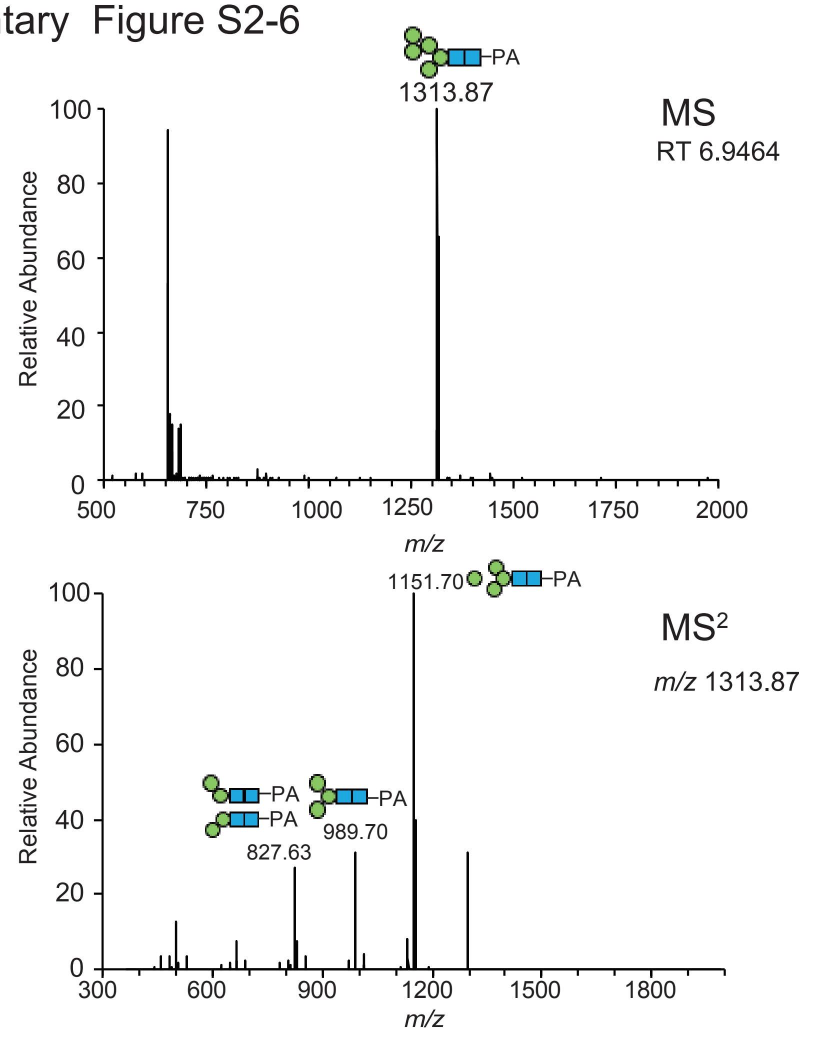

N-8-2

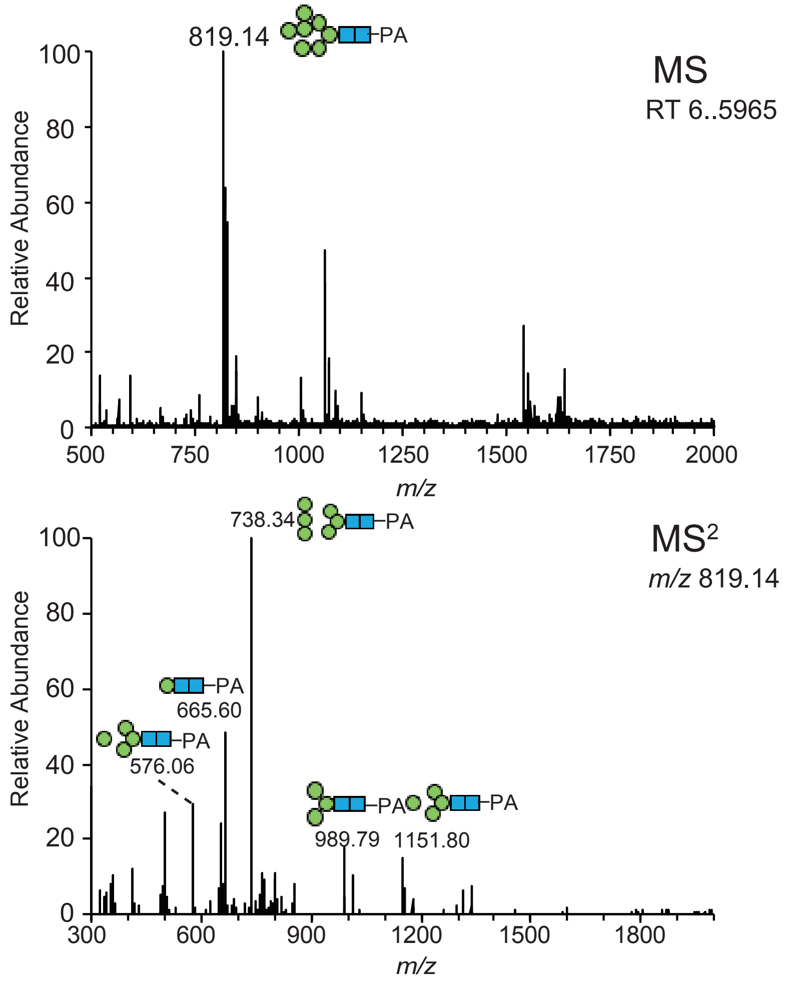

Supplementary Figure S2-7

N-8-3

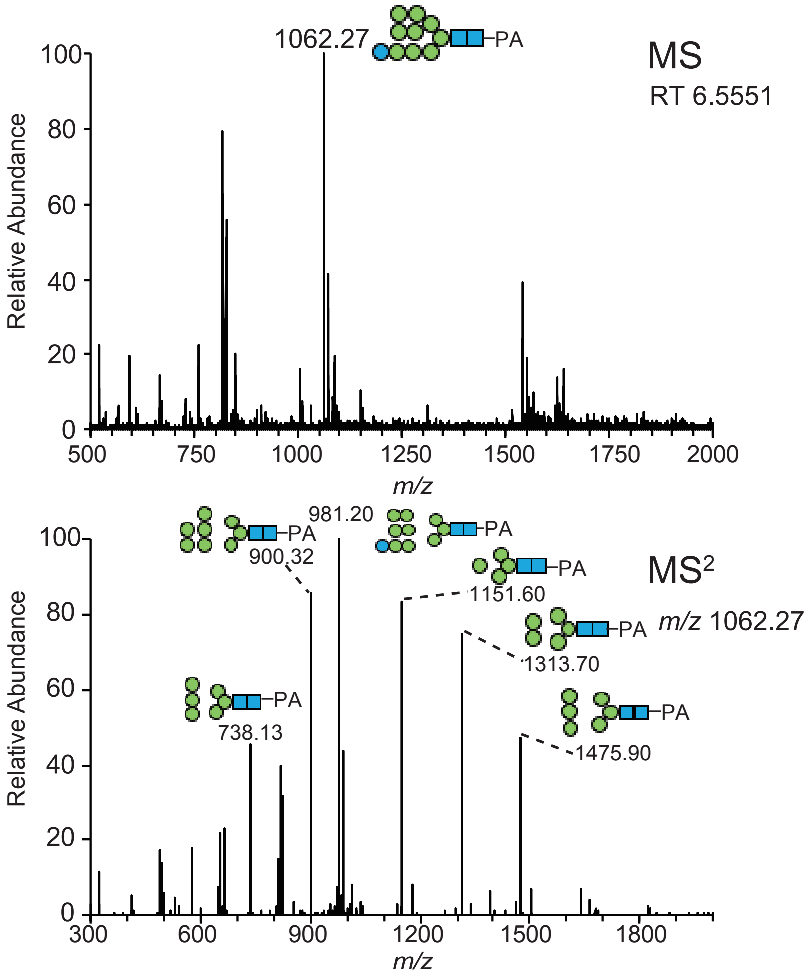

N-10

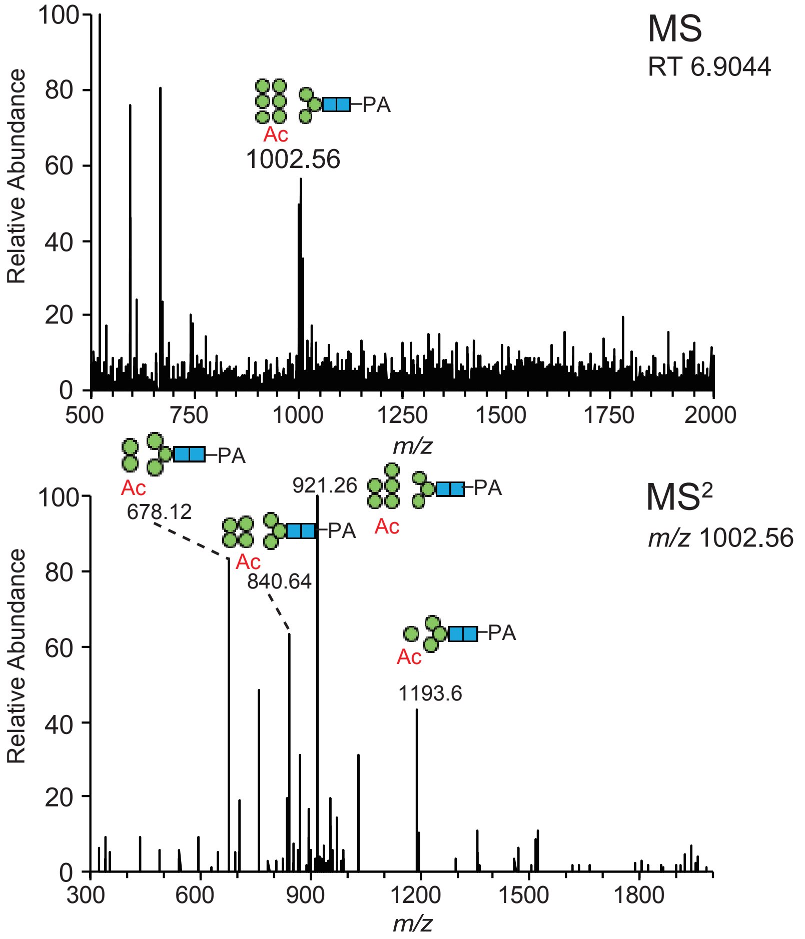

Supplementary Figure S2-8

N-11-1

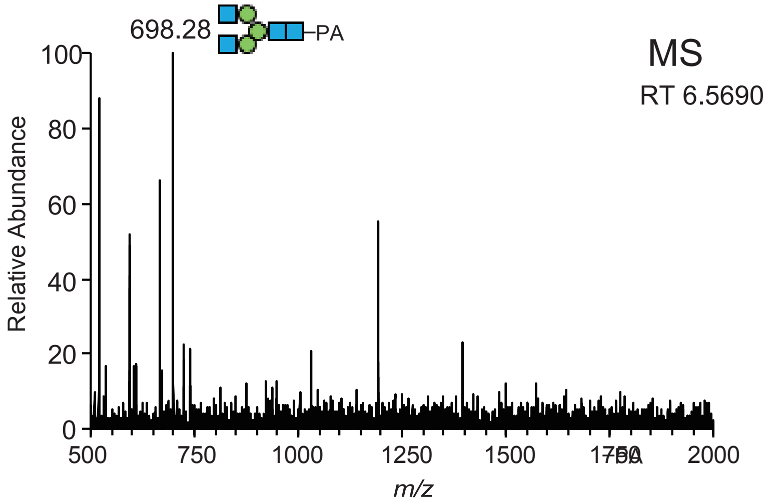

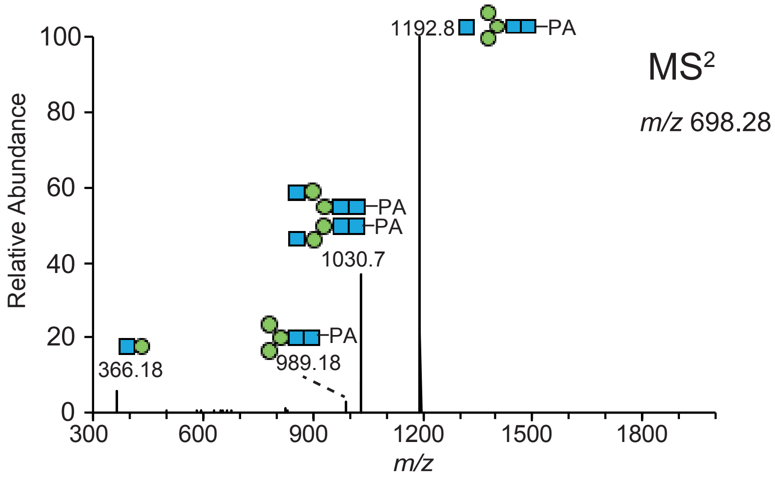

N-11-2

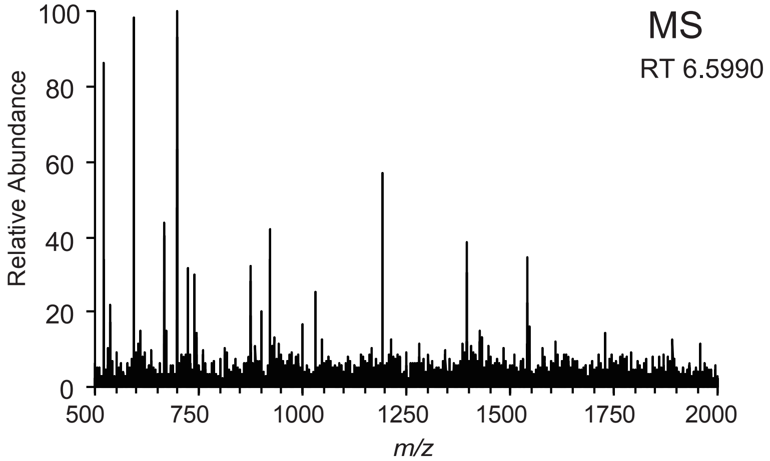

Supplementary Figure S2-9

N-12-1

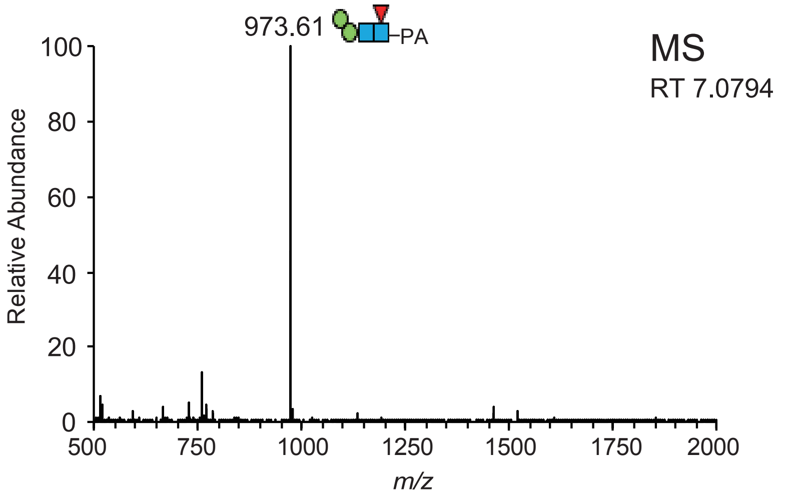

N-12-2

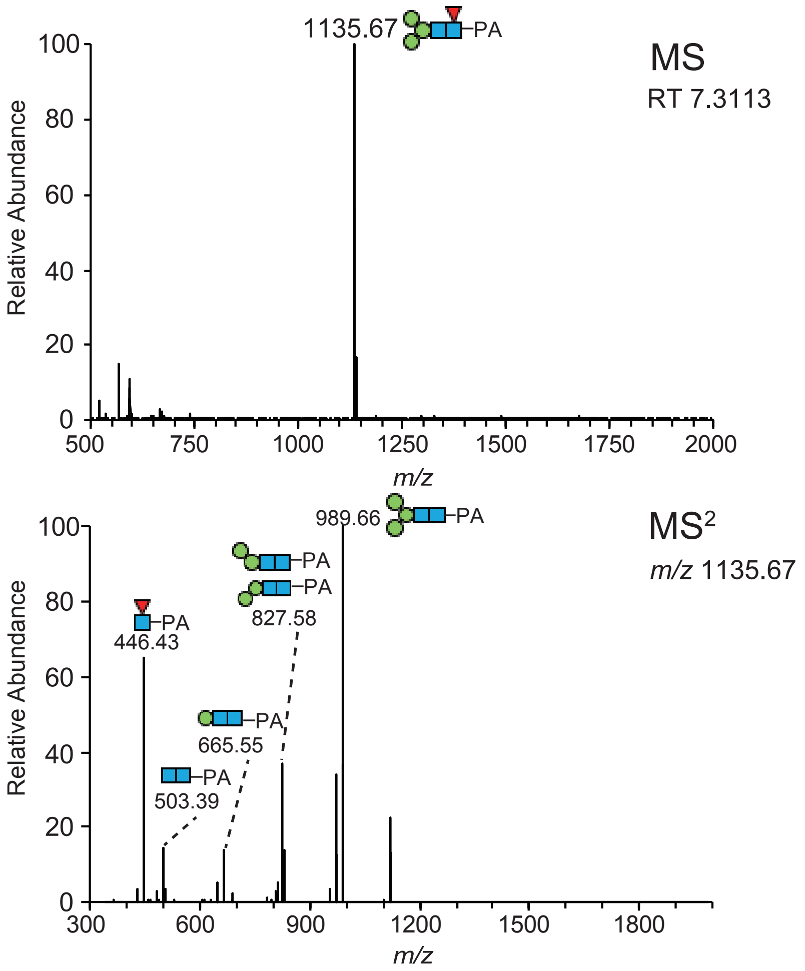

Supplementary Figure S2-10

N-12-3

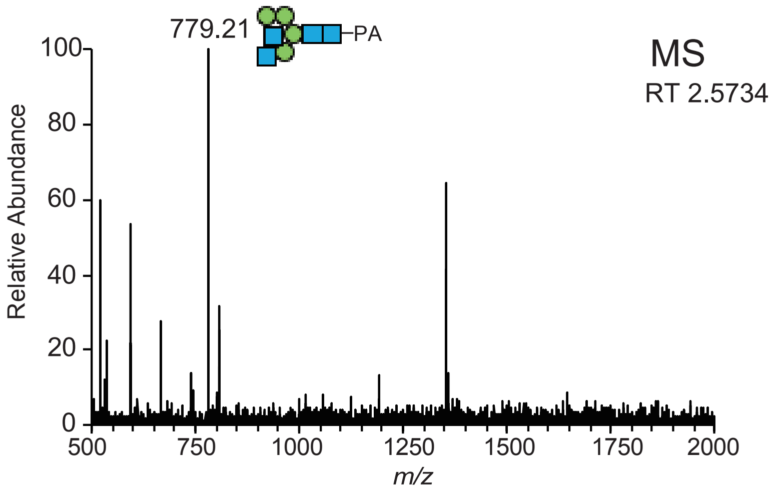

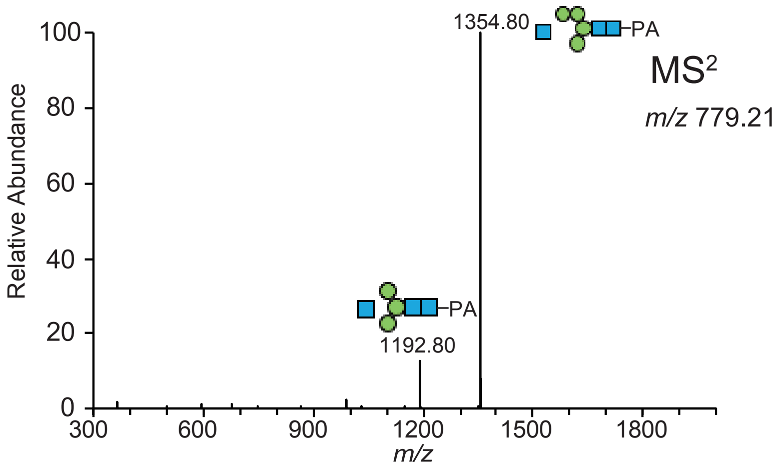

N-14-1

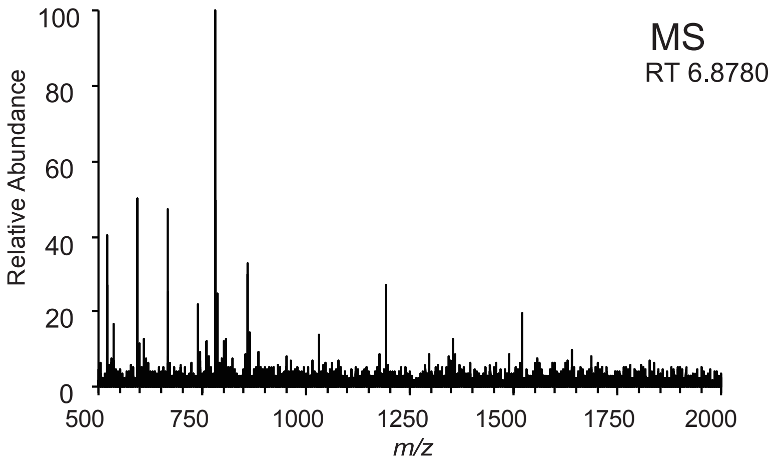

Supplementary Figure S2-11

N-14-2

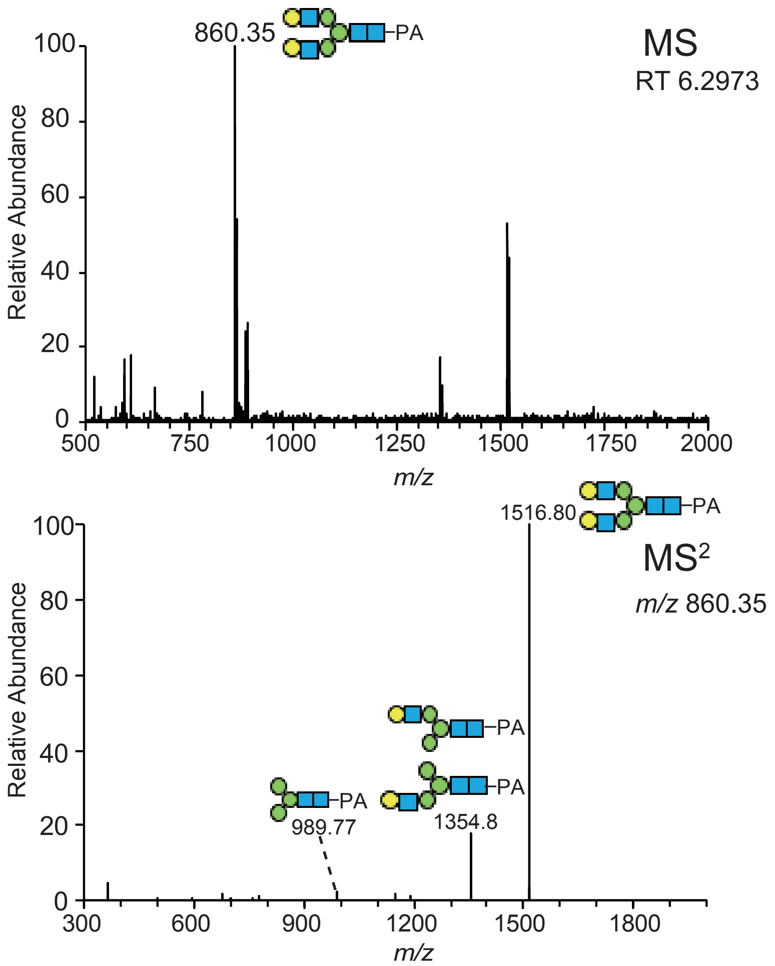

N-15

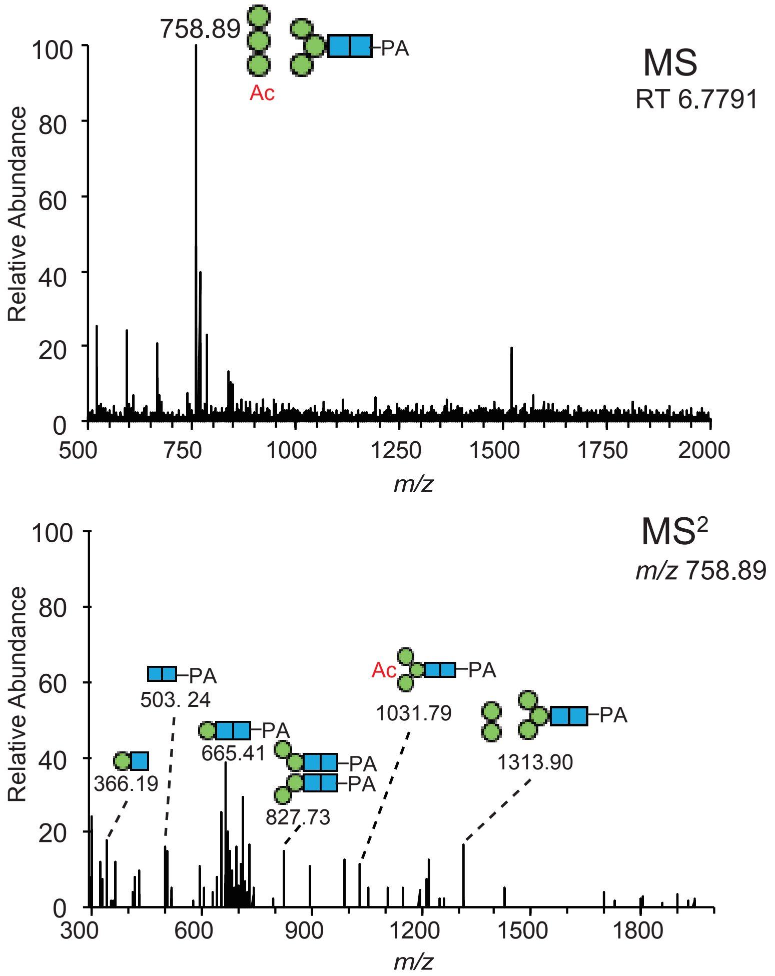

Supplementary Figure S2-12

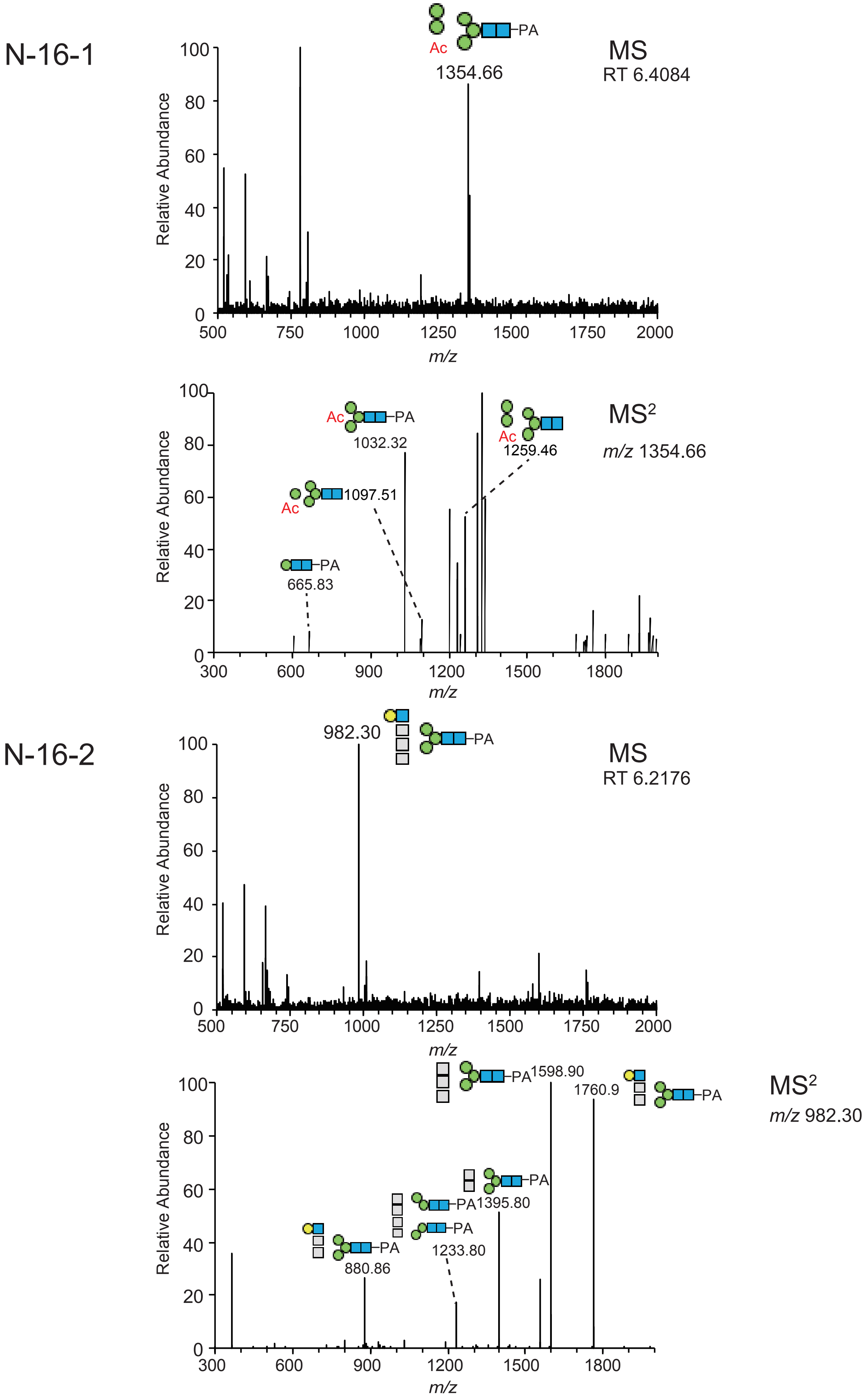

Supplementary Figure S2-13

N-17

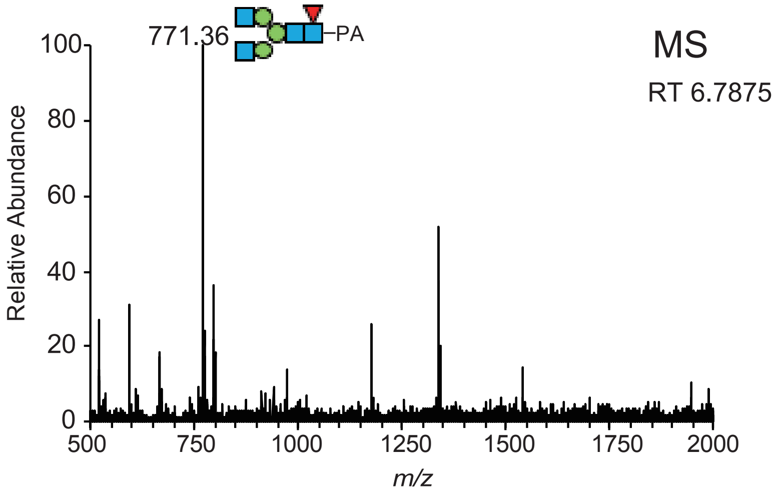

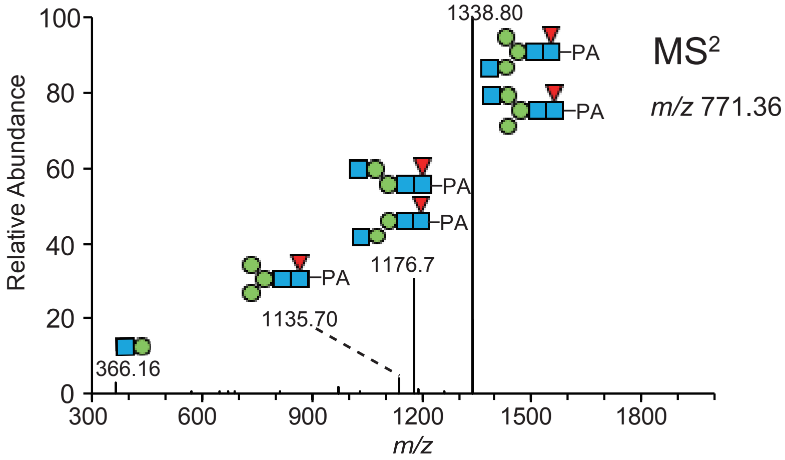

N-20

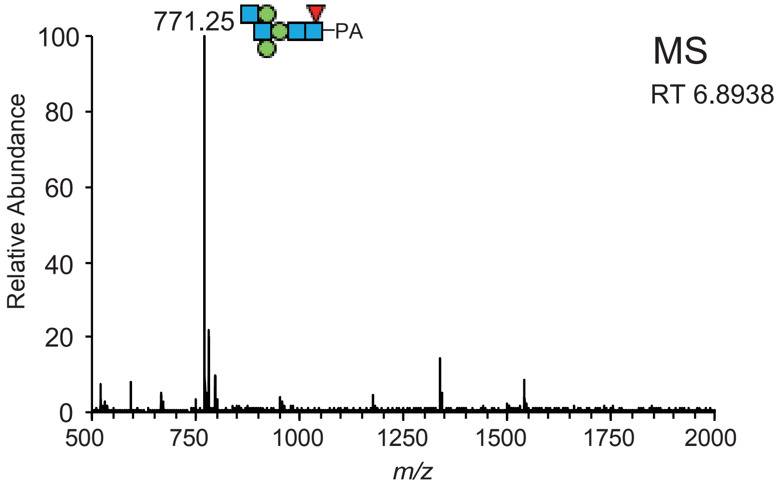

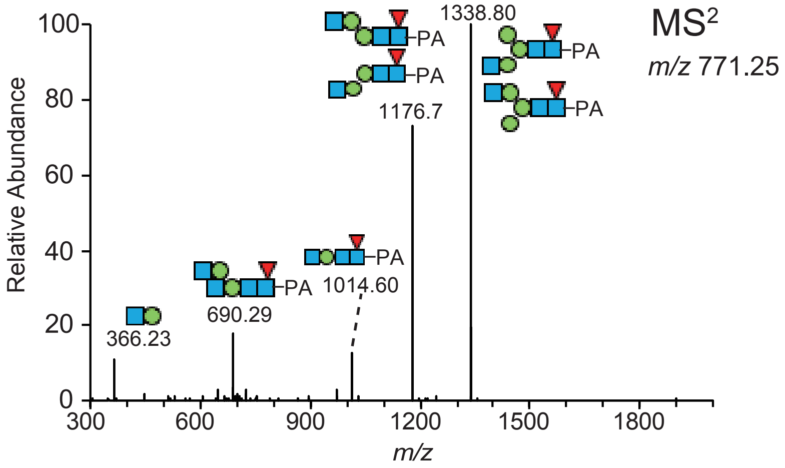

Supplementary Figure S2-14

N-21-1

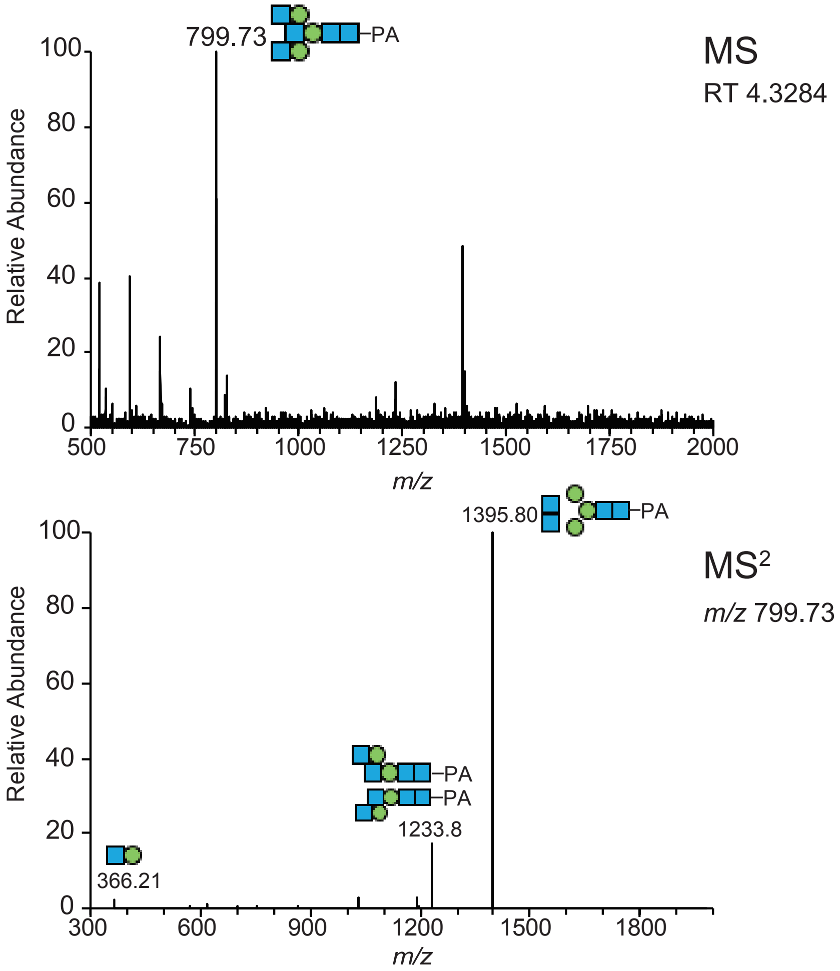

N-21-2

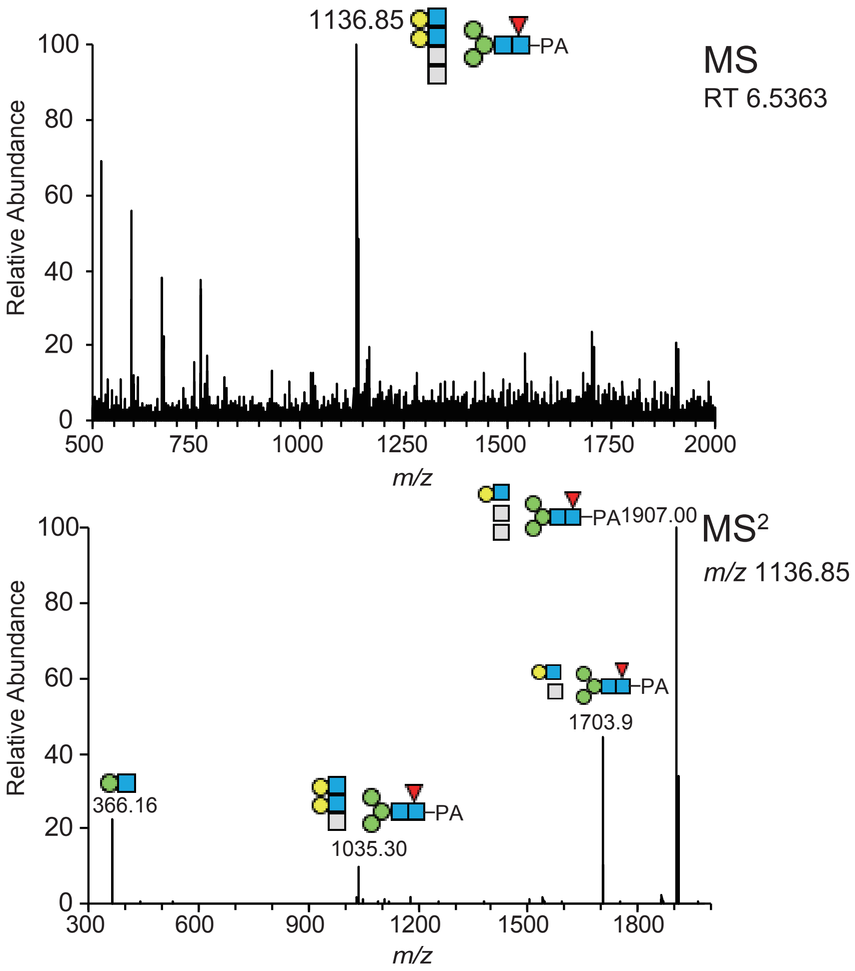

Supplementalry Figure S2-15

N-22-1

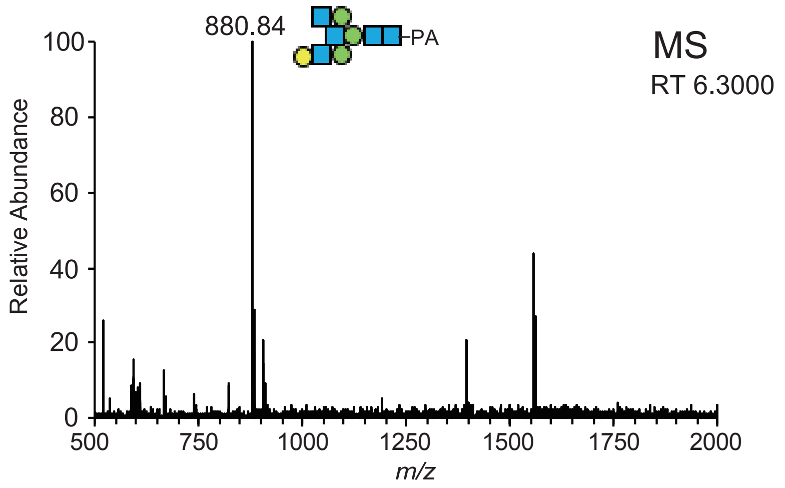

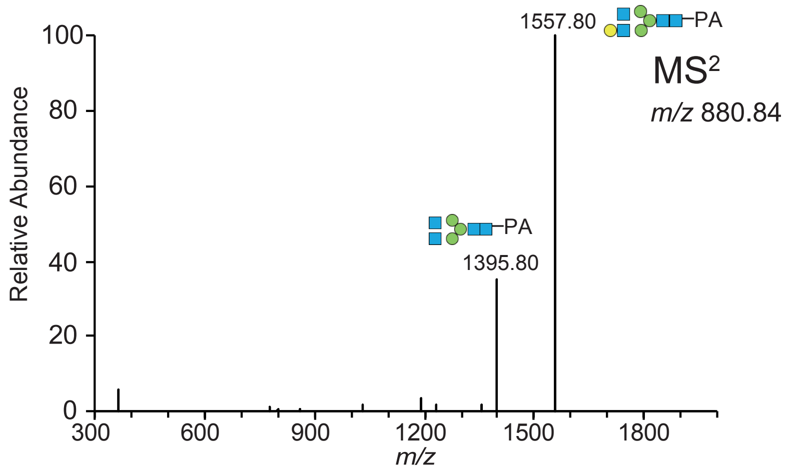

N-22-2

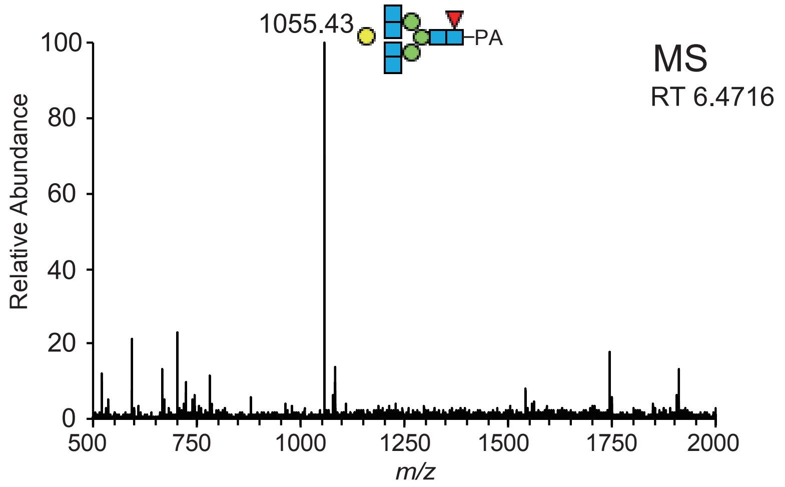

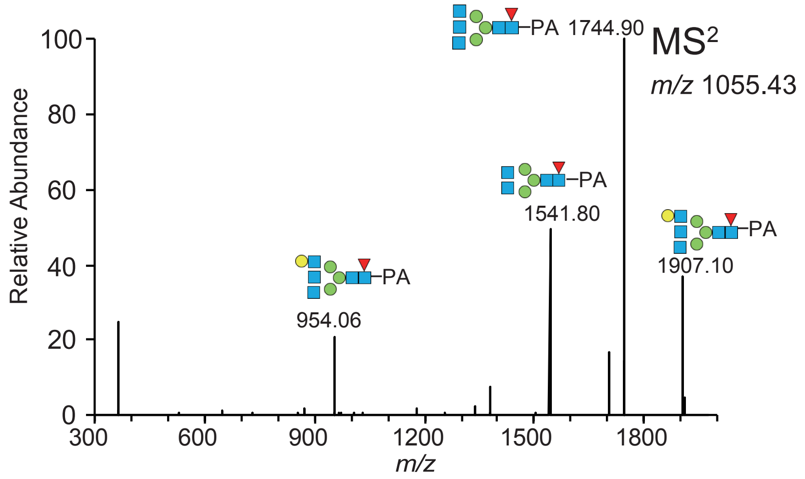

Supplementary Figure S2-16

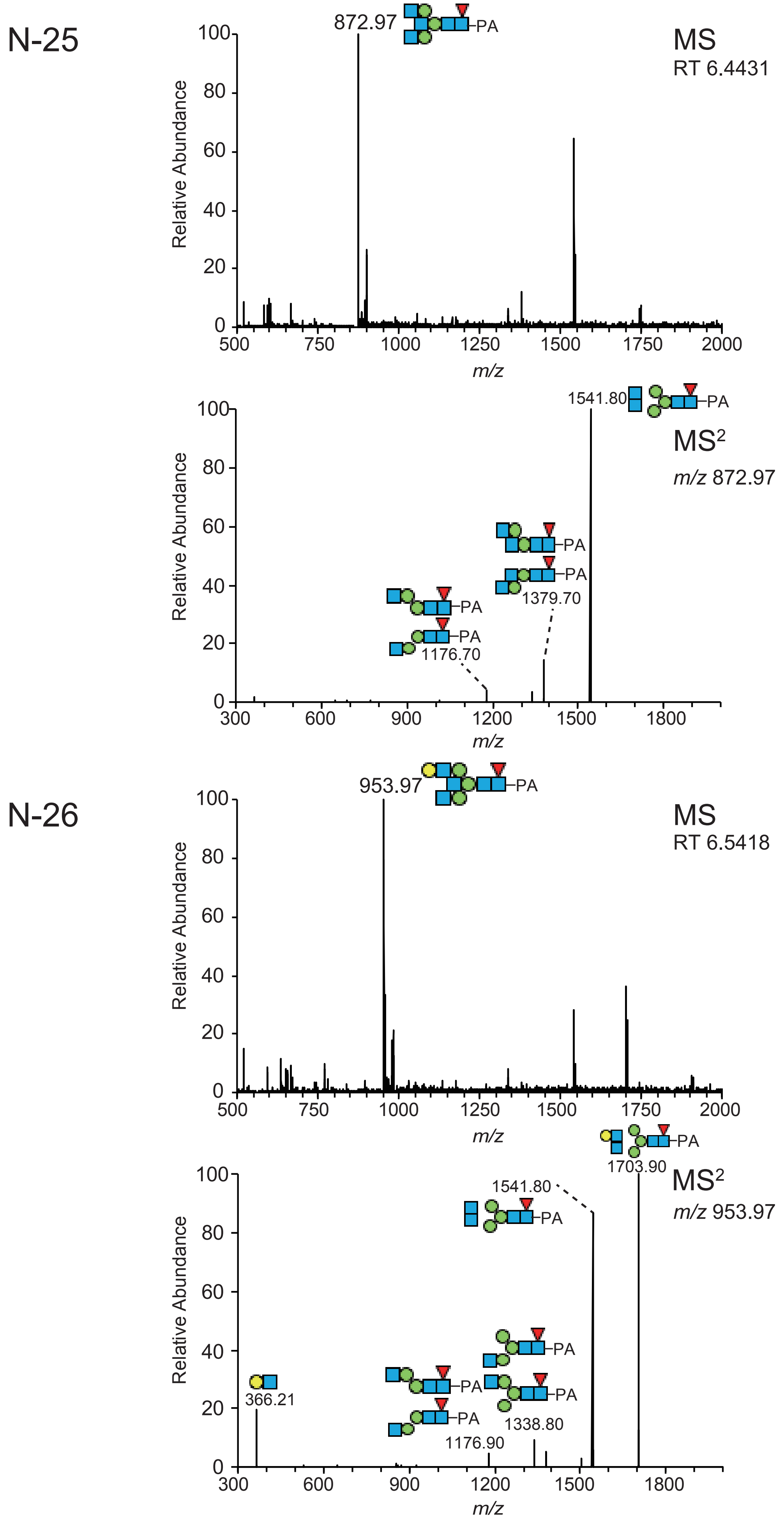

Supplementary Figure S2-17

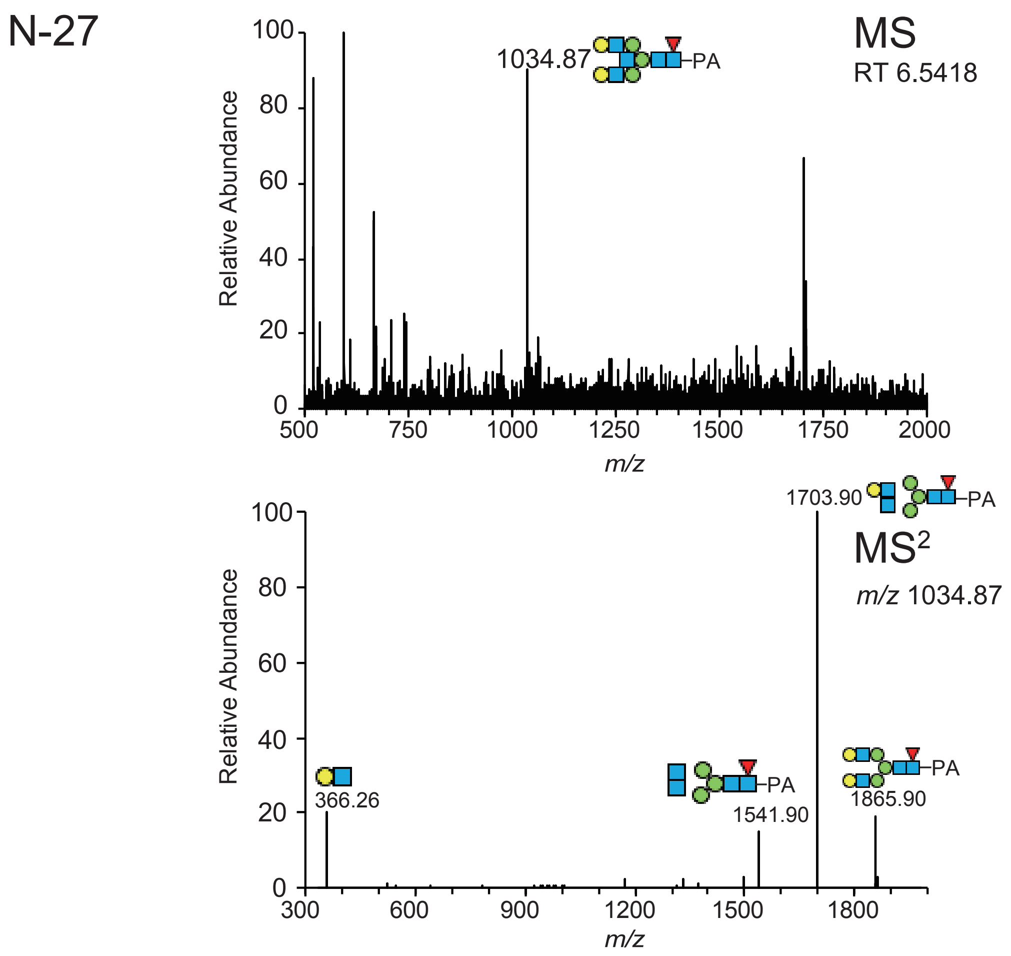

Supplementary Figure S2-18

A1-2

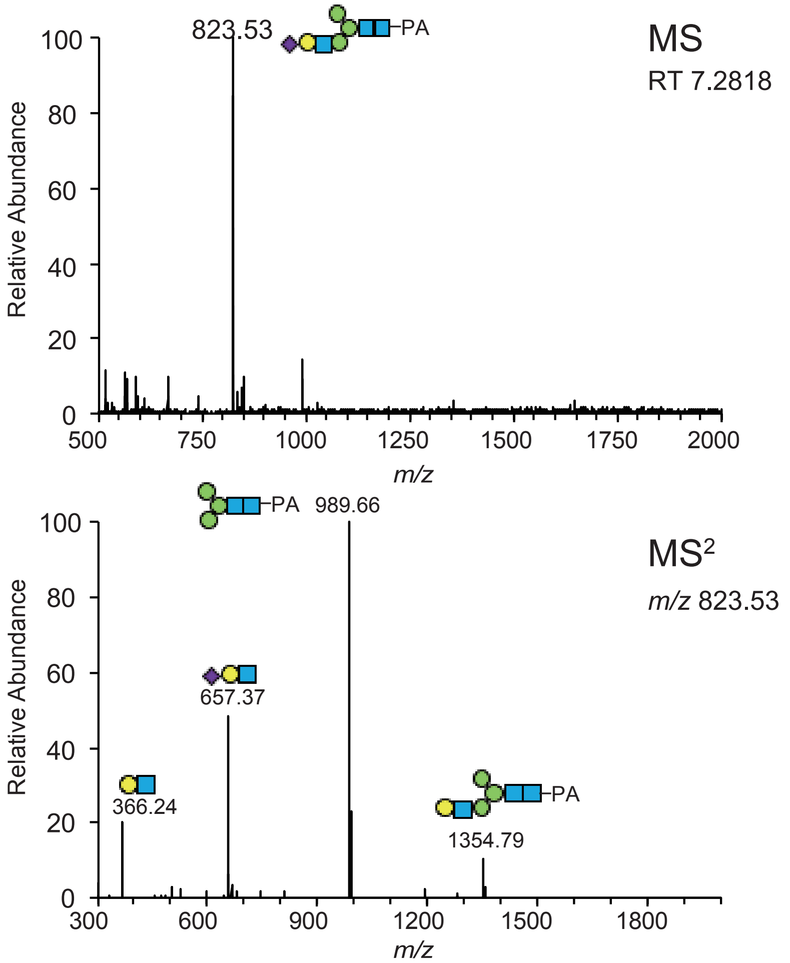

A1-3-1

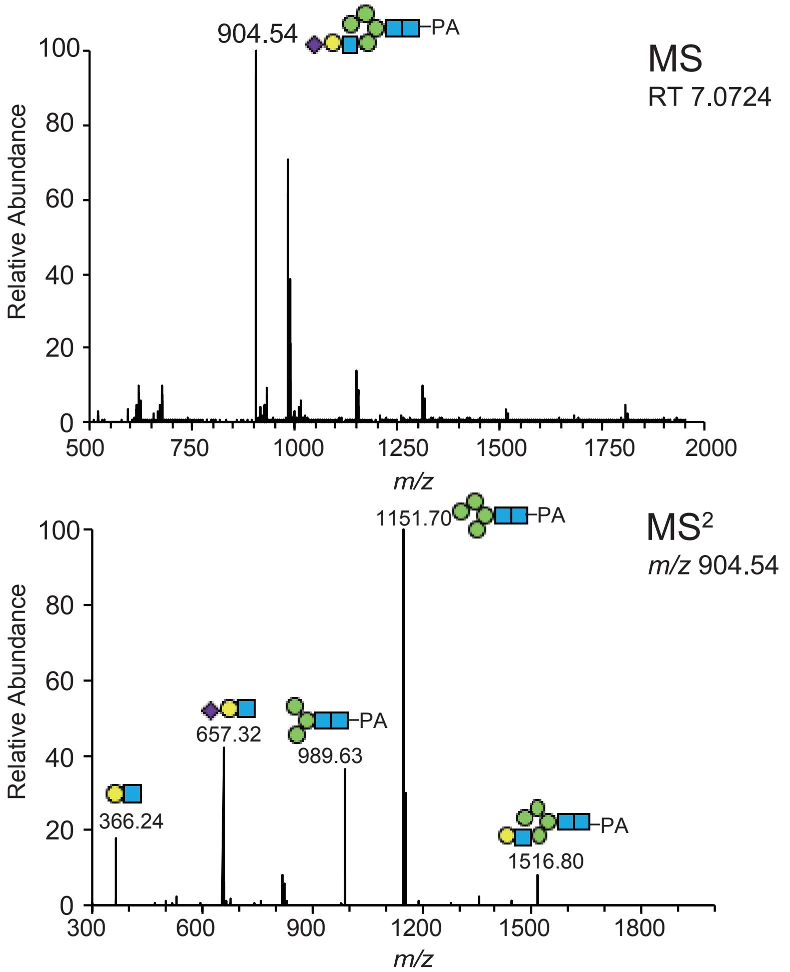

Supplementary Figure S2-19

A1-3-2

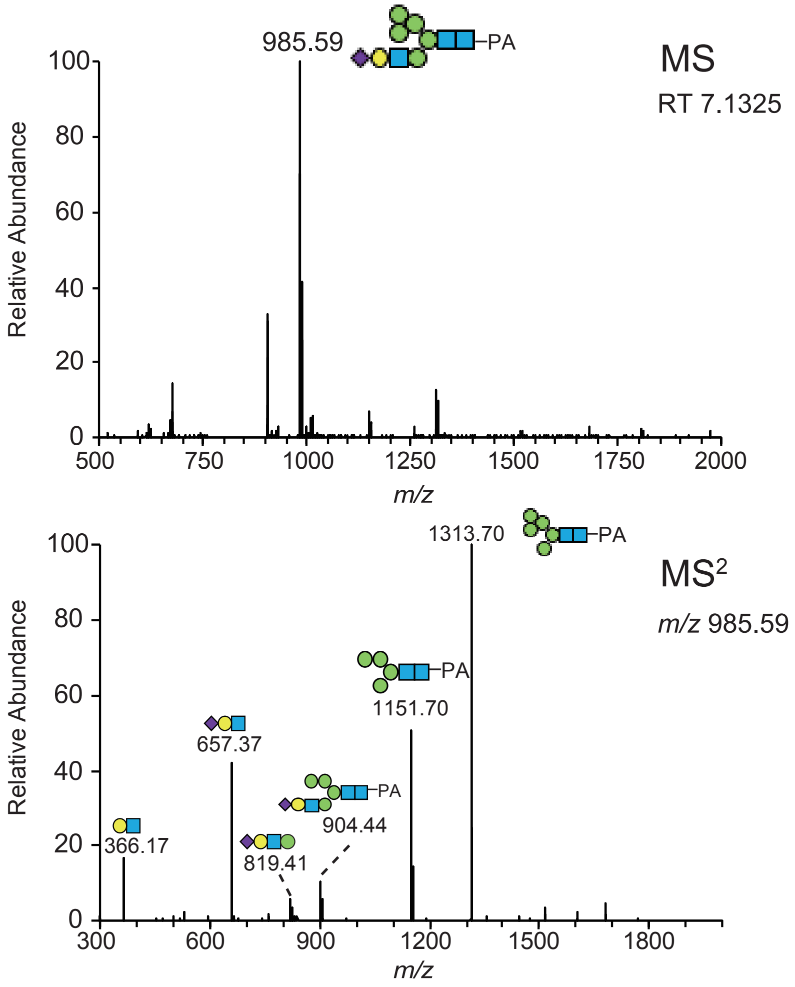

A1-4-1

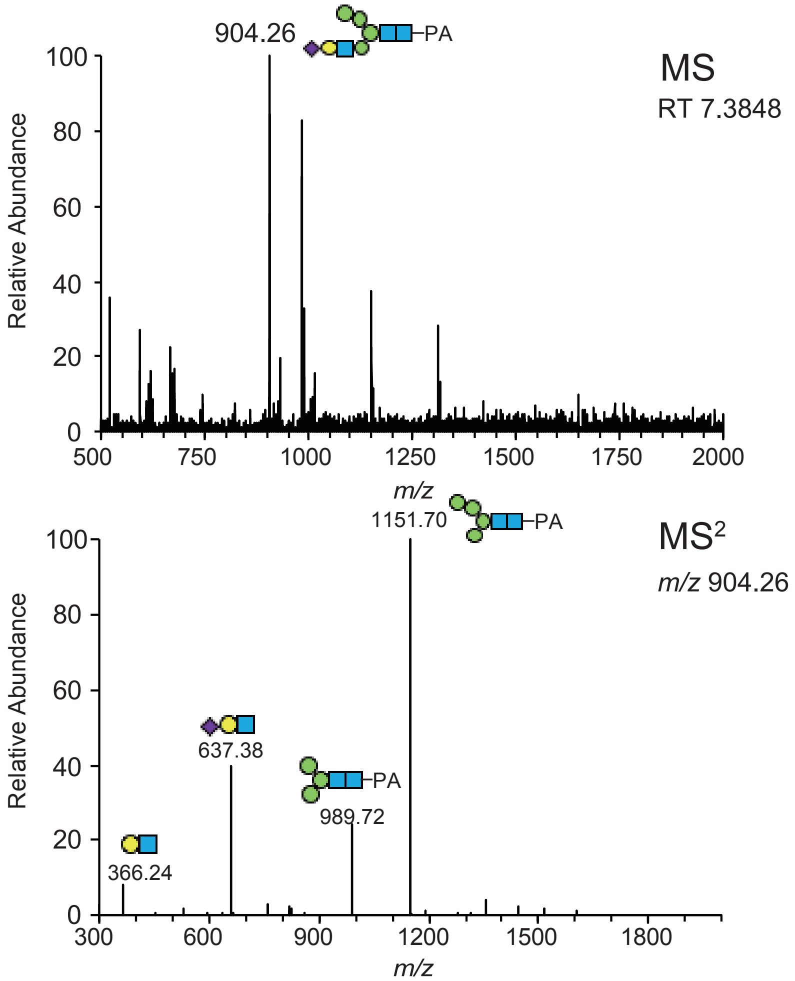

Supplementary Figure S2-20

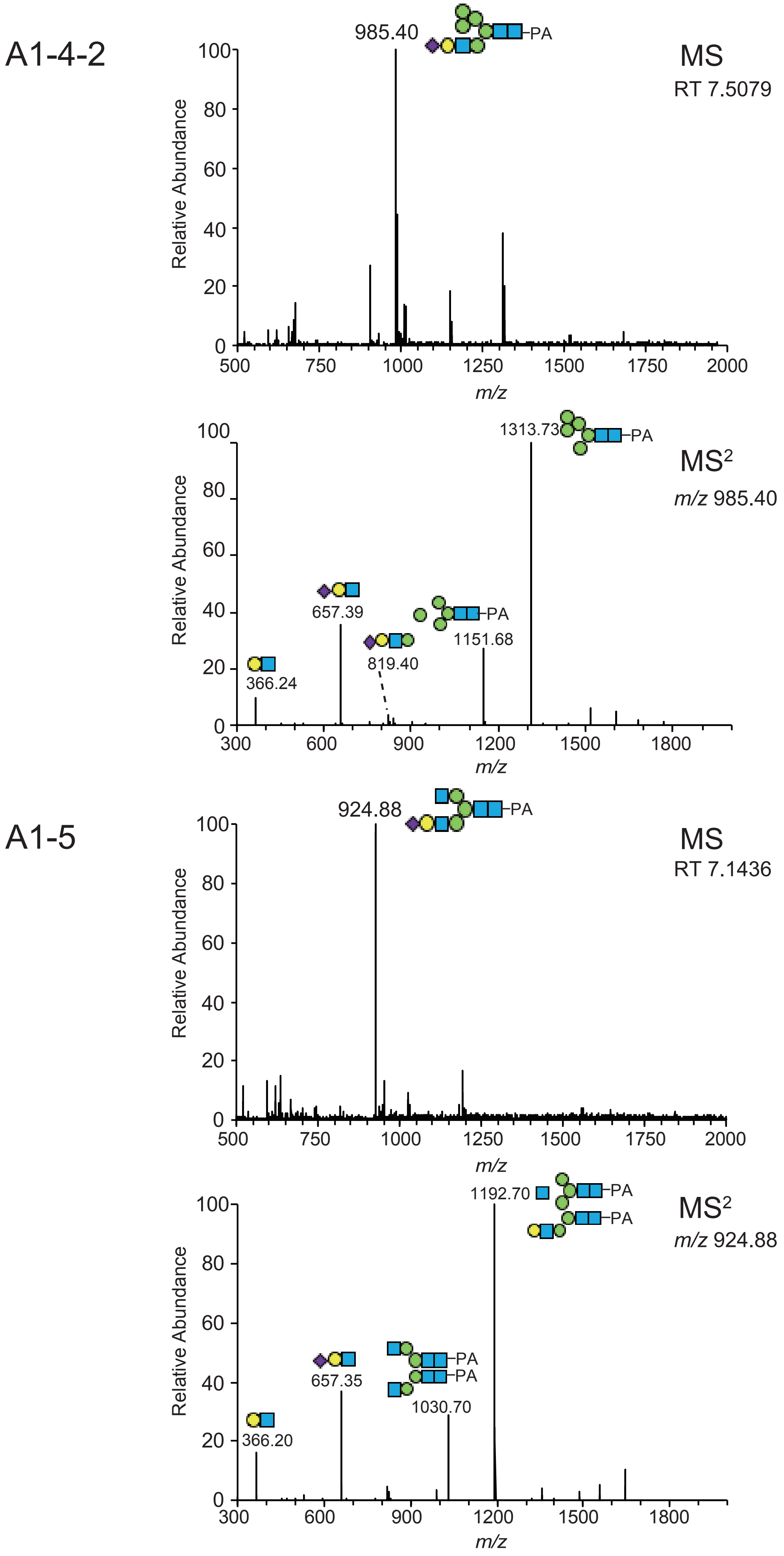

Supplementary Figure S2-21

A1-6

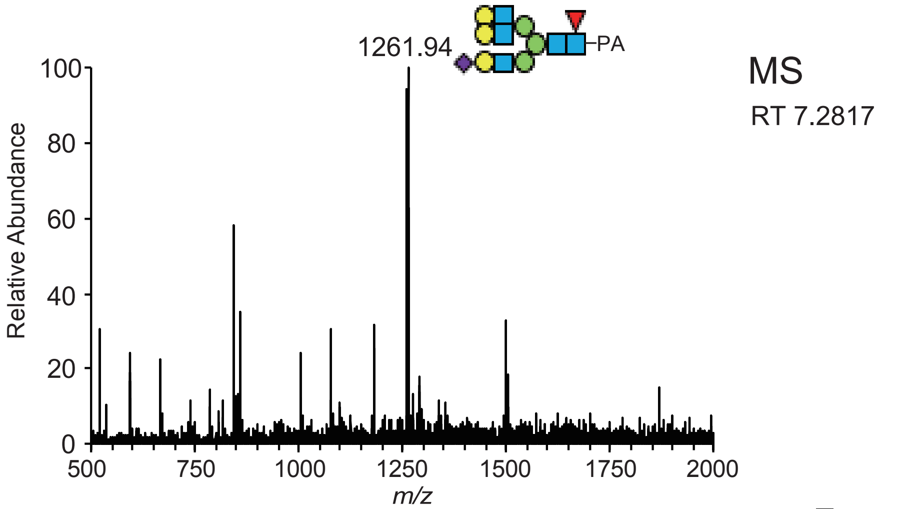

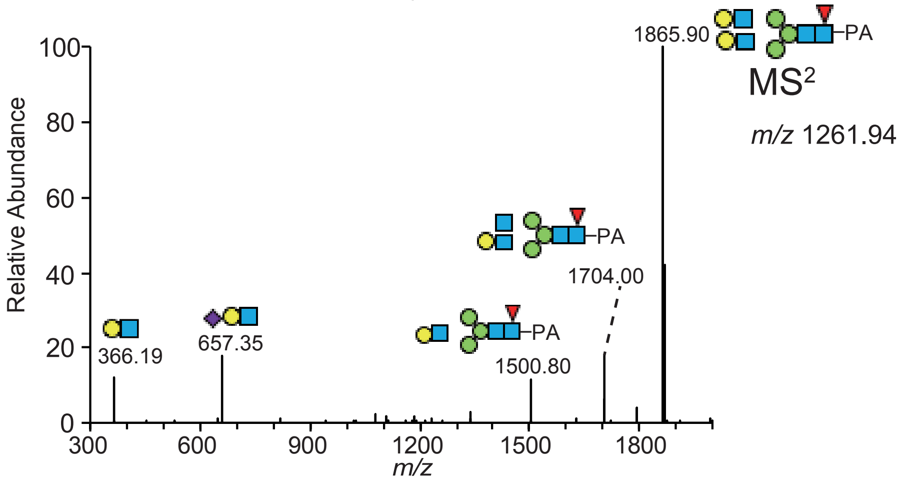

A1-8-1

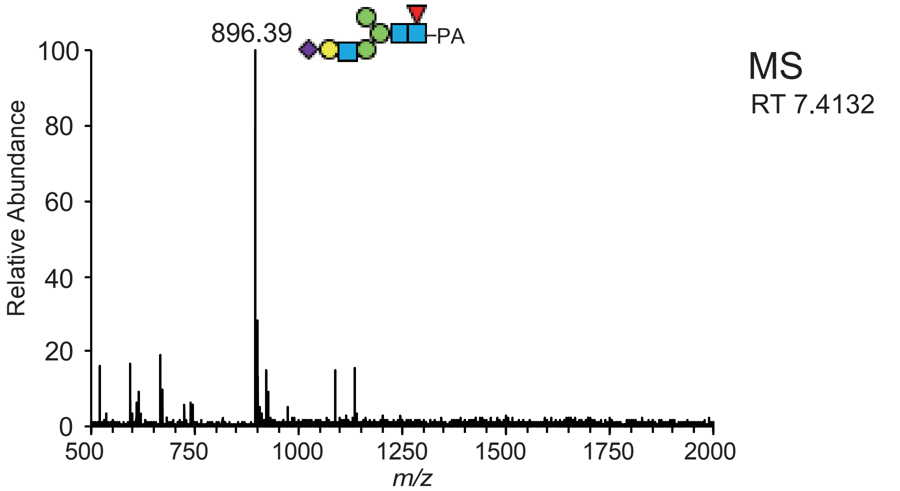

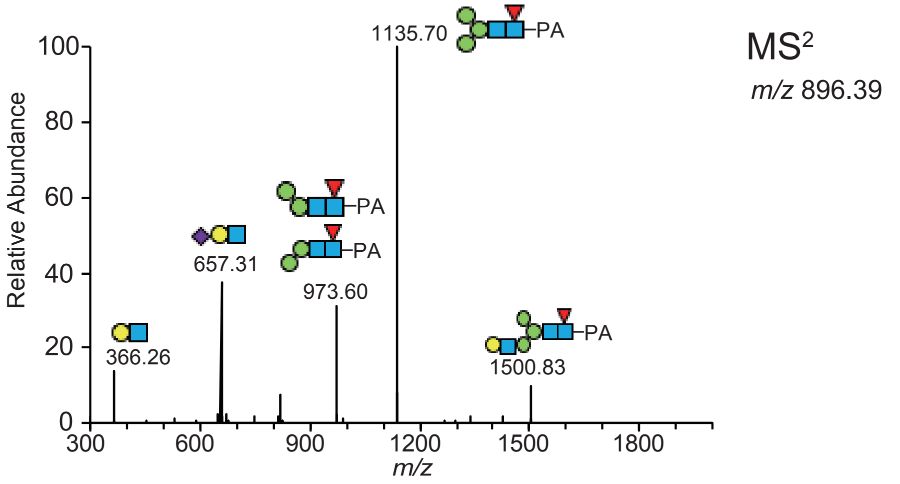

Supplementary Figure S2-22

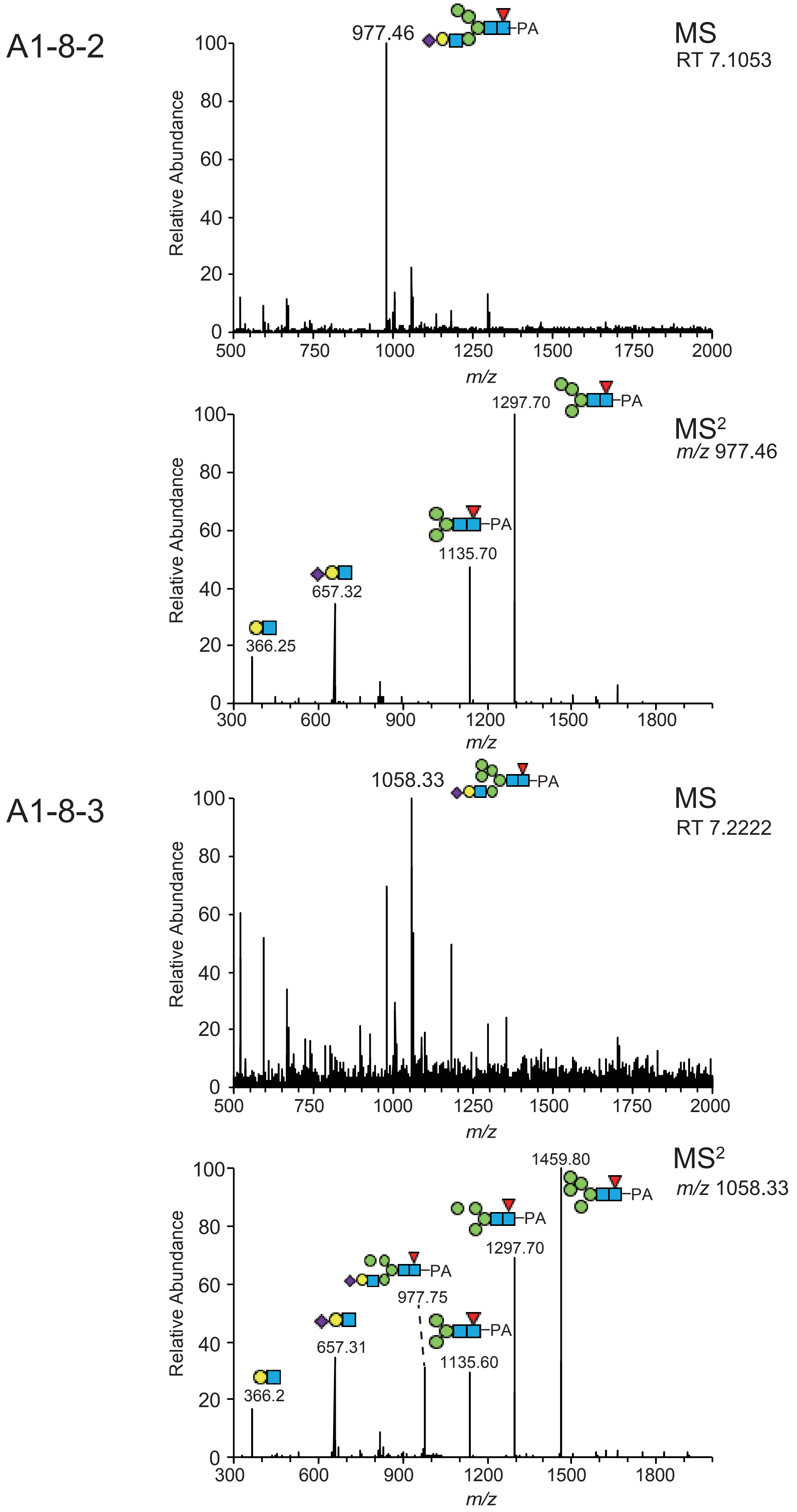

Supplementary Figure S2-23

A1-9

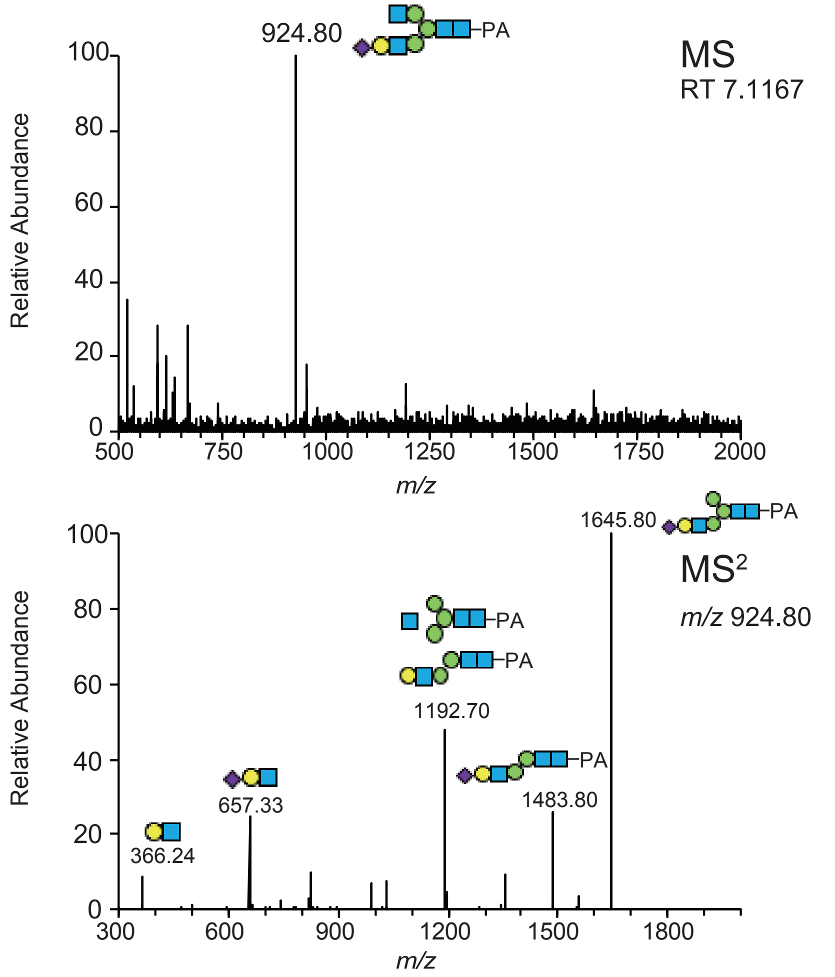

A1-11

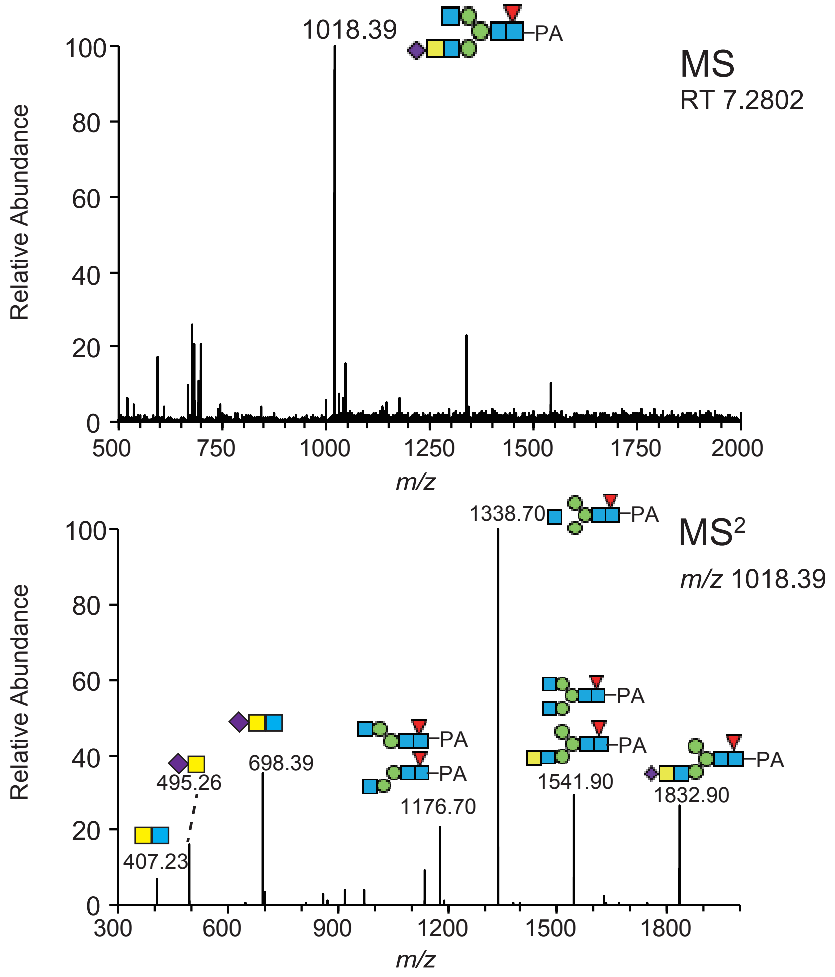

Supplementary Figure S2-24

A1-12

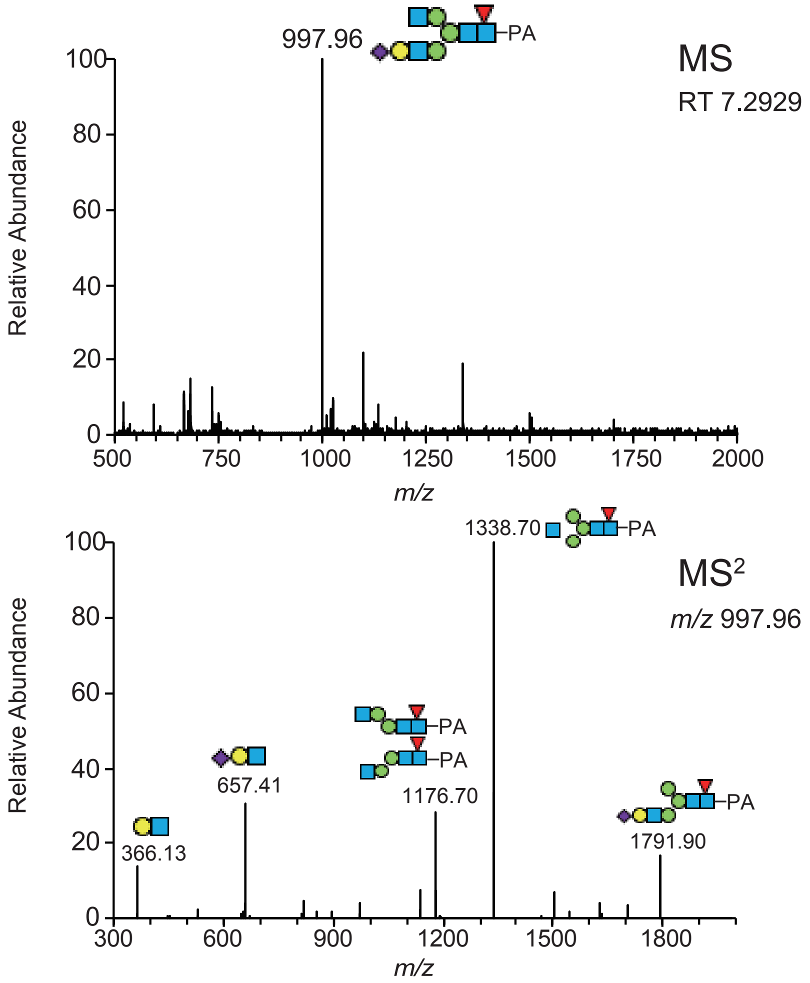

A1-13

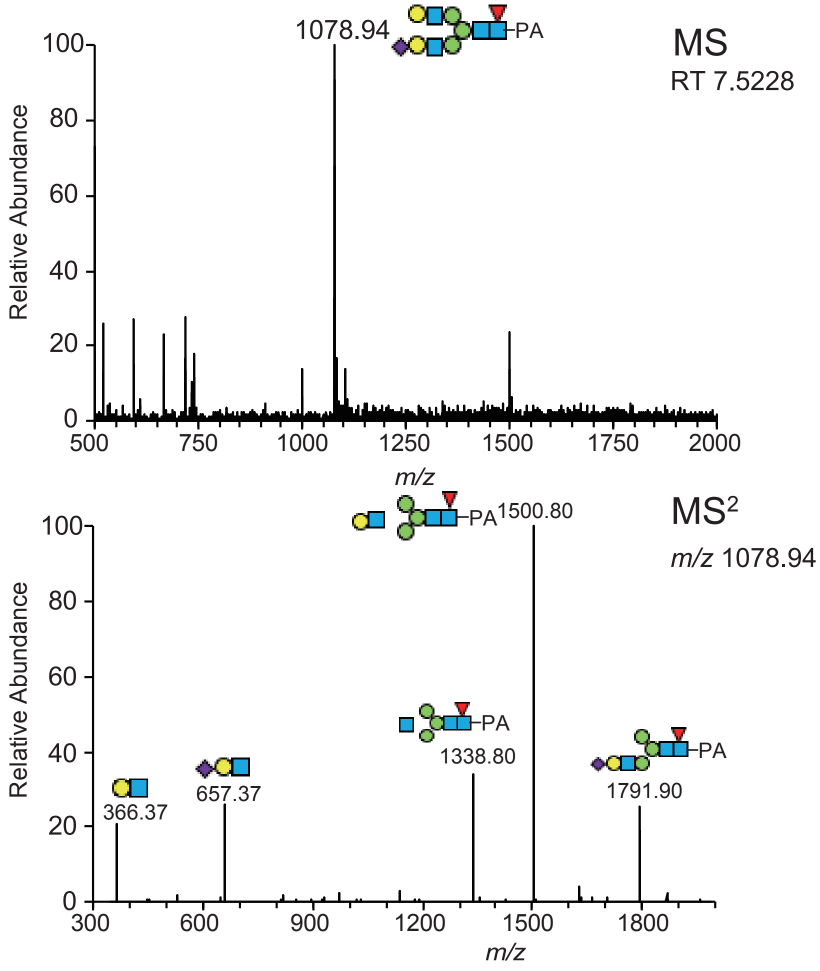

Supplementary Figure S2-25

A1-14-1

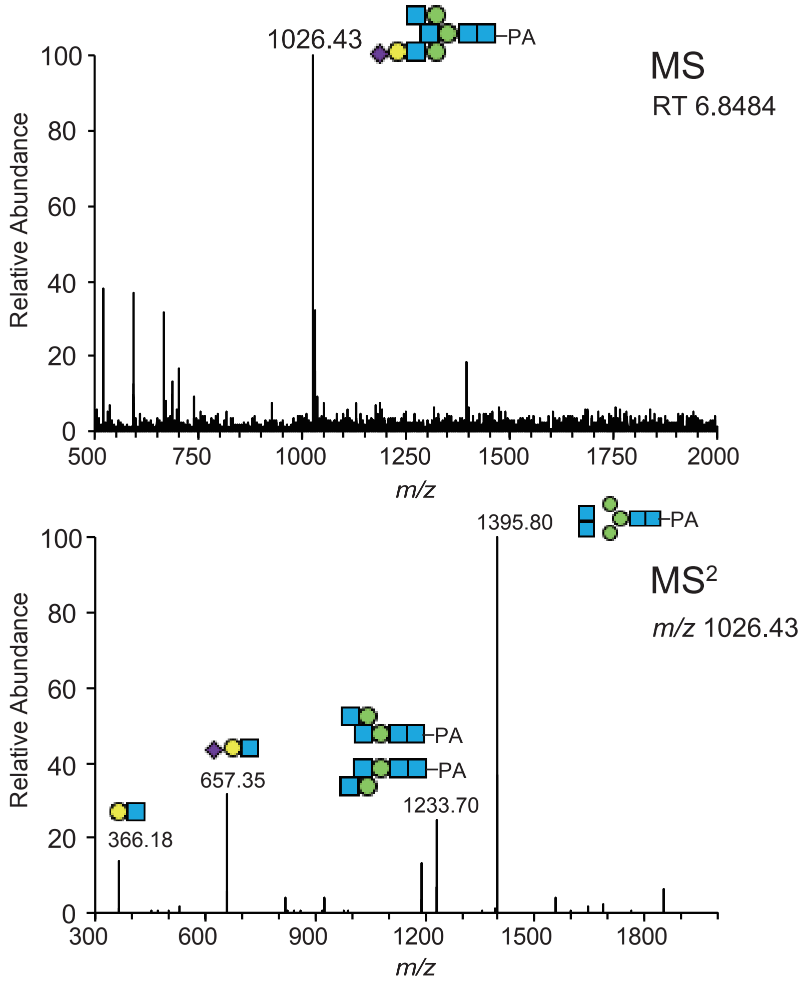

A1-14-2

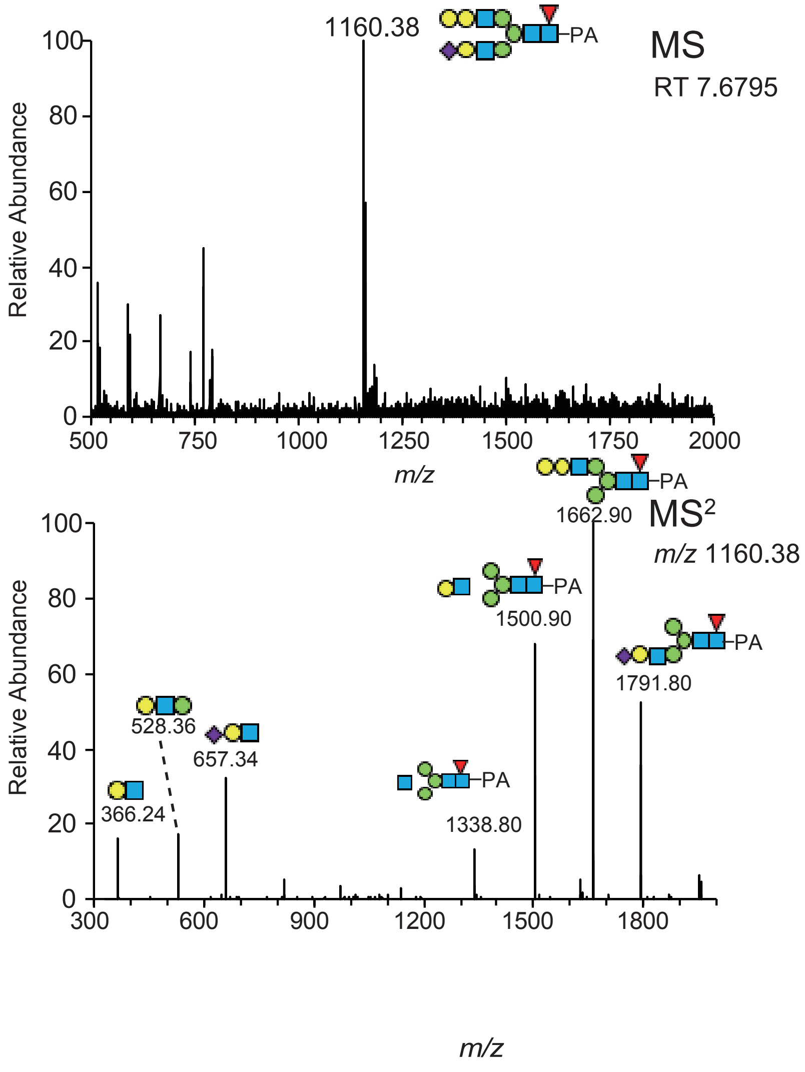

Supplementary Figure S2-26

A1-17-1

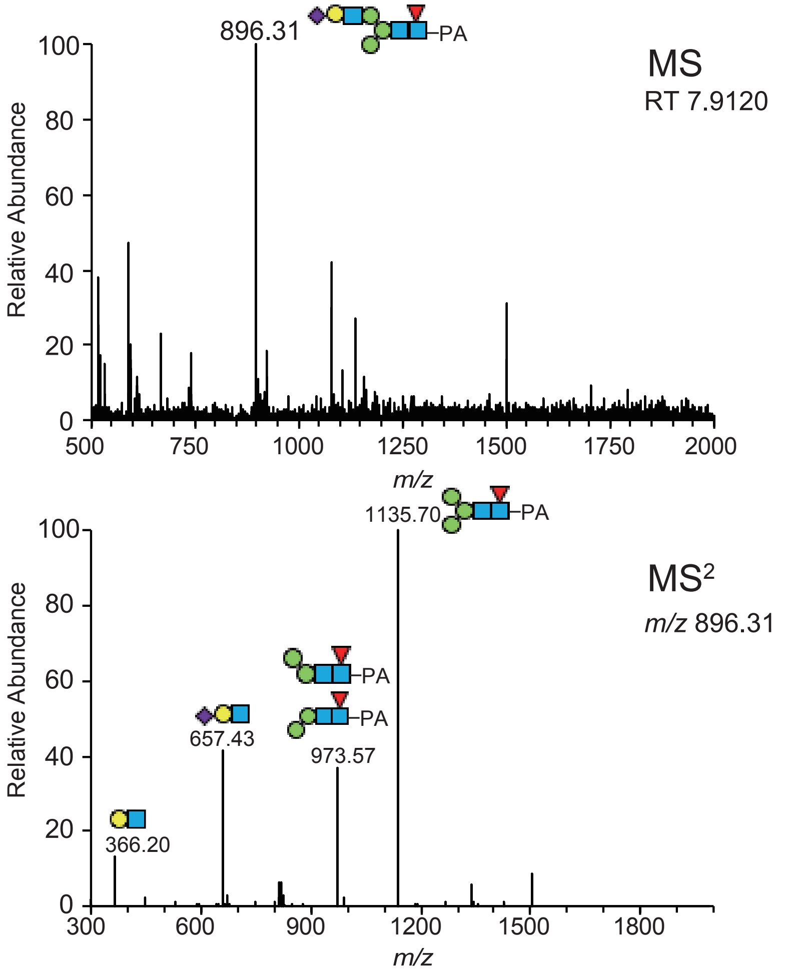

A1-17-2

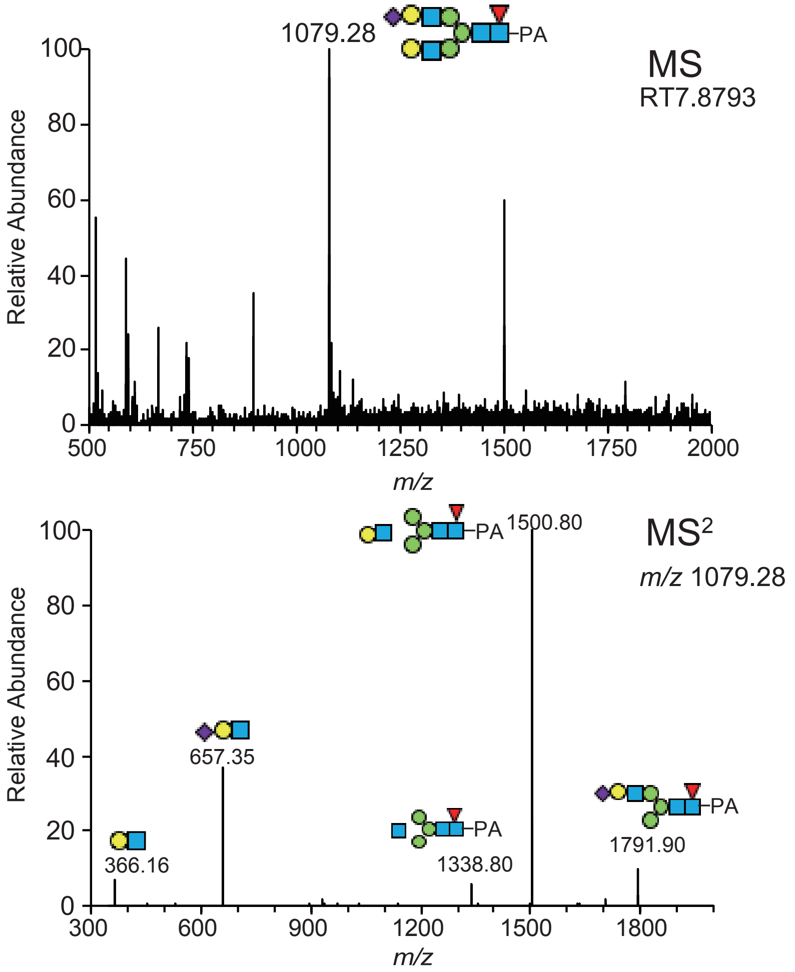

Supplementary Figure S2-27

A1-18

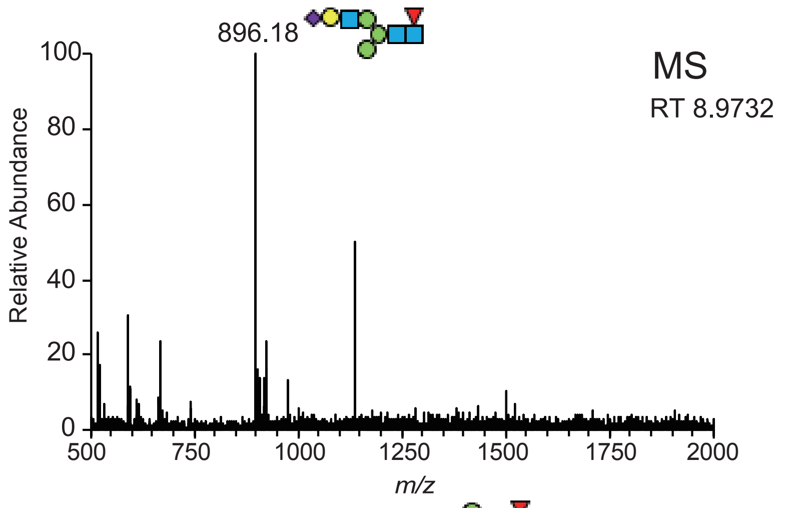

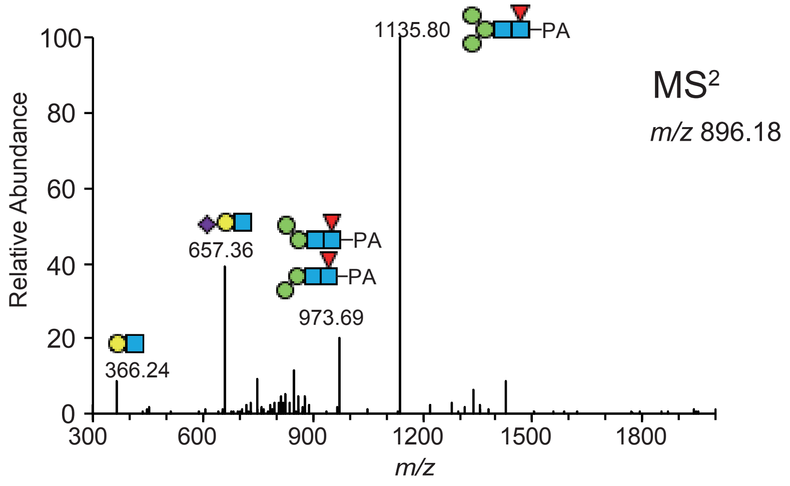

A1-19

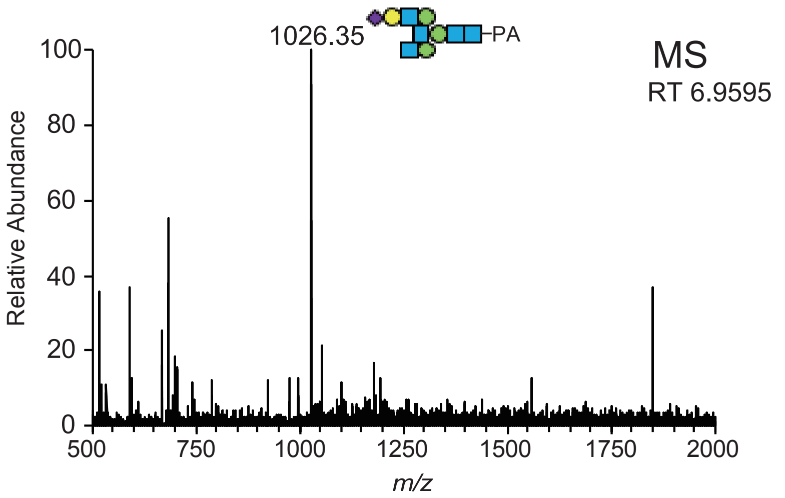

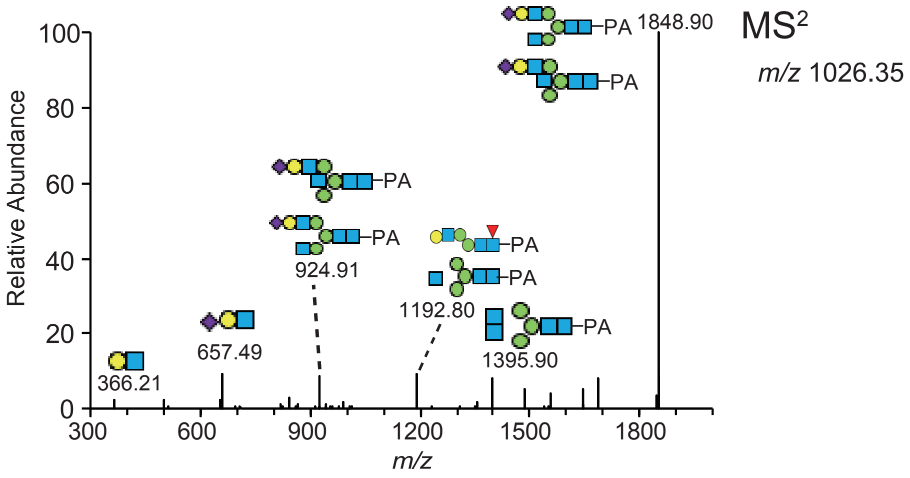

Supplementary Figure S2-28

A1-20

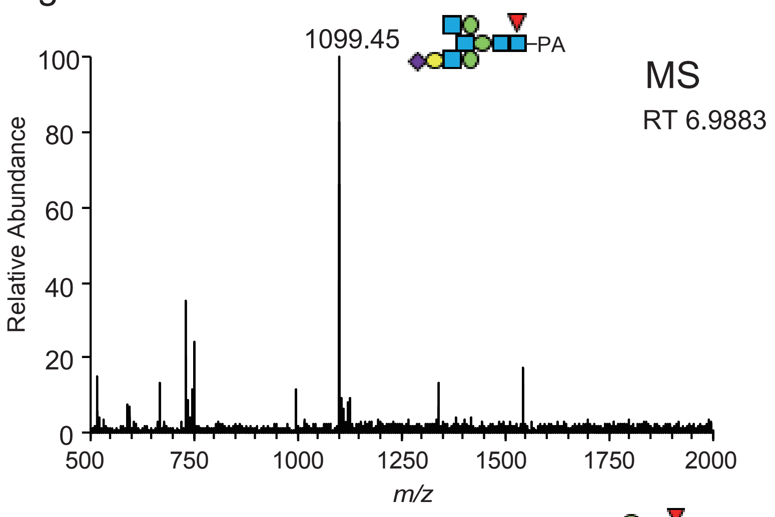

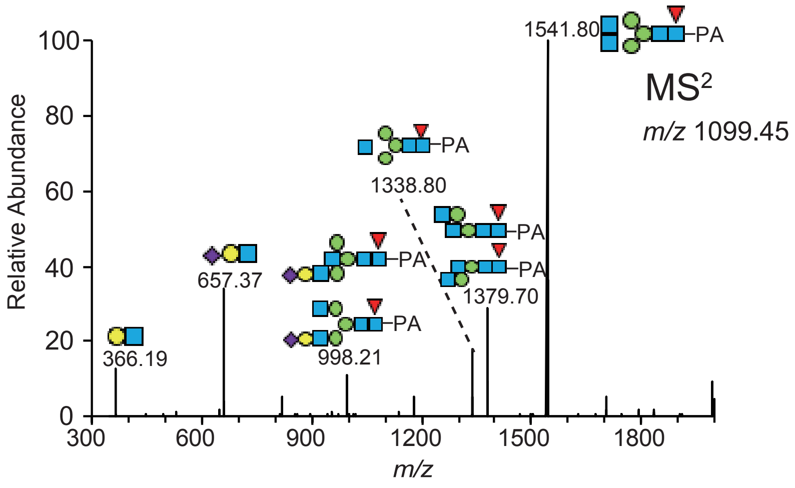

A1-21

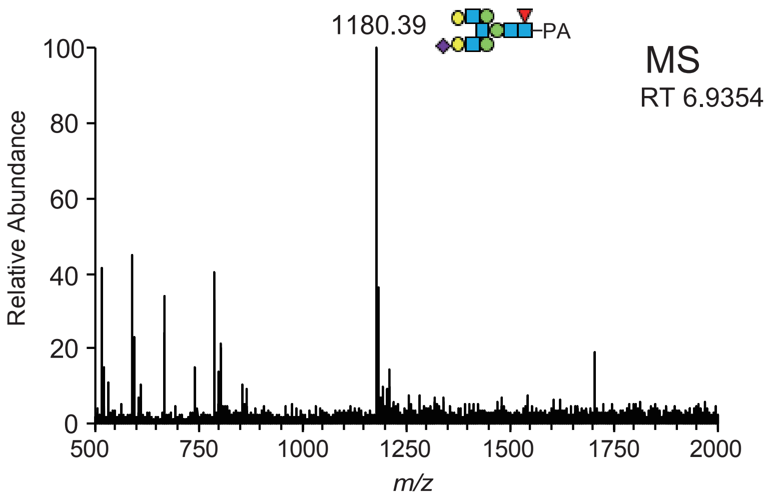

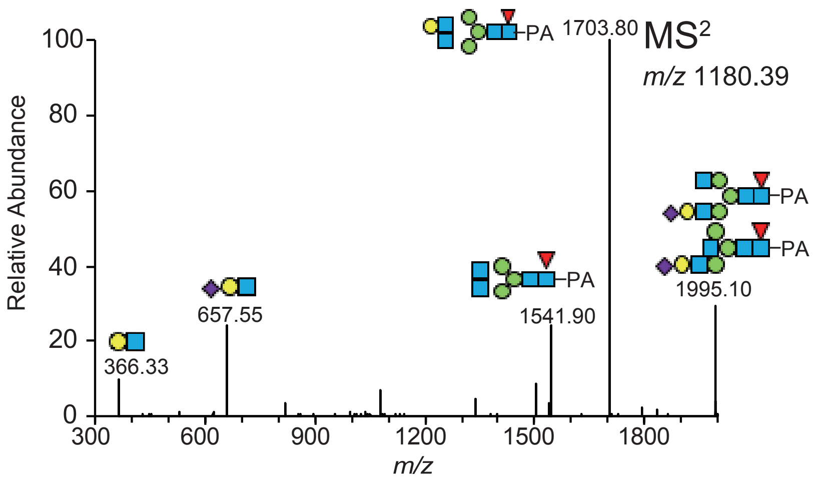

Supplementary Figure S2-29

A1-22

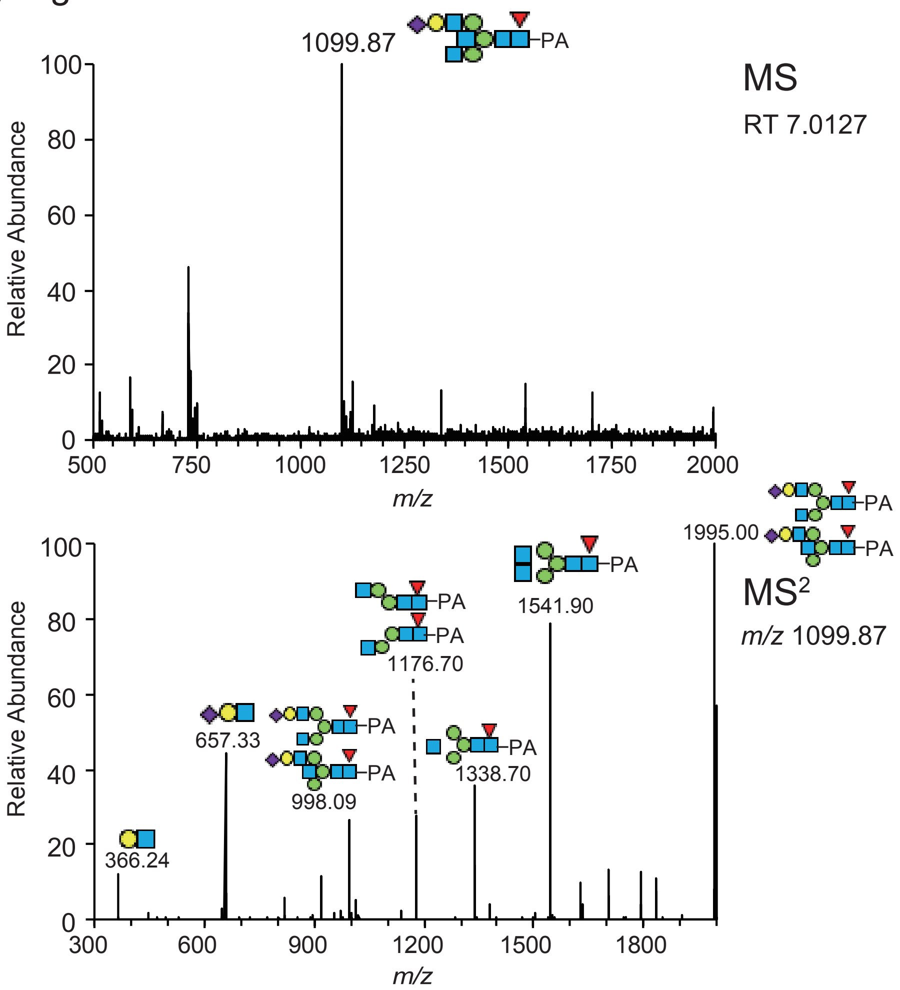

Supplementary Figure S2-30

A2-1

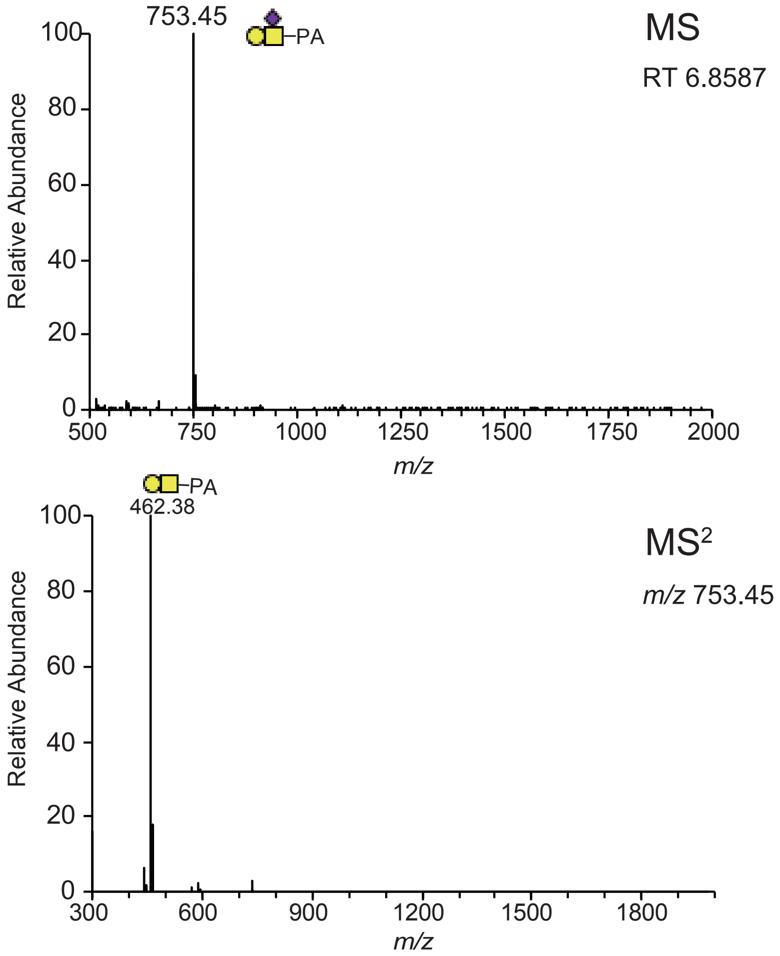

A2-2-1

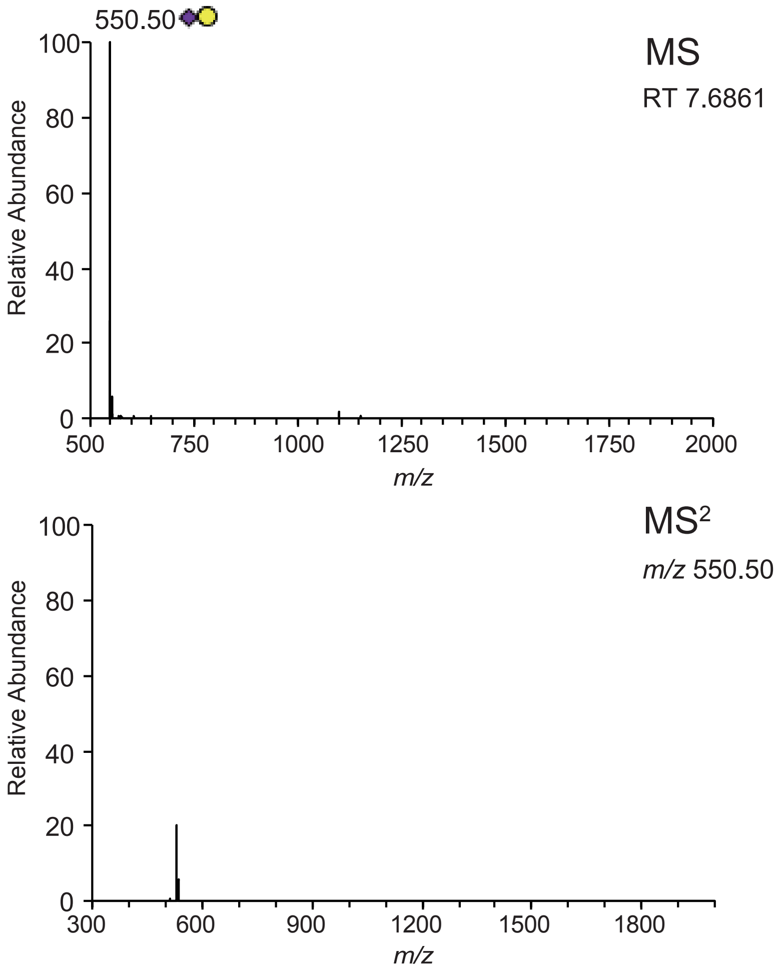

Supplementary Figure S2-31

A2-2-2

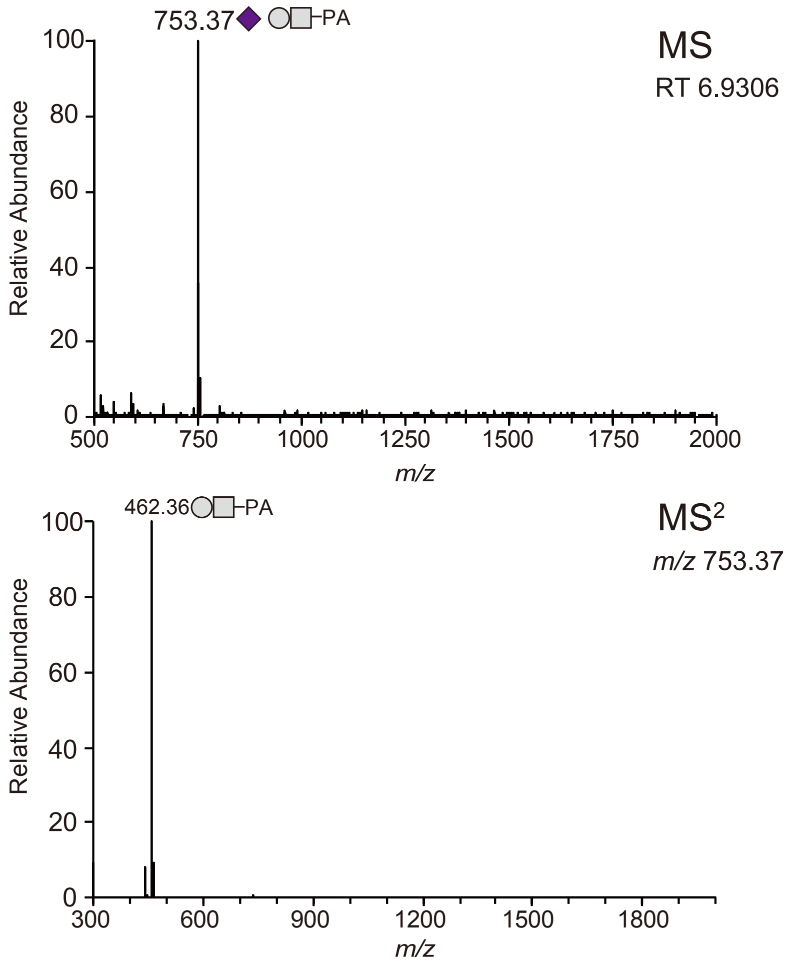

A2-3

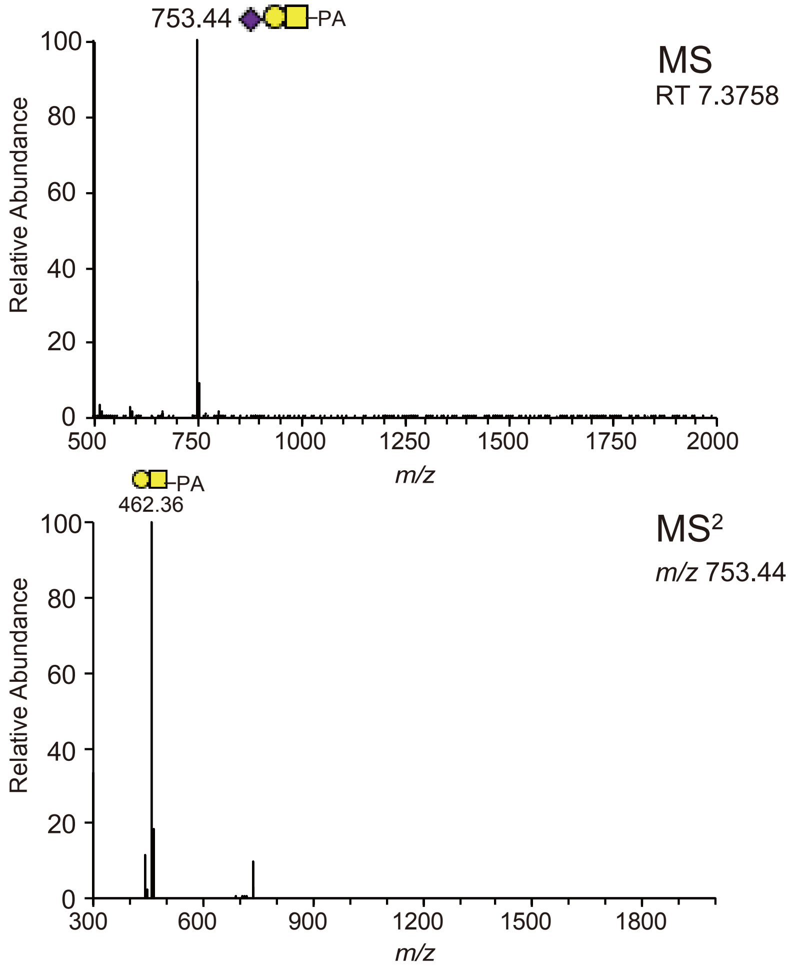

Supplementary Figure S2-32

A2-4

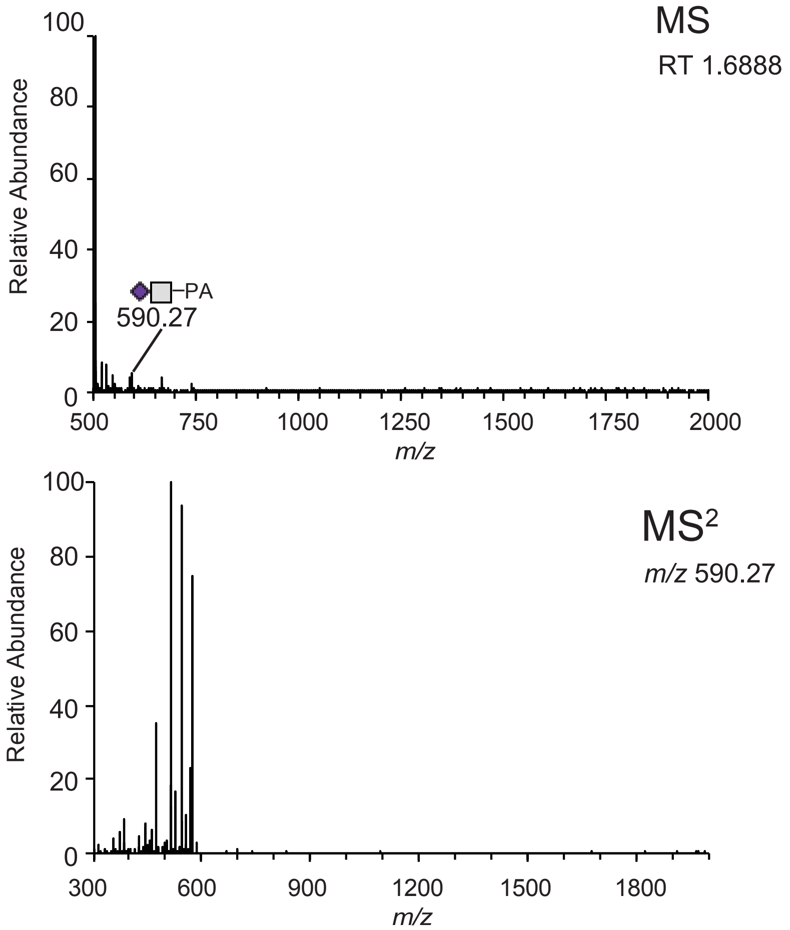

A2-5

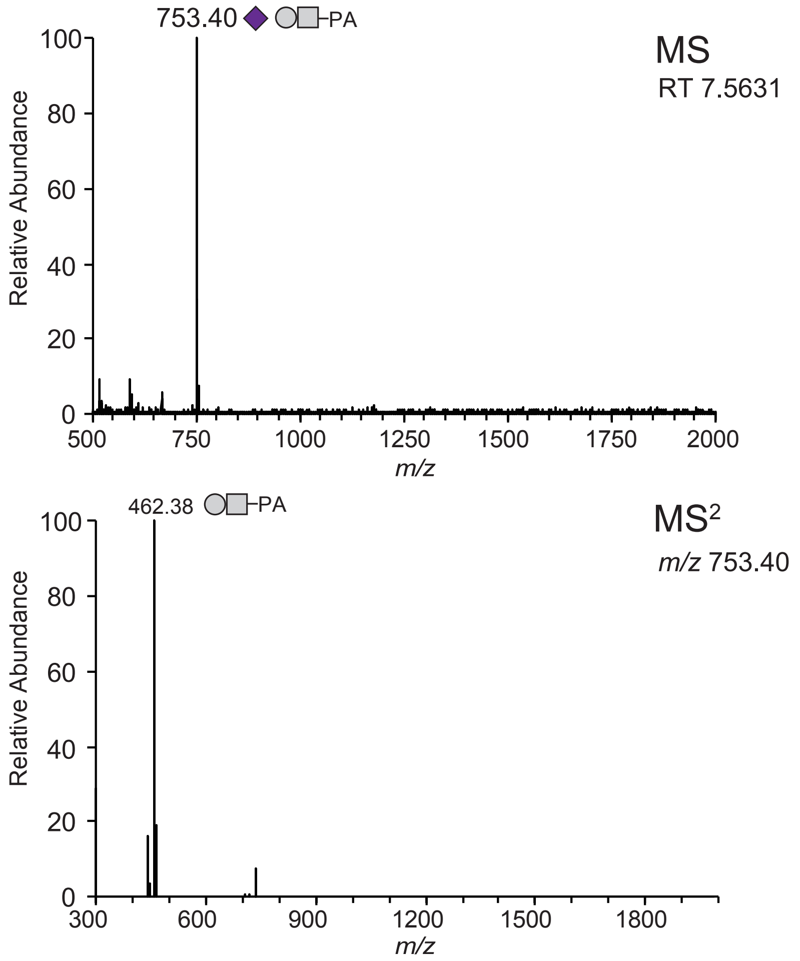

Supplementary Figure S2-33

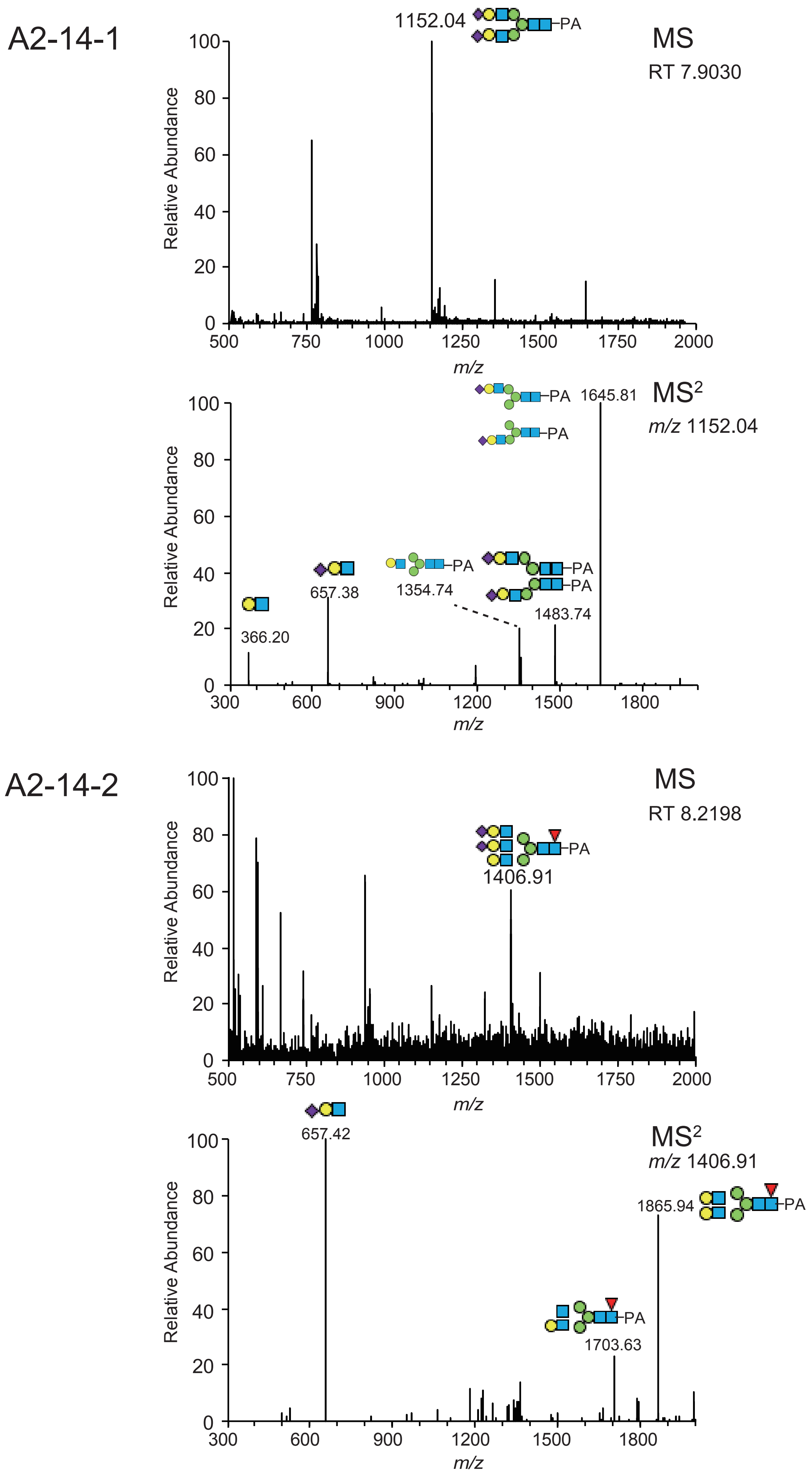

Supplementary Figure S2-34

A2-15

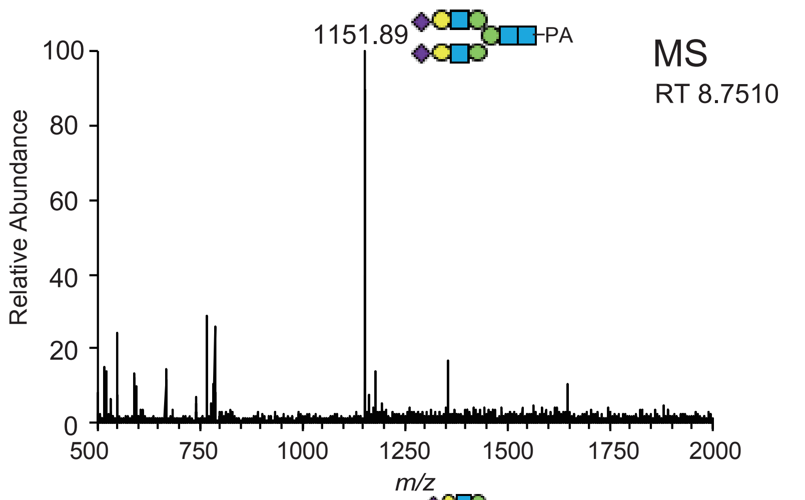

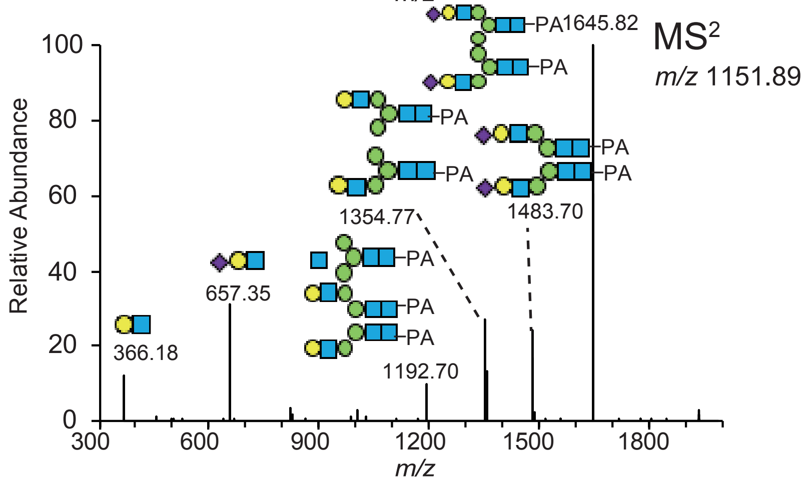

A2-16-1

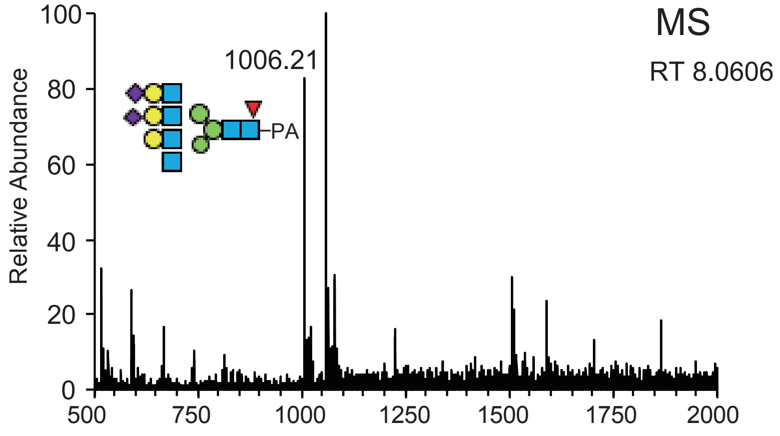

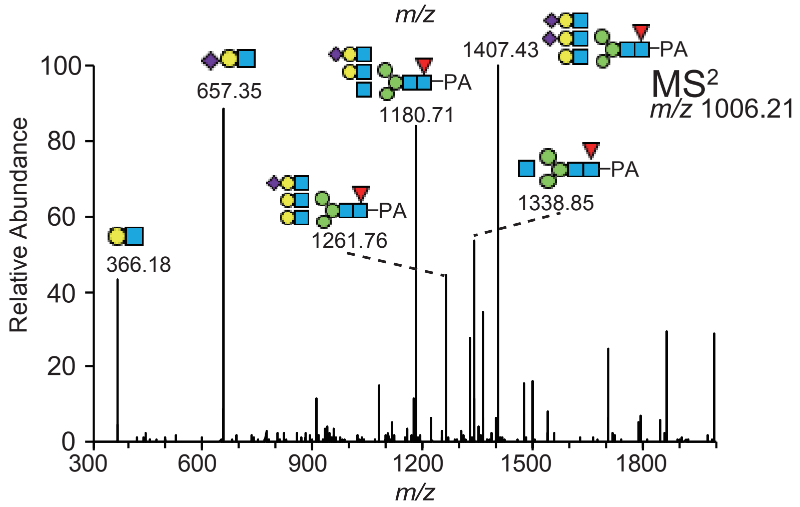

Supplementary Figure S2-35

A2-16-2

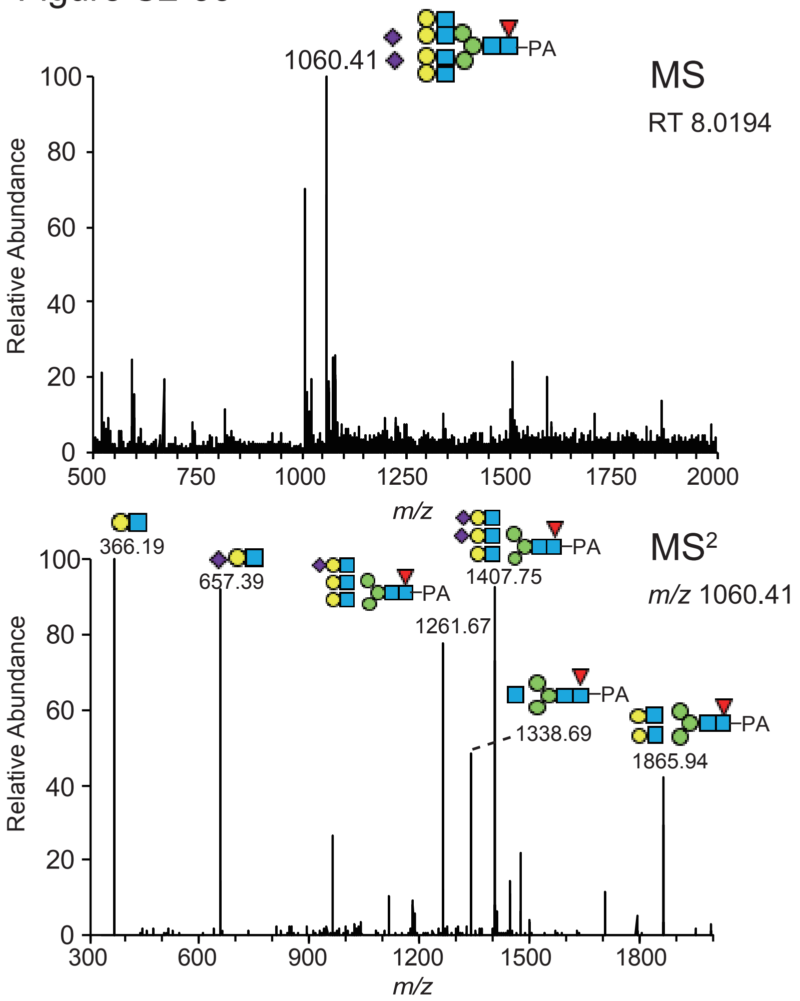

A2-17

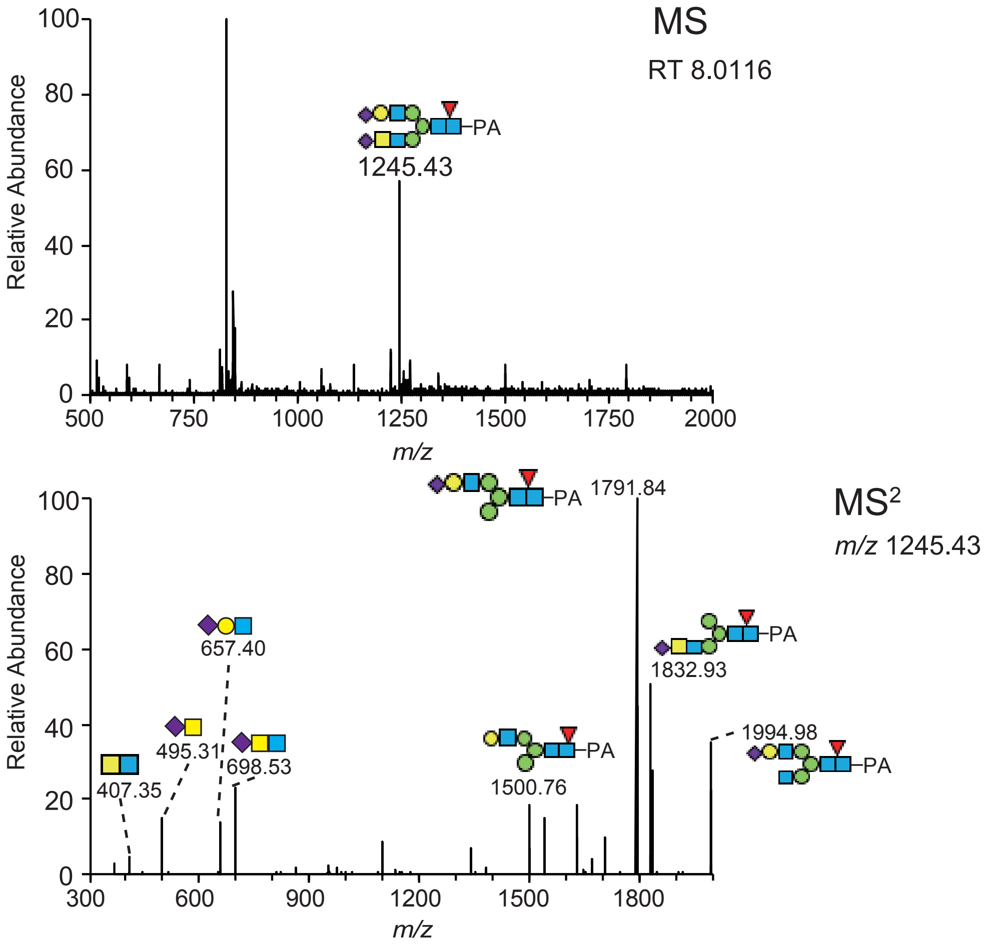

Supplementary Figure S2-36

A2-18

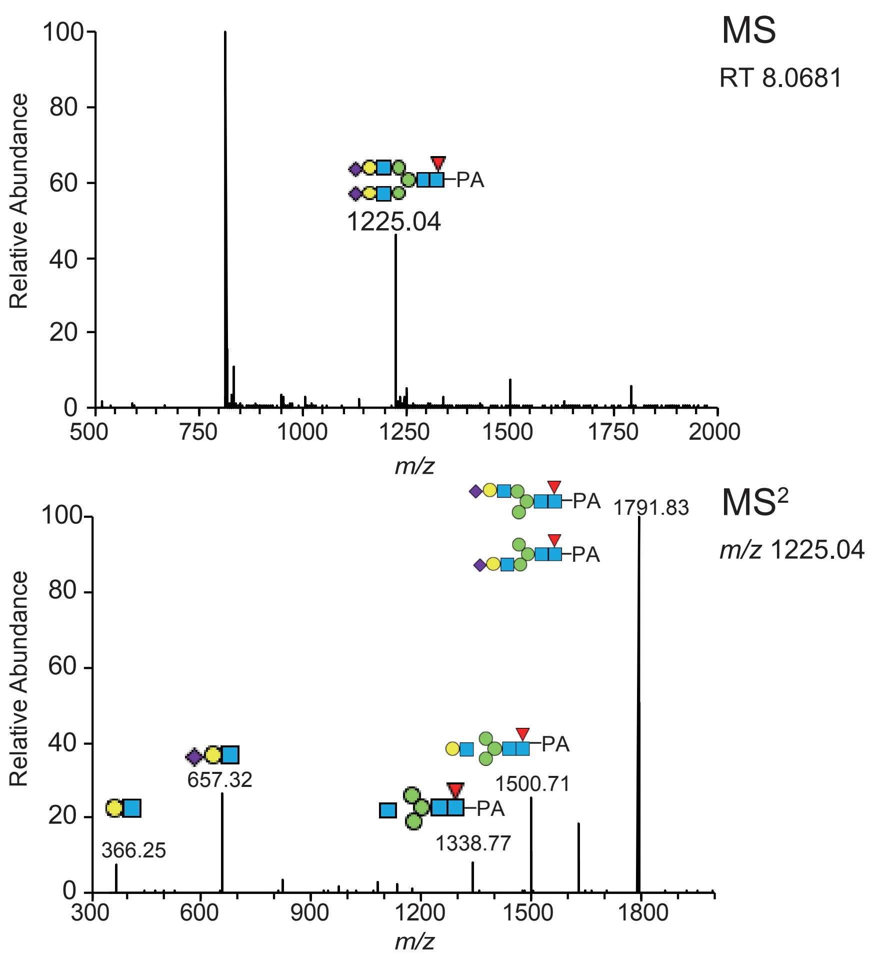

A2-19

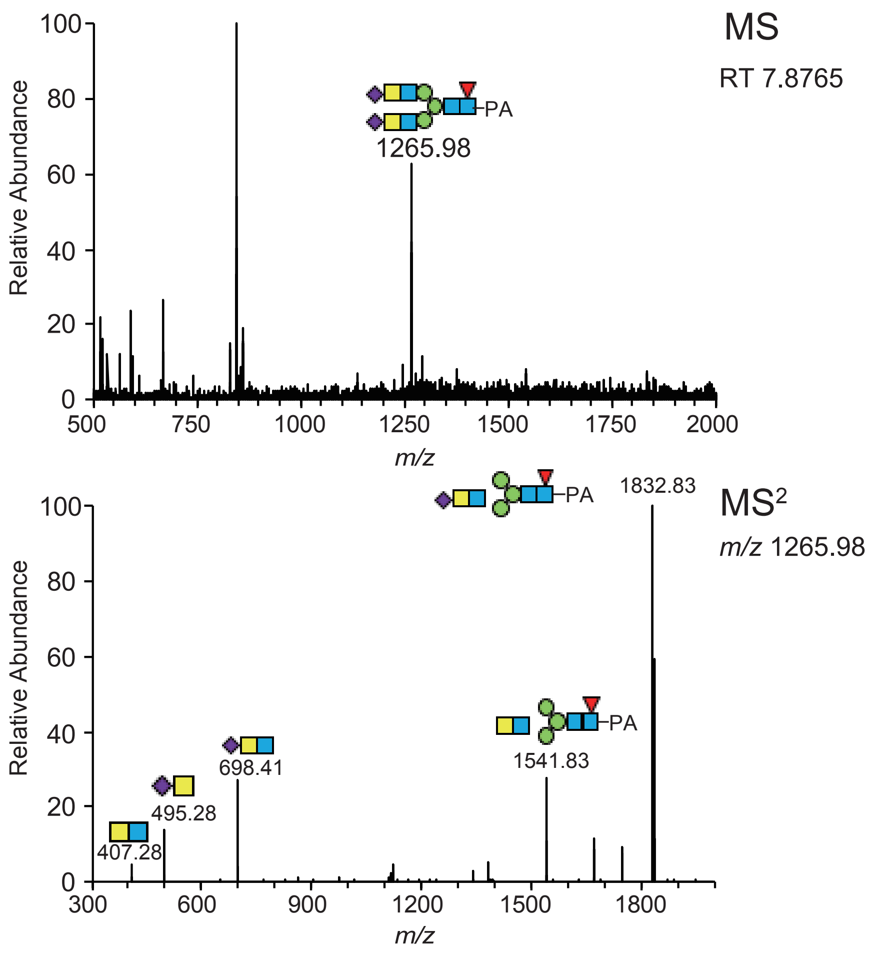

Supplementary Figure S2-37

A2-20

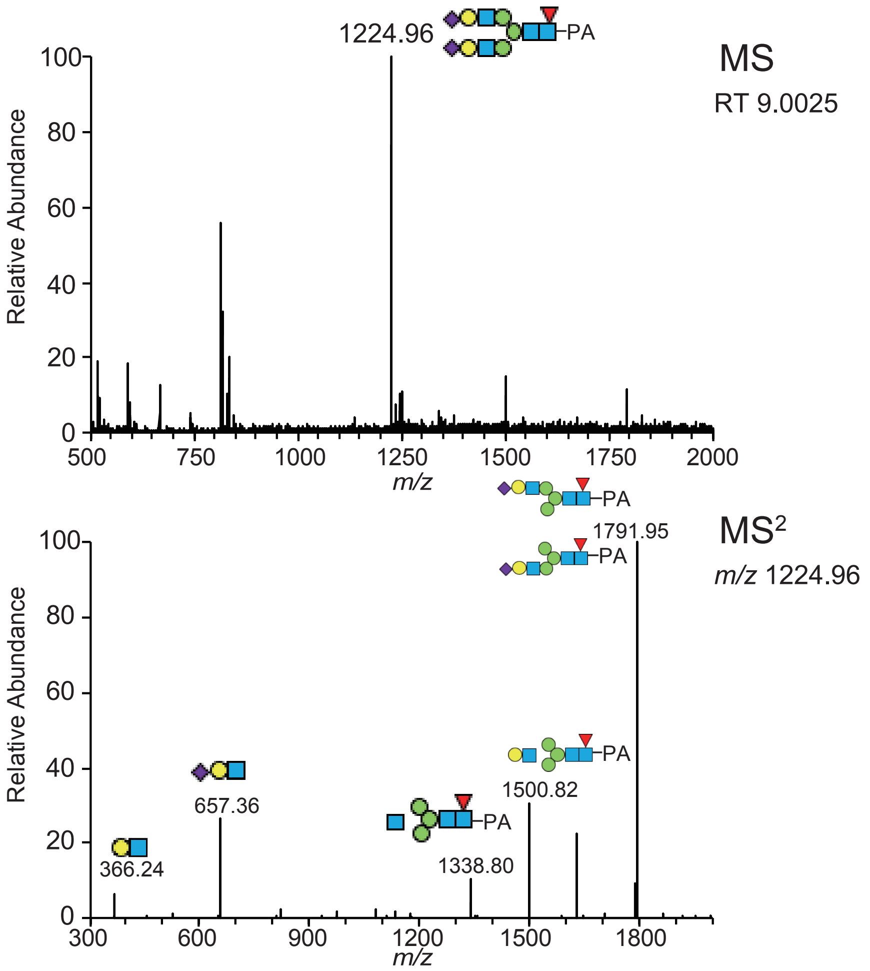

A2-23

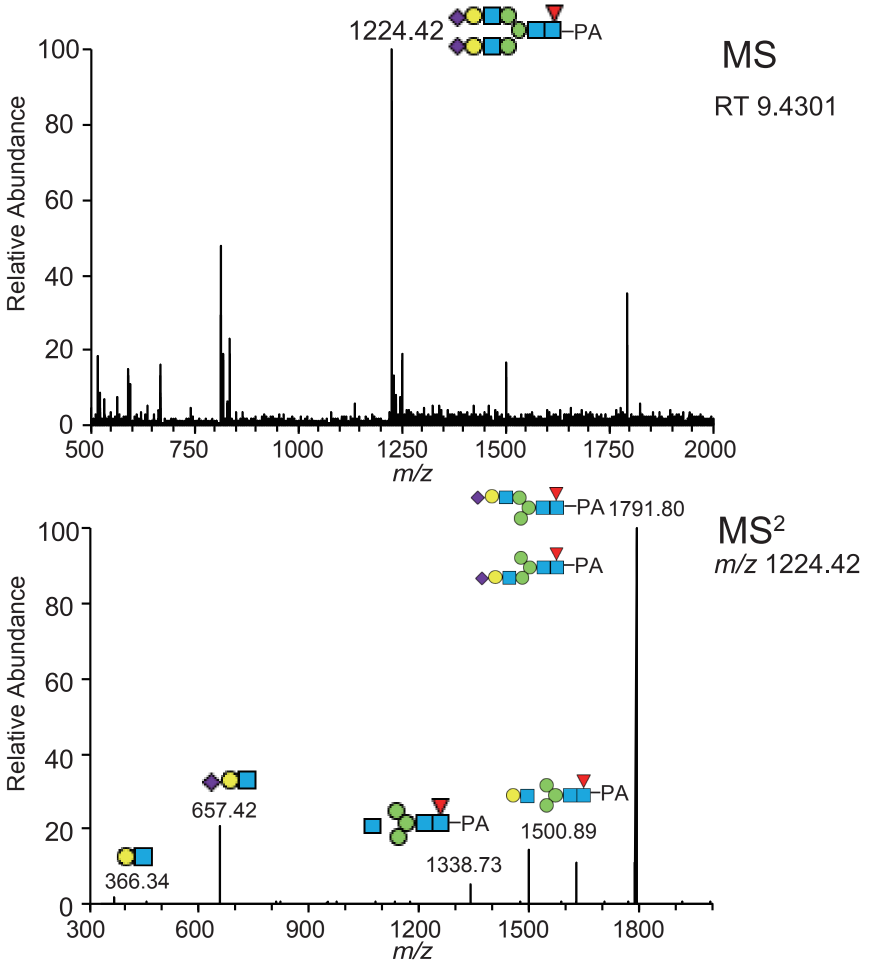

Supplementary Figure S2-38

A2-24

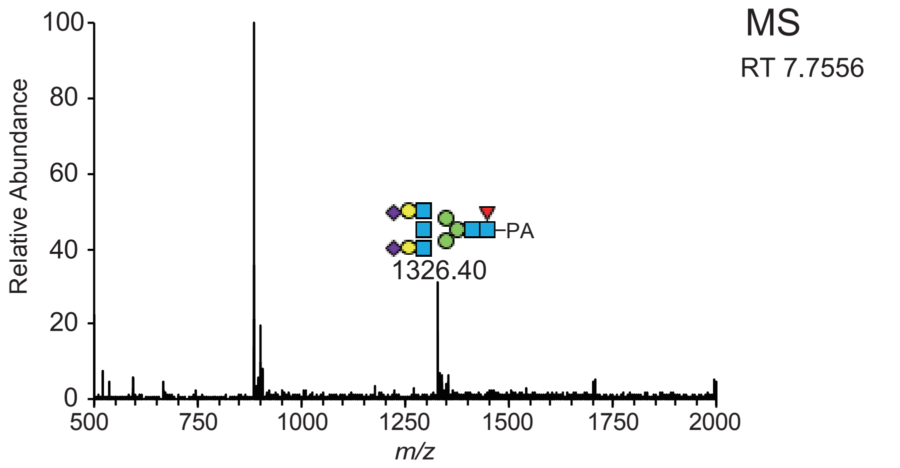

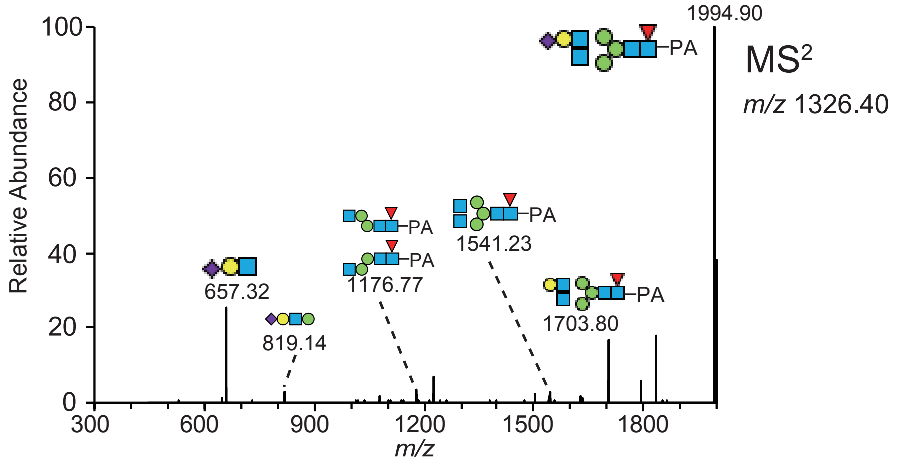

A2-25

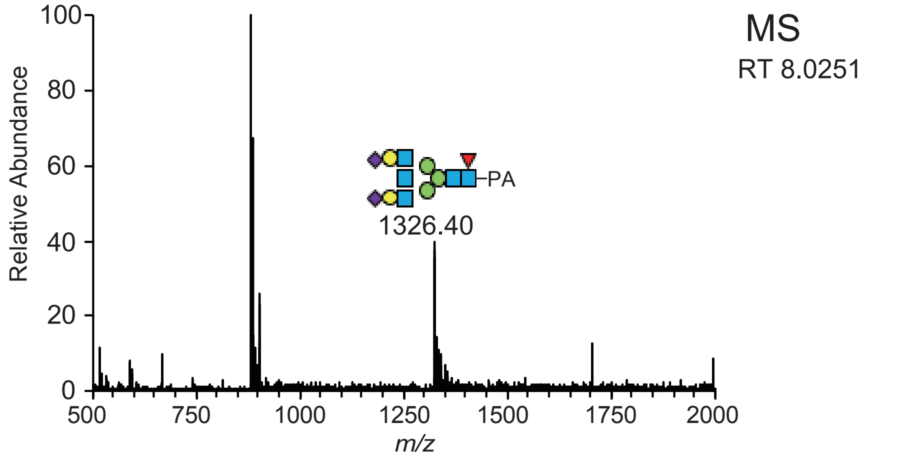

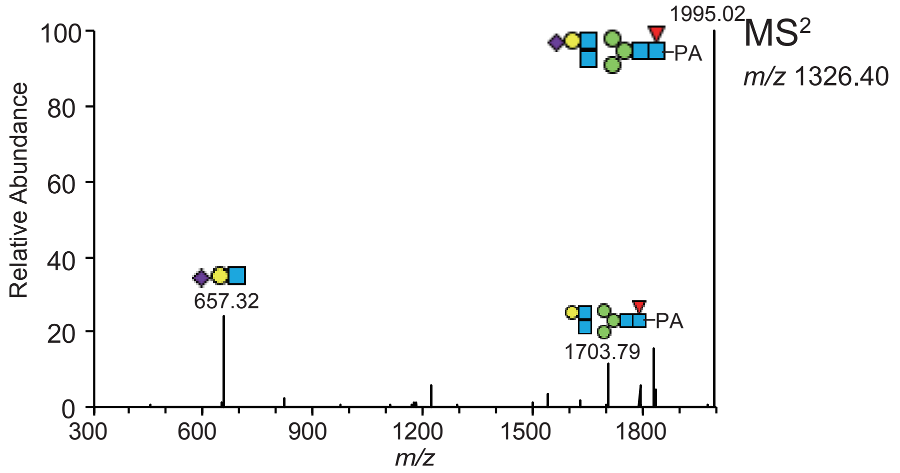

Supplementary Figure S2-39

A2-27

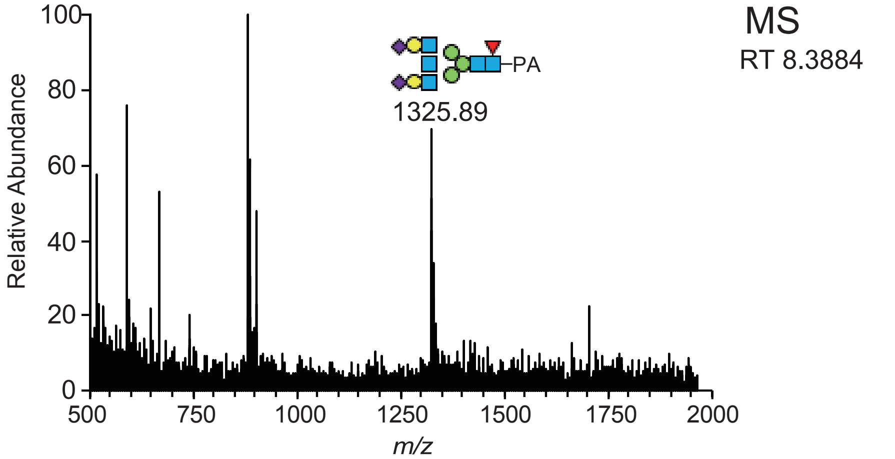

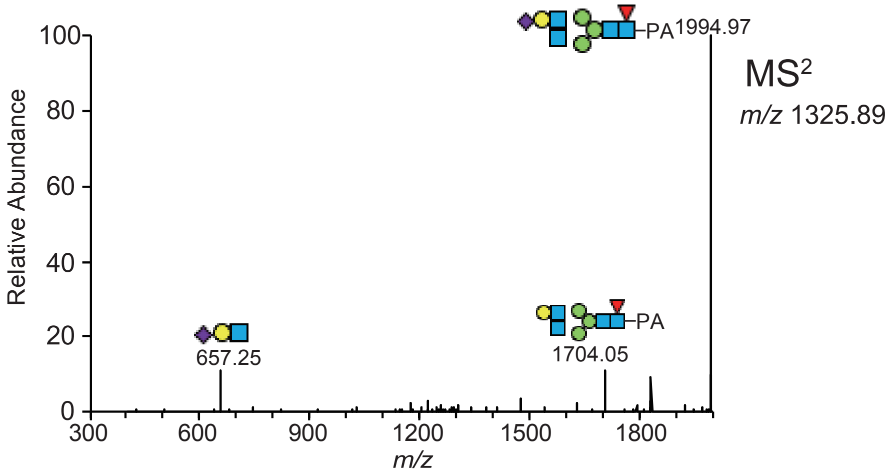

Supplementary Figure S2-40

A3-3

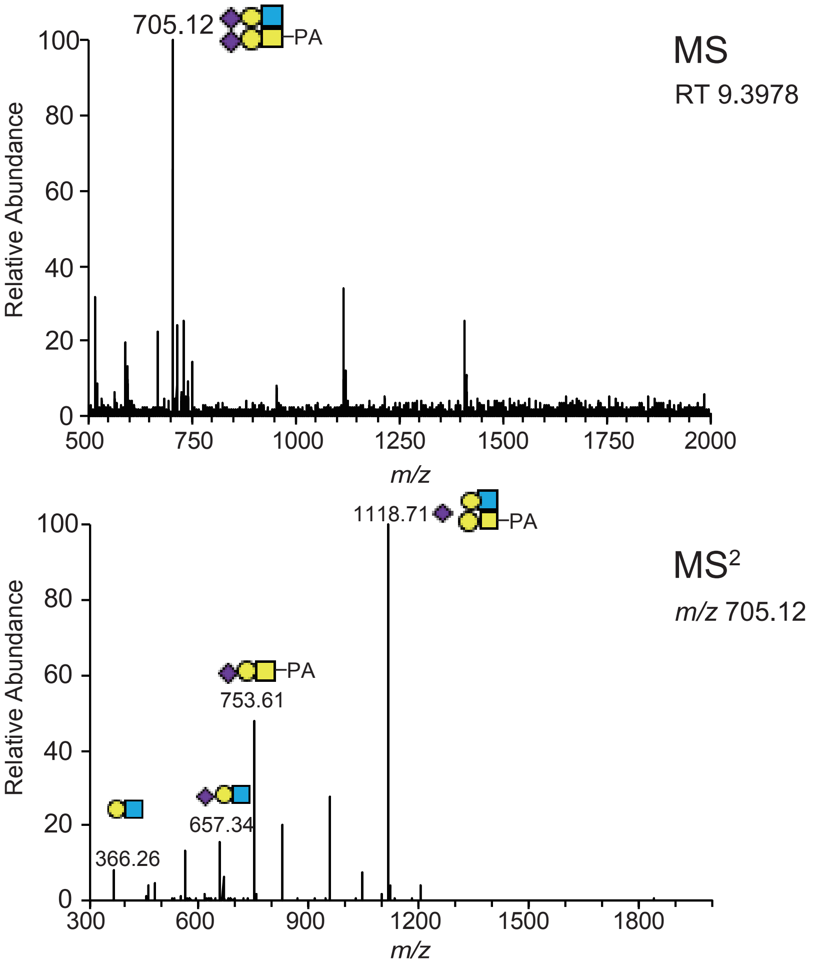

A3-10

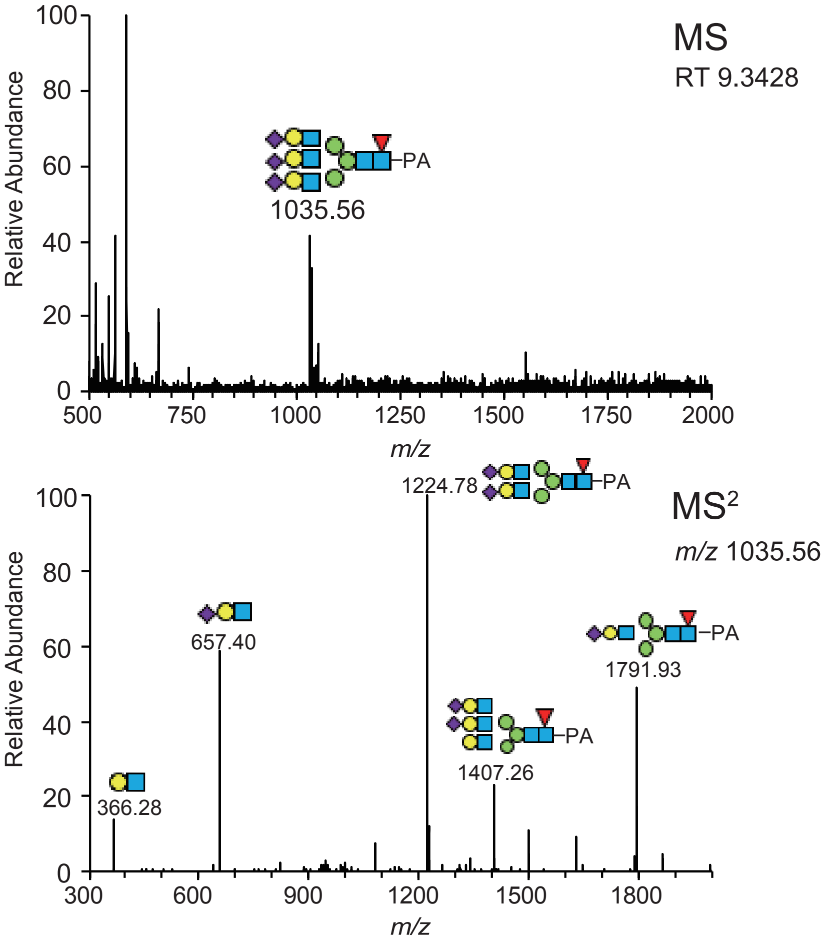

Supplementary Figure S2-41

A3-12

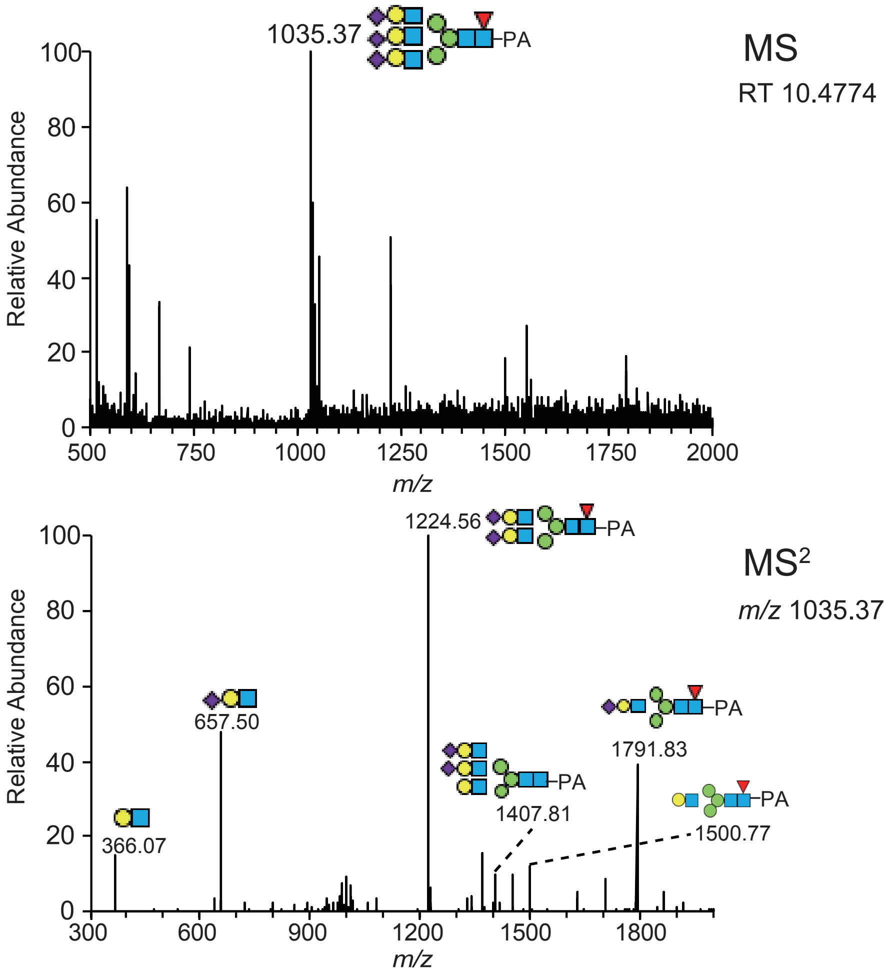

A3-13

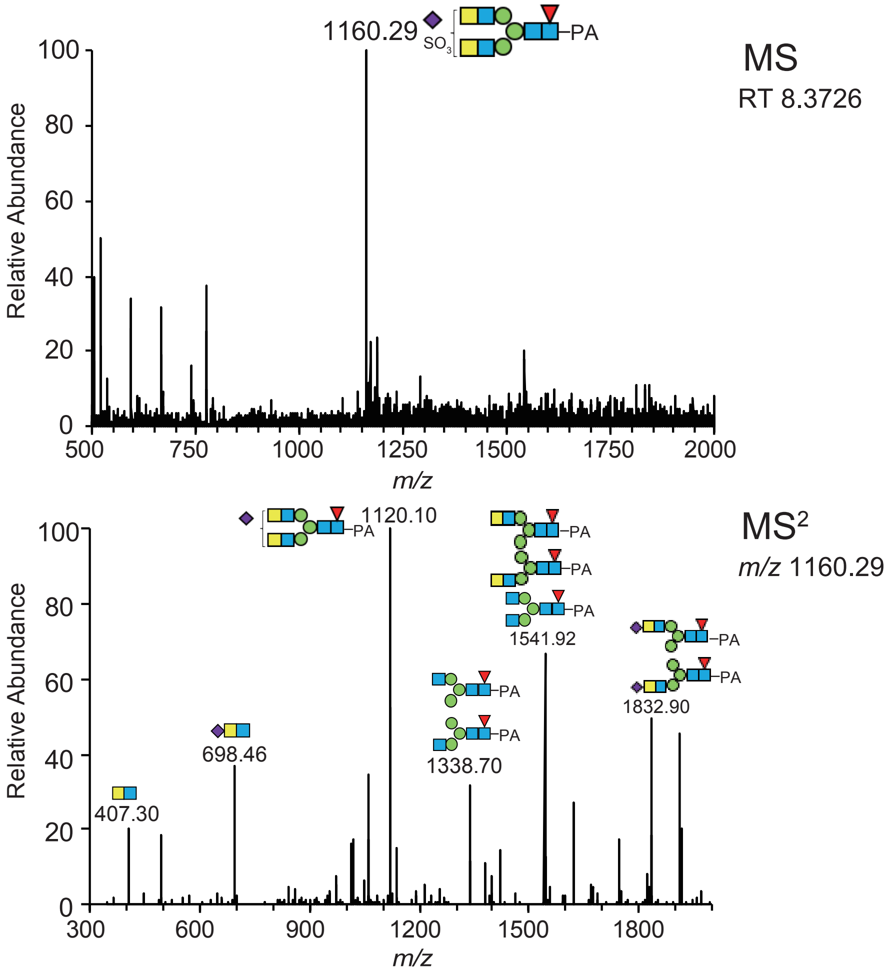

Supplementary Figure S2-42

A3-14

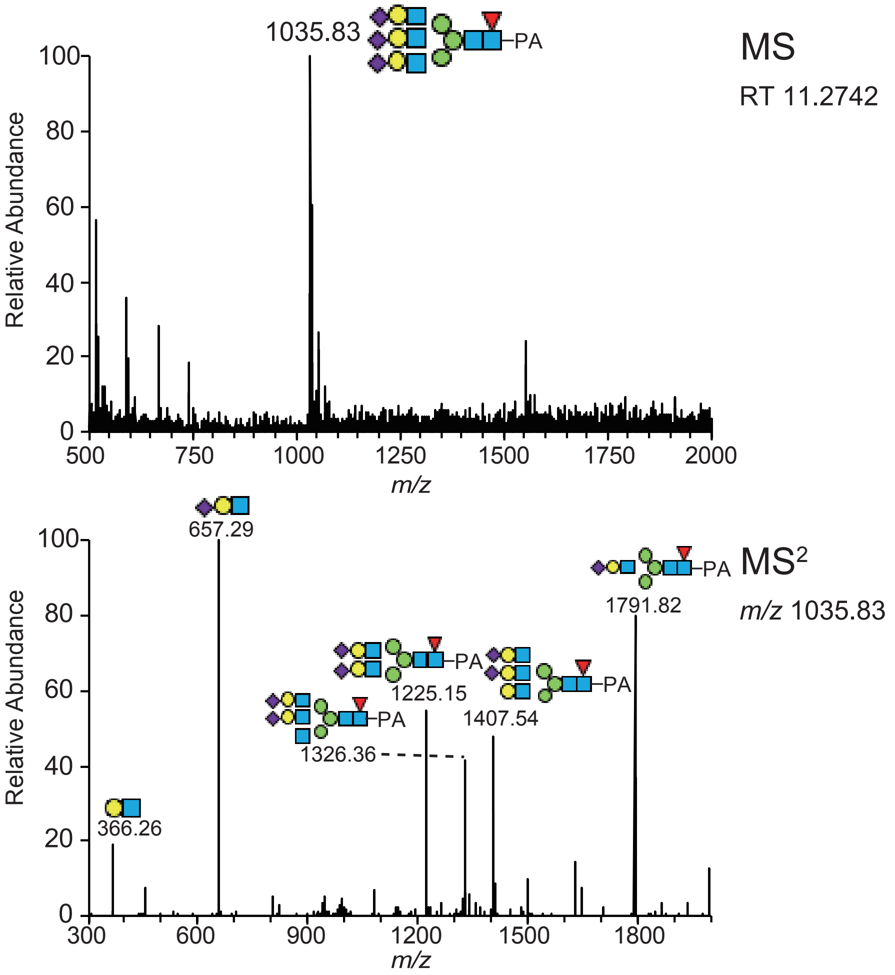

A3-15-1

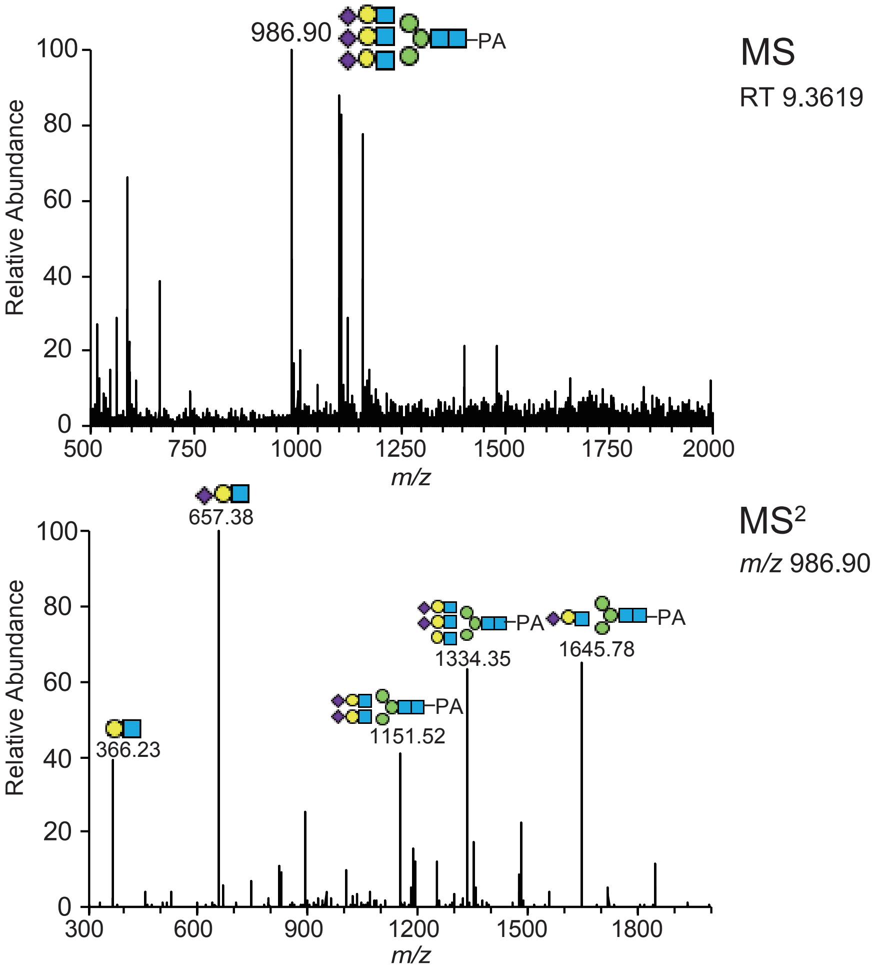

Supplementary Figure S2-43

A3-15-2

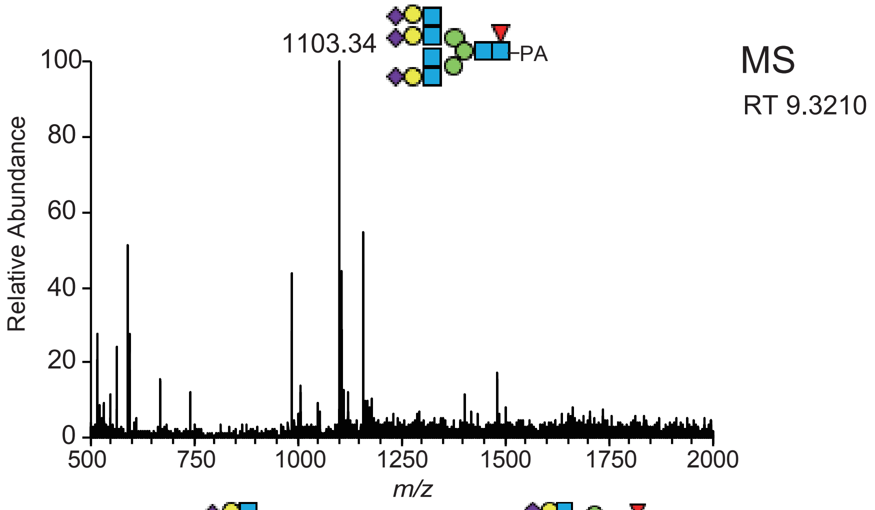

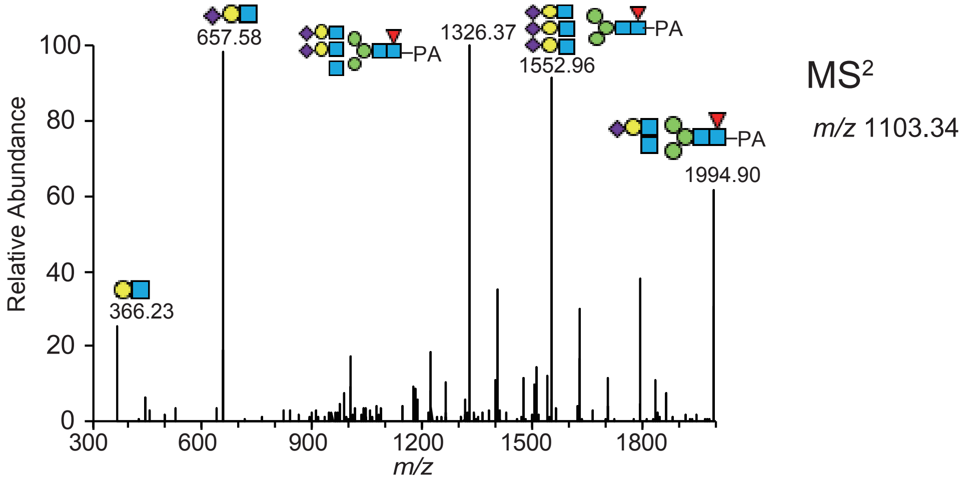

A3-15-3

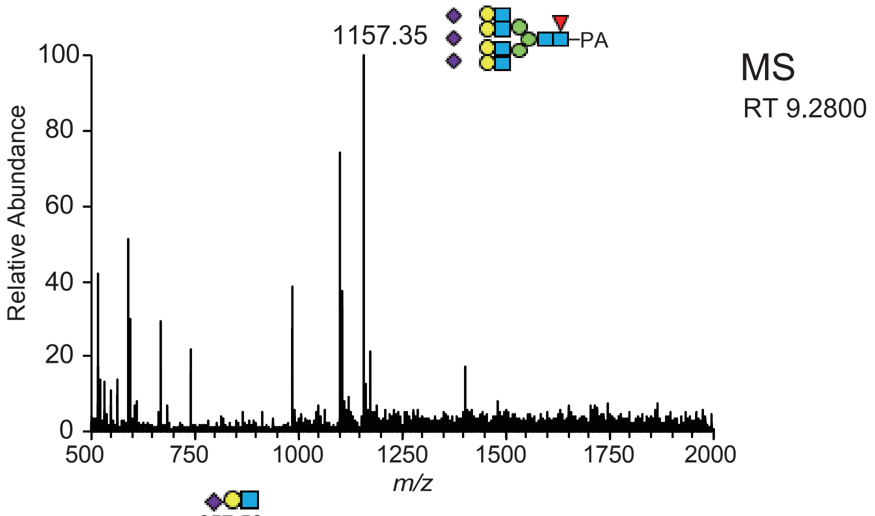

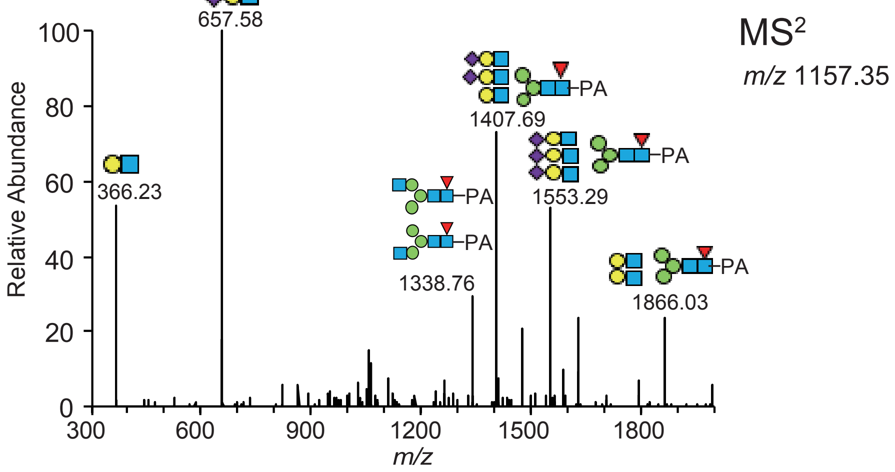

Supplementary Figure S2-44

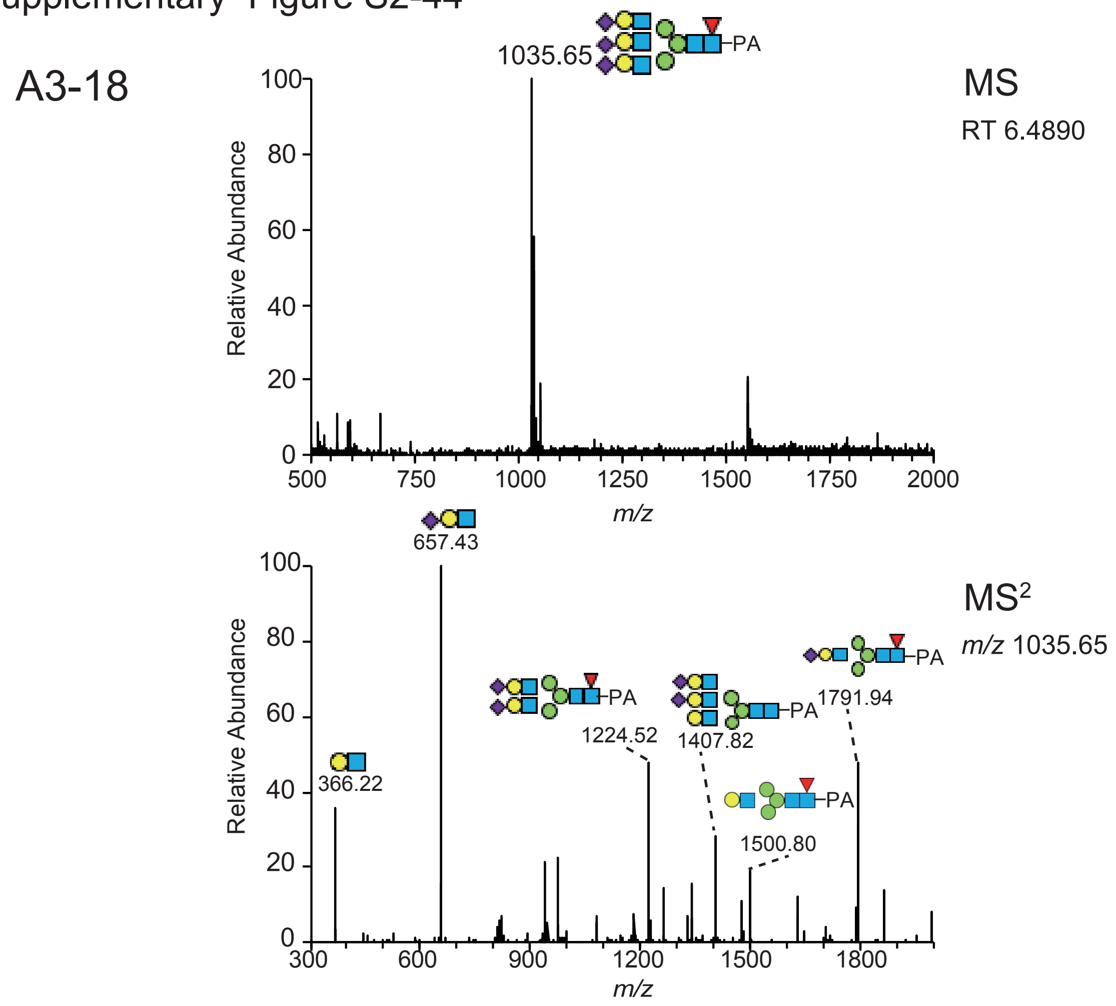

Supplementary Figure S2-45

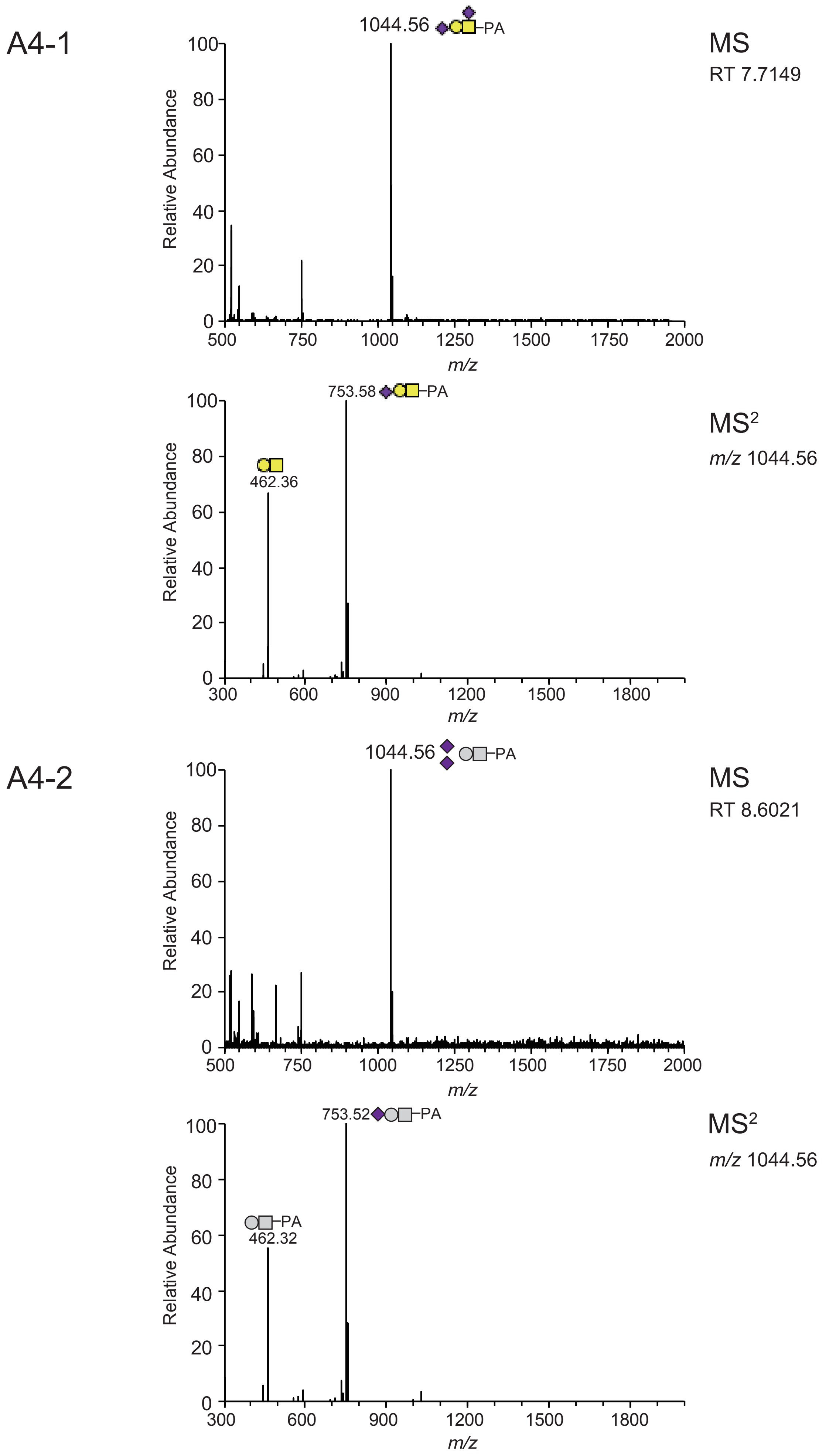

Supplementary Figure S2-46

A4-6

MS

RT 13.0450

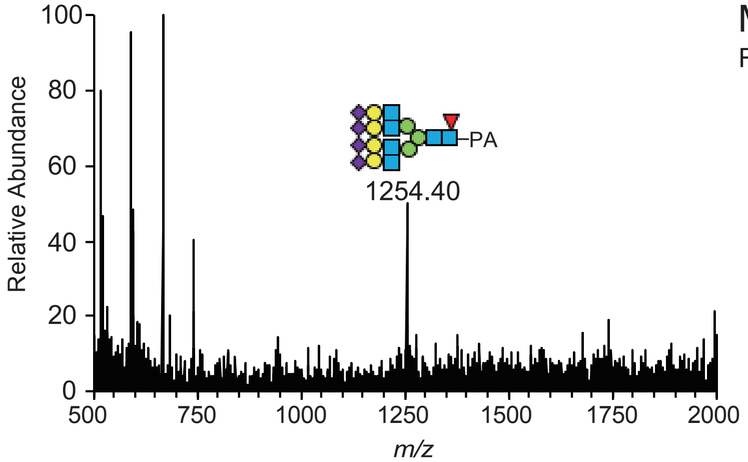

MS²

m/z 1254.40

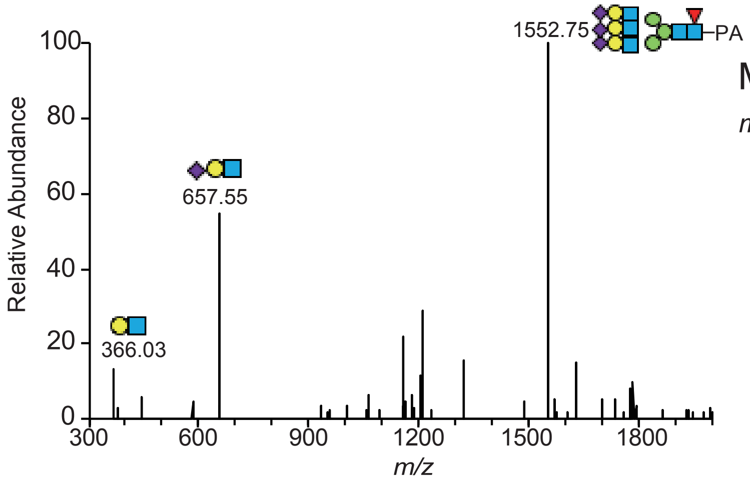

A4-7

MS

RT 13.0750

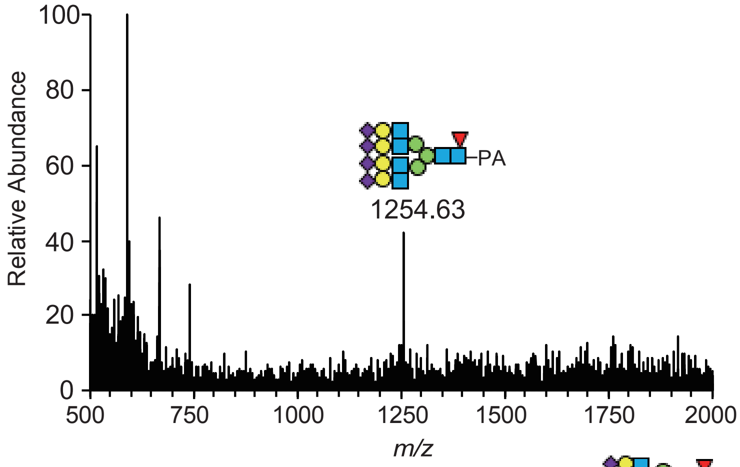

MS²

m/z 1254.63

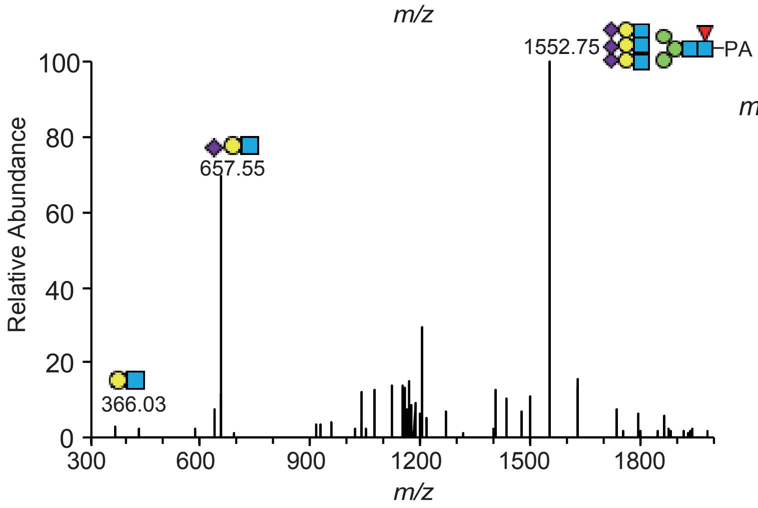

Supplementary Figure S3-1

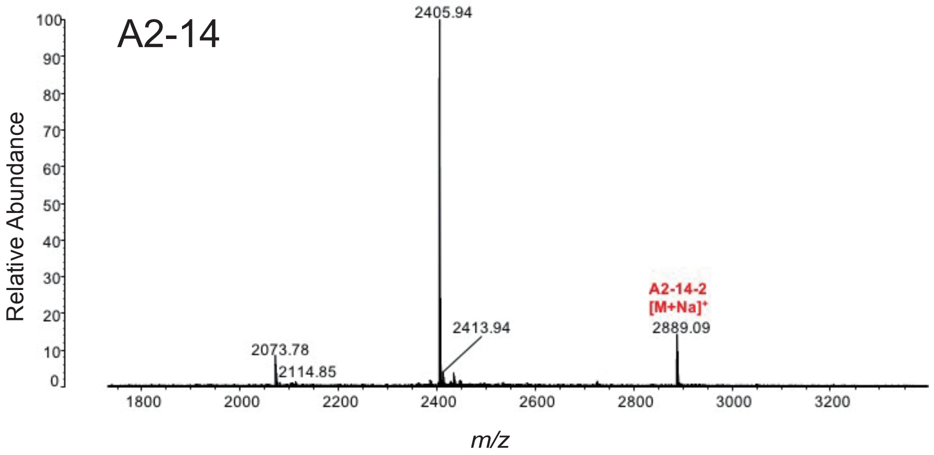

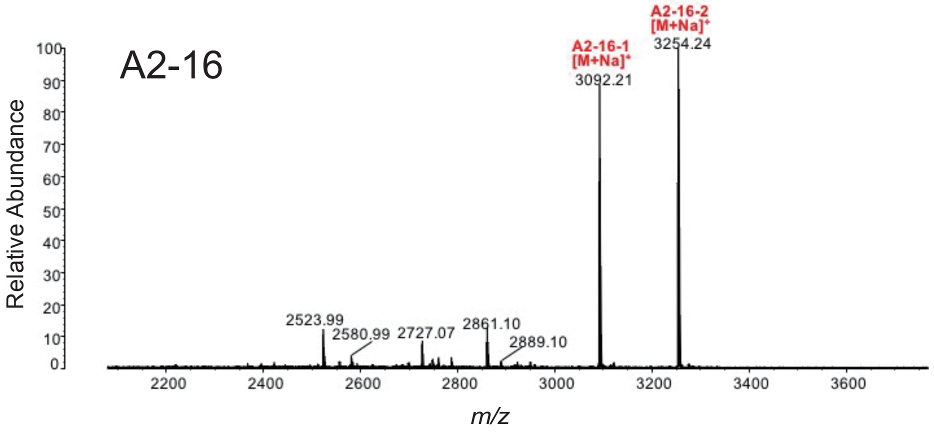

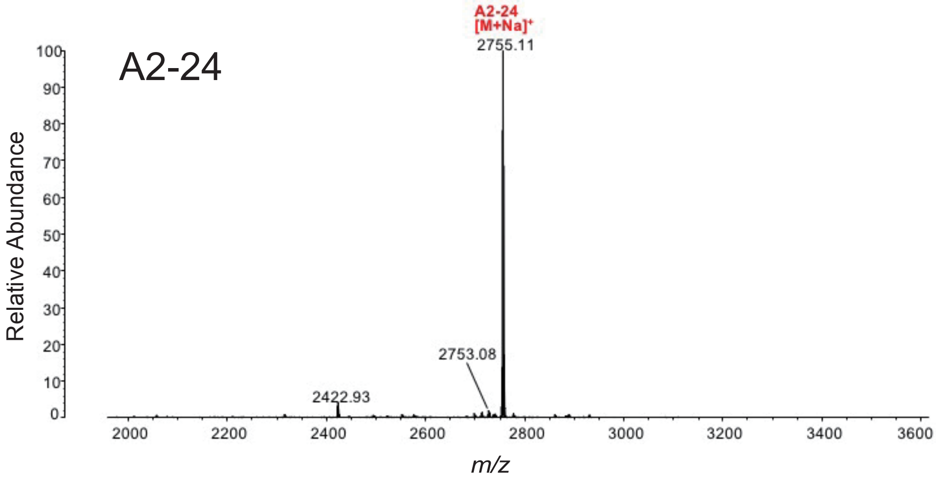

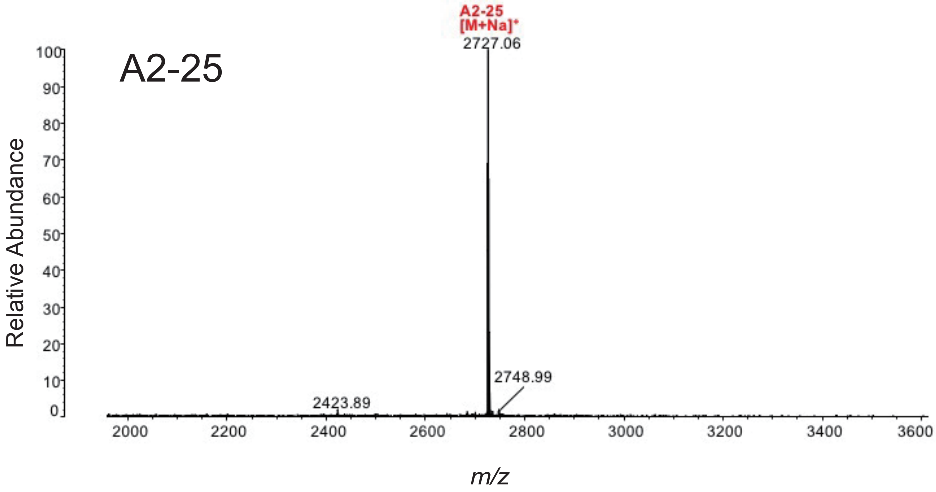

Supplementary Figure S3-2

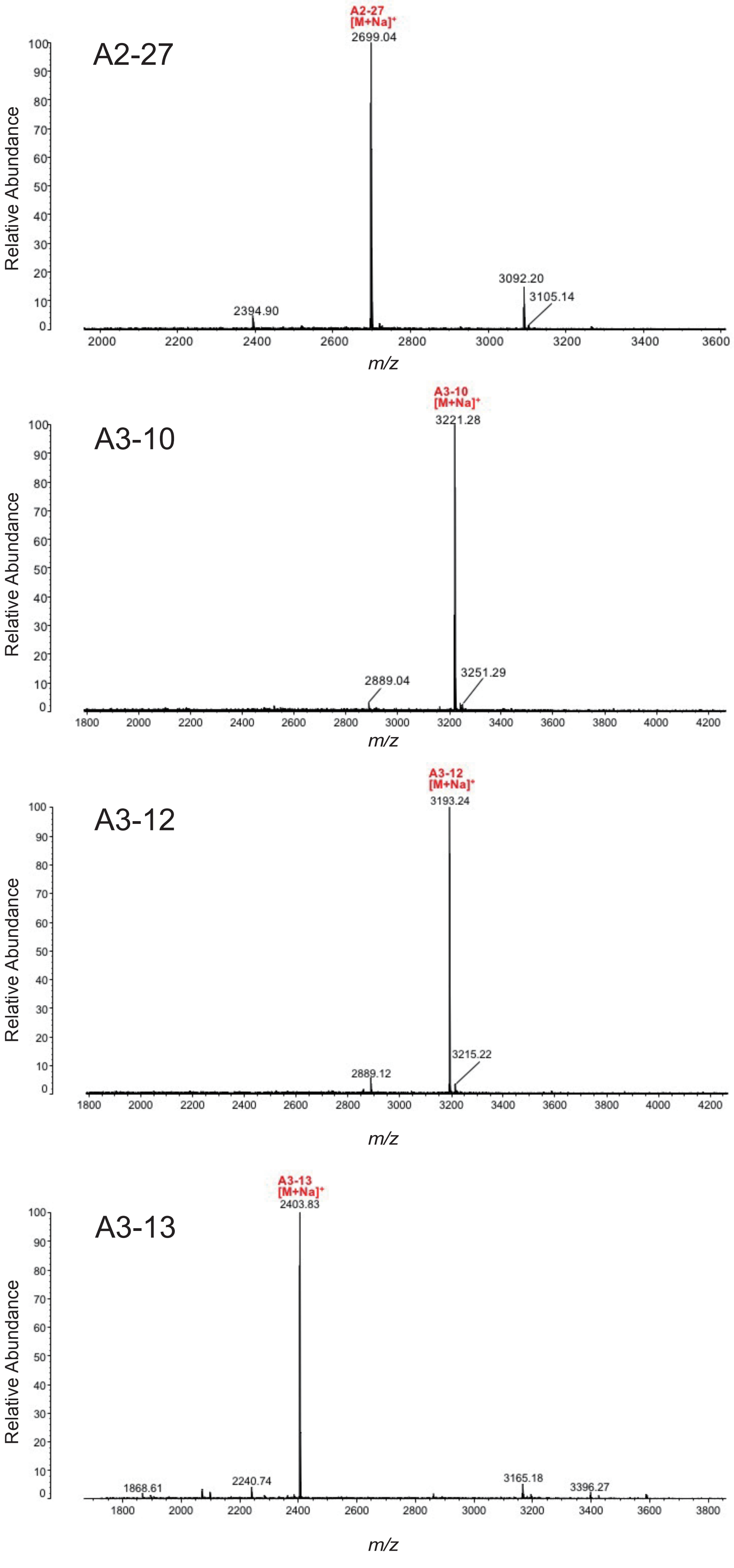

Supplementary Figure S3-3

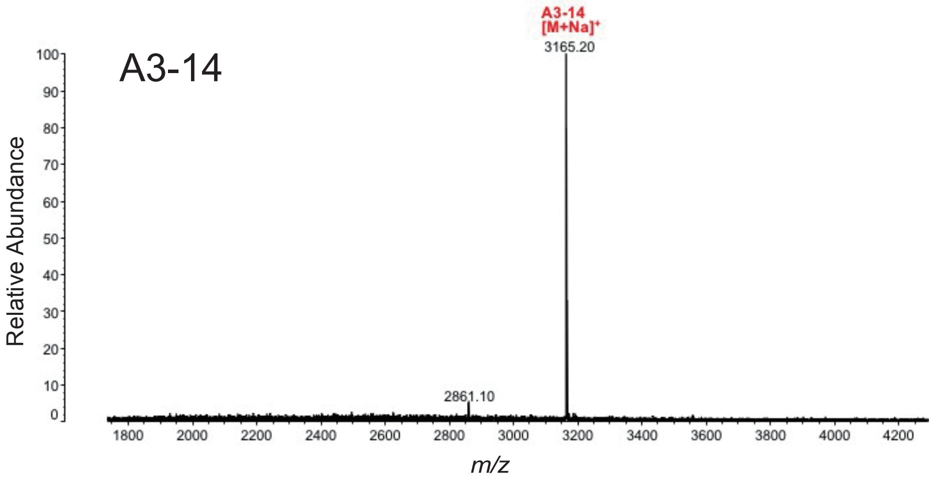

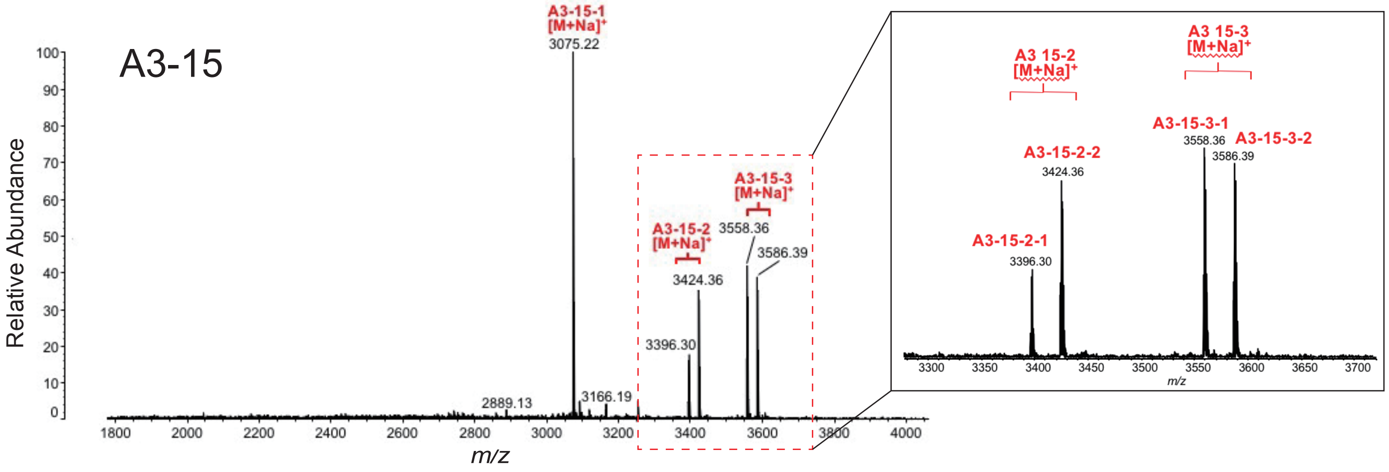

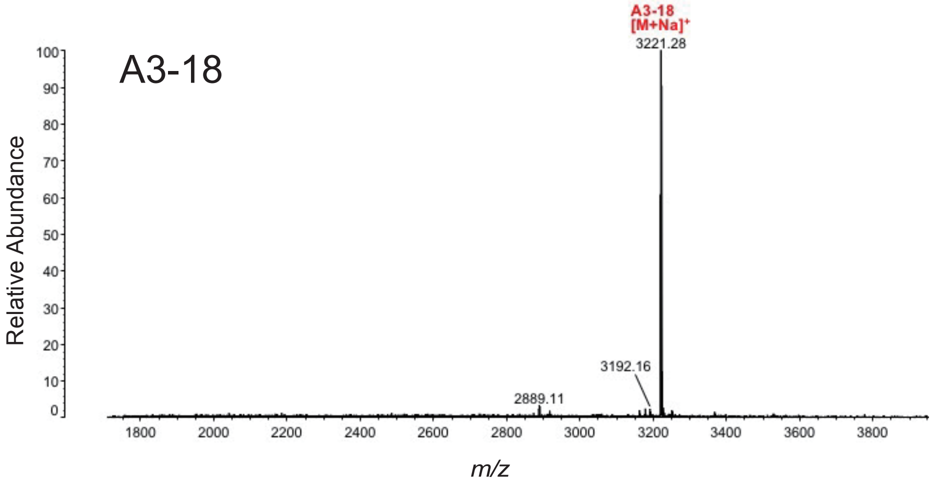

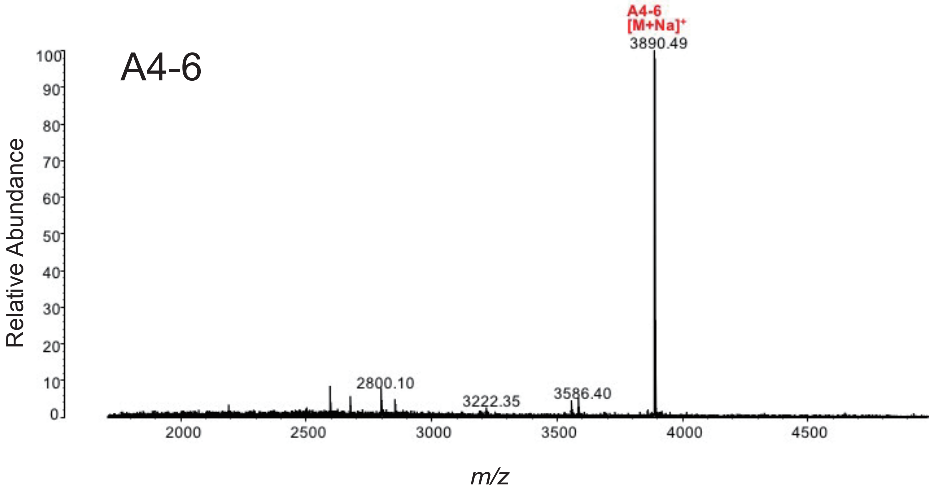

Supplementary Figure S3-4

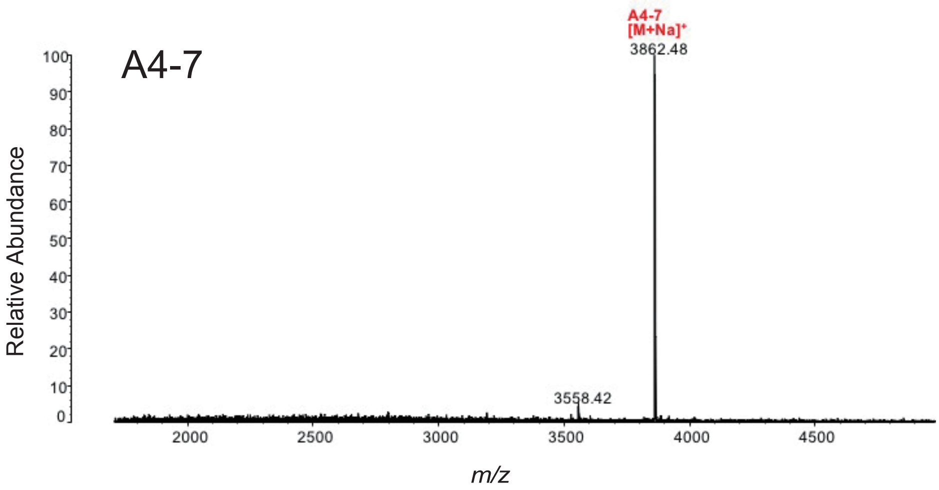

Supplementary Figure S4

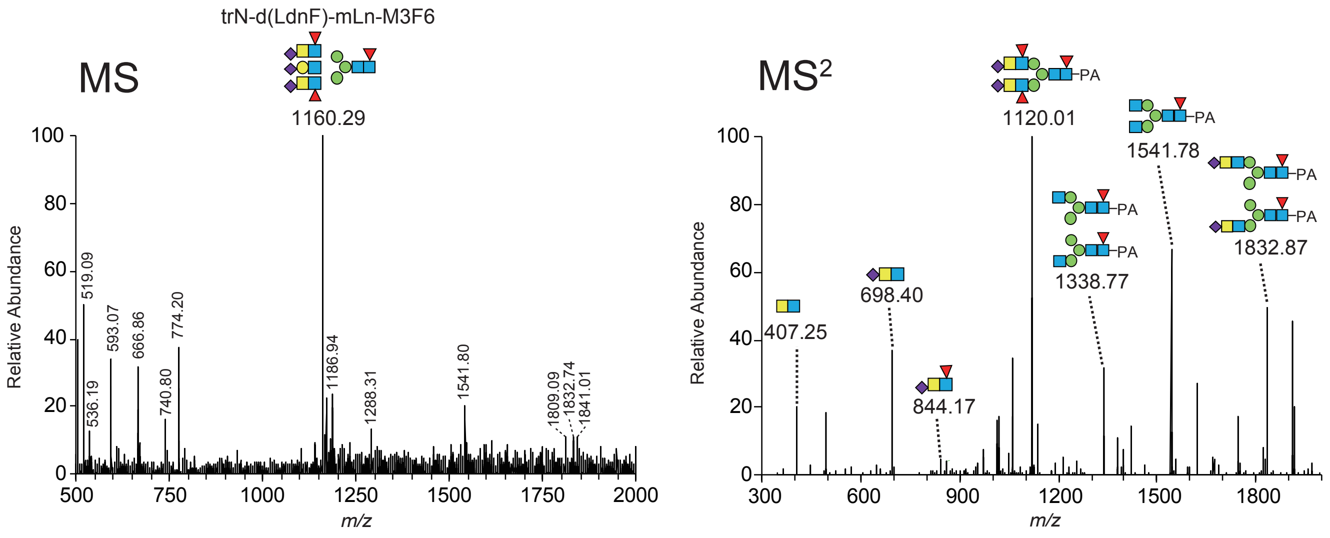

Supplementary Table S1

Mannose (Man)　N-acetylglucosamine (GlcNAc)　Galactose (Gal)　N-acetylgalactosamine (GalNAc)

Glucose (Glu)　fucose

N-Acetylneuraminic acid（NeuAc）　β　β1,4-Galactose　3　α2,3-linked NeuAc　6　α2,6-linked NeuAc

Core structure of N-glycan

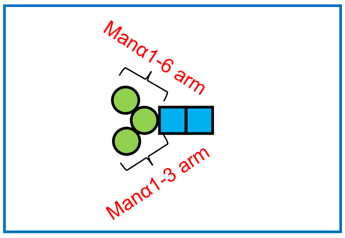

<High-mannose type>

・MX: M indicates mannose.

X shows the number of mannose residues attached to the core GlcNAcs.

・GX: G indicates glucose attached to the mannose residue at the non-reducing end.

X shows the number of glucose residues.

e.g.

G1M9

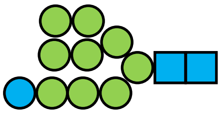

<Hybrid type>

<u>A) The number of mannose residues exceeds three.</u>

・LnMX: Ln indicates LacNAc (GalGlcNAc) in the Manα1-3 arm of the core structure.

X shows the number of mannose residues (x > 3).

e.g.

LnM5

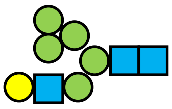

・GnMX: Gn indicates GlcNAc in the Manα1-3 arm of the core structure.

X shows the number of mannose residues (x > 3).

e.g.

GnM5

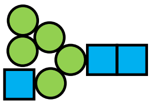

B) The number of mannose residues is three.

- MO: Mono-antennary type.

  MO1: A galactose and GlcNAc residue attached to the Manα1-3 arm of the core structure.

  MO2: A galactose and GlcNAc residue attached to the Manα1-6 arm of the core structure.

Case 1)

MO1

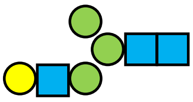

Case 2)

MO2

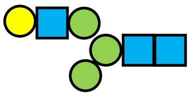

- AG: Mono-antennary-agalacto type.

  AG1: A galactose residue attached to the Manα1-3 arm of the core structure.

  AG2: A galactose residue attached to the Manα1-6 arm of the core structure.

Case 1)

AG1

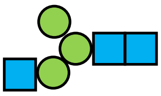

Case 2)

AG2

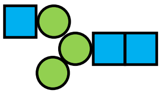

C) Sialyl linkage patterns.

- 3N: An α2,3-linked sialic acid attached to the non-reducing end of the antenna.
- 6N: An α2,6-linked sialic acid attached to the non-reducing end of the antenna.

e.g.

3N-LnM5

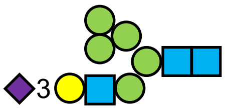

6N-LnM5

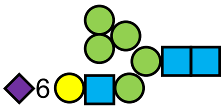

<Complex type>

A) Basic structure.

- BI: Bi-antennary-complex type.

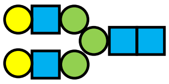

- TR: Tri-antennary-complex type.

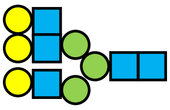

- TE: Tetra-antennary-complex type.

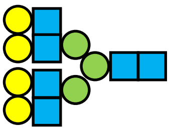

・AG12: Bi-antennary-agalacto-complex type.

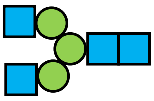

B) Sialyl linkage patterns.

・mN : Mono-sialic type.

03N: An α2,3-linked sialic acid on the Manα1-3 antenna.

30N: An α2,3-linked sialic acid on the Manα1-6 antenna.

06N: An α2,6-linked sialic acid on the Manα1-3 antenna.

60N: An α2,6-linked sialic acid on the Manα1-6 antenna.

e.g.

03N-BI 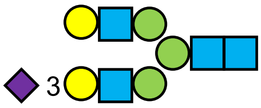 30N-BI 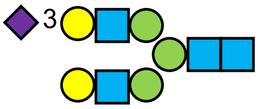

06N-BI 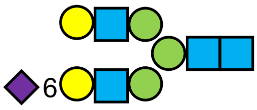 60N-BI 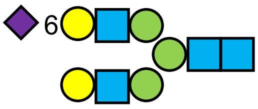

・dN: Di-sialic type.

33N: Two α2,3-linked sialic acid residues are attached; one is on the Manα1-3 antenna and the other is on the Manα1-6 antenna.

36N: An α2,3-linked sialic acid attached to the Manα1-6 antenna and an α2,6-linked sialic acid attached to the Manα1-3 antenna.

63N: An α2,6-linked sialic acid attached to the Manα1-6 antenna and an α2,3-linked sialic acid attached to the Manα1-3 antenna.

66N: Two α2,6-linked sialic acid residues are attached; one is on the Manα1-3 antenna and the other is on the Manα1-6 antenna.

e.g.

33N-BI

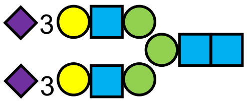

36N-BI

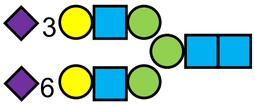

63N-BI

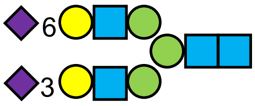

66N-BI

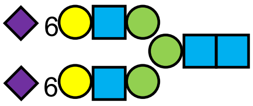

・trN: Tri-sialic type.

・teN: Tetra-sialic type.

C) Other variations

・-G: Lack of a galactose in BI.

-G1: Lack of a galactose in the Manα1-3 antenna.

-G2: Lack of a galactose in the Manα1-6 antenna.

Case 1

BI-G1

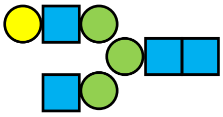

Case 2

BI-G2

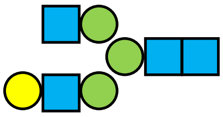

・-XG: -G indicates a lack of one or multiple galactose residues in TR or TE.

X shows the number of lacking galactose residues.

e.g.

TE-1G

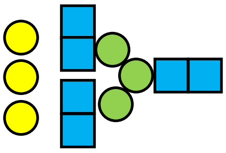

TE-2G

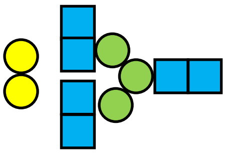

TE-3G

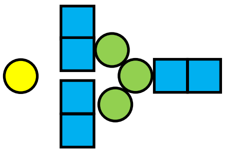

<Modifications>

・F6: An α1,6-linked fucose (core fucose).

e.g.

AG12F6

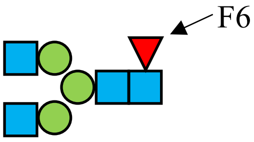

・Bs: A bisecting GlcNAc, which is a β1,4-linked GlcNAc residue attached to a β-mannose of the core structure.

e.g.

AG2Bs

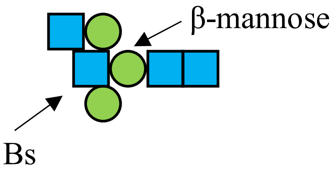

- Ldn: LacdiNAc (GalNACGlcNAc).
  - mLdn: A LacdiNAc attached to the core structure.

    mLdn1: A LacdiNAc attached to the Manα1-3 arm of the core structure.

    mLdn2: A LacdiNAc attached to the Manα1-6 arm of the core structure.

Case 1

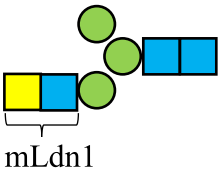

Case 2

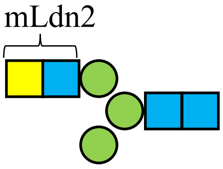

  - dLdn1,2: Two LacdiNAcs attached to both Manα1-3 and Manα1-6 arms in the core structure.

dLdn1,2

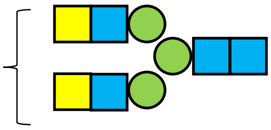

- Gaβ$_4$2: It indicates that the galactose residue at the non-reducing end was bound to the other galactose through a β1,4-linkage.

Gaβ$_4$2-BIF6

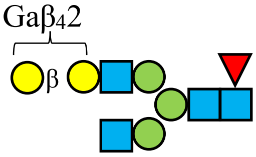

- Ac: Acetyl group attached to a sugar residue.
- SO_3^-: Sulphate group attached to a sugar residue.

Supplementary Table S2

Fraction No.		R_{stria}	S_{stria}	R_{std} or R_{calc}	S_{std} or S_{calc}	HPLC peak area	MALDI-MS total peak intensity	Relative amount	observed m/z	calculated m/z	Ion species	Assigned structure	
N	1	31.757	8.37	32.7	8.4	1518199		27.1	900.20	900.33	M+2H	$Man_8GlcNAc_2$: (M8)	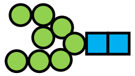
	2	33.304	7.51	34.4	7.5	715540		12.8	819.08	819.31	M+2H	$Man_7GlcNAc_2$: (M7)	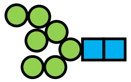
	3	35.137	9.05	36.4	9.1	1805278		32.3	981.39	981.36	M+2H	$Man_9GlcNAc_2$: (M9)	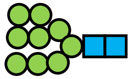
	4-1	38.140	7.46	39.4	7.5	580159		10.4	819.22	819.31	M+2H	$Man_7GlcNAc_2$: (M7)	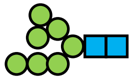
	4-2	38.140	8.21	38.7	8.2	165907		3.0	900.09	900.33	M+2H	$Man_8GlcNAc_2$: (M8)	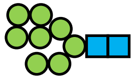
	5	40.152	6.64	41.2	6.6	5597338		100.0	738.40	738.28	M+2H	$Man_6GlcNAc_2$: (M6)	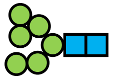
	6-1	42.858	3.15	43.1	3.2	429883		7.7	827.53	827.34	M+H	$Man_2GlcNAc_2$: (M2)	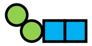
	6-2	42.858	6.02	44.5	6.1	222692		4.0	758.76	758.79	M+2H	$GlcNAcMan_5GlcNAc_2$: (GnM5)	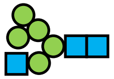
	7-1	43.755	4.11	44.5	4.1	503969		9.0	989.57	989.39	M+H	$Man_3GlcNAc_2$: (M3)	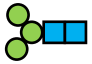
	7-2	43.755	5.01	43.7	5.0	288810		5.2	1151.67	1151.45	M+H	$Man_4GlcNAc_2$: (M4)	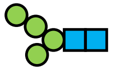

8-1	45.266	5.80	46.2	5.8	3763899	67.2	1313.87	1313.50	M+H	Man$_5$GlcNAc$_2$: (M5)	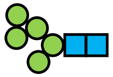
8-2	45.266	7.30	47.0	7.3	237077	4.2	819.14	819.31	M+2H	Man$_7$GlcNAc$_2$: (M7)	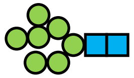
8-3	45.266	9.67	47.0	9.8	162438	2.9	1062.27	1062.39	M+2H	GluMan$_9$GlcNAc$_2$: (G1M9)	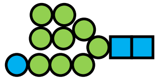
10	49.780	6.48			109528	2.0	1002.56	1002.34	M+2H	Ac-Man$_9$GlcNAc$_2$: (Ac-M9)	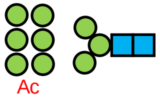
11-1	51.191	4.87	52.3	4.9	133395	2.4	698.28	698.28	M+2H	GlcNAc$_2$Man$_3$GlcNAc$_2$: (AG12)	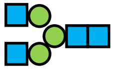
12-1	52.739	3.55	51.5	3.4	450004	8.0	973.61	973.40	M+H	Man$_2$FucGlcNAc$_2$: (M2F6)	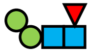
12-2	53.507	4.45	54.0	4.4	559826	10.0	1135.67	1135.45	M+H	Man$_3$FucGlcNAc$_2$: (M3F6)	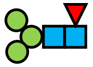
12-3	53.507	5.61	53.7	5.6	642147	11.5	779.21	779.31	M+2H	GlcNAc$_2$Man$_4$GlcNAc$_2$: (GnM4Bs)	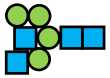
14-2	55.595	6.31	56.3	6.4	433487	7.7	860.35	860.33	M+2H	Gal$_2$GlcNAc$_2$Man$_3$GlcNAc$_2$: (BI)	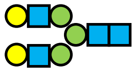
15	56.807	4.89			205594	3.7	758.89	759.27	M+2H	Ac-Man$_6$GlcNAc$_2$: (Ac-M6)	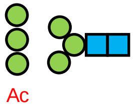
16-1	58.699	5.62			117737	2.1	1354.66	1355.47	M+H	Ac-Man$_5$GlcNAc$_2$: (Ac-M5)	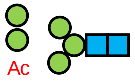

	16-2	58.699	6.48			110575	2.0	982.30	982.39	M+2H	GalGlcNAcHexNAc$_3$Man$_3$GlcNAc$_2$: (Hex$_4$HexNAc$_6$)	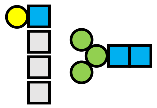
	17	59.934	5.12	60.3	5.1	120906	2.2	771.36	771.31	M+2H	GlcNAc$_2$Man$_3$FucGlcNAc$_2$: (AG12F6)	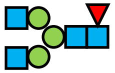
	20	64.501	5.17	64.7	5.2	215886	3.9	771.25	771.31	M+2H	GlcNAc$_2$Man$_3$FucGlcNAc$_2$: (AG2BsF6)	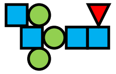
	21-1	66.000	5.16	66.6	5.2	998602	17.8	799.73	799.82	M+2H	GlcNAc$_3$Man$_3$GlcNAc$_2$: (AG12Bs)	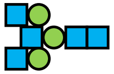
	21-2	66.000	7.39			126497	2.3	1136.85	1136.44	M+2H	Gal$_2$GlcNAc$_2$HexNAc$_2$Man$_3$FucGlcNAc$_2$: (Hex$_5$HexNAc$_6$dHex)	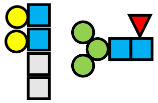
	22-1	68.153	5.80	70.2	5.8	310986	5.6	880.84	880.85	M+2H	GalGlcNAc$_3$Man$_3$GlcNAc$_2$: (BIBs-G2)	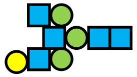
	22-2	68.153	6.69			191309	3.4	1055.43	1055.41	M+2H	GalGlcNAc$_4$Man$_3$FucGlcNAc$_2$: (TEF6-3G)	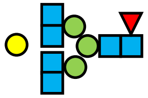
	25	76.047	5.38	76.1	5.4	715106	12.8	872.97	872.85	M+2H	GlcNAc$_3$Man$_3$FucGlcNAc$_2$: (AG12BsF6)	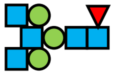
	26	78.263	5.97	78.4	6.0	385168	6.9	953.97	953.87	M+2H	GalGlcNAc$_3$Man$_3$FucGlcNAc$_2$: (BIBsF6-G1)	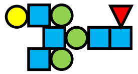
	27	79.883	6.65	80.0	6.7	143641	2.6	1034.87	1034.90	M+2H	Gal$_2$GlcNAc$_3$Man$_3$FucGlcNAc$_2$: (BIBsF6)	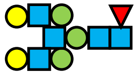
A1	2	53.324	5.83	55.3	6.0	365229	6.5	823.53	823.31	M+2H	NeuAcGalGlcNAcMan$_3$GlcNAc$_2$: (6N-MO1)	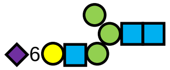

3-1	54.312	6.47	56.2	6.6	1505040	26.9	904.54	904.32	M+2H	NeuAcGalGlcNAcMan$_4$GlcNAc$_2$: (6N-LnM4)	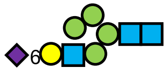
3-2	54.312	7.24	56.6	7.4	3121982	55.8	985.59	985.37	M+2H	NeuAcGalGlcNAcMan$_5$GlcNAc$_2$: (6N-LnM5)	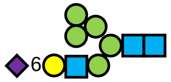
4-1	58.874	6.03	61.2	5.9	170612	3.0	904.26	904.32	M+2H	NeuAcGalGlcNAcMan$_4$GlcNAc$_2$: (3N-LnM4)	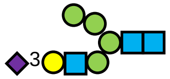
4-2	58.874	6.74	60.7	6.9	501075	9.0	985.40	985.37	M+2H	NeuAcGalGlcNAcMan$_5$GlcNAc$_2$: (3N-LnM5)	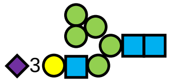
5	59.998	6.05	61.7	6.1	229914	4.1	924.88	924.85	M+2H	NeuAcGalGlcNAc$_2$Man$_3$GlcNAc$_2$: (06N-BI-G2)	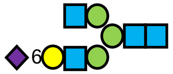
6	61.064	8.07	62.6	8.4	133380	2.4	1261.94	1261.48	M+2H	NeuAcGal$_3$GlcNAc$_3$Man$_3$FucGlcNAc$_2$: (06N-TRF6)	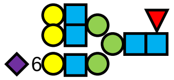
8-1	62.732	6.06	62.9	6.1	256436	4.6	896.39	896.34	M+2H	NeuAcGalGlcNAcMan$_3$FucGlcNAc$_2$: (6N-MO1F6)	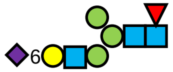
8-2	62.732	6.71	64.9	6.5	554308	9.9	977.46	977.37	M+2H	NeuAcGalGlcNAcMan$_4$FucGlcNAc$_2$: (6N-LnM4F6)	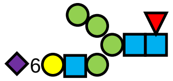
8-3	62.732	7.42	64.4	7.5	175219	3.1	1058.33	1058.40	M+2H	NeuAcGalGlcNAcMan$_5$FucGlcNAc$_2$: (6N-LnM5F6)	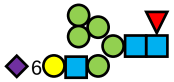
9	64.148	5.96	67.2	5.7	156596	2.8	924.80	924.85	M+2H	NeuAcGalGlcNAc$_2$Man$_3$GlcNAc$_2$: (03N-BI-G2)	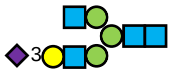
11	67.625	5.89	69.4	5.9	137670	2.5	1018.39	1018.40	M+2H	NeuAcGalNAcGlcNAc$_2$Man$_3$FucGlcNAc$_2$:[06N-BI(mLdn1)F6-G2]	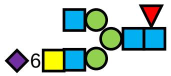
12	68.806	6.28	71.0	6.4	329743	5.9	997.96	997.88	M+2H	NeuAcGalGlcNAc$_2$Man$_3$FucGlcNAc$_2$: (06N-BIF6-G2)	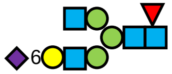

	13	70.422	6.93	72.5	7.1	226299		4.0	1078.94	1078.91	M+2H	NeuAcGal2GlcNAc2Man3FucGlcNAc2: (06N-BIF6)	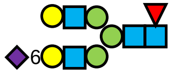
	14-1	71.278	6.30	74.4	6.4	123392		2.2	1026.43	1026.39	M+2H	NeuAcGalGlcNAc3Man3GlcNAc2: (06N-BIBs-G2)	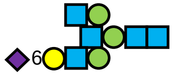
	14-2	71.278	7.61	72.2	7.6	152612		2.7	1160.38	1159.94	M+2H	NeuAcGal3GlcNAc2Man3FucGlcNAc2: (06N-Gaβ4 2-BIF6)	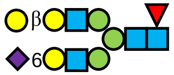
	17-1	75.313	6.02	76.6	5.9	121824		2.2	896.31	896.34	M+2H	NeuAcGalGlcNAcMan3FucGlcNAc2: (6N-MO2F6)	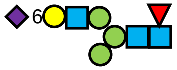
	17-2	75.313	6.49	76.4	6.6	155359		2.8	1079.28	1078.91	M+2H	NeuAcGal2GlcNAc2Man3FucGlcNAc2: (30N-BIF6)	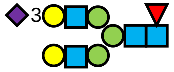
	18	76.811	5.57	77.7	5.6	159687		2.9	896.18	896.34	M+2H	NeuAcGalGlcNAcMan3FucGlcNAc2: (3N-MO2F6)	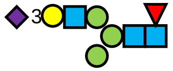
	19	79.040	6.08	78.9	6.2	192797		3.4	1026.35	1026.39	M+2H	NeuAcGalGlcNAc3Man3GlcNAc2: (60-BIBs-G1)	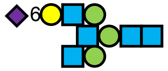
	20	81.076	6.55	82.7	6.6	211682		3.8	1099.45	1099.42	M+2H	NeuAcGalGlcNAc3Man3FucGlcNAc2: (06N-BIBsF6-G2)	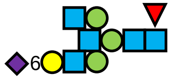
	21	84.022	7.10	86.2	7.2	187856		3.4	1180.39	1180.45	M+2H	NeuAcGal2GlcNAc3Man3FucGlcNA2: (06N-BIBsF6)	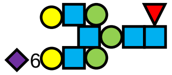
	22	86.338	6.29	89.1	6.5	331521		5.9	1099.87	1099.42	M+2H	NeuAcGalGlcNAc3Man3FucGlcNAc2: (06-BIBsF6-G2)	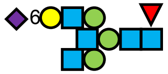
A2	14-1	71.510	7.11	75.2	7.3	1228538		21.9	1152.04	1151.43	M+2H	NeuAc2Gal2GlcNAc2Man3GlcNAc2: (66N-BI)	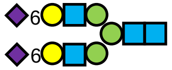
	14-2	71.510	7.83			115771		2.1	1406.91	1407.02	M+2H	NeuAc2Gal3GlcNAc3Man3FucGlcNAc2 : (dN-TRF6)	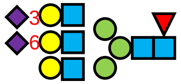

15	73.106	6.68	76.5	6.9	270034	4.8	1151.49	1151.43	M+2H	NeuAc2Gal2GlcNAc2Man3GlcNAc2 : (36N-BI)	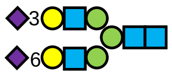
16-1	75.619	8.04			122833	2.2	1006.21	1006.04	M+3H	NeuAc2Gal3GlcNAc4Man3FucGlcNAc2 : (dN-TEF6-1G or dN-TRBsF6)	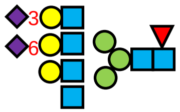
16-2	75.619	8.61			188907	3.4	1060.41	1060.06	M+3H	NeuAc2Gal4GlcNAc4Man3FucGlcNAc2: (dN-TEF6)	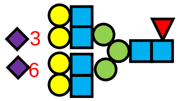
17	76.989	6.89	79.8	7.1	452182	8.1	1245.43	1244.97	M+2H	NeuAc2GalGalNAcGlcNAc2Man3FucGlcNAc2: [66N-BI(mLdn1)F6]	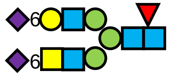
18	77.890	7.32	81.4	7.5	1075980	19.2	1225.04	1224.46	M+2H	NeuAc2Gal2GlcNAc2Man3FucGlcNAc2: (66N-BIF6)	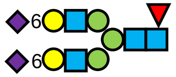
19	79.027	6.60	82.0	6.8	150910	2.7	1265.98	1265.48	M+2H	NeuAc2Gal2GlcNAc2Man3FucGlcNAc2: [66N-BI(dLdn1,2)F6]	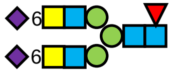
20	80.969	6.90	84.2	7.1	474863	8.5	1224.96	1224.46	M+2H	NeuAc2Gal2GlcNAc2Man3FucGlcNAc2: (36N-BIF6)	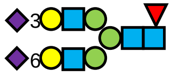
23	85.992	6.54	89.0	6.7	280050	5.0	1224.42	1224.46	M+2H	NeuAc2Gal2GlcNAc2Man3FucGlcNAc2: (33N-BIF6)	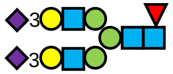
24	90.046	7.46			360900	6.4	1326.40	1326.00	M+2H	NeuAc2Gal2GlcNAc3Man3FucGlcNAc2: (dN-BIBsF6 or dN-TRF6-1G)	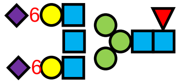
25	93.339	6.92			286508	5.1	1325.40	1326.00	M+2H	NeuAc2Gal2GlcNAc3Man3FucGlcNAc2: (dN-BIBsF6 or dN-TRF6-1G)	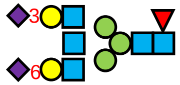
27	99.235	6.50			116455	2.1	1325.89	1326.00	M+2H	NeuAc2Gal2GlcNAc2Man3FucGlcNAc2: (dN-BIBsF6 or dN-TRF6-1G)	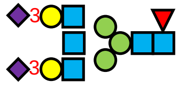

A3	10	77.082	8.11	163570		2.9	1035.56	1035.38	M+3H	$NeuAc_3Gal_3GlcNAc_3Man_3FucGlcNAc_2$: (trN-TRF6)	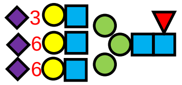
	12	79.491	7.81	293157		5.2	1035.37	1035.38	M+3H	$NeuAc_3Gal_3GlcNAc_3Man_3FucGlcNAc_2$: (trN-TRF6)	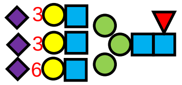
	13	81.419	5.59	155074		2.8	1160.29	1160.11	M+2H	SO_3^--$NeuAcGalNAc_2GlcNAc_2Man_3FucGlcNAc_2$: [$SO_3^-$-6N-BI(dLdn1,2)F6]	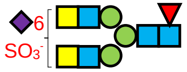
	14	82.500	7.40	163492		2.9	1035.83	1035.38	M+3H	$NeuAc_3Gal_3GlcNAc_3Man_3FucGlcNAc_2$: (trN-TRF6)	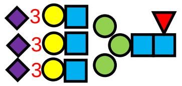
	15-1	83.595	7.93	173971		3.1	986.90	986.70	M+3H	$NeuAc_3Gal_3GlcNAc_3Man_3GlcNAc_2$: (trN-TR)	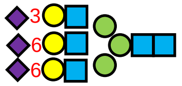
	15-2-1	83.595	8.52	220989	385536	1.2	1103.34	1103.08	M+3H	$NeuAc_3Gal_3GlcNAc_4Man_3FucGlcNAc_2$: (trN-TRBsF6 or trN-TEF6-1G)	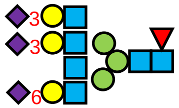
	15-2-2	83.595	8.52	220989	874880	2.7	1103.34	1103.08	M+3H	$NeuAc_3Gal_3GlcNAc_4Man_3FucGlcNAc_2$: (trN-TRBsF6 or trN-TEF6-1G)	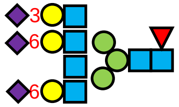
	15-3-1	83.595	8.95	175659	996224	1.5	1157.35	1157.09	M+3H	$NeuAc_3Gal_4GlcNAc_4Man_3FucGlcNAc_2$: (trN-TEF6)	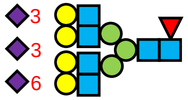
	15-3-2	83.595	8.95	175659	1031808	1.6	1157.35	1157.09	M+3H	$NeuAc_3Gal_4GlcNAc_4Man_3FucGlcNA_2$: (trN-TEF6)	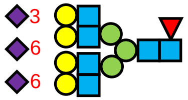
	18	90.797	8.05	424247		7.6	1035.65	1035.38	M+3H	$NeuAc_3Gal_3GlcNAc_3Man_3FucGlcNAc_2$: (trN-TRF6)	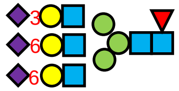

A4	6	88.863	8.88	118264	2.1	1254.40	1254.13	M+3H	$NeuAc_4Gal_4GlcNAc_4Man_3FucGlcNAc_2$: (teN-TEF6)	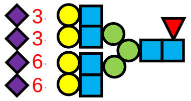
	7	90.864	8.54	122688	2.2	1254.63	1254.13	M+3H	$NeuAc_4Gal_4GlcNAc_4Man_3FucGlcNAc_2$: (teN-TEF6)	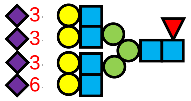

Mannose (Man) N-acetylglucosamine (GlcNAc) Galactose (Gal) N-acetylgalactosamine (GalNAc)

N-acetylhexosamine (HexNAc: GalNAc or GlcNAc) Fucose (Fuc) Glucose (Glu) N-Acetylneuraminic acid（NeuAc）

β β1,4-Galactose 3 α2,3-linked NeuAc 6 α2,6-linked NeuAc

3 α2,3-linked NeuAc identified by SALSA 6 α2,6-linked NeuAc identified by SALSA

Ac: Acetyl group SO_3^-: Sulphate group

Supplementary Table S3

Fraction No.		R_{stria}	S_{stria}	Relative amount	calculated m/z	observed m/z	ion species	Assigned structure	
N	11-2	51.191	6.49	7.0				n.d.	
	14-1	55.595	5.71	3.9				n.d.	
A2	1	23.466	2.72	12.3	753.27	753.45	M+H	NeuAcGalGalNAc: (60N-core1)	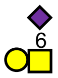
	2-1	28.276	2.25	95.6	550.22	550.50	M+H	NeuAcGal: (mN-Gal)	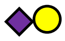
	2-2	28.276	2.97	4.0	753.27	753.37	M+H	NeuAcHexHexNAc: (mN-H1HN1)	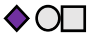
	3	29.508	2.52	11.2	753.27	753.44	M+H	NeuAcGalGalNAc: (03N-core1) NeuAcGalNAc:	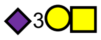
	4	31.007	2.21	13.8	591.21	590.27	M+H	(mN-GalNAc) NeuAcHexHexNAc:	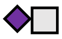
	5	34.410	2.53	3.0	753.27	753.40	M+H	(mN-H1HN1) $NeuAc_2Gal_2GalNAcGlcNAc$	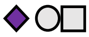
A3	3	49.561	4.22	3.4	705.27	705.12	M+H	: (33N-$G\beta_4$core2) $NeuAc_2GalGalNAc$:	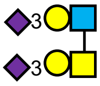
A4	1	37.970	3.46	26.1	1044.4	1044.56	M+H	(36N-core1) $NeuAc_2HexHexNAc$	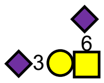
	2	49.615	3.43	3.6	1044.4	1044.56	M+H	(dN-H1HN1)	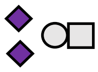

N-acetylglucosamine (GlcNAc)   Galactose (Gal)   N-acetylgalactosamine (GalNAc)

N-acetylhexosamine (HexNAc: GalNAc or GlcNAc)   Hexose (Hex: Man or Gal)

N-Acetylneuraminic acid（NeuAc）   3 α2,3-linked NeuAc   6 α2,6-linked NeuAc

Supplementary Table S4

Database of standard glycans

・**Monosaccharide symbols**

○Galactose, ◯ Mannose, ●Glucose, □ N-acetylgalactosamine, ■N-acetylglucosamine,
△ Fucose, ☆ Xylose, ◇N-Acetylneuraminic acid, ◆Glucuronic acid, PA: pyridylamine

・**References (Ref)**

1) Shunji Natsuka, Mayumi Masuda, Wataru Sumiyoshi, Shin-ichi Nakakita.
Improved method for drawing of a glycan map, and the firstpage of Glycan Atlas, which is a compilation of glycan maps for a whole organism.
PlosOne, **9** (7) e102219 (2014).

2) Ken Hanzawa, Noriko Suzuki, Shunji Natsuka.
Structures and developmental alterations of *N*-glycans of zebrafish embryos.
Glycobiology, **27** (3) 228-245 (2017).

	Glycan structure	S_{std}	R_{std}	Ref
【High mannose type】				
M5	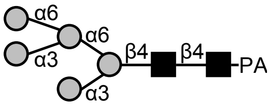	5.8	46.2	1
M5	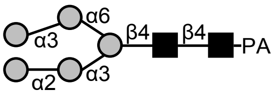	5.7	40.4	1
M6	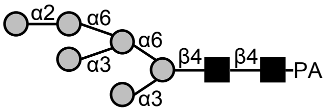	6.7	38.8	1
M6	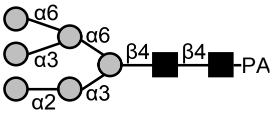	6.6	41.2	1
M6	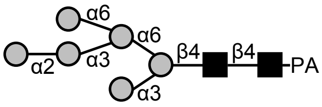	6.5	51.0	1
M6	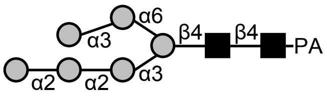	6.5	38.6	1

M7	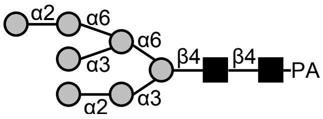	7.5	34.4	1
M7	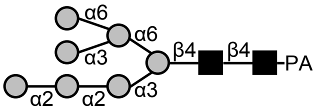	7.5	39.4	1
M7	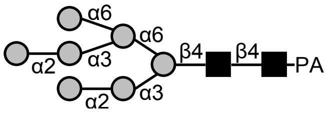	7.3	47.0	1
M8	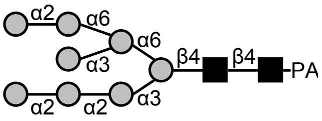	8.4	32.7	1
M8	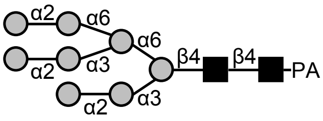	8.2	38.7	1
M8	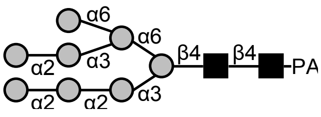	8.2	44.9	1
M9	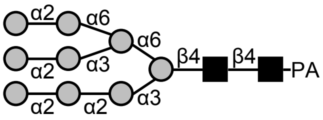	9.1	36.4	1
G1M4E	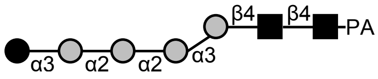	5.5	39.6	1
G1M5	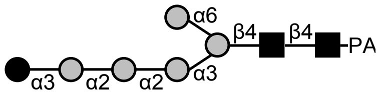	6.4	47.2	1
G1M7	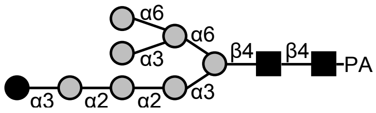	8.2	49.4	1
G1M8	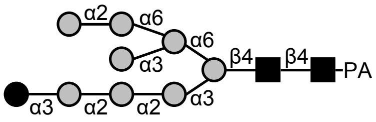	9.0	43.0	2

G1M8	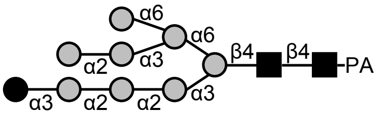	8.8	53.5	1
G1M9	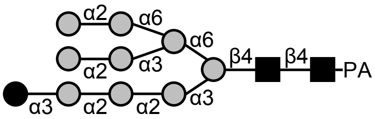	9.8	47.0	1
G2M9	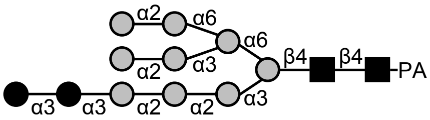	10.3	51.7	1
G3M9	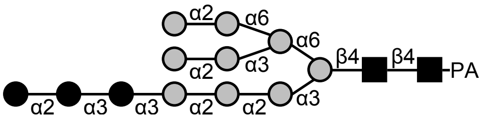	11.3	48.8	1
【Paucimannose type】				
GN	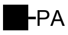	0.9	16.4	1
GNF6	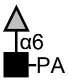	1.6	25.9	1
GN2	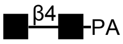	1.8	28.3	1
GN2F3	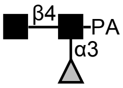	2.3	19.3	1
GN2F6	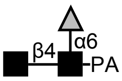	2.1	37.7	1
M1	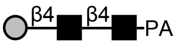	2.4	35.0	1
M1F3	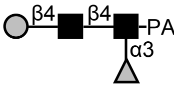	3.1	27.3	1
M1F3X	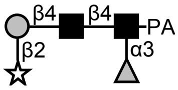	3.4	32.0	1
M2	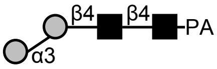	3.2	34.8	1

M2	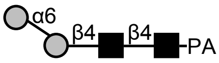	3.2	43.1	1
M2F3X	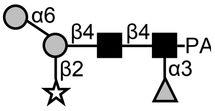	4.2	37.2	1
M2F6	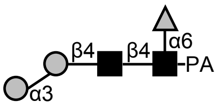	3.6	43.8	1
M3	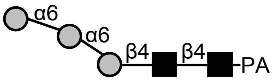	4.2	43.0	1
M3	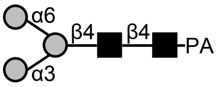	4.1	44.5	1
M3	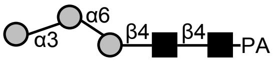	4.1	43.1	1
M3X	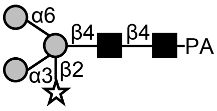	4.6	43.7	1
M3F6	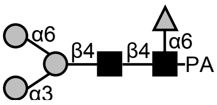	4.4	54.0	1
M3F6X	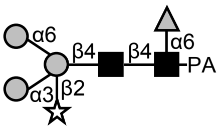	4.8	53.3	1
M3F3X	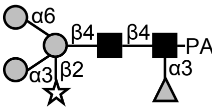	5.4	32.0	1
M4	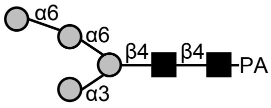	5.0	43.7	1
M4	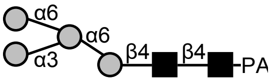	5.0	45.0	1

M4	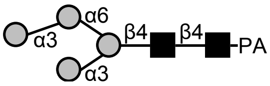	4.8	45.5	1
M4X	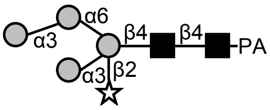	5.3	45.4	1

【Agalacto-complex type】

AG1	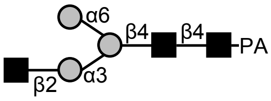	4.5	42.4	1
AG2	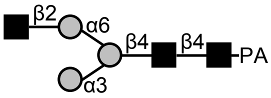	4.5	55.2	1
AG3	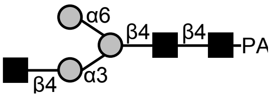	4.5	50.2	1
AG4	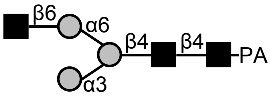	4.7	47.4	1
AG1F$_6$	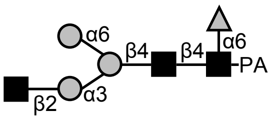	4.8	52.3	1
AG1F$_6$-M$_6$	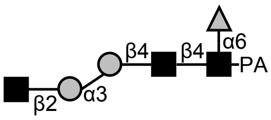	4.1	48.7	1
AG2F$_6$	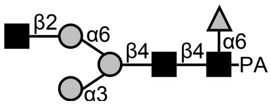	4.8	63.4	1
AG2BsF$_6$	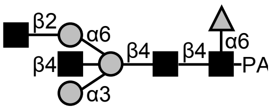	5.2	64.7	1
AG2F$_3$X	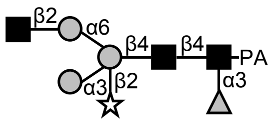	5.7	38.9	1

AG12	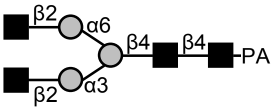	4.9	52.3	1
AG13	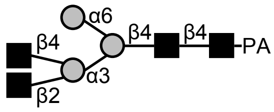	4.8	52.2	1
AG14	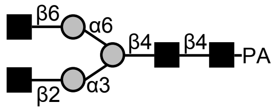	5.0	44.9	1
AG23	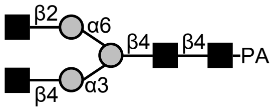	4.8	60.1	1
AG24	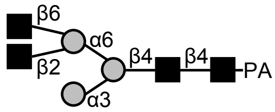	5.2	43.0	1
AG34	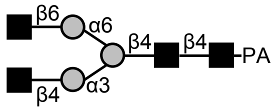	5.0	52.4	1
AG12Bs	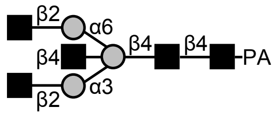	5.2	66.6	1
AG12F$_6$	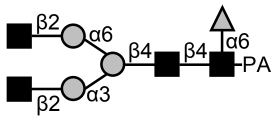	5.1	60.3	1
AG12BsF$_6$	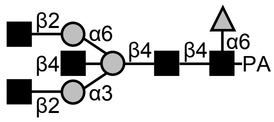	5.4	76.1	1
AG12F$_3$X	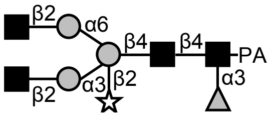	5.9	35.3	1
AG123	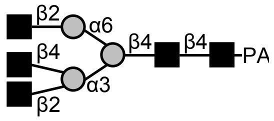	5.1	60.2	1

AG124	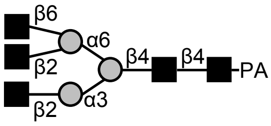	5.4	41.5	1
AG134	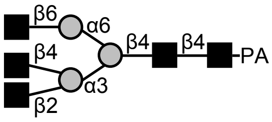	5.4	54.2	1
AG234	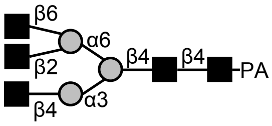	5.4	49.5	1
AG123Bs	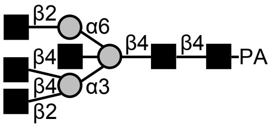	5.3	79.8	1
AG123F6	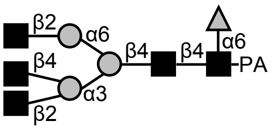	5.3	68.6	1
AG1234	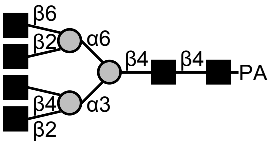	5.7	52.8	1
AG12345Bs	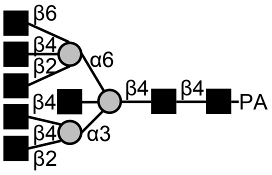	6.2	69.1	1

【Hybrid type】

GnM5	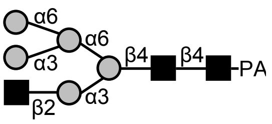	6.1	44.5	1
GnM4Bs	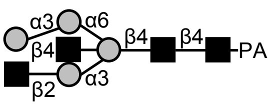	5.6	53.7	1
GnM5Bs	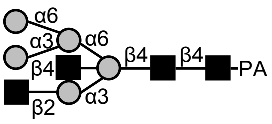	6.5	52.1	1

LnM5	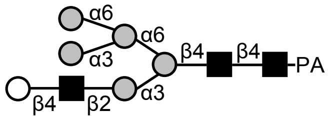	6.9	47.9	1
6N-LnM4	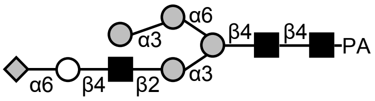	6.6	56.2	2
3N-LnM5	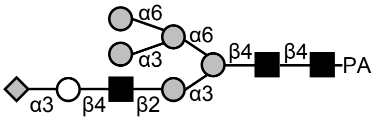	6.9	60.7	2
6N-LnM5	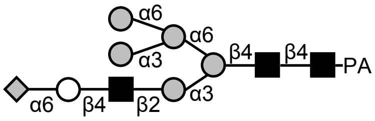	7.4	56.6	2
6N-LnM6	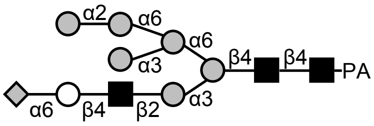	7.6	51.3	2
3N-LnM5F_6	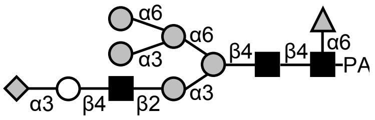	7.0	68.0	2
6N-LnM5F_6	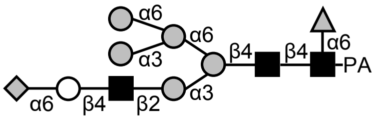	7.5	64.4	2
6N-LdnM4	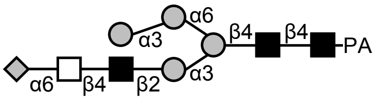	6.2	55.3	2
6N-LdnM5	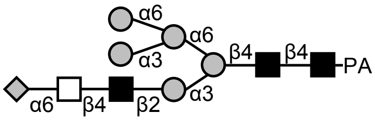	6.9	55.3	2
Gaβ_4LnM5	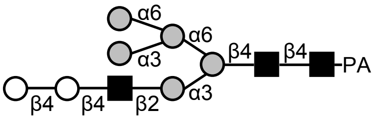	7.6	46.0	2

【Asialo-complex type】

MO1	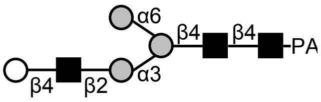	5.4	46.4	1
MO2	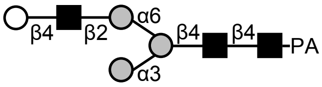	5.3	57.1	1
MO1-M$_6$	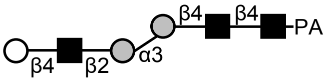	4.6	40.3	1
MO2-M$_3$	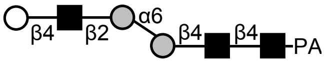	4.6	54.9	1
MO1F$_6$	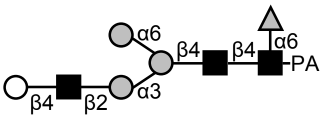	5.6	54.9	1
MO2F$_6$	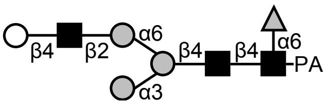	5.5	64.8	1
MO1F$_6$-M$_6$	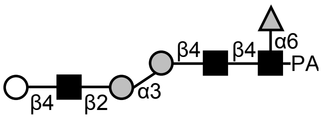	4.8	51.1	1
BI	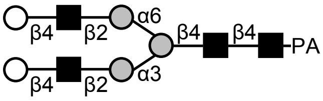	6.4	56.3	1
BI-G1	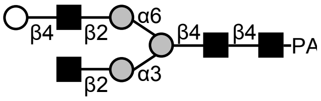	5.6	54.1	1
BI-G2	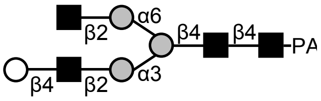	5.7	54.8	1
BIF$_6$	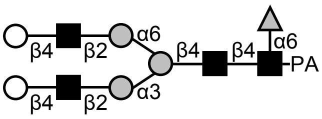	6.6	64.0	1
BIF$_6$-G1	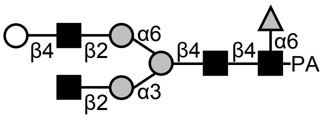	5.8	62.0	1

BIF$_6$-G2	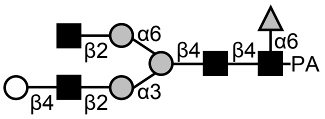	5.9	62.6	1
BIBs	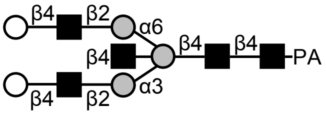	6.5	70.6	1
BIBsF$_6$	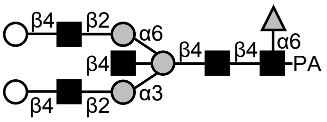	6.7	80.0	1
BIBsF$_6$-G1	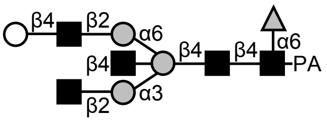	6.0	78.4	1
diF$_3$BI	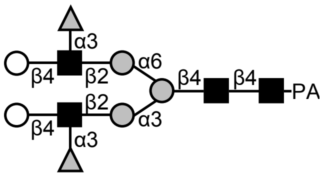	8.1	39.4	1
Gaβ$_4$1BI-G2	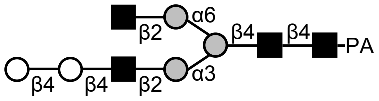	6.2	54.8	2
Gaβ$_4$1BI	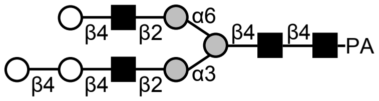	7.0	53.9	2
Gaβ$_4$2BI	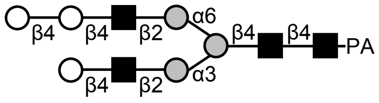	7.0	56.4	2
Gaβ$_4$12BI	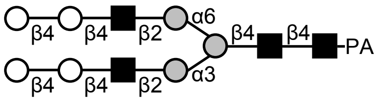	7.5	54.8	2
diF$_3$Gaβ$_4$12BI	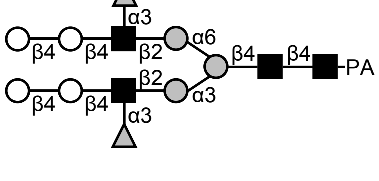	9.3	38.6	2
TR	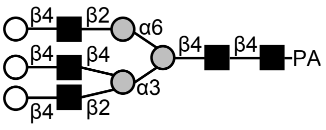	7.4	65.3	1

TRF$_6$	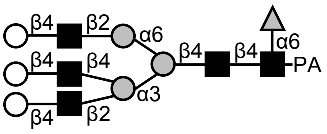	7.6	73.2	1
F$_3$(3)TR	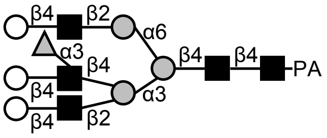	8.2	63.6	1
TR124	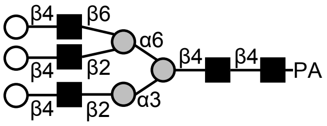	7.7	46.2	1
TRF$_6$	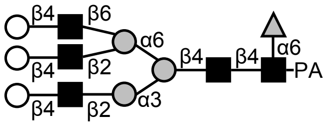	7.9	54.6	1
TE	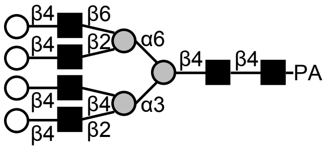	8.7	55.8	1
TEF$_6$	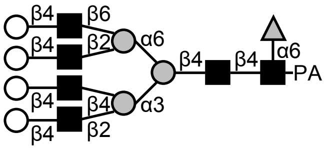	8.9	63.7	1
F$_3$(3)TE	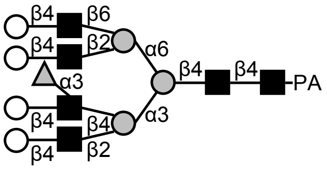	9.4	54.2	1

【Sialyl-complex type】

6N-MO1	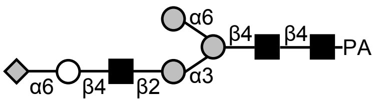	6.0	55.3	2
6N-Ldn1M3B	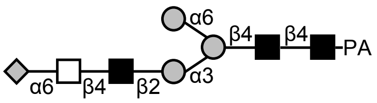	5.5	54.3	2
3N-MO1F$_6$	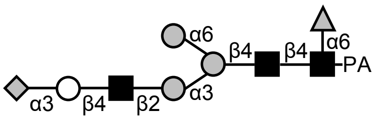	5.7	68.6	1

3N-MO2F_6	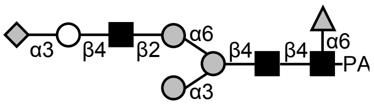	5.6	77.7	1
03N-BI	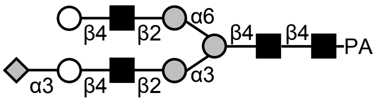	6.4	69.7	1
30N-BI	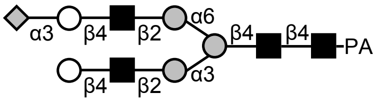	6.4	68.9	1
33N-BI	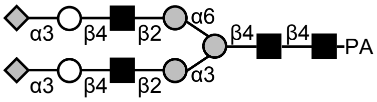	6.4	81.9	1
03N-BI-G2	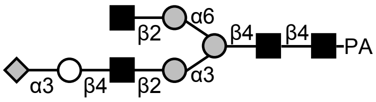	5.7	67.2	2
06N-BI	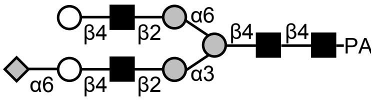	6.8	64.3	1
60N-BI	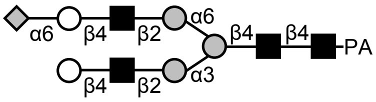	6.8	68.1	1
66N-BI	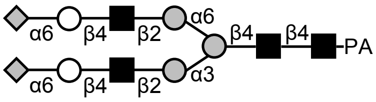	7.3	75.2	1
36N-BI	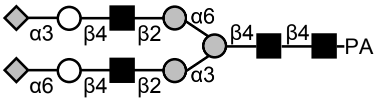	6.9	76.5	1
63N-BI	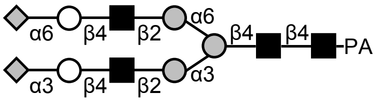	6.9	80.5	1
06N-BI-G2	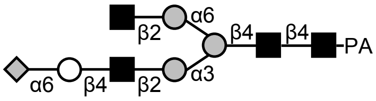	6.1	61.7	2
03N-BIF_6-G2	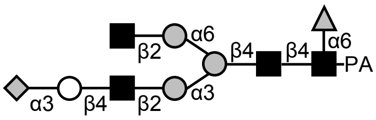	6.0	75.9	1

30N-BIF$_6$-G1	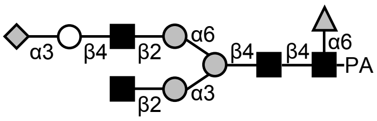	5.8	74.6	1
03N-BIF$_6$	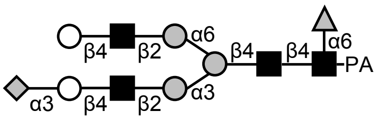	6.6	77.1	1
30N-BIF$_6$	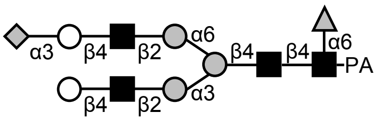	6.6	76.4	1
33N-BIF$_6$	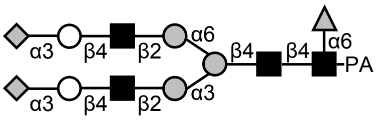	6.7	89.0	1
06N-BIF$_6$	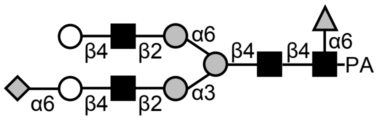	7.1	72.5	1
66N-BIF$_6$	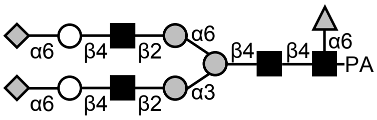	7.5	81.4	1
36N-BIF$_6$	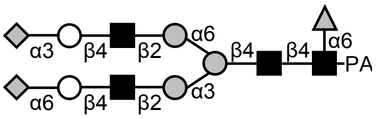	7.1	84.2	1
06N-BIBsF$_6$-G2	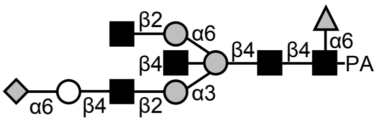	6.6	82.7	2
06N-BIBs	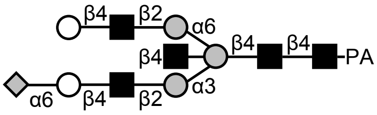	7.1	76.9	2
06N-BIBsF$_6$	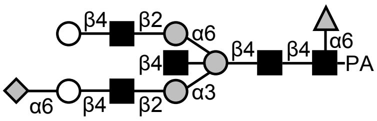	7.2	86.0	2
06N-BI(Ldn1)	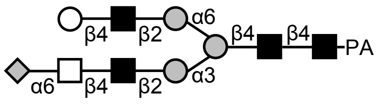	6.4	62.6	2
06N-BI(Ldn2)	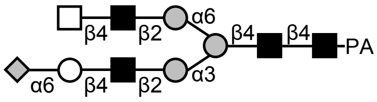	6.5	67.3	2

06N-BI(Ldn12)	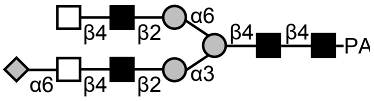	6.1	66.2	2
66N-BI(Ldn1)	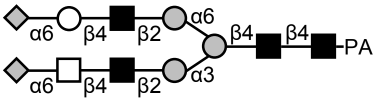	6.9	73.6	2
66N-BI(Ldn2)	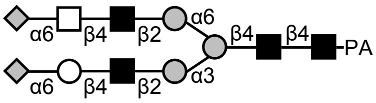	7.0	76.4	2
66N-BI(Ldn12)	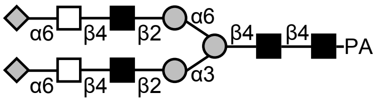	6.6	75.8	2
06N-Gaβ42BI	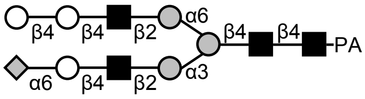	7.4	64.4	2
06N-Gaβ42BIF6	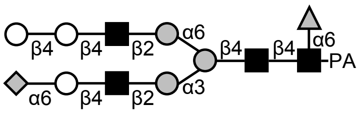	7.6	72.2	2
06N-Gaβ42BIBs	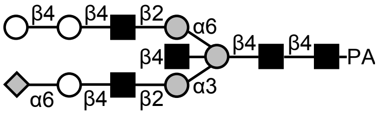	7.5	76.7	2
06N-Gaβ42BIBsF6	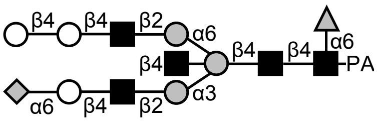	7.7	85.8	2
0xN-Gaβ41BI	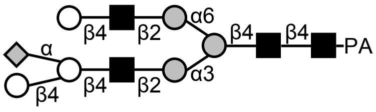	7.2	62.6	2
06N-Gnβx2BI	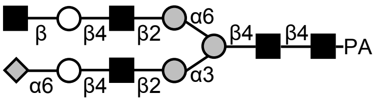	7.1	66.1	2
06N-Ln2BI	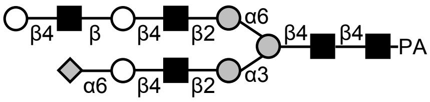	7.8	66.9	2
06N-Gaβ42Ln2BI	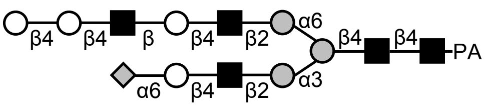	8.5	68.2	2

03N-Gaβ41F3BI-G2	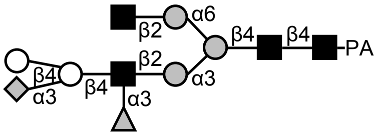	7.2	56.6	2
03N-Gaβ412F312BI	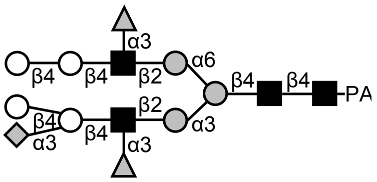	9.2	50.8	2
30N-Gaβ412F312BI	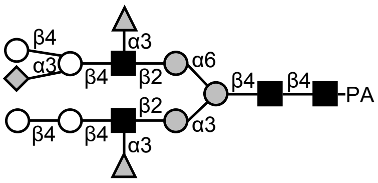	9.3	51.8	2
33N-Gaβ41F31BI	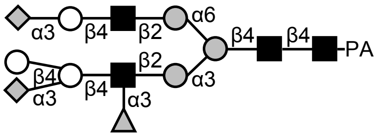	7.8	70.2	2
33N-Gaβ42F32BI	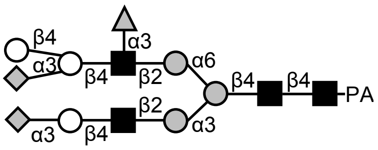	7.8	72.4	2
33N-Gaβ412F312BI	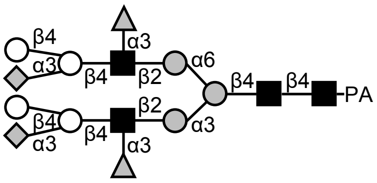	9.3	61.9	2
063N-TR3	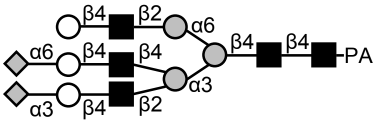	7.7	79.8	1
663N-TR	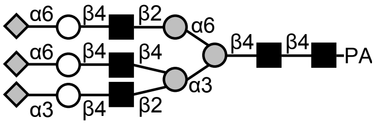	8.2	89.4	1
666N-TR	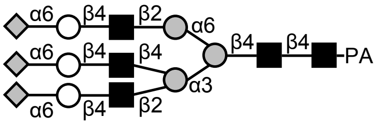	8.6	93.7	1

63(6)6N-TR123(Lm3)	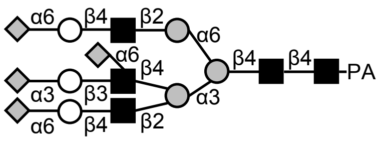	8.4	99.6	1
333N-TR	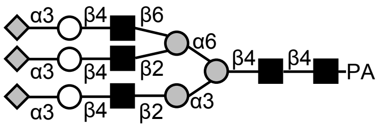	7.5	79.7	1
303N-TR	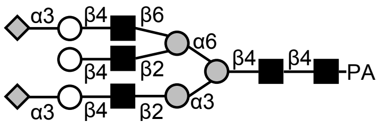	7.5	68.3	1
006N-TR4	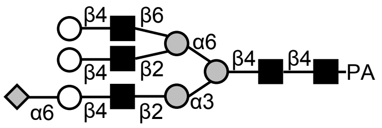	8.1	54.1	2
333N-TRF6	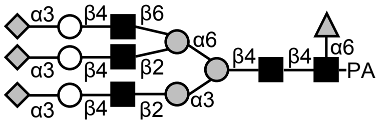	7.7	87.2	1
333N-Gaβ4124F3124TR	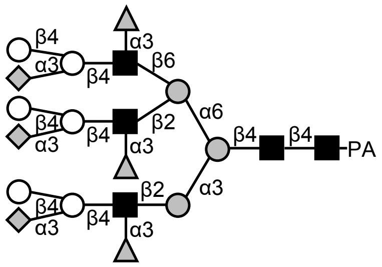	11.3	65.5	2

【Linkage region of glycosaminoglycan】

Linkage3	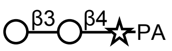	2.6	15.1	1
Linkage4	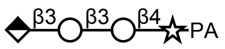	4.1	25.1	1
Linkage5	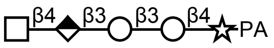	4.5	31.3	1
Linkage6	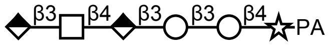	6.1	32.3	1

【O-linked glycan】

XG	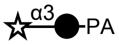	1.7	15.1	1
X2G	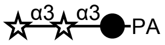	2.1	20.7	1
Tn	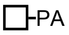	0.6	20.3	1
6N-Tn	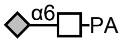	1.9	21.8	1
core1	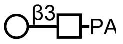	1.7	14.0	1
core3	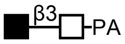	1.4	22.9	1
core6	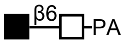	1.8	22.6	1
core7	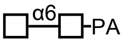	1.7	24.1	1
core8	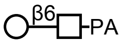	2.0	18.3	1
03N-core1	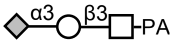	2.5	30.5	1
60N-core1	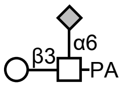	2.7	24.8	1
Ga$\beta_4$core1	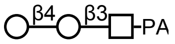	2.4	18.6	1
Ganβ_3Gb$_4$core1	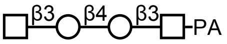	2.9	21.4	1
F$_3$Ganβ_3Ga$\beta_4$core1	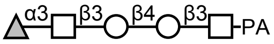	3.3	27.7	1
Ganα_3(F$_2$)core1	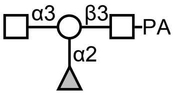	2.4	23.8	1
36N-core1	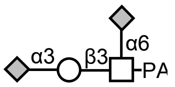	3.5	40.0	1
3N-Gan$\beta_4$core6	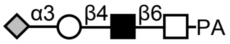	3.0	41.5	1
Ga$\beta_4$core2	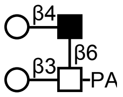	3.1	17.0	1

30N-Gaβ4core2	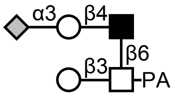	3.6	31.8	1
03N-Gaβ4core2	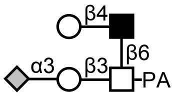	3.8	34.0	1
33N-Gaβ4core2	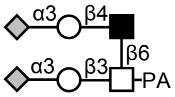	4.2	51.6	1
3N-Lnβ2F	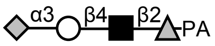	3.1	39.0	1

Supplementary Table S5

Fraction No.		R_{stria}	S_{stria}	R_{std}	S_{std}	R_{calc}	S_{calc}	error R_{std}	error S_{std}	error R_{calc}	error S_{calc}
N	1	31.757	8.37	32.7	8.4			2.8	0.7		
	2	33.304	7.51	34.4	7.5			3.0	0.4		
	3	35.137	9.05	36.4	9.1			3.4	0.9		
	4-1	38.14	7.46	39.4	7.5			3.1	0.4		
	4-2	38.14	8.21	38.7	8.2			1.3	0.4		
	5	40.152	6.64	41.2	6.6			2.5	0.2		
	6-1	42.858	3.15	43.1	3.2			0.6	0.0		
	6-2	42.858	6.02	44.5	6.1			3.7	0.5		
	7-1	43.755	4.11	44.5	4.1			1.8	0.5		
	7-2	43.755	5.01	43.7	5.0			0.1	0.0		
	8-1	45.266	5.8	46.2	5.8			2.1	0.2		
	8-2	45.266	7.3	47.0	7.3			3.8	0.4		
	8-3	45.266	9.67	47.0	9.8			3.6	0.8		
	11-1	51.191	4.87	52.3	4.9			2.2	0.0		
	12-1	52.739	3.55			51.5	3.4			2.4	4.4
	12-2	53.507	4.45	54.0	4.4			0.8	0.2		
	12-3	53.507	5.61	53.7	5.6			0.4	0.2		
	14-2	55.595	6.31	56.3	6.4			1.3	0.8		
	17	59.934	5.12	60.3	5.1			0.6	0.8		
	20-1	64.501	5.17	64.7	5.2			0.3	0.2		
	21-1	66.000	5.16	66.6	5.2			0.9	0.0		
	22-1	68.153	5.8			70.2	5.8			2.9	0.0
	25	76.047	5.38	76.1	5.4			0.0	0.4		
	26	78.263	5.97	78.4	6.0			0.2	0.3		
	27	79.883	6.65	80.0	6.7			0.1	0.7		
A1	2	53.324	5.83	55.3	6.0			3.6	3.0		
	3-1	54.312	6.47	56.2	6.6			3.4	2.0		
	3-2	54.312	7.24	56.6	7.4			4.0	2.2		
	4-1	58.874	6.03			61.2	5.9			3.8	-2.2
	4-2	58.874	6.74	60.7	6.9			3.0	2.2		
	5-1	59.998	6.05	61.7	6.1			2.8	0.8		
	6	61.064	8.07			62.6	8.4			2.5	3.9
	8-1	62.732	6.06			62.9	6.1			0.3	0.7
	8-2	62.732	6.71			64.9	6.5			3.3	3.2

	8-3	62.732	7.42	64.4	7.5			2.6	1.1		
	9	64.148	5.96	67.2	5.7			4.5	4.6		
	11	67.625	5.89			69.4	5.9			2.6	0.2
	12	68.806	6.28			71.0	6.4			3.1	1.9
	13	70.422	6.93	72.5	7.1			2.9	1.8		
	14-1	71.278	6.3			74.4	6.4			4.2	1.6
	14-2	71.278	7.61	72.2	7.6			1.3	0.1		
	17-1	75.313	6.02			76.6	5.9			1.7	2.0
	17-2	75.313	6.49	76.4	6.6			1.4	1.7		
	18	76.811	5.57	77.7	5.6			1.1	0.5		
	19	79.04	6.08			78.9	6.2			0.2	1.9
	20	81.076	6.55	82.7	6.6			2.0	0.8		
	21	84.022	7.1	86.2	7.2			2.5	1.4		
	22	86.338	6.29			89.1	6.5			3.1	3.2
A2	14-1	71.51	7.11	75.2	7.3			4.9	2.6		
	15	73.106	6.68	76.5	6.9			4.4	3.2		
	17	76.989	6.89			79.8	7.1			3.5	3.0
	18	77.89	7.32	81.4	7.5			4.3	2.4		
	19	79.027	6.6			82.0	6.8			3.6	2.9
	20	80.969	6.9	84.2	7.1			3.8	2.8		
	23	85.992	6.54	89.0	6.7			3.4	2.4		

Supplementary Table S6

Fraction No.		Glycan composition with possible α2,3-/ α2,6- combination	Ion species	Calculated *m/z*	Observed *m/z*
A2	14-2	PA-Hex6HexNAc5dHex1NeuAc(α2.3-)2	$[M+Na]^+$	2861.084265	
		PA-Hex6HexNAc5dHex1NeuAc(α2.6-)1NeuAc(α2.3-)1	$[M+Na]^+$	2889.115565	2889.09
		PA-Hex6HexNAc5dHex1NeuAc(α2.6-)2	$[M+Na]^+$	2917.146865	
A2	16-1	PA-Hex6HexNAc6dHex1NeuAc(α2.3-)2	$[M+Na]^+$	3064.163665	
		PA-Hex6HexNAc6dHex1NeuAc(α2.6-)1NeuAc(α2.3-)1	$[M+Na]^+$	3092.194965	3092.21
		PA-Hex6HexNAc6dHex1NeuAc(α2.6-)2	$[M+Na]^+$	3120.226265	
	16-2	PA-Hex7HexNAc6dHex1NeuAc(α2.3-)2	$[M+Na]^+$	3226.216465	
		PA-Hex7HexNAc6dHex1NeuAc(α2.6-)1NeuAc(α2.3-)1	$[M+Na]^+$	3254.247765	3254.24
		PA-Hex7HexNAc6dHex1NeuAc(α2.6-)2	$[M+Na]^+$	3282.279065	
A2	24	PA-Hex5HexNAc5dHex1NeuAc(α2.3-)2	$[M+Na]^+$	2699.031465	
		PA-Hex5HexNAc5dHex1NeuAc(α2.6-)1NeuAc(α2.3-)1	$[M+Na]^+$	2727.062765	
		PA-Hex5HexNAc5dHex1NeuAc(α2.6-)2	$[M+Na]^+$	2755.094065	2755.11
A2	25	PA-Hex5HexNAc5dHex1NeuAc(α2.3-)2	$[M+Na]^+$	2699.031465	
		PA-Hex5HexNAc5dHex1NeuAc(α2.6-)1NeuAc(α2.3-)1	$[M+Na]^+$	2727.062765	2727.06
		PA-Hex5HexNAc5dHex1NeuAc(α2.6-)2	$[M+Na]^+$	2755.094065	
A2	27	PA-Hex5HexNAc5dHex1NeuAc(α2.3-)2	$[M+Na]^+$	2699.031465	2699.04
		PA-Hex5HexNAc5dHex1NeuAc(α2.6-)1NeuAc(α2.3-)1	$[M+Na]^+$	2727.062765	
		PA-Hex5HexNAc5dHex1NeuAc(α2.6-)2	$[M+Na]^+$	2755.094065	
A3	10	PA-Hex6HexNAc5dHex1NeuAc(α2.3-)3	$[M+Na]^+$	3165.211265	
		PA-Hex6HexNAc5dHex1NeuAc(α2.6-)1NeuAc(α2.3-)2	$[M+Na]^+$	3193.242565	
		PA-Hex6HexNAc5dHex1NeuAc(α2.6-)2NeuAc(α2.3-)1	$[M+Na]^+$	3221.273865	3221.28
		PA-Hex6HexNAc5dHex1NeuAc(α2.6-)3	$[M+Na]^+$	3249.305165	
A3	12	PA-Hex6HexNAc5dHex1NeuAc(α2.3-)3	$[M+Na]^+$	3165.211265	
		PA-Hex6HexNAc5dHex1NeuAc(α2.6-)1NeuAc(α2.3-)2	$[M+Na]^+$	3193.242565	3193.24
		PA-Hex6HexNAc5dHex1NeuAc(α2.6-)2NeuAc(α2.3-)1	$[M+Na]^+$	3221.273865	
		PA-Hex6HexNAc5dHex1NeuAc(α2.6-)3	$[M+Na]^+$	3249.305165	
A3	13	PA-Hex6HexNAc3dHex1NeuAc(α2.3-)1S1	$[M+Na]^+$	2353.835065	
		same as the above	$[M-H+2Na]^+$	2375.816965	

		PA-Hex6HexNAc3dHex1NeuAc(α2.6-)1S1	$[M+Na]^+$	2381.866365	
		same as the above	$[M-H+2Na]^+$	2403.848265	2403.83
A3	14	PA-Hex6HexNAc5dHex1NeuAc(α2.3-)3	$[M+Na]^+$	3165.211265	3165.2
		PA-Hex6HexNAc5dHex1NeuAc(α2.6-)1NeuAc(α2.3-)2	$[M+Na]^+$	3193.242565	
		PA-Hex6HexNAc5dHex1NeuAc(α2.6-)2NeuAc(α2.3-)1	$[M+Na]^+$	3221.273865	
		PA-Hex6HexNAc5dHex1NeuAc(α2.6-)3	$[M+Na]^+$	3249.305165	
A3	15-1	PA-Hex6HexNAc5NeuAc(α2.3-)3	$[M+Na]^+$	3019.153365	
		PA-Hex6HexNAc5NeuAc(α2.6-)1NeuAc(α2.3-)2	$[M+Na]^+$	3047.184665	
		PA-Hex6HexNAc5NeuAc(α2.6-)2NeuAc(α2.3-)1	$[M+Na]^+$	3075.215965	3075.22
		PA-Hex6HexNAc5NeuAc(α2.6-)3	$[M+Na]^+$	3103.247265	
	15-2	PA-Hex6HexNAc6dHex1NeuAc(α2.3-)3	$[M+Na]^+$	3368.290665	
	-1	PA-Hex6HexNAc6dHex1NeuAc(α2.6-)1NeuAc(α2.3-)2	$[M+Na]^+$	3396.321965	3396.3
	-2	PA-Hex6HexNAc6dHex1NeuAc(α2.6-)2NeuAc(α2.3-)1	$[M+Na]^+$	3424.353265	3242.36
		PA-Hex6HexNAc6dHex1NeuAc(α2.6-)3	$[M+Na]^+$	3452.384565	
	15-3	PA-Hex7HexNAc6dHex1NeuAc(α2.3-)3	$[M+Na]^+$	3530.343465	
	-1	PA-Hex7HexNAc6dHex1NeuAc(α2.6-)1NeuAc(α2.3-)2	$[M+Na]^+$	3558.374765	3558.36
	-2	PA-Hex7HexNAc6dHex1NeuAc(α2.6-)2NeuAc(α2.3-)1	$[M+Na]^+$	3586.406065	3586.39
		PA-Hex7HexNAc6dHex1NeuAc(α2.6-)3	$[M+Na]^+$	3614.437365	
A3	18	PA-Hex6HexNAc5dHex1NeuAc(α2.3-)3	$[M+Na]^+$	3165.211265	
		PA-Hex6HexNAc5dHex1NeuAc(α2.6-)1NeuAc(α2.3-)2	$[M+Na]^+$	3193.242565	
		PA-Hex6HexNAc5dHex1NeuAc(α2.6-)2NeuAc(α2.3-)1	$[M+Na]^+$	3221.273865	3221.28
		PA-Hex6HexNAc5dHex1NeuAc(α2.6-)3	$[M+Na]^+$	3249.305165	
A4	6	PA-Hex7HexNAc6dHex1NeuAc(α2.3-)4	$[M+Na]^+$	3834.470465	
		PA-Hex7HexNAc6dHex1NeuAc(α2.6-)1NeuAc(α2.3-)3	$[M+Na]^+$	3862.501765	
		PA-Hex7HexNAc6dHex1NeuAc(α2.6-)2NeuAc(α2.3-)2	$[M+Na]^+$	3890.533065	3890.49
		PA-Hex7HexNAc6dHex1NeuAc(α2.6-)3NeuAc(α2.3-)1	$[M+Na]^+$	3918.564365	
		PA-Hex7HexNAc6dHex1NeuAc(α2.6-)4	$[M+Na]^+$	3946.595665	
A4	7	PA-Hex7HexNAc6dHex1NeuAc(α2.3-)4	$[M+Na]^+$	3834.470465	
		PA-Hex7HexNAc6dHex1NeuAc(α2.6-)1NeuAc(α2.3-)3	$[M+Na]^+$	3862.501765	3862.48
		PA-Hex7HexNAc6dHex1NeuAc(α2.6-)2NeuAc(α2.3-)2	$[M+Na]^+$	3890.533065	
		PA-Hex7HexNAc6dHex1NeuAc(α2.6-)3NeuAc(α2.3-)1	$[M+Na]^+$	3918.564365	
		PA-Hex7HexNAc6dHex1NeuAc(α2.6-)4	$[M+Na]^+$	3946.595665	

Supplementary Table S7

PA, pyridylaminated; M3PA, Man3GlcNAc2-PA; Hex, hexose; HexNAc, N-acetylhexosamin; dHex, deoxy-hexose; Pen, pentose; NANA, N-acetylneuraminic acid
Me, methylation; Ac, acetylation; HPO_3, phosphorylation; SO_3, sulfation

**asialo glycans**	reducing end		non-reducing end					modification					*m/z*			
	PA	M3PA	Hex	HexNAc	dHex	Pen	NANA	Me	Ac	HPO_3	SO_3	MS number	+H	+Na	+2H	+3H
Pen	1					1						228.11	229.12	251.10	115.06	77.04
dHex	1				1							242.13	243.13	265.12	122.07	81.72
Me-Pen	1					1		1				242.13	243.13	265.12	122.07	81.72
Me-Fuc	1				1			1				256.14	257.15	279.13	129.08	86.39
Hex	1		1									258.12	259.13	281.11	130.07	87.05
Me-Hex	1		1					1				272.14	273.14	295.13	137.08	91.72
HexNAc	1			1								299.15	300.16	322.14	150.58	100.72
Ac-Hex	1		1						1			300.13	301.14	323.12	151.07	101.05
Me-HexNAc	1			1				1				313.16	314.17	336.15	157.59	105.40
Ac-HexNAc	1			1					1			341.16	342.17	364.15	171.59	114.73
XG	1		1			1						390.16	391.17	413.15	196.09	131.06
Hex2	1		2									420.17	421.18	443.16	211.09	141.07
GNF	1			1	1							445.21	446.21	468.20	223.61	149.41
GnMan, core1, core8	1		1	1								461.20	462.21	484.19	231.61	154.74
M1'	1		1	1								461.20	462.21	484.19	231.61	154.74
Ac-Hex2	1		2						1			462.18	463.19	485.17	232.10	155.07
Ac-GNF	1			1	1				1			487.22	488.22	510.21	244.62	163.41
GN2, core3, core6	1			2								502.23	503.23	525.22	252.12	168.42
X2G	1		1			2						522.21	523.21	545.20	262.11	175.08
Ac-GN2	1			2					1			544.24	545.25	567.23	273.13	182.42
GAG-L3	1		2			1						552.22	553.22	575.21	277.12	185.08
Hex3	1		3									582.23	583.23	605.22	292.12	195.08
GNdF	1			1	2							591.26	592.27	614.25	296.64	198.10
G-Gn-F	1		1	1	1							607.26	608.27	630.25	304.64	203.43
Ln-Man, Ga-core1	1		2	1								623.25	624.26	646.24	312.63	208.76
M2'	1		2	1								623.25	624.26	646.24	312.63	208.76
Ac-Hex3	1		3						1			624.24	625.25	647.23	313.13	209.09
GN2F	1			2	1							648.29	649.29	671.27	325.15	217.10
M1	1		1	2								664.28	665.29	687.27	333.15	222.43
dGn-Man	1		1	2								664.28	665.29	687.27	333.15	222.43
Ac-GN2F	1			2	1				1			690.30	691.30	713.29	346.16	231.11
H2F1X1	1		2		1	1						698.27	699.28	721.26	350.14	233.77
Ac-M1	1		1	2					1			706.29	707.30	729.28	354.15	236.44
Hex4	1		4									744.28	745.29	767.27	373.15	249.10
H1Hn1F2	1		1	1	2							753.32	754.32	776.31	377.67	252.11
mF-G-core1	1		2	1	1							769.31	770.32	792.30	385.66	257.44
M3'	1		3	1								785.31	786.31	808.30	393.66	262.78
Ac-Hex4	1		4						1			786.29	787.30	809.28	394.15	263.10
GN2dF	1			2	2							794.34	795.35	817.33	398.18	265.79
M1X	1		1	2		1						796.32	797.33	819.31	399.17	266.45
M1F	1		1	2	1							810.34	811.35	833.33	406.18	271.12
F-Gan-core1	1		1	2	1							810.34	811.35	833.33	406.18	271.12
M2	1		2	2								826.33	827.34	849.32	414.17	276.45
GnLn-Man	1		2	2								826.33	827.34	849.32	414.17	276.45
Gan-G-core1	1		2	2								826.33	827.34	849.32	414.17	276.45
G-core2	1		2	2								826.33	827.34	849.32	414.17	276.45
Ac-M1F	1		1	2	1				1			852.35	853.36	875.34	427.18	285.12
M1BS	1		1	3								867.36	868.37	890.35	434.69	290.13
Ac-M2	1		2	2					1			868.34	869.35	891.33	435.18	290.46
Hex5	1		5									906.33	907.34	929.32	454.17	303.12
M1FX	1		1	2	1	1						942.38	943.39	965.37	472.20	315.13
M4'	1		4	1								947.36	948.37	970.35	474.69	316.79
Ac-Hex5	1		5						1			948.34	949.35	971.33	475.18	317.12
M1dF	1		1	2	2							956.40	957.40	979.39	479.21	319.81
M2X	1		2	2		1						958.38	959.38	981.36	480.19	320.47
M2F	1		2	2	1							972.39	973.40	995.38	487.20	325.14
mF-Gan-G-core1	1		2	2	1							972.39	973.40	995.38	487.20	325.14
M3		1										988.39	989.39	1011.38	495.20	330.47
dLn-Man	1		3	2								988.39	989.39	1011.38	495.20	330.47
M1BSF	1		1	3	1							1013.42	1014.42	1036.41	507.72	338.81
Ac-M2F	1		2	2	1				1			1014.40	1015.41	1037.39	508.21	339.14
GnM2	1		2	3								1029.41	1030.42	1052.40	515.71	344.14
Ac-M3		1							1			1030.40	1031.40	1053.39	516.21	344.47
Hex6	1		6									1068.39	1069.39	1091.37	535.20	357.14
Ac2-M3		1							2			1072.41	1073.41	1095.40	537.21	358.48
M1dF-X	1		1	2	2	1						1088.44	1089.45	1111.43	545.23	363.82
M2FX	1		2	2	1	1						1104.43	1105.44	1127.42	553.22	369.15
M5'	1		5	1								1109.41	1110.42	1132.40	555.71	370.81
Ac-Hex6	1		6						1			1110.40	1111.40	1133.39	556.21	371.14
M2dF	1		2	2	2							1118.45	1119.46	1141.44	560.23	373.82

	M3X		1				1						1120.43	1121.44	1143.42	561.22	374.48
	M3F		1			1							1134.44	1135.45	1157.43	568.23	379.16
	GnM2BS			2	4								1136.42	1137.43	1159.41	569.22	379.81
	M4		1	1									1150.44	1151.45	1173.43	576.23	384.49
	M1BSdF	1		1	3	2							1159.48	1160.48	1182.46	580.75	387.50
	GnM2F	1		2	3	1							1175.47	1176.48	1198.46	588.74	392.83
	Ac-M3F		1			1				1			1176.45	1177.46	1199.44	589.23	393.16
	AG1		1		1								1191.47	1192.47	1214.45	596.74	398.16
	LnM2	1		3	3								1191.47	1192.47	1214.45	596.74	398.16
	Ac-M4		1	1						1			1192.45	1193.46	1215.44	597.23	398.49
	Hex7	1		7									1230.44	1231.45	1253.43	616.23	411.15
	LdnM2	1		2	4								1232.49	1233.50	1255.48	617.25	411.84
	Ac-AG1		1		1					1			1233.48	1234.48	1256.47	617.75	412.17
	Ac2-M4		1	1						2			1234.46	1235.47	1257.45	618.24	412.49
	M2dF-X	1		2	2	2	1						1250.49	1251.50	1273.48	626.25	417.84
	M3FX		1			1	1						1266.49	1267.49	1289.48	634.25	423.17
	M6'	1		6	1								1271.47	1272.47	1294.45	636.74	424.83
	Ac-Hex7	1		7						1			1272.45	1273.46	1295.44	637.23	425.16
	M3dF		1			2							1280.50	1281.51	1303.49	641.26	427.84
	M4X		1	1			1						1282.48	1283.49	1305.47	642.25	428.50
	M4F		1	1		1							1296.50	1297.50	1319.49	649.26	433.17
	M5		1	2									1312.49	1313.50	1335.48	657.25	438.50
	mF-GnM2F	1		2	3	2							1321.53	1322.54	1344.52	661.77	441.52
	AG1X		1		1		1						1323.51	1324.51	1346.50	662.76	442.18
	AG1F		1		1	1							1337.52	1338.53	1360.51	669.77	446.85
	LnM2F	1		3	3	1							1337.52	1338.53	1360.51	669.77	446.85
	Ac-M4F		1	1		1				1			1338.51	1339.51	1361.50	670.26	447.18
	MO, GnM4		1	1	1	0							1353.52	1354.53	1376.51	677.77	452.18
	Ac-M5		1	2						1			1354.50	1355.51	1377.49	678.26	452.51
	H1Hn2F2	1		1	4	2							1362.55	1363.56	1385.54	682.28	455.19
	GnM2BSF	1		2	4	1							1378.55	1379.56	1401.54	690.28	460.52
	LdnM2F	1		2	4	1							1378.55	1379.56	1401.54	690.28	460.52
	Hex8	1		8									1392.49	1393.50	1415.48	697.25	465.17
	AG1BS, AG12, LdnM3		1		2								1394.54	1395.55	1417.53	698.28	465.86
	LnM2BS	1		3	4								1394.54	1395.55	1417.53	698.28	465.86
	Ac2-M5		1	2						2			1396.51	1397.52	1419.50	699.26	466.51
	M3dF-X		1			2	1						1412.54	1413.55	1435.53	707.28	471.86
	mF-GnM2BSF			2	4	2							1428.54	1429.55	1451.53	715.28	477.19
	M4FX		1	1		1	1						1428.54	1429.55	1451.53	715.28	477.19
	M7'	1		7	1								1433.52	1434.53	1456.51	717.77	478.85
	Ac-Hex8	1		8						1			1434.50	1435.51	1457.49	718.26	479.17
	LdnM2BS	1		2	5								1435.57	1436.58	1458.56	718.79	479.53
	GnLdnM2	1		2	5								1435.57	1436.58	1458.56	718.79	479.53
	Ac-AG12		1		2					1			1436.56	1437.56	1459.54	719.28	479.86
	M4dF		1	1		2							1442.55	1443.56	1465.54	722.28	481.86
	M5X		1	2			1						1444.53	1445.54	1467.52	723.27	482.52
	M5F		1	2		1							1458.55	1459.56	1481.54	730.28	487.19
	dF-GnM2F	1		2	3	3							1467.59	1468.59	1490.58	734.80	490.20
	AG1FX		1		1	1	1						1469.57	1470.57	1492.55	735.79	490.86
	M6		1	3									1474.54	1475.55	1497.53	738.28	492.52
	mF-AG1F		1		1	2							1483.58	1484.59	1506.57	742.80	495.53
	mF-LnM2F	1		3	3	2							1483.58	1484.59	1506.57	742.80	495.53
	MOF, GnM4F		1	1	1	1							1499.58	1500.58	1522.57	750.80	500.87
	Ac-M5F		1	2		1				1			1500.56	1501.57	1523.55	751.29	501.19
	LnM4, GnM5, mGa-MO		1	2	1								1515.57	1516.58	1538.56	758.79	506.20
	BI'	1		5	3								1515.57	1516.58	1538.56	758.79	506.20
	Ac-M6		1	3						1			1516.55	1517.56	1539.54	759.28	506.53
	mF-LdnF	1		2	4	2							1524.61	1525.61	1547.60	763.31	509.21
	AG12X		1		2		1						1526.59	1527.59	1549.58	764.30	509.87
	AG1BSF, AG12F		1		2	1							1540.60	1541.61	1563.59	771.31	514.54
	LdnM3F		1		2	1							1540.60	1541.61	1563.59	771.31	514.54
	LnM2BSF	1		3	4	1							1540.60	1541.61	1563.59	771.31	514.54
	Hex9	1		9									1554.54	1555.55	1577.53	778.28	519.19
	MOBS		1	1	2	0							1556.60	1557.60	1579.59	779.31	519.87
	BI-G		1	1	2	0							1556.60	1557.60	1579.59	779.31	519.87
	GnM4BS		1	1	2	0							1556.60	1557.60	1579.59	779.31	519.87
	LdnM4		1	1	2								1556.60	1557.60	1579.59	779.31	519.87
	BI'(mLdn)	1		4	4								1556.60	1557.60	1579.59	779.31	519.87
	Ac2-M6		1	3						2			1558.57	1559.57	1581.55	780.29	520.53
	M4dF-X		1	1		2	1						1574.60	1575.60	1597.59	788.31	525.87
	dF-GnM2BSF			2	4	3							1574.60	1575.60	1597.59	788.31	525.87
	LdnM2BSF	1		2	5	1							1581.63	1582.64	1604.62	791.82	528.22
	GnLdnM2F	1		2	5	1							1581.63	1582.64	1604.62	791.82	528.22
	M8'	1		8	1								1595.57	1596.58	1618.56	798.79	532.86
	Ac-Hex9	1		9						1			1596.55	1597.56	1619.54	799.28	533.19
	AG12BS, AG123		1		3								1597.62	1598.63	1620.61	799.82	533.55

	LdnAG1, LdnM3BS		1		3								1597.62	1598.63	1620.61	799.82	533.55
	BI'(dLdn)	1		3	5								1597.62	1598.63	1620.61	799.82	533.55
	Ac-BI-G		1	1	2					1			1598.61	1599.62	1621.60	800.31	533.88
	M5dF		1	2		2							1604.61	1605.61	1627.60	803.31	535.88
	M6X		1	3			1						1606.59	1607.59	1629.58	804.30	536.54
	AG1dF-X		1		1	2	1						1615.62	1616.63	1638.61	808.82	539.55
	M6F		1	3		1							1620.60	1621.61	1643.59	811.31	541.21
	dF-AG1F		1		1	3							1629.64	1630.65	1652.63	815.83	544.22
	M7		1	4									1636.60	1637.60	1659.59	819.31	546.54
	dLdnM2	1		2	6								1638.65	1639.66	1661.64	820.33	547.22
	mF-MOF, mF-GnM4F		1	1	1	2							1645.63	1646.64	1668.62	823.82	549.55
	mGal-MOF		1	2	1	1							1661.63	1662.64	1684.62	831.82	554.88
	LnM4F, GnM5F		1	2	1	1							1661.63	1662.64	1684.62	831.82	554.88
	mF-BI'	1		5	3	1							1661.63	1662.64	1684.62	831.82	554.88
	Ac-M6F		1	3		1				1			1662.61	1663.62	1685.60	832.31	555.21
	AG12FX		1		2	1	1						1672.64	1673.65	1695.63	837.33	558.56
	LnM5,GnM6		1	3	1	0							1677.62	1678.63	1700.61	839.82	560.22
	H6HN3	1		6	3								1677.62	1678.63	1700.61	839.82	560.22
	Ac-M7		1	4						1			1678.61	1679.62	1701.60	840.31	560.54
	mF-AG1BSF, mF-AG12F		1		2	2							1686.66	1687.67	1709.65	844.34	563.23
	mF-LnM2BSF	1		3	4	2							1686.66	1687.67	1709.65	844.34	563.23
	mF-LdnM3F		1		2	2							1686.66	1687.67	1709.65	844.34	563.23
	MOBSF, BIF-G		1	1	2	1							1702.66	1703.66	1725.64	852.33	568.56
	GnM4BSF		1	1	2	1							1702.66	1703.66	1725.64	852.33	568.56
	LdnM4F		1	1	2	1							1702.66	1703.66	1725.64	852.33	568.56
	Hex10	1		10									1716.60	1717.60	1739.59	859.31	573.21
	BI		1	2	2								1718.65	1719.66	1741.64	860.33	573.89
	mGa-MOBS, mGa-BI-G		1	2	2								1718.65	1719.66	1741.64	860.33	573.89
	LnM4BS, GnM5BS		1	2	2								1718.65	1719.66	1741.64	860.33	573.89
	LdnM5, GnLnM4		1	2	2								1718.65	1719.66	1741.64	860.33	573.89
	Ac2-M7		1	4						2			1720.62	1721.63	1743.61	861.32	574.55
	mF-LdnBSF	1		2	5	2							1727.69	1728.69	1750.68	864.85	576.90
	mF-GnLdnM2F	1		2	5	2							1727.69	1728.69	1750.68	864.85	576.90
	AG12BSF, AG123F		1		3	1							1743.68	1744.69	1766.67	872.85	582.23
	LdnAG1F, LdnM3BSF		1		3	1							1743.68	1744.69	1766.67	872.85	582.23
	M9'	1		9	1								1757.62	1758.63	1780.61	879.82	586.88
	Ac-Hex10	1		10						1			1758.61	1759.61	1781.60	880.31	587.21
	BIBS-G, TR-2G		1	1	3								1759.68	1760.68	1782.67	880.85	587.57
	BI(mLdn), LdnM4BS		1	1	3								1759.68	1760.68	1782.67	880.85	587.57
	GnLdnM4		1	1	3								1759.68	1760.68	1782.67	880.85	587.57
	Ac-BI		1	2	2					1			1760.66	1761.67	1783.65	881.34	587.89
	M7X		1	4			1						1768.64	1769.65	1791.63	885.33	590.55
	dLdnM2F	1		2	6	1							1784.71	1785.72	1807.70	893.36	595.91
	M8, G1M7		1	5									1798.65	1799.66	1821.64	900.33	600.56
	AG123BS, AG1234		1		4								1800.70	1801.71	1823.69	901.36	601.24
	BI(dLdn), GnLdnM3BS		1		4								1800.70	1801.71	1823.69	901.36	601.24
	mGa-mF-MOF		1	2	1	2							1807.69	1808.69	1830.68	904.85	603.57
	mF-LnM4F, mF-GnM5F		1	2	1	2							1807.69	1808.69	1830.68	904.85	603.57
	AG12dF-X		1		2	2	1						1818.70	1819.71	1841.69	910.36	607.24
	LnM5F		1	3	1	1							1823.68	1824.69	1846.67	912.85	608.90
	dF-AG1BSF, dF-AG12F		1		2	3							1832.72	1833.73	1855.71	917.37	611.91
	mGa-LnM5, LnM6		1	4	1	0							1839.68	1840.68	1862.67	920.85	614.23
	H7HN3		1	4	1								1839.68	1840.68	1862.67	920.85	614.23
	Ac-M8		1	5						1			1840.66	1841.67	1863.65	921.34	614.56
	mF-MOBSF, mF-BIF-G		1	1	2	2							1848.71	1849.72	1871.70	925.36	617.25
	mF-GnM4BSF		1	1	2	2							1848.71	1849.72	1871.70	925.36	617.25
	mF-LdnM4F		1	1	2	2							1848.71	1849.72	1871.70	925.36	617.25
	BIF, mGa-BIF-G		1	2	2	1							1864.71	1865.72	1887.70	933.36	622.58
	mF-BI, mGa-MOBSF,		1	2	2	1							1864.71	1865.72	1887.70	933.36	622.58
	LnM4BSF, GnLnM4F		1	2	2	1							1864.71	1865.72	1887.70	933.36	622.58
	GnM5BSF, LdnM5F		1	2	2	1							1864.71	1865.72	1887.70	933.36	622.58
	dF-GnLdnM2F	1		2	5	3							1873.74	1874.75	1896.73	937.88	625.59
	Hex11	1		11									1878.65	1879.66	1901.64	940.33	627.22
	mGal-BI, dLnM4		1	3	2								1880.70	1881.71	1903.69	941.36	627.91
	LnM5BS, GnLnM5		1	3	2								1880.70	1881.71	1903.69	941.36	627.91
	Ac2-M8		1	5						2			1882.67	1883.68	1905.66	942.34	628.56
	mF-AG12BSF, mF-AG123F		1		3	2							1889.74	1890.75	1912.73	945.88	630.92
	mF-LdnM3BSF		1		3	2							1889.74	1890.75	1912.73	945.88	630.92
	mF-GnLdnM3F		1		3	2							1889.74	1890.75	1912.73	945.88	630.92
	BIBSF-G, TRF-2G		1	1	3	1							1905.73	1906.74	1928.72	953.87	636.25
	BI(mLdn)F, LdnM4BSF		1	1	3	1							1905.73	1906.74	1928.72	953.87	636.25
	GnLdnM4F		1	1	3	1							1905.73	1906.74	1928.72	953.87	636.25
	Ac-Hex11	1		11						1			1920.66	1921.67	1943.65	961.34	641.23
	BIBS, TR-G		1	2	3								1921.73	1922.74	1944.72	961.87	641.58
	mGa-BIBS-G		1	2	3								1921.73	1922.74	1944.72	961.87	641.58
	GnLnM4BS		1	2	3								1921.73	1922.74	1944.72	961.87	641.58

	LdnM5BS, GnLdnM5		1	2	3								1921.73	**1922.74**	**1944.72**	**961.87**	**641.58**
	M8X		1	5			1						1930.69	**1931.70**	**1953.68**	**966.35**	**644.57**
	mF-dLdnM2F	1		2	6	2							1930.77	**1931.77**	**1953.76**	**966.39**	**644.60**
	AG123BSF, AG1234F		1		4	1							1946.76	**1947.77**	**1969.75**	**974.39**	**649.93**
	BI(dLdn)F, GnLdnM3BSF		1		4	1							1946.76	**1947.77**	**1969.75**	**974.39**	**649.93**
	M9, G1M8		1	6									1960.70	**1961.71**	**1983.69**	**981.36**	**654.57**
	TRBS-2G		1	1	4								1962.76	**1963.76**	**1985.75**	**982.39**	**655.26**
	BI(mLdn)BS		1	1	4								1962.76	**1963.76**	**1985.75**	**982.39**	**655.26**
	GnLdnM4BS		1	1	4								1962.76	**1963.76**	**1985.75**	**982.39**	**655.26**
	mF-LnM5F		1	3	1	2							1969.74	**1970.75**	**1992.73**	**985.88**	**657.59**
	trF-AG12F		1		2	4							1978.78	**1979.78**	**2001.77**	**990.40**	**660.60**
	mG-LnM5F		1	4	1	1							1985.73	**1986.74**	**2008.72**	**993.87**	**662.92**
	dF-BIF-G		1	1	2	3							1994.77	**1995.78**	**2017.76**	**998.39**	**665.93**
	Ga-LnM6		1	5	1								2001.73	**2002.74**	**2024.72**	**1001.87**	**668.25**
	Ac-M9		1	6						1			2002.71	**2003.72**	**2025.70**	**1002.36**	**668.58**
	AG1234BS		1		5								2003.78	**2004.79**	**2026.77**	**1002.90**	**668.93**
	BI(dLdn)BS		1		5								2003.78	**2004.79**	**2026.77**	**1002.90**	**668.93**
	mGa-mF-MOBSF		1	2	2	2							2010.77	**2011.77**	**2033.76**	**1006.39**	**671.26**
	mGa-mF-BIF-G		1	2	2	2							2010.77	**2011.77**	**2033.76**	**1006.39**	**671.26**
	diF-BI, mF-BIF		1	2	2	2							2010.77	**2011.77**	**2033.76**	**1006.39**	**671.26**
	mF-LnM4BSF		1	2	2	2							2010.77	**2011.77**	**2033.76**	**1006.39**	**671.26**
	mF-GnM5BSF		1	2	2	2							2010.77	**2011.77**	**2033.76**	**1006.39**	**671.26**
	mF-GnLnM4F		1	2	2	2							2010.77	**2011.77**	**2033.76**	**1006.39**	**671.26**
	mF-LdnM5F		1	2	2	2							2010.77	**2011.77**	**2033.76**	**1006.39**	**671.26**
	trF-GnLdnM2F	1		2	5	4							2019.80	**2020.81**	**2042.79**	**1010.91**	**674.27**
	mGa-BIF, mGa-mF-BI		1	3	2	1							2026.76	**2027.77**	**2049.75**	**1014.39**	**676.59**
	LnM5BSF, GnLnM5F		1	3	2	1							2026.76	**2027.77**	**2049.75**	**1014.39**	**676.59**
	diLnM4F		1	3	2	1							2026.76	**2027.77**	**2049.75**	**1014.39**	**676.59**
	dF-AG12BSF, dF-AG123F		1		3	3							2035.80	**2036.81**	**2058.79**	**1018.91**	**679.61**
	dF-LdnAG1F		1		3	3							2035.80	**2036.81**	**2058.79**	**1018.91**	**679.61**
	Hex12	1		12									2040.70	**2041.71**	**2063.69**	**1021.36**	**681.24**
	dGa-BI		1	4	2								2042.76	**2043.76**	**2065.75**	**1022.39**	**681.93**
	dLnM5, mGa-LnM5BS		1	4	2								2042.76	**2043.76**	**2065.75**	**1022.39**	**681.93**
	Ac2-M9		1	6						2			2044.72	**2045.73**	**2067.71**	**1023.37**	**682.58**
	mF-BIBSF-G, mF-TRF-2G		1	1	3	2							2051.79	**2052.80**	**2074.78**	**1026.90**	**684.94**
	mF-LdnMOF		1	1	3	2							2051.79	**2052.80**	**2074.78**	**1026.90**	**684.94**
	mF-LdnM4BSF		1	1	3	2							2051.79	**2052.80**	**2074.78**	**1026.90**	**684.94**
	mF-GnLdnM4F		1	1	3	2							2051.79	**2052.80**	**2074.78**	**1026.90**	**684.94**
	BIBSF, mF-BIBS, TRF-G		1	2	3	1							2067.79	**2068.79**	**2090.78**	**1034.90**	**690.27**
	mGa-BIBSF-G		1	2	3	1							2067.79	**2068.79**	**2090.78**	**1034.90**	**690.27**
	mGa-mF-BIBS-G		1	2	3	1							2067.79	**2068.79**	**2090.78**	**1034.90**	**690.27**
	GnLnM4BSF		1	2	3	1							2067.79	**2068.79**	**2090.78**	**1034.90**	**690.27**
	LdnM5BSF, GnLdnM5F		1	2	3	1							2067.79	**2068.79**	**2090.78**	**1034.90**	**690.27**
	dF-dLdnM2F	1		2	6	3							2076.82	**2077.83**	**2099.81**	**1039.42**	**693.28**
	Ac-Hex12	1		12						1			2082.71	**2083.72**	**2105.70**	**1042.36**	**695.25**
	TR, mGa-BIBS		1	3	3								2083.78	**2084.79**	**2106.77**	**1042.90**	**695.60**
	dLnM4BS, GnLnM5BS		1	3	3								2083.78	**2084.79**	**2106.77**	**1042.90**	**695.60**
	M9X		1	6			1						2092.75	**2093.75**	**2115.73**	**1047.38**	**698.59**
	mF-AG123BSF		1		4	2							2092.82	**2093.83**	**2115.81**	**1047.42**	**698.61**
	mF-GnLdnM3BSF		1		4	2							2092.82	**2093.83**	**2115.81**	**1047.42**	**698.61**
	mF-dLdnM3F		1		4	2							2092.82	**2093.83**	**2115.81**	**1047.42**	**698.61**
	TRBSF-2G		1	1	4	1							2108.81	**2109.82**	**2131.80**	**1055.41**	**703.95**
	BI(mLdn)BSF		1	1	4	1							2108.81	**2109.82**	**2131.80**	**1055.41**	**703.95**
	GnLdnM4BSF		1	1	4	1							2108.81	**2109.82**	**2131.80**	**1055.41**	**703.95**
	G1M9		1	7									2122.76	**2123.76**	**2145.74**	**1062.39**	**708.59**
	TRBS-G		1	2	4	0							2124.81	**2125.82**	**2147.80**	**1063.41**	**709.28**
	TR(mLdn)		1	2	4								2124.81	**2125.82**	**2147.80**	**1063.41**	**709.28**
	GnLdnM5BS		1	2	4								2124.81	**2125.82**	**2147.80**	**1063.41**	**709.28**
	dFdGa-BI'	1		7	3	2							2131.79	**2132.80**	**2154.78**	**1066.90**	**711.60**
	H2Hn7F2	1		2	7	2							2133.85	**2134.85**	**2156.84**	**1067.93**	**712.29**
	AG12345, AG1234BSF		1		5	1							2149.84	**2150.85**	**2172.83**	**1075.93**	**717.62**
	dLdnM3BSF		1		5	1							2149.84	**2150.85**	**2172.83**	**1075.93**	**717.62**
	dF-BIF		1	2	2	3							2156.82	**2157.83**	**2179.81**	**1079.42**	**719.95**
	diF-BIF		1	2	2	3							2156.82	**2157.83**	**2179.81**	**1079.42**	**719.95**
	Ac-G1M9		1	7						1			2164.77	**2165.77**	**2187.76**	**1083.39**	**722.60**
	mGal-diF-BI		1	3	2	2							2172.82	**2173.83**	**2195.81**	**1087.42**	**725.28**
	mGal-mF-BIF		1	3	2	2							2172.82	**2173.83**	**2195.81**	**1087.42**	**725.28**
	mF-LnM5BSF		1	3	2	2							2172.82	**2173.83**	**2195.81**	**1087.42**	**725.28**
	mF-GnLnM5F		1	3	2	2							2172.82	**2173.83**	**2195.81**	**1087.42**	**725.28**
	mF-dLnM4F		1	3	2	2							2172.82	**2173.83**	**2195.81**	**1087.42**	**725.28**
	trF-AG12BSF		1		3	4							2181.86	**2182.86**	**2204.84**	**1091.94**	**728.29**
	trF-AG123F		1		3	4							2181.86	**2182.86**	**2204.84**	**1091.94**	**728.29**
	trF-LdnAG1F		1		3	4							2181.86	**2182.86**	**2204.84**	**1091.94**	**728.29**
	dGa-BIF, dGa-mF-BI		1	4	2	1							2188.81	**2189.82**	**2211.80**	**1095.41**	**730.61**
	mG-LnM5BSF		1	4	2	1							2188.81	**2189.82**	**2211.80**	**1095.41**	**730.61**
	diLnM5F		1	4	2	1							2188.81	**2189.82**	**2211.80**	**1095.41**	**730.61**

	dF-BIBSF-G		1	1	3	3							2197.85	2198.86	2220.84	1099.93	733.62
	dF-TRF-2G		1	1	3	3							2197.85	2198.86	2220.84	1099.93	733.62
	dF-BI(mLdn)F		1	1	3	3							2197.85	2198.86	2220.84	1099.93	733.62
	Hex13	1		13									2202.76	2203.76	2225.74	1102.39	735.26
	TR(trLdn)		1		6								2206.86	2207.87	2229.85	1104.44	736.63
	TR(dLdn), AG12345BS		1	1	5					1			2207.85	2208.85	2230.84	1104.93	736.96
	mF-BIBSF, mF-TRF-G		1	2	3	2							2213.85	2214.85	2236.83	1107.93	738.96
	mGal-mF-BIBSF-G		1	2	3	2							2213.85	2214.85	2236.83	1107.93	738.96
	mF-BIBSF, diF-BIBS		1	2	3	2							2213.85	2214.85	2236.83	1107.93	738.96
	mF-GnLnM4BSF		1	2	3	2							2213.85	2214.85	2236.83	1107.93	738.96
	mF-LdnM5BSF		1	2	3	2							2213.85	2214.85	2236.83	1107.93	738.96
	mF-GnLdnM5F		1	2	3	2							2213.85	2214.85	2236.83	1107.93	738.96
	trF-dLdnM2F	1		2	6	4							2222.88	2223.89	2245.87	1112.45	741.97
	TRF, mGa-BIBSF		1	3	3	1							2229.84	2230.85	2252.83	1115.93	744.29
	mF-TR, mGa-mF-BIBS		1	3	3	1							2229.84	2230.85	2252.83	1115.93	744.29
	GnLnM5BSF		1	3	3	1							2229.84	2230.85	2252.83	1115.93	744.29
	diLnM4BSF		1	3	3	1							2229.84	2230.85	2252.83	1115.93	744.29
	dF-AG123BSF		1		4	3							2238.88	2239.88	2261.87	1120.45	747.30
	dF-LdnAG1BSF		1		4	3							2238.88	2239.88	2261.87	1120.45	747.30
	dF-BI(dLdn)F		1		4	3							2238.88	2239.88	2261.87	1120.45	747.30
	Ac-Hex13	1		13						1			2244.77	2245.77	2267.76	1123.39	749.26
	mGa-TR, dGa-BIBS		1	4	3								2245.84	2246.84	2268.82	1123.92	749.62
	dLnM5BS		1	4	3								2245.84	2246.84	2268.82	1123.92	749.62
	mF-TRBSF-2G		1	1	4	2							2254.87	2255.88	2277.86	1128.44	752.63
	mFBI(m-Ldn)BSF		1	1	4	2							2254.87	2255.88	2277.86	1128.44	752.63
	mF-GnLdnM4BSF		1	1	4	2							2254.87	2255.88	2277.86	1128.44	752.63
	TRBSF-G		1	2	4	1							2270.87	2271.87	2293.86	1136.44	757.96
	TR(mLdn)F		1	2	4	1							2270.87	2271.87	2293.86	1136.44	757.96
	GnLdnM5BSF		1	2	4	1							2270.87	2271.87	2293.86	1136.44	757.96
	G2M9		1	8									2284.81	2285.82	2307.80	1143.41	762.61
	TRBS, TE-G		1	3	4	0							2286.86	2287.87	2309.85	1144.44	763.29
	mF-AG1234BSF		1		5	2							2295.90	2296.91	2318.89	1148.96	766.31
	mF-dLdnM3BSF		1		5	2							2295.90	2296.91	2318.89	1148.96	766.31
	TR(dLdn)F		1	1	5	1							2311.89	2312.90	2334.88	1156.95	771.64
	mGal-diF-BIF		1	3	2	3							2318.88	2319.88	2341.87	1160.45	773.97
	Ac-G2M9		1	8						1			2326.82	2327.83	2349.81	1164.42	776.61
	dGa-mF-BIF, dGa-dF-BI		1	4	2	2							2334.87	2335.88	2357.86	1168.44	779.30
	mF-diLnM5F		1	4	2	2							2334.87	2335.88	2357.86	1168.44	779.30
	trF-TRF-2G		1	1	3	4							2343.91	2344.92	2366.90	1172.96	782.31
	trF-LdnMOF		1	1	3	4							2343.91	2344.92	2366.90	1172.96	782.31
	TR(trLdn)F		1		6	1							2352.92	2353.93	2375.91	1177.47	785.31
	dF-BIBSF		1	2	3	3							2359.90	2360.91	2382.89	1180.96	787.64
	dF-TRF-G		1	2	3	3							2359.90	2360.91	2382.89	1180.96	787.64
	diF-BIBSF		1	2	3	3							2359.90	2360.91	2382.89	1180.96	787.64
	Hex14	1		14									2364.81	2365.82	2387.80	1183.41	789.28
	mF-TRF, dF-TR		1	3	3	2							2375.90	2376.91	2398.89	1188.96	792.97
	mGa-dF-BIBS		1	3	3	2							2375.90	2376.91	2398.89	1188.96	792.97
	mGa-mF-BIBSF		1	3	3	2							2375.90	2376.91	2398.89	1188.96	792.97
	mF-GnLnM5BSF		1	3	3	2							2375.90	2376.91	2398.89	1188.96	792.97
	mF-dLnM4BSF		1	3	3	2							2375.90	2376.91	2398.89	1188.96	792.97
	Ac-dG-dF-BI		1	4	2	2				1			2376.88	2377.89	2399.87	1189.45	793.30
	trF-AG123BSF		1		4	4							2384.94	2385.94	2407.92	1193.47	795.99
	trF-dLdnM3F		1		4	4							2384.94	2385.94	2407.92	1193.47	795.99
	mGa-TRF, mGa-mF-TR		1	4	3	1							2391.89	2392.90	2414.88	1196.95	798.31
	dGa-BIBSF		1	4	3	1							2391.89	2392.90	2414.88	1196.95	798.31
	dGa-mF-BIBS		1	4	3	1							2391.89	2392.90	2414.88	1196.95	798.31
	dLnM5BSF		1	4	3	1							2391.89	2392.90	2414.88	1196.95	798.31
	H7HN5F1		1	4	3	1							2391.89	2392.90	2414.88	1196.95	798.31
	dF-TRBSF-2G		1	1	4	3							2400.93	2401.94	2423.92	1201.47	801.32
	dF-BI(mLdn)BSF		1	1	4	3							2400.93	2401.94	2423.92	1201.47	801.32
	Ac-Hex14	1		14						1			2406.82	2407.83	2429.81	1204.42	803.28
	dGa-TR		1	5	3								2407.89	2408.90	2430.88	1204.95	803.64
	TR(trLdn)BS		1		7								2409.94	2410.95	2432.93	1205.98	804.32
	mF-TRBSF-G		1	2	4	2							2416.92	2417.93	2439.91	1209.47	806.65
	mF-TR(mLdn)F		1	2	4	2							2416.92	2417.93	2439.91	1209.47	806.65
	mF-GnLdnM5BSF		1	2	4	2							2416.92	2417.93	2439.91	1209.47	806.65
	TRBSF, mF-TRBS		1	3	4	1							2432.92	2433.93	2455.91	1217.47	811.98
	dF-AG1234BSF		1		5	3							2441.96	2442.96	2464.95	1221.99	814.99
	dF-dLdnM3BSF		1		5	3							2441.96	2442.96	2464.95	1221.99	814.99
	G3M9		1	9									2446.86	2447.87	2469.85	1224.44	816.63
	TE, mGa-TRBS		1	4	4	0							2448.91	2449.92	2471.90	1225.46	817.31
	dLn-LdnM5A		1	4	4								2448.91	2449.92	2471.90	1225.46	817.31
	mF-TR(dLdn)F		1	1	5	2							2457.95	2458.96	2480.94	1229.98	820.32
	H5HN7F1		1	2	5	1							2473.95	2474.95	2496.94	1237.98	825.66
	diGal-diF-BIF		1	4	2	3							2480.93	2481.94	2503.92	1241.47	827.98
	Ac-G3M9		1	9						1			2488.87	2489.88	2511.86	1245.44	830.63

	mF-trLdnM3F		1		6	2							2498.98	2499.99	2521.97	1250.50	834.00
	trF-TRF-G		1	2	3	4							2505.96	2506.97	2528.95	1253.99	836.33
	trF-TR, dF-TRF		1	3	3	3							2521.96	2522.96	2544.95	1261.99	841.66
	mGal-diF-BIBSF		1	3	3	3							2521.96	2522.96	2544.95	1261.99	841.66
	Hex15	1		15									2526.86	2527.87	2549.85	1264.44	843.29
	mGa-dF-TR		1	4	3	2							2537.95	2538.96	2560.94	1269.98	846.99
	mGa-mF-TRF		1	4	3	2							2537.95	2538.96	2560.94	1269.98	846.99
	dGa-dF-BIBS		1	4	3	2							2537.95	2538.96	2560.94	1269.98	846.99
	dGa-mF-BIBSF		1	4	3	2							2537.95	2538.96	2560.94	1269.98	846.99
	mF-dLnM5BSF		1	4	3	2							2537.95	2538.96	2560.94	1269.98	846.99
	trF-TRBSF-2G		1	1	4	4							2546.99	2548.00	2569.98	1274.50	850.00
	trF-BI(mLdn)BSF		1	1	4	4							2546.99	2548.00	2569.98	1274.50	850.00
	dGa-TRF, dGa-mF-TR		1	5	3	1							2553.95	2554.95	2576.94	1277.98	852.32
	trLdnM3BSF		1		7	1							2556.00	2557.01	2578.99	1279.01	853.01
	dF-TRBSF-G		1	2	4	3							2562.98	2563.99	2585.97	1282.50	855.33
	dF-LdnBIF		1	2	4	3							2562.98	2563.99	2585.97	1282.50	855.33
	Ac-Hex15	1		15						1			2568.87	2569.88	2591.86	1285.44	857.30
	triGal-TR		1	6	3								2569.94	2570.95	2592.93	1285.98	857.65
	mF-TRBSF, diF-TRBS		1	3	4	2							2578.98	2579.99	2601.97	1290.50	860.67
	trF-AG1234BSF		1		5	4							2588.01	2589.02	2611.00	1295.01	863.68
	trF-dLdnM3BSF		1		5	4							2588.01	2589.02	2611.00	1295.01	863.68
	TEF		1	4	4	1							2594.97	2595.98	2617.96	1298.49	866.00
	mGa-TRBSF		1	4	4	1							2594.97	2595.98	2617.96	1298.49	866.00
	mGa-mF-TRBS		1	4	4	1							2594.97	2595.98	2617.96	1298.49	866.00
	mF-dLn-LdnM5A		1	4	4	1							2594.97	2595.98	2617.96	1298.49	866.00
	dF-dLdnMOF		1	1	5	3							2604.01	2605.02	2627.00	1303.01	869.01
	diGal-TRBS		1	5	4								2610.97	2611.97	2633.96	1306.49	871.33
	teLdnM3		1		8								2613.02	2614.03	2636.01	1307.52	872.01
	H5HN7F2		1	2	5	2							2620.00	2621.01	2642.99	1311.01	874.34
	dF-trLdnM3F		1		6	3							2645.04	2646.04	2668.03	1323.53	882.69
	TEBS		1	4	5	0							2651.99	2653.00	2674.98	1327.00	885.01
	trF-TRF		1	3	3	4							2668.01	2669.02	2691.00	1335.01	890.35
	mGa-trF-TR		1	4	3	3							2684.01	2685.02	2707.00	1343.01	895.68
	mGa-dF-TRF		1	4	3	3							2684.01	2685.02	2707.00	1343.01	895.68
	dGa-dF-BIBSF		1	4	3	3							2684.01	2685.02	2707.00	1343.01	895.68
	Hex16	1		16									2688.91	2689.92	2711.90	1345.46	897.31
	dGa-dF-TR		1	5	3	2							2700.00	2701.01	2722.99	1351.01	901.01
	dGa-mF-TRF		1	5	3	2							2700.00	2701.01	2722.99	1351.01	901.01
	mF-trLdnM3BSF		1		7	2							2702.06	2703.06	2725.05	1352.04	901.69
	trF-TRBSF-G		1	2	4	4							2709.04	2710.05	2732.03	1355.53	904.02
	trF-LdnBIF		1	2	4	4							2709.04	2710.05	2732.03	1355.53	904.02
	triGal-TRF		1	6	3	1							2716.00	2717.01	2738.99	1359.01	906.34
	triGal-mF-TR		1	6	3	1							2716.00	2717.01	2738.99	1359.01	906.34
	dF-TRBSF		1	3	4	3							2725.04	2726.04	2748.02	1363.53	909.35
	trF-TRBS		1	3	4	3							2725.04	2726.04	2748.02	1363.53	909.35
	teF-AG1234BSF		1		5	5							2734.07	2735.08	2757.06	1368.04	912.36
	mF-TEF		1	4	4	2							2741.03	2742.04	2764.02	1371.52	914.68
	mGa-dF-TRBS		1	4	4	2							2741.03	2742.04	2764.02	1371.52	914.68
	mGa-mF-TRBSF		1	4	4	2							2741.03	2742.04	2764.02	1371.52	914.68
	trF-dLdnMOF		1	1	5	4							2750.07	2751.07	2773.06	1376.04	917.70
	dGa-TRBSF		1	5	4	1							2757.03	2758.03	2780.01	1379.52	920.02
	dGa-mF-TRBS		1	5	4	1							2757.03	2758.03	2780.01	1379.52	920.02
	teLdnM3F		1		8	1							2759.08	2760.09	2782.07	1380.55	920.70
	dGa-TE, trGa-TRBS		1	6	4								2773.02	2774.03	2796.01	1387.52	925.35
	trF-trLdnM3F		1		6	4							2791.09	2792.10	2814.08	1396.55	931.37
	TEBSF		1	4	5	1							2798.05	2799.06	2821.04	1400.03	933.69
	PE		1	5	5								2814.05	2815.05	2837.04	1408.03	939.02
	teLdnM3BS		1		9								2816.10	2817.11	2839.09	1409.06	939.71
	H5Hn8F2		1	2	6	2							2823.08	2824.09	2846.07	1412.55	942.04
	mGa-trF-TRF		1	4	3	4							2830.07	2831.07	2853.06	1416.04	944.36
	dGa-trF-TR		1	5	3	3							2846.06	2847.07	2869.05	1424.04	949.69
	dGa-dF-TRF		1	5	3	3							2846.06	2847.07	2869.05	1424.04	949.69
	dF-trLdnM3BSF		1		7	3							2848.12	2849.12	2871.10	1425.06	950.38
	Hex17	1		17									2850.97	2851.97	2873.96	1426.49	951.33
	triGal-diF-TR		1	6	3	2							2862.06	2863.06	2885.05	1432.04	955.03
	triGal-mF-TRF		1	6	3	2							2862.06	2863.06	2885.05	1432.04	955.03
	trF-TRBSF		1	3	4	4							2871.09	2872.10	2894.08	1436.55	958.04
	dF-TEF		1	4	4	3							2887.09	2888.10	2910.08	1444.55	963.37
	mGal-triF-TRBS		1	4	4	3							2887.09	2888.10	2910.08	1444.55	963.37
	mGal-diF-TRBSF		1	4	4	3							2887.09	2888.10	2910.08	1444.55	963.37
	diGal-diF-TRBS		1	5	4	2							2903.08	2904.09	2926.07	1452.55	968.70
	diGal-mF-TRBSF		1	5	4	2							2903.08	2904.09	2926.07	1452.55	968.70
	mF-teLdnM3F		1		8	2							2905.14	2906.14	2928.13	1453.58	969.39
	triGal-TRBSF		1	6	4	1							2919.08	2920.09	2942.07	1460.55	974.03
	triGal-mF-TRBS		1	6	4	1							2919.08	2920.09	2942.07	1460.55	974.03
	mF-TEBSF		1	4	5	2							2944.11	2945.12	2967.10	1473.06	982.38

	PEF		1	5	5	1							2960.10	**2961.11**	**2983.09**	**1481.06**	**987.71**
	teLdnM3BSF		1		9	1							2962.16	**2963.17**	**2985.15**	**1482.09**	**988.39**
	diGal-triF-TRF		1	5	3	4							2992.12	**2993.13**	**3015.11**	**1497.07**	**998.38**
	trF-trLdnM3BSF		1		7	4							2994.17	**2995.18**	**3017.16**	**1498.09**	**999.07**
	mFdLn-dLdnM5A		1	4	6	1							3001.13	**3002.14**	**3024.12**	**1501.57**	**1001.38**
	triGal-triF-TR		1	6	3	3							3008.11	**3009.12**	**3031.10**	**1505.06**	**1003.71**
	triGal-diF-TRF		1	6	3	3							3008.11	**3009.12**	**3031.10**	**1505.06**	**1003.71**
	Hex18	1		18									3013.02	**3014.03**	**3036.01**	**1507.52**	**1005.35**
	PEBS		1	5	6								3017.13	**3018.13**	**3040.12**	**1509.57**	**1006.72**
	mGal-triF-TRBSF		1	4	4	4							3033.15	**3034.15**	**3056.14**	**1517.58**	**1012.06**
	diGal-triF-TRBS		1	5	4	3							3049.14	**3050.15**	**3072.13**	**1525.58**	**1017.39**
	diGal-diF-TRBSF		1	5	4	3							3049.14	**3050.15**	**3072.13**	**1525.58**	**1017.39**
	dF-teLdnM3F		1		8	3							3051.19	**3052.20**	**3074.18**	**1526.60**	**1018.07**
	triGal-diF-TRBS		1	6	4	2							3065.14	**3066.14**	**3088.13**	**1533.58**	**1022.72**
	triGal-mF-TRBSF		1	6	4	2							3065.14	**3066.14**	**3088.13**	**1533.58**	**1022.72**
	dF-TEBSF		1	4	5	3							3090.17	**3091.18**	**3113.16**	**1546.09**	**1031.06**
	mF-teLdnM3BSF		1		9	2							3108.22	**3109.22**	**3131.21**	**1555.12**	**1037.08**
	dFdLn-dLdnM5A		1	4	6	2							3147.19	**3148.20**	**3170.18**	**1574.60**	**1050.07**
	triGal-triF-TRF		1	6	3	4							3154.17	**3155.18**	**3177.16**	**1578.09**	**1052.40**
	PEBSF		1	5	6	1							3163.18	**3164.19**	**3186.17**	**1582.60**	**1055.40**
	Hex19	1		19									3175.07	**3176.08**	**3198.06**	**1588.54**	**1059.36**
	diGal-triF-TRBSF		1	5	4	4							3195.20	**3196.21**	**3218.19**	**1598.61**	**1066.07**
	trF-teLdnM3F		1		8	4							3197.25	**3198.26**	**3220.24**	**1599.63**	**1066.76**
	triGal-triF-TRBS		1	6	4	3							3211.19	**3212.20**	**3234.18**	**1606.60**	**1071.41**
	triGal-diF-TRBSF		1	6	4	3							3211.19	**3212.20**	**3234.18**	**1606.60**	**1071.41**
	dF-teLdnM3BSF		1		9	3							3254.27	**3255.28**	**3277.26**	**1628.14**	**1085.77**
	Hex20	1		20									3337.13	**3338.13**	**3360.11**	**1669.57**	**1113.38**
	triGal-triF-TRBSF		1	6	4	4							3357.25	**3358.26**	**3380.24**	**1679.63**	**1120.09**
	trF-teLdnM3BSF		1		9	4							3400.33	**3401.34**	**3423.32**	**1701.17**	**1134.45**
	H9Hn9F1		1	6	7	1							3528.32	**3529.32**	**3551.31**	**1765.17**	**1177.11**

monosialo (mN) glycans		reducing end		non-reducing end				modification					*m/z*			
		PA	M3PA	Hex	HexNAc	dHex	NANA	Me	Ac	HPO_3	SO_3	MS number	**+H**	**+Na**	**+2H**	**+3H**
mN	Gal	1		1			1					549.22	**550.22**	**572.21**	**275.62**	**184.08**
mN	Tn	1			1		1					590.24	**591.25**	**613.23**	**296.13**	**197.76**
mN	Gn-Man	1		1	1		1					752.30	**753.30**	**775.29**	**377.16**	**251.77**
mN	core1	1		1	1		1					752.30	**753.30**	**775.29**	**377.16**	**251.77**
mN	Ga-Gn-F	1		1	1	1	1					898.35	**899.36**	**921.34**	**450.18**	**300.46**
mN	Ln-Man, G-core1	1		2	1		1					914.35	**915.36**	**937.34**	**458.18**	**305.79**
mN	core2, dGn-Man	1		1	2		1					955.38	**956.38**	**978.36**	**478.70**	**319.47**
mN	mF-G-core1	1		2	1	1	1					1060.41	**1061.41**	**1083.40**	**531.21**	**354.48**
mN	mF-Gan-G-core1	1		1	2	1	1					1101.43	**1102.44**	**1124.42**	**551.72**	**368.15**
mN	GnLn-Man	1		2	2		1					1117.43	**1118.44**	**1140.42**	**559.72**	**373.48**
mN	Gan-Ga-core1	1		2	2		1					1117.43	**1118.44**	**1140.42**	**559.72**	**373.48**
mN	G-core2	1		2	2		1					1117.43	**1118.44**	**1140.42**	**559.72**	**373.48**
mN	mF-Gan-G-core1	1		2	2	1	1					1263.49	**1264.49**	**1286.48**	**632.75**	**422.17**
mGnP	M3		1		1					1		1271.43	**1272.44**	**1294.42**	**636.72**	**424.82**
mN	dLn-Man	1		3	2		1					1279.48	**1280.49**	**1302.47**	**640.75**	**427.50**
mN	M1BSF	1		1	3	1	1					1304.51	**1305.52**	**1327.50**	**653.26**	**435.84**
mN	GnM2	1		2	3		1					1320.51	**1321.52**	**1343.50**	**661.26**	**441.18**
mGnP	M4		1	1	1					1		1433.48	**1434.49**	**1456.47**	**717.75**	**478.84**
mN	M1BSdF	1		1	3	2	1					1450.57	**1451.58**	**1473.56**	**726.29**	**484.53**
mN	GnM2F	1		2	3	1	1					1466.57	**1467.57**	**1489.56**	**734.29**	**489.86**
mN	LnM2, AG1	1		3	3		1					1482.56	**1483.57**	**1505.55**	**742.29**	**495.19**
mN	GnM2BS	1		2	4		1					1523.59	**1524.59**	**1546.58**	**762.80**	**508.87**
mN	LdnM2	1		2	4		1					1523.59	**1524.59**	**1546.58**	**762.80**	**508.87**
mN	Ac-AG1		1		1		1		1			1524.57	**1525.58**	**1547.56**	**763.29**	**509.20**
mN	GnM2BSF			2	4	1	1					1573.58	**1574.58**	**1596.57**	**787.80**	**525.53**
mGnP	M5		1	2	1					1		1595.54	**1596.54**	**1618.53**	**798.78**	**532.85**
mN	mF-GnM2F	1		2	3	2	1					1612.62	**1613.63**	**1635.61**	**807.32**	**538.55**
mN	LnM2F, AG1F		1		1	1	1					1628.62	**1629.63**	**1651.61**	**815.32**	**543.88**
mN	GnM4		1	1	1	0	1					1644.61	**1645.62**	**1667.60**	**823.31**	**549.21**
mN	MO		1	1	1	0	1					1644.61	**1645.62**	**1667.60**	**823.31**	**549.21**
mN	LdnM2F	1		2	4	1	1					1669.65	**1670.65**	**1692.63**	**835.83**	**557.56**
mN	LdnM3, AG12, AG1BS		1		2		1					1685.64	**1686.65**	**1708.63**	**843.83**	**562.89**
mN	LnM2BS	1		3	4		1					1685.64	**1686.65**	**1708.63**	**843.83**	**562.89**
mN	mF-GnM2BSF			2	4	2	1					1719.63	**1720.64**	**1742.62**	**860.82**	**574.22**
mN	LdnM2BS	1		2	5		1					1726.67	**1727.67**	**1749.66**	**864.34**	**576.56**
mN	GnLdnM2	1		2	5		1					1726.67	**1727.67**	**1749.66**	**864.34**	**576.56**
mN	Ac-AG12		1		2		1		1			1727.65	**1728.66**	**1750.64**	**864.83**	**576.89**
mGnP	M6		1	3	1					1		1757.59	**1758.60**	**1780.58**	**879.80**	**586.87**
mN	dF-GnM2F	1		2	3	3	1					1758.68	**1759.69**	**1781.67**	**880.35**	**587.23**
mN	mF-AG1F		1		1	2	1					1774.68	**1775.68**	**1797.67**	**888.35**	**592.57**
mN	mF-LnM2F	1		3	3	2	1					1774.68	**1775.68**	**1797.67**	**888.35**	**592.57**
mN	MOF, GnM4F		1	1	1	1	1					1790.67	**1791.68**	**1813.66**	**896.34**	**597.90**
mN	mGal-MO		1	2	1		1					1806.67	**1807.67**	**1829.66**	**904.34**	**603.23**
mN	LnM4		1	2	1	0	1					1806.67	**1807.67**	**1829.66**	**904.34**	**603.23**
mN	GnM5		1	2	1	0	1					1806.67	**1807.67**	**1829.66**	**904.34**	**603.23**
mN	mF-LdnF	1		2	4	2	1					1815.70	**1816.71**	**1838.69**	**908.86**	**606.24**
mN	AG12F, AG1BSF		1		2	1	1					1831.70	**1832.71**	**1854.69**	**916.86**	**611.57**
mN	LdnM3F		1		2	1	1					1831.70	**1832.71**	**1854.69**	**916.86**	**611.57**
mN	LnM2BSF	1		3	4	1	1					1831.70	**1832.71**	**1854.69**	**916.86**	**611.57**
mN	BI-Ga, MOBS, LdnM4		1	1	2	0	1					1847.69	**1848.70**	**1870.68**	**924.85**	**616.90**
mN	GnM4BS		1	1	2	0	1					1847.69	**1848.70**	**1870.68**	**924.85**	**616.90**
mN	LdnM4		1	1	2		1					1847.69	**1848.70**	**1870.68**	**924.85**	**616.90**
mN	dF-GnM2BSF			2	4	3	1					1865.69	**1866.70**	**1888.68**	**933.85**	**622.90**
mN	LdnM2BSF	1		2	5	1	1					1872.72	**1873.73**	**1895.71**	**937.37**	**625.25**
mN	GnLdnM2F	1		2	5	1	1					1872.72	**1873.73**	**1895.71**	**937.37**	**625.25**
mN	AG123, AG12BS		1		3		1					1888.72	**1889.73**	**1911.71**	**945.37**	**630.58**
mN	GnLdnM3, LdnM3BS		1		3		1					1888.72	**1889.73**	**1911.71**	**945.37**	**630.58**
mN	Ac-BI-Ga		1	1	2		1		1			1889.70	**1890.71**	**1912.69**	**945.86**	**630.91**
mGnP	M7		1	4	1					1		1919.64	**1920.65**	**1942.63**	**960.83**	**640.89**
mN	dF-AG1F		1		1	3	1					1920.73	**1921.74**	**1943.72**	**961.37**	**641.25**
mN	dLdnM2	1		2	6		1					1929.75	**1930.75**	**1952.74**	**965.88**	**644.26**
mN	mF-MOF, mF-GnM4F		1	1	1	2	1					1936.73	**1937.74**	**1959.72**	**969.37**	**646.58**
mN	mGal-MOF		1	2	1	1	1					1952.72	**1953.73**	**1975.71**	**977.37**	**651.92**
mN	LnM4F, GnM5F		1	2	1	1	1					1952.72	**1953.73**	**1975.71**	**977.37**	**651.92**
mN	LnM5, GnM6		1	3	1	0	1					1968.72	**1969.73**	**1991.71**	**985.37**	**657.25**
mN	mF-AG12F, mF-AG1BSF		1		2	2	1					1977.76	**1978.76**	**2000.75**	**989.89**	**660.26**
mN	mF-LnM2BSF	1		3	4	2	1					1977.76	**1978.76**	**2000.75**	**989.89**	**660.26**
mN	mF-LdnM3F		1		2	2	1					1977.76	**1978.76**	**2000.75**	**989.89**	**660.26**
mN	BIF-Ga, MOBSF		1	1	2	1	1					1993.75	**1994.76**	**2016.74**	**997.88**	**665.59**
mN	GnM4BSF		1	1	2	1	1					1993.75	**1994.76**	**2016.74**	**997.88**	**665.59**
mN	LdnM4F		1	1	2	1	1					1993.75	**1994.76**	**2016.74**	**997.88**	**665.59**

mN	BI		1	2	2	0	1					2009.75	**2010.75**	**2032.73**	**1005.88**	**670.92**
mN	mGal-MOBS		1	2	2		1					2009.75	**2010.75**	**2032.73**	**1005.88**	**670.92**
mN	mGal-BI-G		1	2	2		1					2009.75	**2010.75**	**2032.73**	**1005.88**	**670.92**
mN	LnM4BS, GnM5BS		1	2	2	0	1					2009.75	**2010.75**	**2032.73**	**1005.88**	**670.92**
mN	LdnM5, GnLnM4		1	2	2		1					2009.75	**2010.75**	**2032.73**	**1005.88**	**670.92**
mN	Ac1-LnM5		1	3	1		1		1			2010.73	**2011.74**	**2033.72**	**1006.37**	**671.25**
mN	mF-LdnBSF	1		2	5	2	1					2018.78	**2019.79**	**2041.77**	**1010.40**	**673.93**
mN	mF-GnLdnM2F	1		2	5	2	1					2018.78	**2019.79**	**2041.77**	**1010.40**	**673.93**
mN	AG12BSF, AG123F		1		3	1	1					2034.78	**2035.78**	**2057.77**	**1018.40**	**679.27**
mN	GnLdnM3F, LdnM3BSF		1		3	1	1					2034.78	**2035.78**	**2057.77**	**1018.40**	**679.27**
mN	BIBS-G, TR-2G		1	1	3	0	1					2050.77	**2051.78**	**2073.76**	**1026.39**	**684.60**
mN	BI(mLdn), LdnM4BS		1	1	3		1					2050.77	**2051.78**	**2073.76**	**1026.39**	**684.60**
mN	GnLdnM4		1	1	3		1					2050.77	**2051.78**	**2073.76**	**1026.39**	**684.60**
mN	Ac-BI		1	2	2		1		1			2051.76	**2052.76**	**2074.75**	**1026.89**	**684.93**
mN	dLdnM2F	1		2	6	1	1					2075.80	**2076.81**	**2098.79**	**1038.91**	**692.94**
mGnP	M8		1	5	1					1		2081.70	**2082.70**	**2104.69**	**1041.86**	**694.91**
mN	GnLdnM3BS, AG123BS		1		4		1					2091.80	**2092.81**	**2114.79**	**1046.91**	**698.27**
mN	BI(dLdn)		1		4		1					2091.80	**2092.81**	**2114.79**	**1046.91**	**698.27**
mN	mGal-mF-MOF		1	2	1	2	1					2098.78	**2099.79**	**2121.77**	**1050.40**	**700.60**
mN	mF-LnM4F		1	2	1	2	1					2098.78	**2099.79**	**2121.77**	**1050.40**	**700.60**
mN	mF-GnM5F		1	2	1	2	1					2098.78	**2099.79**	**2121.77**	**1050.40**	**700.60**
mN	LnM5F		1	3	1	1	1					2114.78	**2115.78**	**2137.77**	**1058.40**	**705.93**
mN	dF-AG1BSF		1		2	3	1					2123.81	**2124.82**	**2146.80**	**1062.91**	**708.95**
mN	dF-AG12F		1		2	3	1					2123.81	**2124.82**	**2146.80**	**1062.91**	**708.95**
mN	mG-LnM5		1	4	1	0	1					2130.77	**2131.78**	**2153.76**	**1066.39**	**711.26**
mN	mF-MOBSF		1	1	2	2	1					2139.81	**2140.82**	**2162.80**	**1070.91**	**714.28**
mN	mF-BIF-G		1	1	2	2	1					2139.81	**2140.82**	**2162.80**	**1070.91**	**714.28**
mN	mF-GnM4BSF		1	1	2	2	1					2139.81	**2140.82**	**2162.80**	**1070.91**	**714.28**
mN	mF-LdnM4F		1	1	2	2	1					2139.81	**2140.82**	**2162.80**	**1070.91**	**714.28**
mN	BIF, mF-BI		1	2	2	1	1					2155.80	**2156.81**	**2178.79**	**1078.91**	**719.61**
mN	mGa-MOBSF		1	2	2	1	1					2155.80	**2156.81**	**2178.79**	**1078.91**	**719.61**
mN	mGa-BIF-G		1	2	2	1	1					2155.80	**2156.81**	**2178.79**	**1078.91**	**719.61**
mN	mGa-mF-MOBS		1	2	2	1	1					2155.80	**2156.81**	**2178.79**	**1078.91**	**719.61**
mN	LnM4BSF		1	2	2	1	1					2155.80	**2156.81**	**2178.79**	**1078.91**	**719.61**
mN	GnM5BSF		1	2	2	1	1					2155.80	**2156.81**	**2178.79**	**1078.91**	**719.61**
mN	GnLnM4F		1	2	2	1	1					2155.80	**2156.81**	**2178.79**	**1078.91**	**719.61**
mN	LdnM5F		1	2	2	1	1					2155.80	**2156.81**	**2178.79**	**1078.91**	**719.61**
mN	dF-GnLdnM2F	1		2	5	3	1					2164.84	**2165.85**	**2187.83**	**1083.43**	**722.62**
mN	mGa-BI		1	3	2		1					2171.80	**2172.81**	**2194.79**	**1086.91**	**724.94**
mN	LnM5BS		1	3	2		1					2171.80	**2172.81**	**2194.79**	**1086.91**	**724.94**
mN	GnLnM5		1	3	2		1					2171.80	**2172.81**	**2194.79**	**1086.91**	**724.94**
mN	dLnM4		1	3	2		1					2171.80	**2172.81**	**2194.79**	**1086.91**	**724.94**
mN	mF-AG12BSF		1		3	2	1					2180.84	**2181.84**	**2203.82**	**1091.42**	**727.95**
mN	mF-AG123F		1		3	2	1					2180.84	**2181.84**	**2203.82**	**1091.42**	**727.95**
mN	mF-LdnM3BSF		1		3	2	1					2180.84	**2181.84**	**2203.82**	**1091.42**	**727.95**
mN	mF-LdnAG1F		1		3	2	1					2180.84	**2181.84**	**2203.82**	**1091.42**	**727.95**
mN	BIBSF-Ga		1	1	3	1	1					2196.83	**2197.84**	**2219.82**	**1099.42**	**733.28**
mN	TRF-2Ga		1	1	3	1	1					2196.83	**2197.84**	**2219.82**	**1099.42**	**733.28**
mN	BI(mLdn)F		1	1	3	1	1					2196.83	**2197.84**	**2219.82**	**1099.42**	**733.28**
mN	LdnM4BSF		1	1	3	1	1					2196.83	**2197.84**	**2219.82**	**1099.42**	**733.28**
mN	GnLdnM4F		1	1	3	1	1					2196.83	**2197.84**	**2219.82**	**1099.42**	**733.28**
mN	BIBS		1	2	3	0	1					2212.83	**2213.83**	**2235.81**	**1107.42**	**738.62**
mN	TR-Ga		1	2	3		1					2212.83	**2213.83**	**2235.81**	**1107.42**	**738.62**
mN	mGal-BIBS-Ga		1	2	3		1					2212.83	**2213.83**	**2235.81**	**1107.42**	**738.62**
mN	GnLnM4BS		1	2	3		1					2212.83	**2213.83**	**2235.81**	**1107.42**	**738.62**
mN	LdnM5BS		1	2	3		1					2212.83	**2213.83**	**2235.81**	**1107.42**	**738.62**
mN	GnLdnM5		1	2	3		1					2212.83	**2213.83**	**2235.81**	**1107.42**	**738.62**
mN	mF-dLdnM2F	1		2	6	2	1					2221.86	**2222.87**	**2244.85**	**1111.94**	**741.63**
mN	AG123BSF		1		4	1	1					2237.86	**2238.86**	**2260.85**	**1119.94**	**746.96**
mN	LdnAG1BSF		1		4	1	1					2237.86	**2238.86**	**2260.85**	**1119.94**	**746.96**
mN	BI(dLdn)F		1		4	1	1					2237.86	**2238.86**	**2260.85**	**1119.94**	**746.96**
mGnP	M9		1	6	1					1		2243.75	**2244.76**	**2266.74**	**1122.88**	**748.92**
mN	TRBS-2Ga		1	1	4		1					2253.85	**2254.86**	**2276.84**	**1127.93**	**752.29**
mN	BI(mLdn)BS		1	1	4		1					2253.85	**2254.86**	**2276.84**	**1127.93**	**752.29**
mN	GnLdnM4BS		1	1	4		1					2253.85	**2254.86**	**2276.84**	**1127.93**	**752.29**
mN	mF-LnM5F		1	3	1	2	1					2260.84	**2261.84**	**2283.82**	**1131.42**	**754.62**
mN	trF-AG12F		1		2	4	1					2269.87	**2270.88**	**2292.86**	**1135.94**	**757.63**
mN	mG-LnM5F		1	4	1	1	1					2276.83	**2277.84**	**2299.82**	**1139.42**	**759.95**
mN	dF-BIF-Ga		1	1	2	3	1					2285.87	**2286.87**	**2308.86**	**1143.94**	**762.96**
mN	AG1234BS		1		5		1					2294.88	**2295.89**	**2317.87**	**1148.45**	**765.97**
mN	BI(dLdn)BS		1		5		1					2294.88	**2295.89**	**2317.87**	**1148.45**	**765.97**
mN	mF-BIF, diF-BI		1	2	2	2	1					2301.86	**2302.87**	**2324.85**	**1151.94**	**768.29**
mN	mGa-mF-MOBSF		1	2	2	2	1					2301.86	**2302.87**	**2324.85**	**1151.94**	**768.29**

mN	mGa-mF-BIF-Ga		1	2	2	2	1					2301.86	**2302.87**	**2324.85**	**1151.94**	**768.29**
mN	mF-LnM4BSF		1	2	2	2	1					2301.86	**2302.87**	**2324.85**	**1151.94**	**768.29**
mN	mF-GnM5BSF		1	2	2	2	1					2301.86	**2302.87**	**2324.85**	**1151.94**	**768.29**
mN	mF-GnLnM4F		1	2	2	2	1					2301.86	**2302.87**	**2324.85**	**1151.94**	**768.29**
mN	mF-LdnM5F		1	2	2	2	1					2301.86	**2302.87**	**2324.85**	**1151.94**	**768.29**
mN	trF-GnLdnM2F	1		2	5	4	1					2310.90	**2311.91**	**2333.89**	**1156.46**	**771.31**
mN	mGa-BIF, mGa-mF-BI		1	3	2	1	1					2317.86	**2318.86**	**2340.85**	**1159.94**	**773.63**
mN	LnM5BSF		1	3	2	1	1					2317.86	**2318.86**	**2340.85**	**1159.94**	**773.63**
mN	GnLnM5F, dLnM4F		1	3	2	1	1					2317.86	**2318.86**	**2340.85**	**1159.94**	**773.63**
mN	dF-AG12BSF		1		3	3	1					2326.89	**2327.90**	**2349.88**	**1164.45**	**776.64**
mN	dF-AG123F		1		3	3	1					2326.89	**2327.90**	**2349.88**	**1164.45**	**776.64**
mN	dF-GnLdnM3F		1		3	3	1					2326.89	**2327.90**	**2349.88**	**1164.45**	**776.64**
mN	dGa-BI		1	4	2		1					2333.85	**2334.86**	**2356.84**	**1167.93**	**778.96**
mN	mG-LnM5BS		1	4	2	0	1					2333.85	**2334.86**	**2356.84**	**1167.93**	**778.96**
mN	dLnM5		1	4	2		1					2333.85	**2334.86**	**2356.84**	**1167.93**	**778.96**
mN	mF-BIBSF-G		1	1	3	2	1					2342.89	**2343.90**	**2365.88**	**1172.45**	**781.97**
mN	mF-TRF-2G		1	1	3	2	1					2342.89	**2343.90**	**2365.88**	**1172.45**	**781.97**
mN	mF-BI(mLdn)F		1	1	3	2	1					2342.89	**2343.90**	**2365.88**	**1172.45**	**781.97**
mN	mF-LdnM4BSF		1	1	3	2	1					2342.89	**2343.90**	**2365.88**	**1172.45**	**781.97**
mN	mF-GnLdnM4F		1	1	3	2	1					2342.89	**2343.90**	**2365.88**	**1172.45**	**781.97**
mN	BIBSF, TRF-Ga		1	2	3	1	1					2358.88	**2359.89**	**2381.87**	**1180.45**	**787.30**
mN	mGa-BIBSF-Ga		1	2	3	1	1					2358.88	**2359.89**	**2381.87**	**1180.45**	**787.30**
mN	mGa-mF-BIBS-Ga		1	2	3	1	1					2358.88	**2359.89**	**2381.87**	**1180.45**	**787.30**
mN	mF-BIBS		1	2	3	1	1					2358.88	**2359.89**	**2381.87**	**1180.45**	**787.30**
mN	GnLnM4BSF		1	2	3	1	1					2358.88	**2359.89**	**2381.87**	**1180.45**	**787.30**
mN	LdnM5BSF		1	2	3	1	1					2358.88	**2359.89**	**2381.87**	**1180.45**	**787.30**
mN	GnLdnM5F		1	2	3	1	1					2358.88	**2359.89**	**2381.87**	**1180.45**	**787.30**
mN	dF-dLdnM2F	1		2	6	3	1					2367.92	**2368.93**	**2390.91**	**1184.97**	**790.31**
mN	TR		1	3	3		1					2374.88	**2375.89**	**2397.87**	**1188.45**	**792.63**
mN	mGa-BIBS		1	3	3		1					2374.88	**2375.89**	**2397.87**	**1188.45**	**792.63**
mN	GnLnM5BS		1	3	3		1					2374.88	**2375.89**	**2397.87**	**1188.45**	**792.63**
mN	dLnM4BS		1	3	3		1					2374.88	**2375.89**	**2397.87**	**1188.45**	**792.63**
mN	mF-AG123BSF		1		4	2	1					2383.91	**2384.92**	**2406.90**	**1192.96**	**795.65**
mN	mF-LdnAG1BSF		1		4	2	1					2383.91	**2384.92**	**2406.90**	**1192.96**	**795.65**
mN	mF-dLdnM3F		1		4	2	1					2383.91	**2384.92**	**2406.90**	**1192.96**	**795.65**
mN	TRBSF-2G		1	1	4	1	1					2399.91	**2400.92**	**2422.90**	**1200.96**	**800.98**
mN	BI(mLdn)BSF		1	1	4	1	1					2399.91	**2400.92**	**2422.90**	**1200.96**	**800.98**
mN	GnLdnM4BSF		1	1	4	1	1					2399.91	**2400.92**	**2422.90**	**1200.96**	**800.98**
mGnP	G1M9		1	7	1					1		2405.80	**2406.81**	**2428.79**	**1203.91**	**802.94**
mN	TRBS-G		1	2	4	0	1					2415.90	**2416.91**	**2438.89**	**1208.96**	**806.31**
mN	TR(mLdn)		1	2	4		1					2415.90	**2416.91**	**2438.89**	**1208.96**	**806.31**
mN	GnLdnM5BS		1	2	4		1					2415.90	**2416.91**	**2438.89**	**1208.96**	**806.31**
mN	AG1234BSF		1		5	1	1					2440.94	**2441.94**	**2463.93**	**1221.48**	**814.65**
mN	BI(dLdn)BSF		1		5	1	1					2440.94	**2441.94**	**2463.93**	**1221.48**	**814.65**
mN	dF-BIF		1	2	2	3	1					2447.92	**2448.93**	**2470.91**	**1224.97**	**816.98**
mN	TR(dLdn)		1	1	5		1					2456.93	**2457.94**	**2479.92**	**1229.47**	**819.98**
mN	mGa-dF-BI		1	3	2	2	1					2463.91	**2464.92**	**2486.90**	**1232.96**	**822.31**
mN	mGa-mF-BIF		1	3	2	2	1					2463.91	**2464.92**	**2486.90**	**1232.96**	**822.31**
mN	mF-LnM5BSF		1	3	2	2	1					2463.91	**2464.92**	**2486.90**	**1232.96**	**822.31**
mN	mF-GnLnM5F		1	3	2	2	1					2463.91	**2464.92**	**2486.90**	**1232.96**	**822.31**
mN	mF-dLnM4F		1	3	2	2	1					2463.91	**2464.92**	**2486.90**	**1232.96**	**822.31**
mN	trF-AG12BSF		1		3	4	1					2472.95	**2473.96**	**2495.94**	**1237.48**	**825.32**
mN	trF-AG123F		1		3	4	1					2472.95	**2473.96**	**2495.94**	**1237.48**	**825.32**
mN	trF-LdnAG1F		1		3	4	1					2472.95	**2473.96**	**2495.94**	**1237.48**	**825.32**
mN	dGa-BIF, dGa-mF-BI		1	4	2	1	1					2479.91	**2480.92**	**2502.90**	**1240.96**	**827.64**
mN	mGa-LnM5BSF		1	4	2	1	1					2479.91	**2480.92**	**2502.90**	**1240.96**	**827.64**
mN	dLnM5F		1	4	2	1	1					2479.91	**2480.92**	**2502.90**	**1240.96**	**827.64**
mN	dF-BIBSF-G		1	1	3	3	1					2488.95	**2489.95**	**2511.94**	**1245.48**	**830.66**
mN	dF-TRF-2G		1	1	3	3	1					2488.95	**2489.95**	**2511.94**	**1245.48**	**830.66**
mN	dF-BI(mLdn)F		1	1	3	3	1					2488.95	**2489.95**	**2511.94**	**1245.48**	**830.66**
mN	TR(trLdn)		1		6		1					2497.96	**2498.96**	**2520.95**	**1249.99**	**833.66**
mN	mF-BIBSF		1	2	3	2	1					2504.94	**2505.95**	**2527.93**	**1253.48**	**835.99**
mN	mF-TRF-G		1	2	3	2	1					2504.94	**2505.95**	**2527.93**	**1253.48**	**835.99**
mN	mGa-mF-BIBSF-G		1	2	3	2	1					2504.94	**2505.95**	**2527.93**	**1253.48**	**835.99**
mN	dF-BIBS, mF-BIBSF		1	2	3	2	1					2504.94	**2505.95**	**2527.93**	**1253.48**	**835.99**
mN	mF-GnLnM4BSF		1	2	3	2	1					2504.94	**2505.95**	**2527.93**	**1253.48**	**835.99**
mN	mF-LdnM5BSF		1	2	3	2	1					2504.94	**2505.95**	**2527.93**	**1253.48**	**835.99**
mN	mF-GnLdnM5F		1	2	3	2	1					2504.94	**2505.95**	**2527.93**	**1253.48**	**835.99**
mN	trF-dLdnM2F	1		2	6	4	1					2513.98	**2514.98**	**2536.97**	**1258.00**	**839.00**
mN	TRF, mF-TR		1	3	3	1	1					2520.94	**2521.94**	**2543.93**	**1261.48**	**841.32**
mN	mGa-BIBSF		1	3	3	1	1					2520.94	**2521.94**	**2543.93**	**1261.48**	**841.32**
mN	mGal-mF-BIBS		1	3	3	1	1					2520.94	**2521.94**	**2543.93**	**1261.48**	**841.32**
mN	GnLnM5BSF		1	3	3	1	1					2520.94	**2521.94**	**2543.93**	**1261.48**	**841.32**

mN	diLnM4BSF		1	3	3	1	1					2520.94	**2521.94**	**2543.93**	**1261.48**	**841.32**
mN	dF-AG123BSF		1		4	3	1					2529.97	**2530.98**	**2552.96**	**1265.99**	**844.33**
mN	dF-LdnAG1BSF		1		4	3	1					2529.97	**2530.98**	**2552.96**	**1265.99**	**844.33**
mN	dF-dLdnM3F		1		4	3	1					2529.97	**2530.98**	**2552.96**	**1265.99**	**844.33**
mN	mGa-TR, dGa-BIBS		1	4	3		1					2536.93	**2537.94**	**2559.92**	**1269.47**	**846.65**
mN	dLnM5BS		1	4	3		1					2536.93	**2537.94**	**2559.92**	**1269.47**	**846.65**
mN	mF-TRBSF-2G		1	1	4	2	1					2545.97	**2546.97**	**2568.96**	**1273.99**	**849.66**
mN	mF-LdnMOBSF		1	1	4	2	1					2545.97	**2546.97**	**2568.96**	**1273.99**	**849.66**
mN	mF-GnLdnM4BSF		1	1	4	2	1					2545.97	**2546.97**	**2568.96**	**1273.99**	**849.66**
mN	TRBSF-G		1	2	4	1	1					2561.96	**2562.97**	**2584.95**	**1281.99**	**854.99**
mN	TR(mLdn)F		1	2	4	1	1					2561.96	**2562.97**	**2584.95**	**1281.99**	**854.99**
mN	GnLdnM5BSF		1	2	4	1	1					2561.96	**2562.97**	**2584.95**	**1281.99**	**854.99**
mN	TRBS		1	3	4	0	1					2577.96	**2578.96**	**2600.95**	**1289.99**	**860.33**
mN	mF-AG1234BSF		1		5	2	1					2586.99	**2588.00**	**2609.98**	**1294.50**	**863.34**
mN	mF-dLdnM3BSF		1		5	2	1					2586.99	**2588.00**	**2609.98**	**1294.50**	**863.34**
mN	TR(dLdn)F		1	1	5	1	1					2602.99	**2604.00**	**2625.98**	**1302.50**	**868.67**
mN	mGa-dF-BIF		1	3	2	3	1					2609.97	**2610.98**	**2632.96**	**1305.99**	**871.00**
mN	dGa-dF-BI		1	4	2	2	1					2625.97	**2626.97**	**2648.96**	**1313.99**	**876.33**
mN	dGa-mF-BIF		1	4	2	2	1					2625.97	**2626.97**	**2648.96**	**1313.99**	**876.33**
mN	mF-diLnM5F		1	4	2	2	1					2625.97	**2626.97**	**2648.96**	**1313.99**	**876.33**
mN	Ac-dG-dF-BI		1	4	2	2	1					2625.97	**2626.97**	**2648.96**	**1313.99**	**876.33**
mN	trF-TRF-2G		1	1	3	4	1					2635.00	**2636.01**	**2657.99**	**1318.51**	**879.34**
mN	trF-LdnMOF		1	1	3	4	1					2635.00	**2636.01**	**2657.99**	**1318.51**	**879.34**
mN	TR(trLdn)F		1		6	1	1					2644.02	**2645.02**	**2667.00**	**1323.02**	**882.35**
mN	dF-BIBSF		1	2	3	3	1					2651.00	**2652.01**	**2673.99**	**1326.51**	**884.67**
mN	dF-TRF-Ga		1	2	3	3	1					2651.00	**2652.01**	**2673.99**	**1326.51**	**884.67**
mN	dF-TR		1	3	3	2	1					2666.99	**2668.00**	**2689.98**	**1334.50**	**890.01**
mN	mF-TRF		1	3	3	2	1					2666.99	**2668.00**	**2689.98**	**1334.50**	**890.01**
mN	mGa-dF-BIBS		1	3	3	2	1					2666.99	**2668.00**	**2689.98**	**1334.50**	**890.01**
mN	mGa-mF-BIBSF		1	3	3	2	1					2666.99	**2668.00**	**2689.98**	**1334.50**	**890.01**
mN	mF-GnLnM5BSF		1	3	3	2	1					2666.99	**2668.00**	**2689.98**	**1334.50**	**890.01**
mN	mF-dLnM4BSF		1	3	3	2	1					2666.99	**2668.00**	**2689.98**	**1334.50**	**890.01**
mN	trF-AG123BSF		1		4	4	1					2676.03	**2677.04**	**2699.02**	**1339.02**	**893.02**
mN	trF-GnLdnM3BSF		1		4	4	1					2676.03	**2677.04**	**2699.02**	**1339.02**	**893.02**
mN	trF-BI(dLdn)F		1		4	4	1					2676.03	**2677.04**	**2699.02**	**1339.02**	**893.02**
mN	mGa-TRF		1	4	3	1	1					2682.99	**2684.00**	**2705.98**	**1342.50**	**895.34**
mN	dGa-BIBSF		1	4	3	1	1					2682.99	**2684.00**	**2705.98**	**1342.50**	**895.34**
mN	mGa-mF-TR		1	4	3	1	1					2682.99	**2684.00**	**2705.98**	**1342.50**	**895.34**
mN	dGa-mF-BIBS		1	4	3	1	1					2682.99	**2684.00**	**2705.98**	**1342.50**	**895.34**
mN	dLnM5BSF		1	4	3	1	1					2682.99	**2684.00**	**2705.98**	**1342.50**	**895.34**
mN	dF-TRBSF-2G		1	1	4	3	1					2692.03	**2693.03**	**2715.01**	**1347.02**	**898.35**
mN	dF-BI(mLdn)BSF		1	1	4	3	1					2692.03	**2693.03**	**2715.01**	**1347.02**	**898.35**
mN	dGa-TR		1	5	3		1					2698.98	**2699.99**	**2721.97**	**1350.50**	**900.67**
mN	TR(trLdn)BS		1		7		1					2701.04	**2702.04**	**2724.03**	**1351.53**	**901.35**
mN	mF-TRBSF-Ga		1	2	4	2	1					2708.02	**2709.03**	**2731.01**	**1355.02**	**903.68**
mN	mF-LdnBIF		1	2	4	2	1					2708.02	**2709.03**	**2731.01**	**1355.02**	**903.68**
mN	mF-GnLdnM5BSF		1	2	4	2	1					2708.02	**2709.03**	**2731.01**	**1355.02**	**903.68**
mN	TRBSF		1	3	4	1	1					2724.02	**2725.02**	**2747.00**	**1363.01**	**909.01**
mN	mF-TRBS		1	3	4	1	1					2724.02	**2725.02**	**2747.00**	**1363.01**	**909.01**
mN	dF-AG1234BSF		1		5	3	1					2733.05	**2734.06**	**2756.04**	**1367.53**	**912.02**
mN	dF-dLdnM3BSF		1		5	3	1					2733.05	**2734.06**	**2756.04**	**1367.53**	**912.02**
mN	TE		1	4	4	0	1					2740.01	**2741.02**	**2763.00**	**1371.01**	**914.34**
mN	mGal-TRBS		1	4	4		1					2740.01	**2741.02**	**2763.00**	**1371.01**	**914.34**
mN	dLn-LdnM5A		1	4	4		1					2740.01	**2741.02**	**2763.00**	**1371.01**	**914.34**
mN	mF-TR(dLdn)F		1	1	5	2	1					2749.05	**2750.05**	**2772.04**	**1375.53**	**917.36**
mN	diGal-diF-BIF		1	4	2	3	1					2772.03	**2773.03**	**2795.01**	**1387.02**	**925.02**
mN	mF-trLdnM3F		1		6	2	1					2790.07	**2791.08**	**2813.06**	**1396.04**	**931.03**
mN	trF-TRF-Ga		1	2	3	4	1					2797.06	**2798.06**	**2820.05**	**1399.54**	**933.36**
mN	dF-TRF		1	3	3	3	1					2813.05	**2814.06**	**2836.04**	**1407.53**	**938.69**
mN	trF-TR		1	3	3	3	1					2813.05	**2814.06**	**2836.04**	**1407.53**	**938.69**
mN	mGa-dF-BIBSF		1	3	3	3	1					2813.05	**2814.06**	**2836.04**	**1407.53**	**938.69**
mN	mGa-dF-TR		1	4	3	2	1					2829.05	**2830.05**	**2852.04**	**1415.53**	**944.02**
mN	mGa-mF-TRF		1	4	3	2	1					2829.05	**2830.05**	**2852.04**	**1415.53**	**944.02**
mN	dGa-dF-BIBS		1	4	3	2	1					2829.05	**2830.05**	**2852.04**	**1415.53**	**944.02**
mN	dGa-mF-BIBSF		1	4	3	2	1					2829.05	**2830.05**	**2852.04**	**1415.53**	**944.02**
mN	mF-dLnM5BSF		1	4	3	2	1					2829.05	**2830.05**	**2852.04**	**1415.53**	**944.02**
mN	trF-TRBSF-2G		1	1	4	4	1					2838.08	**2839.09**	**2861.07**	**1420.05**	**947.04**
mN	trF-BI(mLdn)BSF		1	1	4	4	1					2838.08	**2839.09**	**2861.07**	**1420.05**	**947.04**
mN	dGa-TRF		1	5	3	1	1					2845.04	**2846.05**	**2868.03**	**1423.53**	**949.35**
mN	dGa-mF-TR		1	5	3	1	1					2845.04	**2846.05**	**2868.03**	**1423.53**	**949.35**
mN	trLdnM3BSF		1		7	1	1					2847.09	**2848.10**	**2870.08**	**1424.55**	**950.04**
mN	dF-TRBSF-G		1	2	4	3	1					2854.08	**2855.09**	**2877.07**	**1428.05**	**952.37**
mN	dF-LdnBIF		1	2	4	3	1					2854.08	**2855.09**	**2877.07**	**1428.05**	**952.37**

mN	trGa-TR		1	6	3		1					2861.04	**2862.04**	**2884.03**	**1431.53**	**954.69**
mN	mF-TRBSF, diF-TRBS		1	3	4	2	1					2870.07	**2871.08**	**2893.06**	**1436.04**	**957.70**
mN	trF-AG1234BSF		1		5	4	1					2879.11	**2880.12**	**2902.10**	**1440.56**	**960.71**
mN	trF-BI(dLdn)BSF		1		5	4	1					2879.11	**2880.12**	**2902.10**	**1440.56**	**960.71**
mN	TEF		1	4	4	1	1					2886.07	**2887.08**	**2909.06**	**1444.04**	**963.03**
mN	mGa-TRBSF		1	4	4	1	1					2886.07	**2887.08**	**2909.06**	**1444.04**	**963.03**
mN	mGa-mF-TRBS		1	4	4	1	1					2886.07	**2887.08**	**2909.06**	**1444.04**	**963.03**
mN	mF-dLn-LdnM5A		1	4	4	1	1					2886.07	**2887.08**	**2909.06**	**1444.04**	**963.03**
mN	dF-TR(dLdn)F		1	1	5	3	1					2895.10	**2896.11**	**2918.09**	**1448.56**	**966.04**
mN	dGa-TRBS		1	5	4		1					2902.06	**2903.07**	**2925.05**	**1452.04**	**968.36**
mN	TE(teLdn)		1		8		1					2904.12	**2905.12**	**2927.11**	**1453.07**	**969.05**
mN	dF-TR(trLdn)F		1		6	3	1					2936.13	**2937.14**	**2959.12**	**1469.07**	**979.72**
mN	TEBS		1	4	5		1					2943.09	**2944.10**	**2966.08**	**1472.55**	**982.04**
mN	trF-TRF		1	3	3	4	1					2959.11	**2960.12**	**2982.10**	**1480.56**	**987.38**
mN	mGa-trF-TR		1	4	3	3	1					2975.10	**2976.11**	**2998.09**	**1488.56**	**992.71**
mN	mGa-dF-TRF		1	4	3	3	1					2975.10	**2976.11**	**2998.09**	**1488.56**	**992.71**
mN	dGa-diF-BIBSF		1	4	3	3	1					2975.10	**2976.11**	**2998.09**	**1488.56**	**992.71**
mN	dGa-diF-TR		1	5	3	2	1					2991.10	**2992.11**	**3014.09**	**1496.56**	**998.04**
mN	dGa-mF-TRF		1	5	3	2	1					2991.10	**2992.11**	**3014.09**	**1496.56**	**998.04**
mN	mF-TR(trLdn)BSF		1		7	2	1					2993.15	**2994.16**	**3016.14**	**1497.58**	**998.72**
mN	trF-TRBSF-G		1	2	4	4	1					3000.14	**3001.14**	**3023.13**	**1501.08**	**1001.05**
mN	trF-TR(mLdn)F		1	2	4	4	1					3000.14	**3001.14**	**3023.13**	**1501.08**	**1001.05**
mN	trGa-TRF		1	6	3	1	1					3007.09	**3008.10**	**3030.08**	**1504.55**	**1003.37**
mN	trGa-mF-TR		1	6	3	1	1					3007.09	**3008.10**	**3030.08**	**1504.55**	**1003.37**
mN	dF-TRBSF		1	3	4	3	1					3016.13	**3017.14**	**3039.12**	**1509.07**	**1006.38**
mN	trF-TRBS		1	3	4	3	1					3016.13	**3017.14**	**3039.12**	**1509.07**	**1006.38**
mN	dF-TRBSF		1	3	4	3	1					3016.13	**3017.14**	**3039.12**	**1509.07**	**1006.38**
mN	teF-AG1234BSF		1		5	5	1					3025.17	**3026.18**	**3048.16**	**1513.59**	**1009.40**
mN	mF-TEF		1	4	4	2	1					3032.13	**3033.13**	**3055.12**	**1517.07**	**1011.72**
mN	mGa-dF-TRBS		1	4	4	2	1					3032.13	**3033.13**	**3055.12**	**1517.07**	**1011.72**
mN	mGa-mF-TRBSF		1	4	4	2	1					3032.13	**3033.13**	**3055.12**	**1517.07**	**1011.72**
mN	trF-TR(dLdn)F		1	1	5	4	1					3041.16	**3042.17**	**3064.15**	**1521.59**	**1014.73**
mN	dGa-TRBSF		1	5	4	1	1					3048.12	**3049.13**	**3071.11**	**1525.07**	**1017.05**
mN	dGa-mF-TRBS		1	5	4	1	1					3048.12	**3049.13**	**3071.11**	**1525.07**	**1017.05**
mN	(teLdn)F		1		8	1	1					3050.17	**3051.18**	**3073.16**	**1526.09**	**1017.73**
mN	trGa-TRBS		1	6	4		1					3064.12	**3065.12**	**3087.11**	**1533.07**	**1022.38**
mN	trF-trLdnM3F		1		6	4	1					3082.19	**3083.20**	**3105.18**	**1542.10**	**1028.40**
mN	TEBSF		1	4	5	1	1					3089.15	**3090.15**	**3112.14**	**1545.58**	**1030.72**
mN	TR(teLdn)BS		1		9		1					3107.20	**3108.20**	**3130.18**	**1554.61**	**1036.74**
mN	mGa-trF-TRF		1	4	3	4	1					3121.16	**3122.17**	**3144.15**	**1561.59**	**1041.39**
mN	dGa-trF-TR		1	5	3	3	1					3137.16	**3138.16**	**3160.15**	**1569.59**	**1046.73**
mN	dGa-dF-TRF		1	5	3	3	1					3137.16	**3138.16**	**3160.15**	**1569.59**	**1046.73**
mN	dF-TR(trLdn)BSF		1		7	3	1					3139.21	**3140.22**	**3162.20**	**1570.61**	**1047.41**
mN	trGa-dF-TR		1	6	3	2	1					3153.15	**3154.16**	**3176.14**	**1577.58**	**1052.06**
mN	trGa-mF-TRF		1	6	3	2	1					3153.15	**3154.16**	**3176.14**	**1577.58**	**1052.06**
mN	trF-TRBSF		1	3	4	4	1					3162.19	**3163.20**	**3185.18**	**1582.10**	**1055.07**
mN	dF-TEF		1	4	4	3	1					3178.18	**3179.19**	**3201.17**	**1590.10**	**1060.40**
mN	mGa-trF-TRBS		1	4	4	3	1					3178.18	**3179.19**	**3201.17**	**1590.10**	**1060.40**
mN	mGa-dF-TRBSF		1	4	4	3	1					3178.18	**3179.19**	**3201.17**	**1590.10**	**1060.40**
mN	dGa-dF-TRBS		1	5	4	2	1					3194.18	**3195.19**	**3217.17**	**1598.10**	**1065.73**
mN	dGa-mF-TRBSF		1	5	4	2	1					3194.18	**3195.19**	**3217.17**	**1598.10**	**1065.73**
mN	mF-teLdnM3F		1		8	2	1					3196.23	**3197.24**	**3219.22**	**1599.12**	**1066.42**
mN	trGa-TRBSF		1	6	4	1	1					3210.17	**3211.18**	**3233.16**	**1606.09**	**1071.07**
mN	trGa-mF-TRBS		1	6	4	1	1					3210.17	**3211.18**	**3233.16**	**1606.09**	**1071.07**
mN	mF-TEBSF		1	4	5	2	1					3235.21	**3236.21**	**3258.19**	**1618.61**	**1079.41**
mN	TE(teLdn)BSF		1		9	1	1					3253.25	**3254.26**	**3276.24**	**1627.63**	**1085.43**
mN	dGa-trF-TRF		1	5	3	4	1					3283.22	**3284.22**	**3306.20**	**1642.61**	**1095.41**
mN	trF-TR(trLdn)BSF		1		7	4	1					3285.27	**3286.28**	**3308.26**	**1643.64**	**1096.10**
mN	trGa-trF-TR		1	6	3	3	1					3299.21	**3300.22**	**3322.20**	**1650.61**	**1100.74**
mN	trGa-dF-TRF		1	6	3	3	1					3299.21	**3300.22**	**3322.20**	**1650.61**	**1100.74**
mN	mGa-trF-TRBSF		1	4	4	4	1					3324.24	**3325.25**	**3347.23**	**1663.13**	**1109.09**
mN	dGa-trF-TRBS		1	5	4	3	1					3340.24	**3341.24**	**3363.23**	**1671.13**	**1114.42**
mN	dGa-dF-TRBSF		1	5	4	3	1					3340.24	**3341.24**	**3363.23**	**1671.13**	**1114.42**
mN	dF-TE(teLdn)F		1		8	3	1					3342.29	**3343.30**	**3365.28**	**1672.15**	**1115.10**
mN	trGa-dF-TRBS		1	6	4	2	1					3356.23	**3357.24**	**3379.22**	**1679.12**	**1119.75**
mN	trGa-mF-TRBSF		1	6	4	2	1					3356.23	**3357.24**	**3379.22**	**1679.12**	**1119.75**
mN	dF-TEBSF		1	4	5	3	1					3381.26	**3382.27**	**3404.25**	**1691.64**	**1128.10**
mN	mF-TE(teLdn)BSF		1		9	2	1					3399.31	**3400.32**	**3422.30**	**1700.66**	**1134.11**
mN	dF-dLn-dLdnM5A		1	4	6	2	1					3438.28	**3439.29**	**3461.27**	**1720.15**	**1147.10**
mN	trGa-trF-TRF		1	6	3	4	1					3445.27	**3446.28**	**3468.26**	**1723.64**	**1149.43**
mN	dGa-trF-TRBSF		1	5	4	4	1					3486.29	**3487.30**	**3509.28**	**1744.15**	**1163.11**
mN	trF-(teLdn)F		1		8	4	1					3488.35	**3489.36**	**3511.34**	**1745.18**	**1163.79**
mN	trGa-trF-TRBS		1	6	4	3	1					3502.29	**3503.30**	**3525.28**	**1752.15**	**1168.44**

mN	trGa-dF-TRBSF		1	6	4	3	1					3502.29	**3503.30**	**3525.28**	**1752.15**	**1168.44**
mN	dF-(teLdn)BSF		1		9	3	1					3545.37	**3546.38**	**3568.36**	**1773.69**	**1182.80**
mN	triGal-triF-TRBSF		1	6	4	4	1					3648.35	**3649.35**	**3671.34**	**1825.18**	**1217.12**
mN	trF-teLdnM3BSF		1		9	4	1					3691.43	**3692.43**	**3714.42**	**1846.72**	**1231.48**
mN	dFdGa-BI'	1		7	3	2	1					2422.89	**2423.90**	**2445.88**	**1212.45**	**808.64**
mN	BI'	1		5	3		1					1806.67	**1807.67**	**1829.66**	**904.34**	**603.23**
mN	BI'(dLdn)	1		3	5		1					1888.72	**1889.73**	**1911.71**	**945.37**	**630.58**
mN	BI'(mLdn)	1		4	4		1					1847.69	**1848.70**	**1870.68**	**924.85**	**616.90**
mN	mF-BI'	1		5	3	1	1					1952.72	**1953.73**	**1975.71**	**977.37**	**651.92**

disialo (dN) glycans		reducing end		non-reducing end				modification					*m/z*			
		PA	M3PA	Hex	HexNAc	dHex	NANA	Me	Ac	HPO_3	SO_3	MS number	+H	+Na	+2H	+3H
dN	Tn	1			1		2					881.34	**882.35**	**904.33**	**441.68**	**294.79**
dN	Gal	1		1			2					840.31	**841.32**	**863.30**	**421.16**	**281.11**
dN	core1, Gn-Man	1		1	1		2					1043.39	**1044.40**	**1066.38**	**522.70**	**348.80**
dN	Ga-Gn-F	1		1	1	1	2					1189.45	**1190.46**	**1212.44**	**595.73**	**397.49**
dN	Ln-Man	1		2	1		2					1205.44	**1206.45**	**1228.43**	**603.73**	**402.82**
dN	G-core1	1		2	1		2					1205.44	**1206.45**	**1228.43**	**603.73**	**402.82**
dN	dGn-Man	1		1	2		2					1246.47	**1247.48**	**1269.46**	**624.24**	**416.50**
dN	mF-Gan-core1	1		1	2	1	2					1392.53	**1393.54**	**1415.52**	**697.27**	**465.18**
dN	mF-G-core1	1		2	1	1	2					1351.50	**1352.51**	**1374.49**	**676.76**	**451.51**
dN	GnLn-Man	1		2	2		2					1408.52	**1409.53**	**1431.51**	**705.27**	**470.52**
dN	Gan-G-core1	1		2	2		2					1408.52	**1409.53**	**1431.51**	**705.27**	**470.52**
dN	G-core2	1		2	2		2					1408.52	**1409.53**	**1431.51**	**705.27**	**470.52**
dN	GanGn-Core1	1		1	3		2					1449.55	**1450.56**	**1472.54**	**725.78**	**484.19**
dN	mF-Gan-G-core1	1		2	2	1	2					1554.58	**1555.59**	**1577.57**	**778.30**	**519.20**
dN	dLn-Man	1		3	2		2					1570.58	**1571.58**	**1593.57**	**786.30**	**524.53**
dN	M1BSF	1		1	3	1	2					1595.61	**1596.62**	**1618.60**	**798.81**	**532.88**
dN	GnM2	1		2	3		2					1611.60	**1612.61**	**1634.59**	**806.81**	**538.21**
dN	M1BSdF	1		1	3	2	2					1741.67	**1742.67**	**1764.66**	**871.84**	**581.56**
dN	GnM2F	1		2	3	1	2					1757.66	**1758.67**	**1780.65**	**879.84**	**586.89**
dN	LnM2, AG1	1		3	3		2					1773.66	**1774.66**	**1796.65**	**887.84**	**592.23**
dN	GnM2BS	1		2	4		2					1814.68	**1815.69**	**1837.67**	**908.35**	**605.90**
dN	LdnM2	1		2	4		2					1814.68	**1815.69**	**1837.67**	**908.35**	**605.90**
dN	Ac-AG1		1		1		2		1			1815.67	**1816.67**	**1838.66**	**908.84**	**606.23**
dN	GnM2BSF			2	4	1	2					1864.67	**1865.68**	**1887.66**	**933.34**	**622.56**
dN	mF-GnM2F	1		2	3	2	2					1903.72	**1904.73**	**1926.71**	**952.87**	**635.58**
dN	AG1F		1		1	1	2					1919.71	**1920.72**	**1942.70**	**960.86**	**640.91**
dN	LnM2F	1		3	3	1	2					1919.71	**1920.72**	**1942.70**	**960.86**	**640.91**
dN	GnM4		1	1	1	0	2					1935.71	**1936.72**	**1958.70**	**968.86**	**646.24**
dN	MO		1	1	1	0	2					1935.71	**1936.72**	**1958.70**	**968.86**	**646.24**
dN	LdnM2F	1		2	4	1	2					1960.74	**1961.75**	**1983.73**	**981.38**	**654.59**
dN	LdnM3, AG12, AG1BS		1		2		2					1976.74	**1977.74**	**1999.72**	**989.38**	**659.92**
dN	LnM2BS	1		3	4		2					1976.74	**1977.74**	**1999.72**	**989.38**	**659.92**
dN	mF-GnM2BSF			2	4	2	2					2010.73	**2011.74**	**2033.72**	**1006.37**	**671.25**
dN	LdnM2BS	1		2	5		2					2017.76	**2018.77**	**2040.75**	**1009.89**	**673.59**
dN	GnLdnM2	1		2	5		2					2017.76	**2018.77**	**2040.75**	**1009.89**	**673.59**
dN	Ac-AG12		1		2		2		1			2018.75	**2019.75**	**2041.74**	**1010.38**	**673.92**
dN	dF-GnM2F	1		2	3	3	2					2049.78	**2050.78**	**2072.77**	**1025.90**	**684.27**
dN	mF-AG1F		1		1	2	2					2065.77	**2066.78**	**2088.76**	**1033.89**	**689.60**
dN	mF-LnM2F	1		3	3	2	2					2065.77	**2066.78**	**2088.76**	**1033.89**	**689.60**
dN	MOF		1	1	1	1	2					2081.77	**2082.77**	**2104.76**	**1041.89**	**694.93**
dN	GnM4F		1	1	1	1	2					2081.77	**2082.77**	**2104.76**	**1041.89**	**694.93**
dN	mGal-MO		1	2	1		2					2097.76	**2098.77**	**2120.75**	**1049.89**	**700.26**
dN	LnM4		1	2	1	0	2					2097.76	**2098.77**	**2120.75**	**1049.89**	**700.26**
dN	GnM5		1	2	1	0	2					2097.76	**2098.77**	**2120.75**	**1049.89**	**700.26**
dN	mF-LdnF	1		2	4	2	2					2106.80	**2107.81**	**2129.79**	**1054.41**	**703.27**
dN	AG12F, AG1BSF		1		2	1	2					2122.79	**2123.80**	**2145.78**	**1062.40**	**708.61**
dN	LdnM3F		1		2	1	2					2122.79	**2123.80**	**2145.78**	**1062.40**	**708.61**
dN	LnM2BSF	1		3	4	1	2					2122.79	**2123.80**	**2145.78**	**1062.40**	**708.61**
dN	MOBS		1	1	2	0	2					2138.79	**2139.80**	**2161.78**	**1070.40**	**713.94**
dN	BI-Ga		1	1	2	0	2					2138.79	**2139.80**	**2161.78**	**1070.40**	**713.94**
dN	GnM4BS		1	1	2	0	2					2138.79	**2139.80**	**2161.78**	**1070.40**	**713.94**
dN	LdnM4		1	1	2		2					2138.79	**2139.80**	**2161.78**	**1070.40**	**713.94**
dN	dF-GnM2BSF			2	4	3	2					2156.79	**2157.79**	**2179.78**	**1079.40**	**719.94**
dN	LdnM2BSF	1		2	5	1	2					2163.82	**2164.83**	**2186.81**	**1082.92**	**722.28**
dN	GnLdnM2F	1		2	5	1	2					2163.82	**2164.83**	**2186.81**	**1082.92**	**722.28**
dN	AG123, AG12BS		1		3		2					2179.81	**2180.82**	**2202.80**	**1090.91**	**727.61**
dN	GnLdnM3, LdnM3BS		1		3		2					2179.81	**2180.82**	**2202.80**	**1090.91**	**727.61**
dN	Ac-BI-Ga		1	1	2		2		1			2180.80	**2181.81**	**2203.79**	**1091.41**	**727.94**
dN	dF-AG1F		1		1	3	2					2211.83	**2212.84**	**2234.82**	**1106.92**	**738.28**
dN	dLdnM2	1		2	6		2					2220.84	**2221.85**	**2243.83**	**1111.43**	**741.29**
dN	mF-MOF		1	1	1	2	2					2227.82	**2228.83**	**2250.81**	**1114.92**	**743.62**
dN	mF-GnM4F		1	1	1	2	2					2227.82	**2228.83**	**2250.81**	**1114.92**	**743.62**
dN	mGal-MOF		1	2	1	1	2					2243.82	**2244.83**	**2266.81**	**1122.92**	**748.95**
dN	LnM4F, GnM5F		1	2	1	1	2					2243.82	**2244.83**	**2266.81**	**1122.92**	**748.95**
dN	LnM5, GnM6		1	3	1	0	2					2259.81	**2260.82**	**2282.80**	**1130.91**	**754.28**
dN	mF-AG1BSF		1		2	2	2					2268.85	**2269.86**	**2291.84**	**1135.43**	**757.29**
dN	mF-AG12F		1		2	2	2					2268.85	**2269.86**	**2291.84**	**1135.43**	**757.29**
dN	mF-LnM2BSF	1		3	4	2	2					2268.85	**2269.86**	**2291.84**	**1135.43**	**757.29**
dN	mF-LdnM3F		1		2	2	2					2268.85	**2269.86**	**2291.84**	**1135.43**	**757.29**
dN	MOBSF		1	1	2	1	2					2284.85	**2285.85**	**2307.84**	**1143.43**	**762.62**
dN	BIF-Ga		1	1	2	1	2					2284.85	**2285.85**	**2307.84**	**1143.43**	**762.62**

dN	GnM4BSF		1	1	2	1	2					2284.85	**2285.85**	**2307.84**	**1143.43**	**762.62**
dN	LdnM4F		1	1	2	1	2					2284.85	**2285.85**	**2307.84**	**1143.43**	**762.62**
dN	BI		1	2	2	0	2					2300.84	**2301.85**	**2323.83**	**1151.43**	**767.95**
dN	mGal-MOBS		1	2	2		2					2300.84	**2301.85**	**2323.83**	**1151.43**	**767.95**
dN	mGal-BI-G		1	2	2		2					2300.84	**2301.85**	**2323.83**	**1151.43**	**767.95**
dN	LnM4BS		1	2	2	0	2					2300.84	**2301.85**	**2323.83**	**1151.43**	**767.95**
dN	GnM5BS		1	2	2	0	2					2300.84	**2301.85**	**2323.83**	**1151.43**	**767.95**
dN	GnLnM4		1	2	2		2					2300.84	**2301.85**	**2323.83**	**1151.43**	**767.95**
dN	LdnM5		1	2	2		2					2300.84	**2301.85**	**2323.83**	**1151.43**	**767.95**
dN	mF-LdnBSF	1		2	5	2	2					2309.88	**2310.89**	**2332.87**	**1155.95**	**770.97**
dN	mF-GnLdnM2F	1		2	5	2	2					2309.88	**2310.89**	**2332.87**	**1155.95**	**770.97**
dN	AG12BSF		1		3	1	2					2325.87	**2326.88**	**2348.86**	**1163.94**	**776.30**
dN	AG123F		1		3	1	2					2325.87	**2326.88**	**2348.86**	**1163.94**	**776.30**
dN	GnLdnM3F, LdnM3BSF		1		3	1	2					2325.87	**2326.88**	**2348.86**	**1163.94**	**776.30**
dN	BIBS-G		1	1	3	0	2					2341.87	**2342.88**	**2364.86**	**1171.94**	**781.63**
dN	TR-2G		1	1	3		2					2341.87	**2342.88**	**2364.86**	**1171.94**	**781.63**
dN	BI(mLdn)		1	1	3		2					2341.87	**2342.88**	**2364.86**	**1171.94**	**781.63**
dN	LdnM4BS		1	1	3		2					2341.87	**2342.88**	**2364.86**	**1171.94**	**781.63**
dN	GnLdnM4		1	1	3		2					2341.87	**2342.88**	**2364.86**	**1171.94**	**781.63**
dN	Ac-BI		1	2	2		2		1			2342.85	**2343.86**	**2365.84**	**1172.43**	**781.96**
dN	dLdnM2F	1		2	6	1	2					2366.90	**2367.91**	**2389.89**	**1184.46**	**789.97**
dN	AG123BS		1		4		2					2382.89	**2383.90**	**2405.88**	**1192.45**	**795.31**
dN	GnLdnM3BS		1		4		2					2382.89	**2383.90**	**2405.88**	**1192.45**	**795.31**
dN	BI(dLdn)		1		4		2					2382.89	**2383.90**	**2405.88**	**1192.45**	**795.31**
dN	mGal-mF-MOF		1	2	1	2	2					2389.88	**2390.88**	**2412.87**	**1195.95**	**797.63**
dN	mF-LnM4F		1	2	1	2	2					2389.88	**2390.88**	**2412.87**	**1195.95**	**797.63**
dN	mF-GnM5F		1	2	1	2	2					2389.88	**2390.88**	**2412.87**	**1195.95**	**797.63**
dN	LnM5F		1	3	1	1	2					2405.87	**2406.88**	**2428.86**	**1203.94**	**802.96**
dN	dF-AG1BSF		1		2	3	2					2414.91	**2415.92**	**2437.90**	**1208.46**	**805.98**
dN	dF-AG12F		1		2	3	2					2414.91	**2415.92**	**2437.90**	**1208.46**	**805.98**
dN	mG-LnM5		1	4	1	0	2					2421.87	**2422.87**	**2444.86**	**1211.94**	**808.30**
dN	mF-MOBSF		1	1	2	2	2					2430.90	**2431.91**	**2453.89**	**1216.46**	**811.31**
dN	mF-BIF-G		1	1	2	2	2					2430.90	**2431.91**	**2453.89**	**1216.46**	**811.31**
dN	mF-GnM4BSF		1	1	2	2	2					2430.90	**2431.91**	**2453.89**	**1216.46**	**811.31**
dN	mF-LdnM4F		1	1	2	2	2					2430.90	**2431.91**	**2453.89**	**1216.46**	**811.31**
dN	BIF, mF-BI		1	2	2	1	2					2446.90	**2447.91**	**2469.89**	**1224.46**	**816.64**
dN	mGa-MOBSF		1	2	2	1	2					2446.90	**2447.91**	**2469.89**	**1224.46**	**816.64**
dN	mGa-BIF-G		1	2	2	1	2					2446.90	**2447.91**	**2469.89**	**1224.46**	**816.64**
dN	mGa-mF-MOBS		1	2	2	1	2					2446.90	**2447.91**	**2469.89**	**1224.46**	**816.64**
dN	LnM4BSF		1	2	2	1	2					2446.90	**2447.91**	**2469.89**	**1224.46**	**816.64**
dN	GnM5BSF		1	2	2	1	2					2446.90	**2447.91**	**2469.89**	**1224.46**	**816.64**
dN	GnLnM4F		1	2	2	1	2					2446.90	**2447.91**	**2469.89**	**1224.46**	**816.64**
dN	LdnM5F		1	2	2	1	2					2446.90	**2447.91**	**2469.89**	**1224.46**	**816.64**
dN	dF-GnLdnM2F	1		2	5	3	2					2455.94	**2456.94**	**2478.93**	**1228.98**	**819.65**
dN	mGa-BI		1	3	2		2					2462.89	**2463.90**	**2485.88**	**1232.45**	**821.97**
dN	LnM5BS		1	3	2		2					2462.89	**2463.90**	**2485.88**	**1232.45**	**821.97**
dN	GnLnM5		1	3	2		2					2462.89	**2463.90**	**2485.88**	**1232.45**	**821.97**
dN	dLnM4		1	3	2		2					2462.89	**2463.90**	**2485.88**	**1232.45**	**821.97**
dN	mF-AG12BSF		1		3	2	2					2471.93	**2472.94**	**2494.92**	**1236.97**	**824.98**
dN	mF-AG123F		1		3	2	2					2471.93	**2472.94**	**2494.92**	**1236.97**	**824.98**
dN	mF-LdnM3BSF		1		3	2	2					2471.93	**2472.94**	**2494.92**	**1236.97**	**824.98**
dN	mF-LdnAG1F		1		3	2	2					2471.93	**2472.94**	**2494.92**	**1236.97**	**824.98**
dN	BIBSF-Ga		1	1	3	1	2					2487.93	**2488.93**	**2510.91**	**1244.97**	**830.32**
dN	TRF-2Ga		1	1	3	1	2					2487.93	**2488.93**	**2510.91**	**1244.97**	**830.32**
dN	BI(mLdn)F		1	1	3	1	2					2487.93	**2488.93**	**2510.91**	**1244.97**	**830.32**
dN	LdnM4BSF		1	1	3	1	2					2487.93	**2488.93**	**2510.91**	**1244.97**	**830.32**
dN	GnLdnM4F		1	1	3	1	2					2487.93	**2488.93**	**2510.91**	**1244.97**	**830.32**
dN	BIBS		1	2	3	0	2					2503.92	**2504.93**	**2526.91**	**1252.97**	**835.65**
dN	TR-Ga		1	2	3		2					2503.92	**2504.93**	**2526.91**	**1252.97**	**835.65**
dN	mGal-BIBS-Ga		1	2	3		2					2503.92	**2504.93**	**2526.91**	**1252.97**	**835.65**
dN	GnLnM4BS		1	2	3		2					2503.92	**2504.93**	**2526.91**	**1252.97**	**835.65**
dN	LdnM5BS		1	2	3		2					2503.92	**2504.93**	**2526.91**	**1252.97**	**835.65**
dN	GnLdnM5		1	2	3		2					2503.92	**2504.93**	**2526.91**	**1252.97**	**835.65**
dN	mF-dLdnM2F	1		2	6	2	2					2512.96	**2513.96**	**2535.95**	**1257.49**	**838.66**
dN	AG123BSF		1		4	1	2					2528.95	**2529.96**	**2551.94**	**1265.48**	**843.99**
dN	LdnAG1BSF		1		4	1	2					2528.95	**2529.96**	**2551.94**	**1265.48**	**843.99**
dN	BI(dLdn)F		1		4	1	2					2528.95	**2529.96**	**2551.94**	**1265.48**	**843.99**
dN	TRBS-2Ga		1	1	4		2					2544.95	**2545.95**	**2567.94**	**1273.48**	**849.32**
dN	BI(mLdn)BS		1	1	4		2					2544.95	**2545.95**	**2567.94**	**1273.48**	**849.32**
dN	GnLdnM4BS		1	1	4		2					2544.95	**2545.95**	**2567.94**	**1273.48**	**849.32**
dN	mF-LnM5F		1	3	1	2	2					2551.93	**2552.94**	**2574.92**	**1276.97**	**851.65**
dN	trF-AG12F		1		2	4	2					2560.97	**2561.97**	**2583.96**	**1281.49**	**854.66**
dN	mG-LnM5F		1	4	1	1	2					2567.93	**2568.93**	**2590.91**	**1284.97**	**856.98**

dN	dF-BIF-Ga		1	1	2	3	2					2576.96	**2577.97**	**2599.95**	**1289.49**	**859.99**
dN	AG1234BS		1		5		2					2585.97	**2586.98**	**2608.96**	**1293.99**	**863.00**
dN	BI(dLdn)BS		1		5		2					2585.97	**2586.98**	**2608.96**	**1293.99**	**863.00**
dN	mF-BIF, diF-BI		1	2	2	2	2					2592.96	**2593.96**	**2615.95**	**1297.49**	**865.33**
dN	mGa-mF-MOBSF		1	2	2	2	2					2592.96	**2593.96**	**2615.95**	**1297.49**	**865.33**
dN	mGa-mF-BIF-Ga		1	2	2	2	2					2592.96	**2593.96**	**2615.95**	**1297.49**	**865.33**
dN	mF-LnM4BSF		1	2	2	2	2					2592.96	**2593.96**	**2615.95**	**1297.49**	**865.33**
dN	mF-GnM5BSF		1	2	2	2	2					2592.96	**2593.96**	**2615.95**	**1297.49**	**865.33**
dN	mF-GnLnM4F		1	2	2	2	2					2592.96	**2593.96**	**2615.95**	**1297.49**	**865.33**
dN	mF-LdnM5F		1	2	2	2	2					2592.96	**2593.96**	**2615.95**	**1297.49**	**865.33**
dN	trF-GnLdnM2F	1		2	5	4	2					2601.99	**2603.00**	**2624.98**	**1302.00**	**868.34**
dN	mGa-BIF		1	3	2	1	2					2608.95	**2609.96**	**2631.94**	**1305.48**	**870.66**
dN	mGa-mF-BI		1	3	2	1	2					2608.95	**2609.96**	**2631.94**	**1305.48**	**870.66**
dN	LnM5BSF		1	3	2	1	2					2608.95	**2609.96**	**2631.94**	**1305.48**	**870.66**
dN	GnLnM5F		1	3	2	1	2					2608.95	**2609.96**	**2631.94**	**1305.48**	**870.66**
dN	dLnM4F		1	3	2	1	2					2608.95	**2609.96**	**2631.94**	**1305.48**	**870.66**
dN	dF-AG12BSF		1		3	3	2					2617.99	**2619.00**	**2640.98**	**1310.00**	**873.67**
dN	dF-AG123F		1		3	3	2					2617.99	**2619.00**	**2640.98**	**1310.00**	**873.67**
dN	dF-GnLdnM3F		1		3	3	2					2617.99	**2619.00**	**2640.98**	**1310.00**	**873.67**
dN	dGa-BI		1	4	2		2					2624.95	**2625.95**	**2647.94**	**1313.48**	**875.99**
dN	mG-LnM5BS		1	4	2	0	2					2624.95	**2625.95**	**2647.94**	**1313.48**	**875.99**
dN	dLnM5		1	4	2		2					2624.95	**2625.95**	**2647.94**	**1313.48**	**875.99**
dN	mF-BIBSF-G		1	1	3	2	2					2633.98	**2634.99**	**2656.97**	**1318.00**	**879.00**
dN	mF-TRF-2G		1	1	3	2	2					2633.98	**2634.99**	**2656.97**	**1318.00**	**879.00**
dN	mF-BI(mLdn)F		1	1	3	2	2					2633.98	**2634.99**	**2656.97**	**1318.00**	**879.00**
dN	mF-LdnM4BSF		1	1	3	2	2					2633.98	**2634.99**	**2656.97**	**1318.00**	**879.00**
dN	mF-GnLdnM4F		1	1	3	2	2					2633.98	**2634.99**	**2656.97**	**1318.00**	**879.00**
dN	BIBSF		1	2	3	1	2					2649.98	**2650.99**	**2672.97**	**1326.00**	**884.33**
dN	TRF-Ga		1	2	3	1	2					2649.98	**2650.99**	**2672.97**	**1326.00**	**884.33**
dN	mGa-BIBSF-Ga		1	2	3	1	2					2649.98	**2650.99**	**2672.97**	**1326.00**	**884.33**
dN	mGa-mF-BIBS-Ga		1	2	3	1	2					2649.98	**2650.99**	**2672.97**	**1326.00**	**884.33**
dN	mF-BIBS		1	2	3	1	2					2649.98	**2650.99**	**2672.97**	**1326.00**	**884.33**
dN	GnLnM4BSF		1	2	3	1	2					2649.98	**2650.99**	**2672.97**	**1326.00**	**884.33**
dN	LdnM5BSF		1	2	3	1	2					2649.98	**2650.99**	**2672.97**	**1326.00**	**884.33**
dN	GnLdnM5F		1	2	3	1	2					2649.98	**2650.99**	**2672.97**	**1326.00**	**884.33**
dN	dF-dLdnM2F	1		2	6	3	2					2659.02	**2660.02**	**2682.00**	**1330.51**	**887.35**
dN	TR		1	3	3	0	2					2665.97	**2666.98**	**2688.96**	**1333.99**	**889.67**
dN	mGa-BIBS		1	3	3		2					2665.97	**2666.98**	**2688.96**	**1333.99**	**889.67**
dN	GnLnM5BS		1	3	3		2					2665.97	**2666.98**	**2688.96**	**1333.99**	**889.67**
dN	dLnM4BS		1	3	3		2					2665.97	**2666.98**	**2688.96**	**1333.99**	**889.67**
dN	mF-AG123BSF		1		4	2	2					2675.01	**2676.02**	**2698.00**	**1338.51**	**892.68**
dN	mF-LdnAG1BSF		1		4	2	2					2675.01	**2676.02**	**2698.00**	**1338.51**	**892.68**
dN	mF-dLdnM3F		1		4	2	2					2675.01	**2676.02**	**2698.00**	**1338.51**	**892.68**
dN	TRBSF-2G		1	1	4	1	2					2691.01	**2692.01**	**2713.99**	**1346.51**	**898.01**
dN	BI(mLdn)BSF		1	1	4	1	2					2691.01	**2692.01**	**2713.99**	**1346.51**	**898.01**
dN	GnLdnM4BSF		1	1	4	1	2					2691.01	**2692.01**	**2713.99**	**1346.51**	**898.01**
dN	TRBS-G		1	2	4	0	2					2707.00	**2708.01**	**2729.99**	**1354.51**	**903.34**
dN	TR(mLdn)		1	2	4		2					2707.00	**2708.01**	**2729.99**	**1354.51**	**903.34**
dN	GnLdnM5BS		1	2	4		2					2707.00	**2708.01**	**2729.99**	**1354.51**	**903.34**
dN	AG1234BSF		1		5	1	2					2732.03	**2733.04**	**2755.02**	**1367.02**	**911.68**
dN	BI(dLdn)BSF		1		5	1	2					2732.03	**2733.04**	**2755.02**	**1367.02**	**911.68**
dN	dF-BIF		1	2	2	3	2					2739.01	**2740.02**	**2762.00**	**1370.51**	**914.01**
dN	TR(dLdn)		1	1	5		2					2748.03	**2749.03**	**2771.02**	**1375.02**	**917.02**
dN	mGa-dF-BI		1	3	2	2	2					2755.01	**2756.02**	**2778.00**	**1378.51**	**919.34**
dN	mGa-mF-BIF		1	3	2	2	2					2755.01	**2756.02**	**2778.00**	**1378.51**	**919.34**
dN	mF-LnM5BSF		1	3	2	2	2					2755.01	**2756.02**	**2778.00**	**1378.51**	**919.34**
dN	mF-GnLnM5F		1	3	2	2	2					2755.01	**2756.02**	**2778.00**	**1378.51**	**919.34**
dN	mF-dLnM4F		1	3	2	2	2					2755.01	**2756.02**	**2778.00**	**1378.51**	**919.34**
dN	trF-AG12BSF		1		3	4	2					2764.05	**2765.05**	**2787.04**	**1383.03**	**922.36**
dN	trF-AG123F		1		3	4	2					2764.05	**2765.05**	**2787.04**	**1383.03**	**922.36**
dN	trF-LdnAG1F		1		3	4	2					2764.05	**2765.05**	**2787.04**	**1383.03**	**922.36**
dN	dGa-BIF, dGa-mF-BI		1	4	2	1	2					2771.00	**2772.01**	**2793.99**	**1386.51**	**924.68**
dN	mGa-LnM5BSF		1	4	2	1	2					2771.00	**2772.01**	**2793.99**	**1386.51**	**924.68**
dN	dLnM5F		1	4	2	1	2					2771.00	**2772.01**	**2793.99**	**1386.51**	**924.68**
dN	dF-BIBSF-G		1	1	3	3	2					2780.04	**2781.05**	**2803.03**	**1391.03**	**927.69**
dN	dF-TRF-2G		1	1	3	3	2					2780.04	**2781.05**	**2803.03**	**1391.03**	**927.69**
dN	dF-BI(mLdn)F		1	1	3	3	2					2780.04	**2781.05**	**2803.03**	**1391.03**	**927.69**
dN	TR(trLdn)		1		6		2					2789.05	**2790.06**	**2812.04**	**1395.53**	**930.69**
dN	mF-BIBSF		1	2	3	2	2					2796.04	**2797.04**	**2819.03**	**1399.03**	**933.02**
dN	mF-TRF-G		1	2	3	2	2					2796.04	**2797.04**	**2819.03**	**1399.03**	**933.02**
dN	mGa-mF-BIBSF-G		1	2	3	2	2					2796.04	**2797.04**	**2819.03**	**1399.03**	**933.02**
dN	dF-BIBS, mF-BIBSF		1	2	3	2	2					2796.04	**2797.04**	**2819.03**	**1399.03**	**933.02**
dN	mF-GnLnM4BSF		1	2	3	2	2					2796.04	**2797.04**	**2819.03**	**1399.03**	**933.02**

dN	mF-LdnM5BSF		1	2	3	2	2					2796.04	2797.04	2819.03	1399.03	933.02
dN	mF-GnLdnM5F		1	2	3	2	2					2796.04	2797.04	2819.03	1399.03	933.02
dN	trF-dLdnM2F	1		2	6	4	2					2805.07	2806.08	2828.06	1403.54	936.03
dN	TRF, mF-TR		1	3	3	1	2					2812.03	2813.04	2835.02	1407.02	938.35
dN	mGa-BIBSF		1	3	3	1	2					2812.03	2813.04	2835.02	1407.02	938.35
dN	mGal-mF-BIBS		1	3	3	1	2					2812.03	2813.04	2835.02	1407.02	938.35
dN	GnLnM5BSF		1	3	3	1	2					2812.03	2813.04	2835.02	1407.02	938.35
dN	diLnM4BSF		1	3	3	1	2					2812.03	2813.04	2835.02	1407.02	938.35
dN	dF-AG123BSF		1		4	3	2					2821.07	2822.08	2844.06	1411.54	941.36
dN	dF-LdnAG1BSF		1		4	3	2					2821.07	2822.08	2844.06	1411.54	941.36
dN	dF-dLdnM3F		1		4	3	2					2821.07	2822.08	2844.06	1411.54	941.36
dN	mGa-TR, dGa-BIBS		1	4	3		2					2828.03	2829.03	2851.02	1415.02	943.68
dN	dLnM5BS		1	4	3		2					2828.03	2829.03	2851.02	1415.02	943.68
dN	mF-TRBSF-2G		1	1	4	2	2					2837.06	2838.07	2860.05	1419.54	946.69
dN	mF-LdnMOBSF		1	1	4	2	2					2837.06	2838.07	2860.05	1419.54	946.69
dN	mF-GnLdnM4BSF		1	1	4	2	2					2837.06	2838.07	2860.05	1419.54	946.69
dN	TRBSF-G		1	2	4	1	2					2853.06	2854.07	2876.05	1427.54	952.03
dN	TR(mLdn)F		1	2	4	1	2					2853.06	2854.07	2876.05	1427.54	952.03
dN	GnLdnM5BSF		1	2	4	1	2					2853.06	2854.07	2876.05	1427.54	952.03
dN	TRBS		1	3	4	0	2					2869.05	2870.06	2892.04	1435.53	957.36
dN	mF-AG1234BSF		1		5	2	2					2878.09	2879.10	2901.08	1440.05	960.37
dN	mF-dLdnM3BSF		1		5	2	2					2878.09	2879.10	2901.08	1440.05	960.37
dN	TR(dLdn)F		1	1	5	1	2					2894.08	2895.09	2917.07	1448.05	965.70
dN	mGa-dF-BIF		1	3	2	3	2					2901.07	2902.08	2924.06	1451.54	968.03
dN	dGa-dF-BI		1	4	2	2	2					2917.06	2918.07	2940.05	1459.54	973.36
dN	dGa-mF-BIF		1	4	2	2	2					2917.06	2918.07	2940.05	1459.54	973.36
dN	mF-diLnM5F		1	4	2	2	2					2917.06	2918.07	2940.05	1459.54	973.36
dN	trF-TRF-2G		1	1	3	4	2					2926.10	2927.11	2949.09	1464.06	976.37
dN	trF-LdnMOF		1	1	3	4	2					2926.10	2927.11	2949.09	1464.06	976.37
dN	TR(trLdn)F		1		6	1	2					2935.11	2936.12	2958.10	1468.56	979.38
dN	dF-BIBSF		1	2	3	3	2					2942.09	2943.10	2965.08	1472.05	981.71
dN	dF-TRF-Ga		1	2	3	3	2					2942.09	2943.10	2965.08	1472.05	981.71
dN	dF-TR		1	3	3	2	2					2958.09	2959.10	2981.08	1480.05	987.04
dN	mF-TRF		1	3	3	2	2					2958.09	2959.10	2981.08	1480.05	987.04
dN	mGa-dF-BIBS		1	3	3	2	2					2958.09	2959.10	2981.08	1480.05	987.04
dN	mGa-mF-BIBSF		1	3	3	2	2					2958.09	2959.10	2981.08	1480.05	987.04
dN	mF-GnLnM5BSF		1	3	3	2	2					2958.09	2959.10	2981.08	1480.05	987.04
dN	mF-dLnM4BSF		1	3	3	2	2					2958.09	2959.10	2981.08	1480.05	987.04
dN	Ac-dG-dF-BI		1	4	2	2	2		1			2959.07	2960.08	2982.06	1480.54	987.37
dN	trF-AG123BSF		1		4	4	2					2967.13	2968.13	2990.12	1484.57	990.05
dN	trF-GnLdnM3BSF		1		4	4	2					2967.13	2968.13	2990.12	1484.57	990.05
dN	trF-BI(dLdn)F		1		4	4	2					2967.13	2968.13	2990.12	1484.57	990.05
dN	mGa-TRF		1	4	3	1	2					2974.08	2975.09	2997.07	1488.05	992.37
dN	dGa-BIBSF		1	4	3	1	2					2974.08	2975.09	2997.07	1488.05	992.37
dN	mGa-mF-TR		1	4	3	1	2					2974.08	2975.09	2997.07	1488.05	992.37
dN	dGa-mF-BIBS		1	4	3	1	2					2974.08	2975.09	2997.07	1488.05	992.37
dN	dLnM5BSF		1	4	3	1	2					2974.08	2975.09	2997.07	1488.05	992.37
dN	dF-TRBSF-2G		1	1	4	3	2					2983.12	2984.13	3006.11	1492.57	995.38
dN	dF-BI(mLdn)BSF		1	1	4	3	2					2983.12	2984.13	3006.11	1492.57	995.38
dN	dGa-TR		1	5	3		2					2990.08	2991.09	3013.07	1496.05	997.70
dN	TR(trLdn)BS		1		7		2					2992.13	2993.14	3015.12	1497.07	998.38
dN	mF-TRBSF-Ga		1	2	4	2	2					2999.12	3000.12	3022.10	1500.57	1000.71
dN	mF-LdnBIF		1	2	4	2	2					2999.12	3000.12	3022.10	1500.57	1000.71
dN	mF-GnLdnM5BSF		1	2	4	2	2					2999.12	3000.12	3022.10	1500.57	1000.71
dN	TRBSF		1	3	4	1	2					3015.11	3016.12	3038.10	1508.56	1006.04
dN	mF-TRBS		1	3	4	1	2					3015.11	3016.12	3038.10	1508.56	1006.04
dN	dF-AG1234BSF		1		5	3	2					3024.15	3025.15	3047.14	1513.08	1009.06
dN	dF-dLdnM3BSF		1		5	3	2					3024.15	3025.15	3047.14	1513.08	1009.06
dN	TE		1	4	4	0	2					3031.11	3032.11	3054.09	1516.56	1011.38
dN	mGal-TRBS		1	4	4		2					3031.11	3032.11	3054.09	1516.56	1011.38
dN	dLn-LdnM5A		1	4	4		2					3031.11	3032.11	3054.09	1516.56	1011.38
dN	mF-TR(dLdn)F		1	1	5	2	2					3040.14	3041.15	3063.13	1521.08	1014.39
dN	diGal-diF-BIF		1	4	2	3	2					3063.12	3064.13	3086.11	1532.57	1022.05
dN	mF-trLdnM3F		1		6	2	2					3081.17	3082.18	3104.16	1541.59	1028.06
dN	trF-TRF-Ga		1	2	3	4	2					3088.15	3089.16	3111.14	1545.08	1030.39
dN	dF-TRF		1	3	3	3	2					3104.15	3105.15	3127.14	1553.08	1035.72
dN	trF-TR		1	3	3	3	2					3104.15	3105.15	3127.14	1553.08	1035.72
dN	mGa-dF-BIBSF		1	3	3	3	2					3104.15	3105.15	3127.14	1553.08	1035.72
dN	mGa-dF-TR		1	4	3	2	2					3120.14	3121.15	3143.13	1561.08	1041.05
dN	mGa-mF-TRF		1	4	3	2	2					3120.14	3121.15	3143.13	1561.08	1041.05
dN	dGa-dF-BIBS		1	4	3	2	2					3120.14	3121.15	3143.13	1561.08	1041.05
dN	dGa-mF-BIBSF		1	4	3	2	2					3120.14	3121.15	3143.13	1561.08	1041.05
dN	mF-dLnM5BSF		1	4	3	2	2					3120.14	3121.15	3143.13	1561.08	1041.05

dN	trF-TRBSF-2G		1	1	4	4	2					3129.18	**3130.19**	**3152.17**	**1565.60**	**1044.07**
dN	trF-BI(mLdn)BSF		1	1	4	4	2					3129.18	**3130.19**	**3152.17**	**1565.60**	**1044.07**
dN	dGa-TRF		1	5	3	1	2					3136.14	**3137.14**	**3159.13**	**1569.08**	**1046.39**
dN	dGa-mF-TR		1	5	3	1	2					3136.14	**3137.14**	**3159.13**	**1569.08**	**1046.39**
dN	trLdnM3BSF		1		7	1	2					3138.19	**3139.20**	**3161.18**	**1570.10**	**1047.07**
dN	dF-TRBSF-G		1	2	4	3	2					3145.17	**3146.18**	**3168.16**	**1573.59**	**1049.40**
dN	dF-LdnBIF		1	2	4	3	2					3145.17	**3146.18**	**3168.16**	**1573.59**	**1049.40**
dN	trGa-TR		1	6	3		2					3152.13	**3153.14**	**3175.12**	**1577.07**	**1051.72**
dN	mF-TRBSF, diF-TRBS		1	3	4	2	2					3161.17	**3162.18**	**3184.16**	**1581.59**	**1054.73**
dN	trF-AG1234BSF		1		5	4	2					3170.21	**3171.21**	**3193.19**	**1586.11**	**1057.74**
dN	trF-BI(dLdn)BSF		1		5	4	2					3170.21	**3171.21**	**3193.19**	**1586.11**	**1057.74**
dN	TEF		1	4	4	1	2					3177.16	**3178.17**	**3200.15**	**1589.59**	**1060.06**
dN	mGa-TRBSF		1	4	4	1	2					3177.16	**3178.17**	**3200.15**	**1589.59**	**1060.06**
dN	mGa-mF-TRBS		1	4	4	1	2					3177.16	**3178.17**	**3200.15**	**1589.59**	**1060.06**
dN	mF-dLn-LdnM5A		1	4	4	1	2					3177.16	**3178.17**	**3200.15**	**1589.59**	**1060.06**
dN	dF-TR(dLdn)F		1	1	5	3	2					3186.20	**3187.21**	**3209.19**	**1594.11**	**1063.07**
dN	dGa-TRBS		1	5	4		2					3193.16	**3194.17**	**3216.15**	**1597.59**	**1065.39**
dN	TE(teLdn)		1		8		2					3195.21	**3196.22**	**3218.20**	**1598.61**	**1066.08**
dN	dF-TR(trLdn)F		1		6	3	2					3227.23	**3228.23**	**3250.22**	**1614.62**	**1076.75**
dN	TEBS		1	4	5	0	2					3234.18	**3235.19**	**3257.17**	**1618.10**	**1079.07**
dN	trF-TRF		1	3	3	4	2					3250.21	**3251.21**	**3273.19**	**1626.11**	**1084.41**
dN	mGa-trF-TR		1	4	3	3	2					3266.20	**3267.21**	**3289.19**	**1634.11**	**1089.74**
dN	mGa-dF-TRF		1	4	3	3	2					3266.20	**3267.21**	**3289.19**	**1634.11**	**1089.74**
dN	dGa-diF-BIBSF		1	4	3	3	2					3266.20	**3267.21**	**3289.19**	**1634.11**	**1089.74**
dN	dGa-diF-TR		1	5	3	2	2					3282.19	**3283.20**	**3305.18**	**1642.10**	**1095.07**
dN	dGa-mF-TRF		1	5	3	2	2					3282.19	**3283.20**	**3305.18**	**1642.10**	**1095.07**
dN	mF-TR(trLdn)BSF		1		7	2	2					3284.25	**3285.26**	**3307.24**	**1643.13**	**1095.76**
dN	trF-TRBSF-G		1	2	4	4	2					3291.23	**3292.24**	**3314.22**	**1646.62**	**1098.08**
dN	trF-TR(mLdn)F		1	2	4	4	2					3291.23	**3292.24**	**3314.22**	**1646.62**	**1098.08**
dN	trGa-TRF		1	6	3	1	2					3298.19	**3299.20**	**3321.18**	**1650.10**	**1100.40**
dN	trGa-mF-TR		1	6	3	1	2					3298.19	**3299.20**	**3321.18**	**1650.10**	**1100.40**
dN	dF-TRBSF		1	3	4	3	2					3307.23	**3308.23**	**3330.22**	**1654.62**	**1103.42**
dN	trF-TRBS		1	3	4	3	2					3307.23	**3308.23**	**3330.22**	**1654.62**	**1103.42**
dN	dF-TRBSF		1	3	4	3	2					3307.23	**3308.23**	**3330.22**	**1654.62**	**1103.42**
dN	teF-AG1234BSF		1		5	5	2					3316.26	**3317.27**	**3339.25**	**1659.14**	**1106.43**
dN	mF-TEF		1	4	4	2	2					3323.22	**3324.23**	**3346.21**	**1662.62**	**1108.75**
dN	mGa-dF-TRBS		1	4	4	2	2					3323.22	**3324.23**	**3346.21**	**1662.62**	**1108.75**
dN	mGa-mF-TRBSF		1	4	4	2	2					3323.22	**3324.23**	**3346.21**	**1662.62**	**1108.75**
dN	trF-TR(dLdn)F		1	1	5	4	2					3332.26	**3333.27**	**3355.25**	**1667.14**	**1111.76**
dN	dGa-TRBSF		1	5	4	1	2					3339.22	**3340.22**	**3362.21**	**1670.62**	**1114.08**
dN	dGa-mF-TRBS		1	5	4	1	2					3339.22	**3340.22**	**3362.21**	**1670.62**	**1114.08**
dN	(teLdn)F		1		8	1	2					3341.27	**3342.28**	**3364.26**	**1671.64**	**1114.76**
dN	trGa-TRBS		1	6	4		2					3355.21	**3356.22**	**3378.20**	**1678.61**	**1119.41**
dN	trF-trLdnM3F		1		6	4	2					3373.28	**3374.29**	**3396.27**	**1687.65**	**1125.44**
dN	TEBSF		1	4	5	1	2					3380.24	**3381.25**	**3403.23**	**1691.13**	**1127.75**
dN	TR(teLdn)BS		1		9		2					3398.29	**3399.30**	**3421.28**	**1700.15**	**1133.77**
dN	mGa-trF-TRF		1	4	3	4	2					3412.26	**3413.27**	**3435.25**	**1707.14**	**1138.43**
dN	dGa-trF-TR		1	5	3	3	2					3428.25	**3429.26**	**3451.24**	**1715.13**	**1143.76**
dN	dGa-dF-TRF		1	5	3	3	2					3428.25	**3429.26**	**3451.24**	**1715.13**	**1143.76**
dN	dF-TR(trLdn)BSF		1		7	3	2					3430.31	**3431.31**	**3453.30**	**1716.16**	**1144.44**
dN	trGa-dF-TR		1	6	3	2	2					3444.25	**3445.25**	**3467.24**	**1723.13**	**1149.09**
dN	trGa-mF-TRF		1	6	3	2	2					3444.25	**3445.25**	**3467.24**	**1723.13**	**1149.09**
dN	trF-TRBSF		1	3	4	4	2					3453.28	**3454.29**	**3476.27**	**1727.65**	**1152.10**
dN	dF-TEF		1	4	4	3	2					3469.28	**3470.29**	**3492.27**	**1735.65**	**1157.43**
dN	mGa-trF-TRBS		1	4	4	3	2					3469.28	**3470.29**	**3492.27**	**1735.65**	**1157.43**
dN	mGa-dF-TRBSF		1	4	4	3	2					3469.28	**3470.29**	**3492.27**	**1735.65**	**1157.43**
dN	dGa-dF-TRBS		1	5	4	2	2					3485.27	**3486.28**	**3508.26**	**1743.64**	**1162.77**
dN	dGa-mF-TRBSF		1	5	4	2	2					3485.27	**3486.28**	**3508.26**	**1743.64**	**1162.77**
dN	mF-teLdnM3F		1		8	2	2					3487.33	**3488.33**	**3510.32**	**1744.67**	**1163.45**
dN	trGa-TRBSF		1	6	4	1	2					3501.27	**3502.28**	**3524.26**	**1751.64**	**1168.10**
dN	trGa-mF-TRBS		1	6	4	1	2					3501.27	**3502.28**	**3524.26**	**1751.64**	**1168.10**
dN	mF-TEBSF		1	4	5	2	2					3526.30	**3527.31**	**3549.29**	**1764.16**	**1176.44**
dN	TE(teLdn)BSF		1		9	1	2					3544.35	**3545.36**	**3567.34**	**1773.18**	**1182.46**
dN	dGa-trF-TRF		1	5	3	4	2					3574.31	**3575.32**	**3597.30**	**1788.16**	**1192.44**
dN	trF-TR(trLdn)BSF		1		7	4	2					3576.36	**3577.37**	**3599.35**	**1789.19**	**1193.13**
dN	trGa-trF-TR		1	6	3	3	2					3590.31	**3591.31**	**3613.29**	**1796.16**	**1197.78**
dN	trGa-dF-TRF		1	6	3	3	2					3590.31	**3591.31**	**3613.29**	**1796.16**	**1197.78**
dN	mGa-trF-TRBSF		1	4	4	4	2					3615.34	**3616.34**	**3638.33**	**1808.68**	**1206.12**
dN	dGa-trF-TRBS		1	5	4	3	2					3631.33	**3632.34**	**3654.32**	**1816.67**	**1211.45**
dN	dGa-dF-TRBSF		1	5	4	3	2					3631.33	**3632.34**	**3654.32**	**1816.67**	**1211.45**
dN	dF-TE(teLdn)F		1		8	3	2					3633.39	**3634.39**	**3656.37**	**1817.70**	**1212.14**
dN	trGa-dF-TRBS		1	6	4	2	2					3647.33	**3648.33**	**3670.32**	**1824.67**	**1216.78**
dN	trGa-mF-TRBSF		1	6	4	2	2					3647.33	**3648.33**	**3670.32**	**1824.67**	**1216.78**

dN	dF-TEBSF		1	4	5	3	2					3672.36	**3673.37**	**3695.35**	**1837.19**	**1225.13**
dN	mF-TE(teLdn)BSF		1		9	2	2					3690.41	**3691.41**	**3713.40**	**1846.21**	**1231.14**
dN	dF-dLn-dLdnM5A		1	4	6	2	2					3729.38	**3730.39**	**3752.37**	**1865.70**	**1244.13**
dN	trGa-trF-TRF		1	6	3	4	2					3736.36	**3737.37**	**3759.35**	**1869.19**	**1246.46**
dN	dGa-trF-TRBSF		1	5	4	4	2					3777.39	**3778.40**	**3800.38**	**1889.70**	**1260.14**
dN	trF-(teLdn)F		1		8	4	2					3779.44	**3780.45**	**3802.43**	**1890.73**	**1260.82**
dN	trGa-trF-TRBS		1	6	4	3	2					3793.38	**3794.39**	**3816.37**	**1897.70**	**1265.47**
dN	trGa-dF-TRBSF		1	6	4	3	2					3793.38	**3794.39**	**3816.37**	**1897.70**	**1265.47**
dN	dF-(teLdn)BSF		1		9	3	2					3836.46	**3837.47**	**3859.45**	**1919.24**	**1279.83**
dN	triGal-triF-TRBSF		1	6	4	4	2					3939.44	**3940.45**	**3962.43**	**1970.73**	**1314.15**
dN	trF-teLdnM3BSF		1		9	4	2					3982.52	**3983.53**	**4005.51**	**1992.27**	**1328.51**

	trisialo (trN) glycans	reducing end		non-reducing end				modification					*m/z*			
		PA	M3PA	Hex	HexNAc	dHex	NANA	Me	Ac	HPO_3	SO_3	MS number	+H	+Na	+2H	+3H
trN	Tn	1			1		3					1172.43	**1173.44**	**1195.42**	**587.22**	**391.82**
trN	Gn-Man	1		1	1		3					1334.49	**1335.49**	**1357.48**	**668.25**	**445.84**
trN	core1	1		1	1		3					1334.49	**1335.49**	**1357.48**	**668.25**	**445.84**
trN	Ga-Gn-F	1		1	1	1	3					1480.55	**1481.55**	**1503.53**	**741.28**	**494.52**
trN	Ln-Man	1		2	1		3					1496.54	**1497.55**	**1519.53**	**749.28**	**499.85**
trN	G-core1	1		2	1		3					1496.54	**1497.55**	**1519.53**	**749.28**	**499.85**
trN	dGn-Man	1		1	2		3					1537.57	**1538.57**	**1560.56**	**769.79**	**513.53**
trN	mF-G-core1	1		2	1	1	3					1642.60	**1643.61**	**1665.59**	**822.31**	**548.54**
trN	GnLn-Man	1		2	2		3					1699.62	**1700.63**	**1722.61**	**850.82**	**567.55**
trN	Gan-G-core1	1		2	2		3					1699.62	**1700.63**	**1722.61**	**850.82**	**567.55**
trN	G-core2	1		2	2		3					1699.62	**1700.63**	**1722.61**	**850.82**	**567.55**
trN	M1BS	1		1	3		3					1740.65	**1741.65**	**1763.64**	**871.33**	**581.22**
trN	mF-Gan-G-core1	1		2	2	1	3					1845.68	**1846.68**	**1868.67**	**923.85**	**616.23**
trN	dLn-Man	1		3	2		3					1861.67	**1862.68**	**1884.66**	**931.84**	**621.56**
trN	M1BSF	1		1	3	1	3					1886.70	**1887.71**	**1909.69**	**944.36**	**629.91**
trN	GnM2	1		2	3		3					1902.70	**1903.71**	**1925.69**	**952.36**	**635.24**
trN	M1BSdF	1		1	3	2	3					2032.76	**2033.77**	**2055.75**	**1017.39**	**678.59**
trN	GnM2F	1		2	3	1	3					2048.76	**2049.76**	**2071.75**	**1025.39**	**683.93**
trN	LnM2, AG1	1		3	3		3					2064.75	**2065.76**	**2087.74**	**1033.38**	**689.26**
trN	GnM2BS	1		2	4		3					2105.78	**2106.79**	**2128.77**	**1053.90**	**702.93**
trN	LdnM2	1		2	4		3					2105.78	**2106.79**	**2128.77**	**1053.90**	**702.93**
trN	Ac-AG1		1		1		3		1			2106.76	**2107.77**	**2129.75**	**1054.39**	**703.26**
trN	GnM2BSF			2	4	1	3					2155.77	**2156.77**	**2178.76**	**1078.89**	**719.60**
trN	mF-GnM2F	1		2	3	2	3					2194.81	**2195.82**	**2217.80**	**1098.41**	**732.61**
trN	AG1F		1		1	1	3					2210.81	**2211.82**	**2233.80**	**1106.41**	**737.94**
trN	LnM2F	1		3	3	1	3					2210.81	**2211.82**	**2233.80**	**1106.41**	**737.94**
trN	GnM4		1	1	1	0	3					2226.80	**2227.81**	**2249.79**	**1114.41**	**743.28**
trN	MO		1	1	1	0	3					2226.80	**2227.81**	**2249.79**	**1114.41**	**743.28**
trN	LdnM2F	1		2	4	1	3					2251.84	**2252.84**	**2274.83**	**1126.93**	**751.62**
trN	LdnM3, AG12, AG1BS		1		2		3					2267.83	**2268.84**	**2290.82**	**1134.92**	**756.95**
trN	LnM2BS	1		3	4		3					2267.83	**2268.84**	**2290.82**	**1134.92**	**756.95**
trN	mF-GnM2BSF			2	4	2	3					2301.83	**2302.83**	**2324.81**	**1151.92**	**768.28**
trN	LdnM2BS	1		2	5		3					2308.86	**2309.86**	**2331.85**	**1155.44**	**770.63**
trN	GnLdnM2	1		2	5		3					2308.86	**2309.86**	**2331.85**	**1155.44**	**770.63**
trN	Ac-AG12		1		2		3		1			2309.84	**2310.85**	**2332.83**	**1155.93**	**770.95**
trN	dF-GnM2F	1		2	3	3	3					2340.87	**2341.88**	**2363.86**	**1171.44**	**781.30**
trN	mF-AG1F		1		1	2	3					2356.87	**2357.87**	**2379.86**	**1179.44**	**786.63**
trN	mF-LnM2F	1		3	3	2	3					2356.87	**2357.87**	**2379.86**	**1179.44**	**786.63**
trN	MOF		1	1	1	1	3					2372.86	**2373.87**	**2395.85**	**1187.44**	**791.96**
trN	GnM4F		1	1	1	1	3					2372.86	**2373.87**	**2395.85**	**1187.44**	**791.96**
trN	mGal-MO		1	2	1		3					2388.86	**2389.86**	**2411.85**	**1195.44**	**797.29**
trN	LnM4		1	2	1	0	3					2388.86	**2389.86**	**2411.85**	**1195.44**	**797.29**
trN	GnM5		1	2	1	0	3					2388.86	**2389.86**	**2411.85**	**1195.44**	**797.29**
trN	mF-LdnF	1		2	4	2	3					2397.89	**2398.90**	**2420.88**	**1199.95**	**800.31**
trN	AG12F, AG1BSF		1		2	1	3					2413.89	**2414.90**	**2436.88**	**1207.95**	**805.64**
trN	LdnM3F		1		2	1	3					2413.89	**2414.90**	**2436.88**	**1207.95**	**805.64**
trN	LnM2BSF	1		3	4	1	3					2413.89	**2414.90**	**2436.88**	**1207.95**	**805.64**
trN	MOBS		1	1	2	0	3					2429.88	**2430.89**	**2452.87**	**1215.95**	**810.97**
trN	BI-Ga		1	1	2	0	3					2429.88	**2430.89**	**2452.87**	**1215.95**	**810.97**
trN	GnM4BS		1	1	2	0	3					2429.88	**2430.89**	**2452.87**	**1215.95**	**810.97**
trN	LdnM4		1	1	2		3					2429.88	**2430.89**	**2452.87**	**1215.95**	**810.97**
trN	dF-GnM2BSF			2	4	3	3					2447.88	**2448.89**	**2470.87**	**1224.95**	**816.97**
trN	LdnM2BSF	1		2	5	1	3					2454.92	**2455.92**	**2477.90**	**1228.46**	**819.31**
trN	GnLdnM2F	1		2	5	1	3					2454.92	**2455.92**	**2477.90**	**1228.46**	**819.31**
trN	AG123, AG12BS		1		3		3					2470.91	**2471.92**	**2493.90**	**1236.46**	**824.64**
trN	GnLdnM3, LdnM3BS		1		3		3					2470.91	**2471.92**	**2493.90**	**1236.46**	**824.64**
trN	Ac-BI-Ga		1	1	2		3		1			2471.89	**2472.90**	**2494.88**	**1236.95**	**824.97**
trN	dF-AG1F		1		1	3	3					2502.93	**2503.93**	**2525.91**	**1252.47**	**835.32**
trN	dLdnM2	1		2	6		3					2511.94	**2512.94**	**2534.93**	**1256.98**	**838.32**
trN	mF-MOF		1	1	1	2	3					2518.92	**2519.93**	**2541.91**	**1260.47**	**840.65**
trN	mF-GnM4F		1	1	1	2	3					2518.92	**2519.93**	**2541.91**	**1260.47**	**840.65**
trN	mGal-MOF		1	2	1	1	3					2534.92	**2535.92**	**2557.90**	**1268.46**	**845.98**
trN	LnM4F, GnM5F		1	2	1	1	3					2534.92	**2535.92**	**2557.90**	**1268.46**	**845.98**
trN	LnM5, GnM6		1	3	1	0	3					2550.91	**2551.92**	**2573.90**	**1276.46**	**851.31**
trN	mF-AG1BSF		1		2	2	3					2559.95	**2560.95**	**2582.94**	**1280.98**	**854.32**
trN	mF-AG12F		1		2	2	3					2559.95	**2560.95**	**2582.94**	**1280.98**	**854.32**
trN	mF-LnM2BSF	1		3	4	2	3					2559.95	**2560.95**	**2582.94**	**1280.98**	**854.32**
trN	mF-LdnM3F		1		2	2	3					2559.95	**2560.95**	**2582.94**	**1280.98**	**854.32**
trN	MOBSF		1	1	2	1	3					2575.94	**2576.95**	**2598.93**	**1288.98**	**859.65**
trN	BIF-Ga		1	1	2	1	3					2575.94	**2576.95**	**2598.93**	**1288.98**	**859.65**
trN	GnM4BSF		1	1	2	1	3					2575.94	**2576.95**	**2598.93**	**1288.98**	**859.65**

trN	LdnM4F		1	1	2	1	3					2575.94	**2576.95**	**2598.93**	**1288.98**	**859.65**
trN	BI		1	2	2	0	3					2591.94	**2592.94**	**2614.93**	**1296.98**	**864.99**
trN	mGal-MOBS		1	2	2		3					2591.94	**2592.94**	**2614.93**	**1296.98**	**864.99**
trN	mGal-BI-G		1	2	2		3					2591.94	**2592.94**	**2614.93**	**1296.98**	**864.99**
trN	LnM4BS		1	2	2	0	3					2591.94	**2592.94**	**2614.93**	**1296.98**	**864.99**
trN	GnM5BS		1	2	2	0	3					2591.94	**2592.94**	**2614.93**	**1296.98**	**864.99**
trN	GnLnM4		1	2	2		3					2591.94	**2592.94**	**2614.93**	**1296.98**	**864.99**
trN	LdnM5		1	2	2		3					2591.94	**2592.94**	**2614.93**	**1296.98**	**864.99**
trN	mF-LdnBSF	1		2	5	2	3					2600.97	**2601.98**	**2623.96**	**1301.49**	**868.00**
trN	mF-GnLdnM2F	1		2	5	2	3					2600.97	**2601.98**	**2623.96**	**1301.49**	**868.00**
trN	AG12BSF		1		3	1	3					2616.97	**2617.98**	**2639.96**	**1309.49**	**873.33**
trN	AG123F		1		3	1	3					2616.97	**2617.98**	**2639.96**	**1309.49**	**873.33**
trN	GnLdnM3F, LdnM3BSF		1		3	1	3					2616.97	**2617.98**	**2639.96**	**1309.49**	**873.33**
trN	BIBS-G		1	1	3	0	3					2632.96	**2633.97**	**2655.95**	**1317.49**	**878.66**
trN	TR-2G		1	1	3		3					2632.96	**2633.97**	**2655.95**	**1317.49**	**878.66**
trN	BI(mLdn)		1	1	3		3					2632.96	**2633.97**	**2655.95**	**1317.49**	**878.66**
trN	LdnM4BS		1	1	3		3					2632.96	**2633.97**	**2655.95**	**1317.49**	**878.66**
trN	GnLdnM4		1	1	3		3					2632.96	**2633.97**	**2655.95**	**1317.49**	**878.66**
trN	Ac-BI		1	2	2		3		1			2633.95	**2634.95**	**2656.94**	**1317.98**	**878.99**
trN	dLdnM2F	1		2	6	1	3					2657.99	**2659.00**	**2680.98**	**1330.00**	**887.01**
trN	AG123BS		1		4		3					2673.99	**2675.00**	**2696.98**	**1338.00**	**892.34**
trN	GnLdnM3BS		1		4		3					2673.99	**2675.00**	**2696.98**	**1338.00**	**892.34**
trN	BI(dLdn)		1		4		3					2673.99	**2675.00**	**2696.98**	**1338.00**	**892.34**
trN	mGal-mF-MOF		1	2	1	2	3					2680.97	**2681.98**	**2703.96**	**1341.49**	**894.66**
trN	mF-LnM4F		1	2	1	2	3					2680.97	**2681.98**	**2703.96**	**1341.49**	**894.66**
trN	mF-GnM5F		1	2	1	2	3					2680.97	**2681.98**	**2703.96**	**1341.49**	**894.66**
trN	LnM5F		1	3	1	1	3					2696.97	**2697.98**	**2719.96**	**1349.49**	**900.00**
trN	dF-AG1BSF		1		2	3	3					2706.00	**2707.01**	**2728.99**	**1354.01**	**903.01**
trN	dF-AG12F		1		2	3	3					2706.00	**2707.01**	**2728.99**	**1354.01**	**903.01**
trN	mG-LnM5		1	4	1	0	3					2712.96	**2713.97**	**2735.95**	**1357.49**	**905.33**
trN	mF-MOBSF		1	1	2	2	3					2722.00	**2723.01**	**2744.99**	**1362.01**	**908.34**
trN	mF-BIF-G		1	1	2	2	3					2722.00	**2723.01**	**2744.99**	**1362.01**	**908.34**
trN	mF-GnM4BSF		1	1	2	2	3					2722.00	**2723.01**	**2744.99**	**1362.01**	**908.34**
trN	mF-LdnM4F		1	1	2	2	3					2722.00	**2723.01**	**2744.99**	**1362.01**	**908.34**
trN	BIF, mF-BI		1	2	2	1	3					2737.99	**2739.00**	**2760.98**	**1370.00**	**913.67**
trN	mGa-MOBSF		1	2	2	1	3					2737.99	**2739.00**	**2760.98**	**1370.00**	**913.67**
trN	mGa-BIF-G		1	2	2	1	3					2737.99	**2739.00**	**2760.98**	**1370.00**	**913.67**
trN	mGa-mF-MOBS		1	2	2	1	3					2737.99	**2739.00**	**2760.98**	**1370.00**	**913.67**
trN	LnM4BSF		1	2	2	1	3					2737.99	**2739.00**	**2760.98**	**1370.00**	**913.67**
trN	GnM5BSF		1	2	2	1	3					2737.99	**2739.00**	**2760.98**	**1370.00**	**913.67**
trN	GnLnM4F		1	2	2	1	3					2737.99	**2739.00**	**2760.98**	**1370.00**	**913.67**
trN	LdnM5F		1	2	2	1	3					2737.99	**2739.00**	**2760.98**	**1370.00**	**913.67**
trN	dF-GnLdnM2F	1		2	5	3	3					2747.03	**2748.04**	**2770.02**	**1374.52**	**916.68**
trN	mGa-BI		1	3	2		3					2753.99	**2755.00**	**2776.98**	**1378.00**	**919.00**
trN	LnM5BS		1	3	2		3					2753.99	**2755.00**	**2776.98**	**1378.00**	**919.00**
trN	GnLnM5		1	3	2		3					2753.99	**2755.00**	**2776.98**	**1378.00**	**919.00**
trN	dLnM4		1	3	2		3					2753.99	**2755.00**	**2776.98**	**1378.00**	**919.00**
trN	mF-AG12BSF		1		3	2	3					2763.03	**2764.03**	**2786.02**	**1382.52**	**922.02**
trN	mF-AG123F		1		3	2	3					2763.03	**2764.03**	**2786.02**	**1382.52**	**922.02**
trN	mF-LdnM3BSF		1		3	2	3					2763.03	**2764.03**	**2786.02**	**1382.52**	**922.02**
trN	mF-LdnAG1F		1		3	2	3					2763.03	**2764.03**	**2786.02**	**1382.52**	**922.02**
trN	BIBSF-Ga		1	1	3	1	3					2779.02	**2780.03**	**2802.01**	**1390.52**	**927.35**
trN	TRF-2Ga		1	1	3	1	3					2779.02	**2780.03**	**2802.01**	**1390.52**	**927.35**
trN	BI(mLdn)F		1	1	3	1	3					2779.02	**2780.03**	**2802.01**	**1390.52**	**927.35**
trN	LdnM4BSF		1	1	3	1	3					2779.02	**2780.03**	**2802.01**	**1390.52**	**927.35**
trN	GnLdnM4F		1	1	3	1	3					2779.02	**2780.03**	**2802.01**	**1390.52**	**927.35**
trN	BIBS		1	2	3	0	3					2795.02	**2796.02**	**2818.01**	**1398.52**	**932.68**
trN	TR-Ga		1	2	3		3					2795.02	**2796.02**	**2818.01**	**1398.52**	**932.68**
trN	mGal-BIBS-Ga		1	2	3		3					2795.02	**2796.02**	**2818.01**	**1398.52**	**932.68**
trN	GnLnM4BS		1	2	3		3					2795.02	**2796.02**	**2818.01**	**1398.52**	**932.68**
trN	LdnM5BS		1	2	3		3					2795.02	**2796.02**	**2818.01**	**1398.52**	**932.68**
trN	GnLdnM5		1	2	3		3					2795.02	**2796.02**	**2818.01**	**1398.52**	**932.68**
trN	mF-dLdnM2F	1		2	6	2	3					2804.05	**2805.06**	**2827.04**	**1403.03**	**935.69**
trN	AG123BSF		1		4	1	3					2820.05	**2821.05**	**2843.04**	**1411.03**	**941.02**
trN	LdnAG1BSF		1		4	1	3					2820.05	**2821.05**	**2843.04**	**1411.03**	**941.02**
trN	BI(dLdn)F		1		4	1	3					2820.05	**2821.05**	**2843.04**	**1411.03**	**941.02**
trN	TRBS-2Ga		1	1	4		3					2836.04	**2837.05**	**2859.03**	**1419.03**	**946.35**
trN	BI(mLdn)BS		1	1	4		3					2836.04	**2837.05**	**2859.03**	**1419.03**	**946.35**
trN	GnLdnM4BS		1	1	4		3					2836.04	**2837.05**	**2859.03**	**1419.03**	**946.35**
trN	mF-LnM5F		1	3	1	2	3					2843.03	**2844.03**	**2866.02**	**1422.52**	**948.68**
trN	trF-AG12F		1		2	4	3					2852.06	**2853.07**	**2875.05**	**1427.04**	**951.69**
trN	mG-LnM5F		1	4	1	1	3					2859.02	**2860.03**	**2882.01**	**1430.52**	**954.01**
trN	dF-BIF-Ga		1	1	2	3	3					2868.06	**2869.06**	**2891.05**	**1435.04**	**957.03**

trN	AG1234BS		1		5		3					2877.07	2878.08	2900.06	1439.54	960.03
trN	BI(dLdn)BS		1		5		3					2877.07	2878.08	2900.06	1439.54	960.03
trN	mF-BIF, diF-BI		1	2	2	2	3					2884.05	2885.06	2907.04	1443.03	962.36
trN	mGa-mF-MOBSF		1	2	2	2	3					2884.05	2885.06	2907.04	1443.03	962.36
trN	mGa-mF-BIF-Ga		1	2	2	2	3					2884.05	2885.06	2907.04	1443.03	962.36
trN	mF-LnM4BSF		1	2	2	2	3					2884.05	2885.06	2907.04	1443.03	962.36
trN	mF-GnM5BSF		1	2	2	2	3					2884.05	2885.06	2907.04	1443.03	962.36
trN	mF-GnLnM4F		1	2	2	2	3					2884.05	2885.06	2907.04	1443.03	962.36
trN	mF-LdnM5F		1	2	2	2	3					2884.05	2885.06	2907.04	1443.03	962.36
trN	trF-GnLdnM2F	1		2	5	4	3					2893.09	2894.10	2916.08	1447.55	965.37
trN	mGa-BIF		1	3	2	1	3					2900.05	2901.05	2923.04	1451.03	967.69
trN	mGa-mF-BI		1	3	2	1	3					2900.05	2901.05	2923.04	1451.03	967.69
trN	LnM5BSF		1	3	2	1	3					2900.05	2901.05	2923.04	1451.03	967.69
trN	GnLnM5F		1	3	2	1	3					2900.05	2901.05	2923.04	1451.03	967.69
trN	dLnM4F		1	3	2	1	3					2900.05	2901.05	2923.04	1451.03	967.69
trN	dF-AG12BSF		1		3	3	3					2909.08	2910.09	2932.07	1455.55	970.70
trN	dF-AG123F		1		3	3	3					2909.08	2910.09	2932.07	1455.55	970.70
trN	dF-GnLdnM3F		1		3	3	3					2909.08	2910.09	2932.07	1455.55	970.70
trN	dGa-BI		1	4	2		3					2916.04	2917.05	2939.03	1459.03	973.02
trN	mG-LnM5BS		1	4	2	0	3					2916.04	2917.05	2939.03	1459.03	973.02
trN	dLnM5		1	4	2		3					2916.04	2917.05	2939.03	1459.03	973.02
trN	mF-BIBSF-G		1	1	3	2	3					2925.08	2926.09	2948.07	1463.55	976.03
trN	mF-TRF-2G		1	1	3	2	3					2925.08	2926.09	2948.07	1463.55	976.03
trN	mF-BI(mLdn)F		1	1	3	2	3					2925.08	2926.09	2948.07	1463.55	976.03
trN	mF-LdnM4BSF		1	1	3	2	3					2925.08	2926.09	2948.07	1463.55	976.03
trN	mF-GnLdnM4F		1	1	3	2	3					2925.08	2926.09	2948.07	1463.55	976.03
trN	BIBSF		1	2	3	1	3					2941.07	2942.08	2964.06	1471.54	981.37
trN	TRF-Ga		1	2	3	1	3					2941.07	2942.08	2964.06	1471.54	981.37
trN	mGa-BIBSF-Ga		1	2	3	1	3					2941.07	2942.08	2964.06	1471.54	981.37
trN	mGa-mF-BIBS-Ga		1	2	3	1	3					2941.07	2942.08	2964.06	1471.54	981.37
trN	mF-BIBS		1	2	3	1	3					2941.07	2942.08	2964.06	1471.54	981.37
trN	GnLnM4BSF		1	2	3	1	3					2941.07	2942.08	2964.06	1471.54	981.37
trN	LdnM5BSF		1	2	3	1	3					2941.07	2942.08	2964.06	1471.54	981.37
trN	GnLdnM5F		1	2	3	1	3					2941.07	2942.08	2964.06	1471.54	981.37
trN	dF-dLdnM2F	1		2	6	3	3					2950.11	2951.12	2973.10	1476.06	984.38
trN	TR		1	3	3	0	3					2957.07	2958.08	2980.06	1479.54	986.70
trN	mGa-BIBS		1	3	3		3					2957.07	2958.08	2980.06	1479.54	986.70
trN	GnLnM5BS		1	3	3		3					2957.07	2958.08	2980.06	1479.54	986.70
trN	dLnM4BS		1	3	3		3					2957.07	2958.08	2980.06	1479.54	986.70
trN	mF-AG123BSF		1		4	2	3					2966.11	2967.11	2989.09	1484.06	989.71
trN	mF-LdnAG1BSF		1		4	2	3					2966.11	2967.11	2989.09	1484.06	989.71
trN	mF-dLdnM3F		1		4	2	3					2966.11	2967.11	2989.09	1484.06	989.71
trN	TRBSF-2G		1	1	4	1	3					2982.10	2983.11	3005.09	1492.06	995.04
trN	BI(mLdn)BSF		1	1	4	1	3					2982.10	2983.11	3005.09	1492.06	995.04
trN	GnLdnM4BSF		1	1	4	1	3					2982.10	2983.11	3005.09	1492.06	995.04
trN	TRBS-G		1	2	4	0	3					2998.10	2999.10	3021.08	1500.05	1000.37
trN	TR(mLdn)		1	2	4		3					2998.10	2999.10	3021.08	1500.05	1000.37
trN	GnLdnM5BS		1	2	4		3					2998.10	2999.10	3021.08	1500.05	1000.37
trN	AG1234BSF		1		5	1	3					3023.13	3024.13	3046.12	1512.57	1008.72
trN	BI(dLdn)BSF		1		5	1	3					3023.13	3024.13	3046.12	1512.57	1008.72
trN	dF-BIF		1	2	2	3	3					3030.11	3031.12	3053.10	1516.06	1011.04
trN	TR(dLdn)		1	1	5		3					3039.12	3040.13	3062.11	1520.57	1014.05
trN	mGa-dF-BI		1	3	2	2	3					3046.11	3047.11	3069.09	1524.06	1016.38
trN	mGa-mF-BIF		1	3	2	2	3					3046.11	3047.11	3069.09	1524.06	1016.38
trN	mF-LnM5BSF		1	3	2	2	3					3046.11	3047.11	3069.09	1524.06	1016.38
trN	mF-GnLnM5F		1	3	2	2	3					3046.11	3047.11	3069.09	1524.06	1016.38
trN	mF-dLnM4F		1	3	2	2	3					3046.11	3047.11	3069.09	1524.06	1016.38
trN	trF-AG12BSF		1		3	4	3					3055.14	3056.15	3078.13	1528.58	1019.39
trN	trF-AG123F		1		3	4	3					3055.14	3056.15	3078.13	1528.58	1019.39
trN	trF-LdnAG1F		1		3	4	3					3055.14	3056.15	3078.13	1528.58	1019.39
trN	dGa-BIF, dGa-mF-BI		1	4	2	1	3					3062.10	3063.11	3085.09	1532.06	1021.71
trN	mGa-LnM5BSF		1	4	2	1	3					3062.10	3063.11	3085.09	1532.06	1021.71
trN	dLnM5F		1	4	2	1	3					3062.10	3063.11	3085.09	1532.06	1021.71
trN	dF-BIBSF-G		1	1	3	3	3					3071.14	3072.14	3094.13	1536.58	1024.72
trN	dF-TRF-2G		1	1	3	3	3					3071.14	3072.14	3094.13	1536.58	1024.72
trN	dF-BI(mLdn)F		1	1	3	3	3					3071.14	3072.14	3094.13	1536.58	1024.72
trN	TR(trLdn)		1		6		3					3080.15	3081.16	3103.14	1541.08	1027.72
trN	mF-BIBSF		1	2	3	2	3					3087.13	3088.14	3110.12	1544.57	1030.05
trN	mF-TRF-G		1	2	3	2	3					3087.13	3088.14	3110.12	1544.57	1030.05
trN	mGa-mF-BIBSF-G		1	2	3	2	3					3087.13	3088.14	3110.12	1544.57	1030.05
trN	dF-BIBS, mF-BIBSF		1	2	3	2	3					3087.13	3088.14	3110.12	1544.57	1030.05
trN	mF-GnLnM4BSF		1	2	3	2	3					3087.13	3088.14	3110.12	1544.57	1030.05
trN	mF-LdnM5BSF		1	2	3	2	3					3087.13	3088.14	3110.12	1544.57	1030.05

trN	mF-GnLdnM5F		1	2	3	2	3					3087.13	**3088.14**	**3110.12**	**1544.57**	**1030.05**
trN	trF-dLdnM2F	1		2	6	4	3					3096.17	**3097.18**	**3119.16**	**1549.09**	**1033.06**
trN	TRF, mF-TR		1	3	3	1	3					3103.13	**3104.13**	**3126.12**	**1552.57**	**1035.38**
trN	mGa-BIBSF		1	3	3	1	3					3103.13	**3104.13**	**3126.12**	**1552.57**	**1035.38**
trN	mGal-mF-BIBS		1	3	3	1	3					3103.13	**3104.13**	**3126.12**	**1552.57**	**1035.38**
trN	GnLnM5BSF		1	3	3	1	3					3103.13	**3104.13**	**3126.12**	**1552.57**	**1035.38**
trN	diLnM4BSF		1	3	3	1	3					3103.13	**3104.13**	**3126.12**	**1552.57**	**1035.38**
trN	dF-AG123BSF		1		4	3	3					3112.16	**3113.17**	**3135.15**	**1557.09**	**1038.40**
trN	dF-LdnAG1BSF		1		4	3	3					3112.16	**3113.17**	**3135.15**	**1557.09**	**1038.40**
trN	dF-dLdnM3F		1		4	3	3					3112.16	**3113.17**	**3135.15**	**1557.09**	**1038.40**
trN	mGa-TR, dGa-BIBS		1	4	3		3					3119.12	**3120.13**	**3142.11**	**1560.57**	**1040.71**
trN	dLnM5BS		1	4	3		3					3119.12	**3120.13**	**3142.11**	**1560.57**	**1040.71**
trN	mF-TRBSF-2G		1	1	4	2	3					3128.16	**3129.17**	**3151.15**	**1565.09**	**1043.73**
trN	mF-LdnMOBSF		1	1	4	2	3					3128.16	**3129.17**	**3151.15**	**1565.09**	**1043.73**
trN	mF-GnLdnM4BSF		1	1	4	2	3					3128.16	**3129.17**	**3151.15**	**1565.09**	**1043.73**
trN	TRBSF-G		1	2	4	1	3					3144.15	**3145.16**	**3167.14**	**1573.08**	**1049.06**
trN	TR(mLdn)F		1	2	4	1	3					3144.15	**3145.16**	**3167.14**	**1573.08**	**1049.06**
trN	GnLdnM5BSF		1	2	4	1	3					3144.15	**3145.16**	**3167.14**	**1573.08**	**1049.06**
trN	TRBS		1	3	4	0	3					3160.15	**3161.16**	**3183.14**	**1581.08**	**1054.39**
trN	mF-AG1234BSF		1		5	2	3					3169.18	**3170.19**	**3192.17**	**1585.60**	**1057.40**
trN	mF-dLdnM3BSF		1		5	2	3					3169.18	**3170.19**	**3192.17**	**1585.60**	**1057.40**
trN	TR(dLdn)F		1	1	5	1	3					3185.18	**3186.19**	**3208.17**	**1593.60**	**1062.73**
trN	mGa-dF-BIF		1	3	2	3	3					3192.16	**3193.17**	**3215.15**	**1597.09**	**1065.06**
trN	dGa-dF-BI		1	4	2	2	3					3208.16	**3209.17**	**3231.15**	**1605.09**	**1070.39**
trN	dGa-mF-BIF		1	4	2	2	3					3208.16	**3209.17**	**3231.15**	**1605.09**	**1070.39**
trN	mF-diLnM5F		1	4	2	2	3					3208.16	**3209.17**	**3231.15**	**1605.09**	**1070.39**
trN	trF-TRF-2G		1	1	3	4	3					3217.19	**3218.20**	**3240.18**	**1609.60**	**1073.41**
trN	trF-LdnMOF		1	1	3	4	3					3217.19	**3218.20**	**3240.18**	**1609.60**	**1073.41**
trN	TR(trLdn)F		1		6	1	3					3226.21	**3227.21**	**3249.20**	**1614.11**	**1076.41**
trN	dF-BIBSF		1	2	3	3	3					3233.19	**3234.20**	**3256.18**	**1617.60**	**1078.74**
trN	dF-TRF-Ga		1	2	3	3	3					3233.19	**3234.20**	**3256.18**	**1617.60**	**1078.74**
trN	dF-TR		1	3	3	2	3					3249.18	**3250.19**	**3272.17**	**1625.60**	**1084.07**
trN	mF-TRF		1	3	3	2	3					3249.18	**3250.19**	**3272.17**	**1625.60**	**1084.07**
trN	mGa-dF-BIBS		1	3	3	2	3					3249.18	**3250.19**	**3272.17**	**1625.60**	**1084.07**
trN	mGa-mF-BIBSF		1	3	3	2	3					3249.18	**3250.19**	**3272.17**	**1625.60**	**1084.07**
trN	mF-GnLnM5BSF		1	3	3	2	3					3249.18	**3250.19**	**3272.17**	**1625.60**	**1084.07**
trN	mF-dLnM4BSF		1	3	3	2	3					3249.18	**3250.19**	**3272.17**	**1625.60**	**1084.07**
trN	Ac-dG-dF-BI		1	4	2	2	3		1			3250.17	**3251.18**	**3273.16**	**1626.09**	**1084.40**
trN	trF-AG123BSF		1		4	4	3					3258.22	**3259.23**	**3281.21**	**1630.12**	**1087.08**
trN	trF-GnLdnM3BSF		1		4	4	3					3258.22	**3259.23**	**3281.21**	**1630.12**	**1087.08**
trN	trF-BI(dLdn)F		1		4	4	3					3258.22	**3259.23**	**3281.21**	**1630.12**	**1087.08**
trN	mGa-TRF		1	4	3	1	3					3265.18	**3266.19**	**3288.17**	**1633.60**	**1089.40**
trN	dGa-BIBSF		1	4	3	1	3					3265.18	**3266.19**	**3288.17**	**1633.60**	**1089.40**
trN	mGa-mF-TR		1	4	3	1	3					3265.18	**3266.19**	**3288.17**	**1633.60**	**1089.40**
trN	dGa-mF-BIBS		1	4	3	1	3					3265.18	**3266.19**	**3288.17**	**1633.60**	**1089.40**
trN	dLnM5BSF		1	4	3	1	3					3265.18	**3266.19**	**3288.17**	**1633.60**	**1089.40**
trN	dF-TRBSF-2G		1	1	4	3	3					3274.22	**3275.22**	**3297.21**	**1638.12**	**1092.41**
trN	dF-BI(mLdn)BSF		1	1	4	3	3					3274.22	**3275.22**	**3297.21**	**1638.12**	**1092.41**
trN	dGa-TR		1	5	3		3					3281.17	**3282.18**	**3304.16**	**1641.59**	**1094.73**
trN	TR(trLdn)BS		1		7		3					3283.23	**3284.24**	**3306.22**	**1642.62**	**1095.42**
trN	mF-TRBSF-Ga		1	2	4	2	3					3290.21	**3291.22**	**3313.20**	**1646.11**	**1097.74**
trN	mF-LdnBIF		1	2	4	2	3					3290.21	**3291.22**	**3313.20**	**1646.11**	**1097.74**
trN	mF-GnLdnM5BSF		1	2	4	2	3					3290.21	**3291.22**	**3313.20**	**1646.11**	**1097.74**
trN	TRBSF		1	3	4	1	3					3306.21	**3307.21**	**3329.20**	**1654.11**	**1103.08**
trN	mF-TRBS		1	3	4	1	3					3306.21	**3307.21**	**3329.20**	**1654.11**	**1103.08**
trN	dF-AG1234BSF		1		5	3	3					3315.24	**3316.25**	**3338.23**	**1658.63**	**1106.09**
trN	dF-dLdnM3BSF		1		5	3	3					3315.24	**3316.25**	**3338.23**	**1658.63**	**1106.09**
trN	TE		1	4	4	0	3					3322.20	**3323.21**	**3345.19**	**1662.11**	**1108.41**
trN	mGal-TRBS		1	4	4		3					3322.20	**3323.21**	**3345.19**	**1662.11**	**1108.41**
trN	dLn-LdnM5A		1	4	4		3					3322.20	**3323.21**	**3345.19**	**1662.11**	**1108.41**
trN	mF-TR(dLdn)F		1	1	5	2	3					3331.24	**3332.24**	**3354.23**	**1666.63**	**1111.42**
trN	diGal-diF-BIF		1	4	2	3	3					3354.22	**3355.22**	**3377.21**	**1678.12**	**1119.08**
trN	mF-trLdnM3F		1		6	2	3					3372.26	**3373.27**	**3395.25**	**1687.14**	**1125.10**
trN	trF-TRF-Ga		1	2	3	4	3					3379.25	**3380.25**	**3402.24**	**1690.63**	**1127.42**
trN	dF-TRF		1	3	3	3	3					3395.24	**3396.25**	**3418.23**	**1698.63**	**1132.75**
trN	trF-TR		1	3	3	3	3					3395.24	**3396.25**	**3418.23**	**1698.63**	**1132.75**
trN	mGa-dF-BIBSF		1	3	3	3	3					3395.24	**3396.25**	**3418.23**	**1698.63**	**1132.75**
trN	mGa-dF-TR		1	4	3	2	3					3411.24	**3412.24**	**3434.23**	**1706.63**	**1138.09**
trN	mGa-mF-TRF		1	4	3	2	3					3411.24	**3412.24**	**3434.23**	**1706.63**	**1138.09**
trN	dGa-dF-BIBS		1	4	3	2	3					3411.24	**3412.24**	**3434.23**	**1706.63**	**1138.09**
trN	dGa-mF-BIBSF		1	4	3	2	3					3411.24	**3412.24**	**3434.23**	**1706.63**	**1138.09**
trN	mF-dLnM5BSF		1	4	3	2	3					3411.24	**3412.24**	**3434.23**	**1706.63**	**1138.09**
trN	trF-TRBSF-2G		1	1	4	4	3					3420.27	**3421.28**	**3443.26**	**1711.14**	**1141.10**

trN	trF-BI(mLdn)BSF		1	1	4	4	3					3420.27	**3421.28**	**3443.26**	**1711.14**	**1141.10**
trN	dGa-TRF		1	5	3	1	3					3427.23	**3428.24**	**3450.22**	**1714.62**	**1143.42**
trN	dGa-mF-TR		1	5	3	1	3					3427.23	**3428.24**	**3450.22**	**1714.62**	**1143.42**
trN	trLdnM3BSF		1		7	1	3					3429.29	**3430.29**	**3452.27**	**1715.65**	**1144.10**
trN	dF-TRBSF-G		1	2	4	3	3					3436.27	**3437.28**	**3459.26**	**1719.14**	**1146.43**
trN	dF-LdnBIF		1	2	4	3	3					3436.27	**3437.28**	**3459.26**	**1719.14**	**1146.43**
trN	trGa-TR		1	6	3		3					3443.23	**3444.23**	**3466.22**	**1722.62**	**1148.75**
trN	mF-TRBSF, diF-TRBS		1	3	4	2	3					3452.26	**3453.27**	**3475.25**	**1727.14**	**1151.76**
trN	trF-AG1234BSF		1		5	4	3					3461.30	**3462.31**	**3484.29**	**1731.66**	**1154.77**
trN	trF-BI(dLdn)BSF		1		5	4	3					3461.30	**3462.31**	**3484.29**	**1731.66**	**1154.77**
trN	TEF		1	4	4	1	3					3468.26	**3469.27**	**3491.25**	**1735.14**	**1157.09**
trN	mGa-TRBSF		1	4	4	1	3					3468.26	**3469.27**	**3491.25**	**1735.14**	**1157.09**
trN	mGa-mF-TRBS		1	4	4	1	3					3468.26	**3469.27**	**3491.25**	**1735.14**	**1157.09**
trN	mF-dLn-LdnM5A		1	4	4	1	3					3468.26	**3469.27**	**3491.25**	**1735.14**	**1157.09**
trN	dF-TR(dLdn)F		1	1	5	3	3					3477.30	**3478.30**	**3500.28**	**1739.66**	**1160.11**
trN	dGa-TRBS		1	5	4		3					3484.25	**3485.26**	**3507.24**	**1743.13**	**1162.43**
trN	TE(teLdn)		1		8		3					3486.31	**3487.31**	**3509.30**	**1744.16**	**1163.11**
trN	dF-TR(trLdn)F		1		6	3	3					3518.32	**3519.33**	**3541.31**	**1760.17**	**1173.78**
trN	TEBS		1	4	5	0	3					3525.28	**3526.29**	**3548.27**	**1763.65**	**1176.10**
trN	trF-TRF		1	3	3	4	3					3541.30	**3542.31**	**3564.29**	**1771.66**	**1181.44**
trN	mGa-trF-TR		1	4	3	3	3					3557.30	**3558.30**	**3580.28**	**1779.65**	**1186.77**
trN	mGa-dF-TRF		1	4	3	3	3					3557.30	**3558.30**	**3580.28**	**1779.65**	**1186.77**
trN	dGa-diF-BIBSF		1	4	3	3	3					3557.30	**3558.30**	**3580.28**	**1779.65**	**1186.77**
trN	dGa-diF-TR		1	5	3	2	3					3573.29	**3574.30**	**3596.28**	**1787.65**	**1192.10**
trN	dGa-mF-TRF		1	5	3	2	3					3573.29	**3574.30**	**3596.28**	**1787.65**	**1192.10**
trN	mF-TR(trLdn)BSF		1		7	2	3					3575.34	**3576.35**	**3598.33**	**1788.68**	**1192.79**
trN	trF-TRBSF-G		1	2	4	4	3					3582.33	**3583.33**	**3605.32**	**1792.17**	**1195.12**
trN	trF-TR(mLdn)F		1	2	4	4	3					3582.33	**3583.33**	**3605.32**	**1792.17**	**1195.12**
trN	trGa-TRF		1	6	3	1	3					3589.29	**3590.29**	**3612.27**	**1795.65**	**1197.44**
trN	trGa-mF-TR		1	6	3	1	3					3589.29	**3590.29**	**3612.27**	**1795.65**	**1197.44**
trN	dF-TRBSF		1	3	4	3	3					3598.32	**3599.33**	**3621.31**	**1800.17**	**1200.45**
trN	trF-TRBS		1	3	4	3	3					3598.32	**3599.33**	**3621.31**	**1800.17**	**1200.45**
trN	dF-TRBSF		1	3	4	3	3					3598.32	**3599.33**	**3621.31**	**1800.17**	**1200.45**
trN	teF-AG1234BSF		1		5	5	3					3607.36	**3608.37**	**3630.35**	**1804.69**	**1203.46**
trN	mF-TEF		1	4	4	2	3					3614.32	**3615.32**	**3637.31**	**1808.17**	**1205.78**
trN	mGa-dF-TRBS		1	4	4	2	3					3614.32	**3615.32**	**3637.31**	**1808.17**	**1205.78**
trN	mGa-mF-TRBSF		1	4	4	2	3					3614.32	**3615.32**	**3637.31**	**1808.17**	**1205.78**
trN	trF-TR(dLdn)F		1	1	5	4	3					3623.35	**3624.36**	**3646.34**	**1812.68**	**1208.79**
trN	dGa-TRBSF		1	5	4	1	3					3630.31	**3631.32**	**3653.30**	**1816.16**	**1211.11**
trN	dGa-mF-TRBS		1	5	4	1	3					3630.31	**3631.32**	**3653.30**	**1816.16**	**1211.11**
trN	(teLdn)F		1		8	1	3					3632.37	**3633.37**	**3655.35**	**1817.19**	**1211.80**
trN	trGa-TRBS		1	6	4		3					3646.31	**3647.31**	**3669.30**	**1824.16**	**1216.44**
trN	trF-trLdnM3F		1		6	4	3					3664.38	**3665.39**	**3687.37**	**1833.20**	**1222.47**
trN	TEBSF		1	4	5	1	3					3671.34	**3672.35**	**3694.33**	**1836.68**	**1224.79**
trN	TR(teLdn)BS		1		9		3					3689.39	**3690.39**	**3712.38**	**1845.70**	**1230.80**
trN	mGa-trF-TRF		1	4	3	4	3					3703.35	**3704.36**	**3726.34**	**1852.68**	**1235.46**
trN	dGa-trF-TR		1	5	3	3	3					3719.35	**3720.36**	**3742.34**	**1860.68**	**1240.79**
trN	dGa-dF-TRF		1	5	3	3	3					3719.35	**3720.36**	**3742.34**	**1860.68**	**1240.79**
trN	dF-TR(trLdn)BSF		1		7	3	3					3721.40	**3722.41**	**3744.39**	**1861.71**	**1241.47**
trN	trGa-dF-TR		1	6	3	2	3					3735.34	**3736.35**	**3758.33**	**1868.68**	**1246.12**
trN	trGa-mF-TRF		1	6	3	2	3					3735.34	**3736.35**	**3758.33**	**1868.68**	**1246.12**
trN	trF-TRBSF		1	3	4	4	3					3744.38	**3745.39**	**3767.37**	**1873.20**	**1249.13**
trN	dF-TEF		1	4	4	3	3					3760.37	**3761.38**	**3783.36**	**1881.19**	**1254.47**
trN	mGa-trF-TRBS		1	4	4	3	3					3760.37	**3761.38**	**3783.36**	**1881.19**	**1254.47**
trN	mGa-dF-TRBSF		1	4	4	3	3					3760.37	**3761.38**	**3783.36**	**1881.19**	**1254.47**
trN	dGa-dF-TRBS		1	5	4	2	3					3776.37	**3777.38**	**3799.36**	**1889.19**	**1259.80**
trN	dGa-mF-TRBSF		1	5	4	2	3					3776.37	**3777.38**	**3799.36**	**1889.19**	**1259.80**
trN	mF-teLdnM3F		1		8	2	3					3778.42	**3779.43**	**3801.41**	**1890.22**	**1260.48**
trN	trGa-TRBSF		1	6	4	1	3					3792.36	**3793.37**	**3815.35**	**1897.19**	**1265.13**
trN	trGa-mF-TRBS		1	6	4	1	3					3792.36	**3793.37**	**3815.35**	**1897.19**	**1265.13**
trN	mF-TEBSF		1	4	5	2	3					3817.40	**3818.40**	**3840.39**	**1909.71**	**1273.47**
trN	TE(teLdn)BSF		1		9	1	3					3835.44	**3836.45**	**3858.43**	**1918.73**	**1279.49**
trN	dGa-trF-TRF		1	5	3	4	3					3865.41	**3866.41**	**3888.40**	**1933.71**	**1289.48**
trN	trF-TR(trLdn)BSF		1		7	4	3					3867.46	**3868.47**	**3890.45**	**1934.74**	**1290.16**
trN	trGa-trF-TR		1	6	3	3	3					3881.40	**3882.41**	**3904.39**	**1941.71**	**1294.81**
trN	trGa-dF-TRF		1	6	3	3	3					3881.40	**3882.41**	**3904.39**	**1941.71**	**1294.81**
trN	mGa-trF-TRBSF		1	4	4	4	3					3906.43	**3907.44**	**3929.42**	**1954.22**	**1303.15**
trN	dGa-trF-TRBS		1	5	4	3	3					3922.43	**3923.43**	**3945.42**	**1962.22**	**1308.48**
trN	dGa-dF-TRBSF		1	5	4	3	3					3922.43	**3923.43**	**3945.42**	**1962.22**	**1308.48**
trN	dF-TE(teLdn)F		1		8	3	3					3924.48	**3925.49**	**3947.47**	**1963.25**	**1309.17**
trN	trGa-dF-TRBS		1	6	4	2	3					3938.42	**3939.43**	**3961.41**	**1970.22**	**1313.81**
trN	trGa-mF-TRBSF		1	6	4	2	3					3938.42	**3939.43**	**3961.41**	**1970.22**	**1313.81**
trN	dF-TEBSF		1	4	5	3	3					3963.45	**3964.46**	**3986.44**	**1982.73**	**1322.16**

trN	mF-TE(teLdn)BSF		1		9	2	3					3981.50	**3982.51**	**4004.49**	**1991.76**	**1328.17**
trN	dF-dLn-dLdnM5A		1	4	6	2	3					4020.48	**4021.48**	**4043.46**	**2011.25**	**1341.17**
trN	trGa-trF-TRF		1	6	3	4	3					4027.46	**4028.47**	**4050.45**	**2014.74**	**1343.49**
trN	dGa-trF-TRBSF		1	5	4	4	3					4068.49	**4069.49**	**4091.47**	**2035.25**	**1357.17**
trN	trF-(teLdn)F		1		8	4	3					4070.54	**4071.55**	**4093.53**	**2036.28**	**1357.85**
trN	trGa-trF-TRBS		1	6	4	3	3					4084.48	**4085.49**	**4107.47**	**2043.25**	**1362.50**
trN	trGa-dF-TRBSF		1	6	4	3	3					4084.48	**4085.49**	**4107.47**	**2043.25**	**1362.50**
trN	dF-(teLdn)BSF		1		9	3	3					4127.56	**4128.57**	**4150.55**	**2064.79**	**1376.86**
trN	triGal-triF-TRBSF		1	6	4	4	3					4230.54	**4231.55**	**4253.53**	**2116.28**	**1411.19**
trN	trF-teLdnM3BSF		1		9	4	3					4273.62	**4274.63**	**4296.61**	**2137.82**	**1425.55**

**tetrasialo (TeN) glycans**		reducing end		non-reducing end				modification					*m/z*			
		PA	M3PA	Hex	HexNAc	dHex	NANA	Me	Ac	HPO_3	SO_3	MS number	**+H**	**+Na**	**+2H**	**+3H**
TeN	GnM2	1		2	3		4					2193.79	**2194.80**	**2216.78**	**1097.90**	**732.27**
TeN	M1BSdF	1		1	3	2	4					2323.86	**2324.86**	**2346.85**	**1162.94**	**775.63**
TeN	GnM2F	1		2	3	1	4					2339.85	**2340.86**	**2362.84**	**1170.93**	**780.96**
TeN	LnM2, AG1	1		3	3		4					2355.85	**2356.85**	**2378.84**	**1178.93**	**786.29**
TeN	GnM2BS	1		2	4		4					2396.87	**2397.88**	**2419.86**	**1199.44**	**799.97**
TeN	LdnM2	1		2	4		4					2396.87	**2397.88**	**2419.86**	**1199.44**	**799.97**
TeN	Ac-AG1		1		1		4		1			2397.86	**2398.86**	**2420.85**	**1199.94**	**800.29**
TeN	GnM2BSF			2	4	1	4					2446.86	**2447.87**	**2469.85**	**1224.44**	**816.63**
TeN	mF-GnM2F	1		2	3	2	4					2485.91	**2486.92**	**2508.90**	**1243.96**	**829.64**
TeN	AG1F		1		1	1	4					2501.90	**2502.91**	**2524.89**	**1251.96**	**834.98**
TeN	LnM2F	1		3	3	1	4					2501.90	**2502.91**	**2524.89**	**1251.96**	**834.98**
TeN	GnM4		1	1	1	0	4					2517.90	**2518.91**	**2540.89**	**1259.96**	**840.31**
TeN	MO		1	1	1	0	4					2517.90	**2518.91**	**2540.89**	**1259.96**	**840.31**
TeN	LdnM2F	1		2	4	1	4					2542.93	**2543.94**	**2565.92**	**1272.47**	**848.65**
TeN	LdnM3, AG12, AG1BS		1		2		4					2558.93	**2559.93**	**2581.92**	**1280.47**	**853.98**
TeN	LnM2BS	1		3	4		4					2558.93	**2559.93**	**2581.92**	**1280.47**	**853.98**
TeN	mF-GnM2BSF			2	4	2	4					2592.92	**2593.93**	**2615.91**	**1297.47**	**865.31**
TeN	LdnM2BS	1		2	5		4					2599.95	**2600.96**	**2622.94**	**1300.98**	**867.66**
TeN	GnLdnM2	1		2	5		4					2599.95	**2600.96**	**2622.94**	**1300.98**	**867.66**
TeN	Ac-AG12		1		2		4		1			2600.94	**2601.94**	**2623.93**	**1301.48**	**867.99**
TeN	dF-GnM2F	1		2	3	3	4					2631.97	**2632.98**	**2654.96**	**1316.99**	**878.33**
TeN	mF-AG1F		1		1	2	4					2647.96	**2648.97**	**2670.95**	**1324.99**	**883.66**
TeN	mF-LnM2F	1		3	3	2	4					2647.96	**2648.97**	**2670.95**	**1324.99**	**883.66**
TeN	MOF		1	1	1	1	4					2663.96	**2664.97**	**2686.95**	**1332.99**	**888.99**
TeN	GnM4F		1	1	1	1	4					2663.96	**2664.97**	**2686.95**	**1332.99**	**888.99**
TeN	mGal-MO		1	2	1		4					2679.95	**2680.96**	**2702.94**	**1340.98**	**894.32**
TeN	LnM4		1	2	1	0	4					2679.95	**2680.96**	**2702.94**	**1340.98**	**894.32**
TeN	GnM5		1	2	1	0	4					2679.95	**2680.96**	**2702.94**	**1340.98**	**894.32**
TeN	mF-LdnF	1		2	4	2	4					2688.99	**2690.00**	**2711.98**	**1345.50**	**897.34**
TeN	AG12F, AG1BSF		1		2	1	4					2704.98	**2705.99**	**2727.97**	**1353.50**	**902.67**
TeN	LdnM3F		1		2	1	4					2704.98	**2705.99**	**2727.97**	**1353.50**	**902.67**
TeN	LnM2BSF	1		3	4	1	4					2704.98	**2705.99**	**2727.97**	**1353.50**	**902.67**
TeN	MOBS		1	1	2	0	4					2720.98	**2721.99**	**2743.97**	**1361.50**	**908.00**
TeN	BI-Ga		1	1	2	0	4					2720.98	**2721.99**	**2743.97**	**1361.50**	**908.00**
TeN	GnM4BS		1	1	2	0	4					2720.98	**2721.99**	**2743.97**	**1361.50**	**908.00**
TeN	LdnM4		1	1	2		4					2720.98	**2721.99**	**2743.97**	**1361.50**	**908.00**
TeN	dF-GnM2BSF			2	4	3	4					2738.98	**2739.99**	**2761.97**	**1370.50**	**914.00**
TeN	LdnM2BSF	1		2	5	1	4					2746.01	**2747.02**	**2769.00**	**1374.01**	**916.34**
TeN	GnLdnM2F	1		2	5	1	4					2746.01	**2747.02**	**2769.00**	**1374.01**	**916.34**
TeN	AG123, AG12BS		1		3		4					2762.01	**2763.01**	**2784.99**	**1382.01**	**921.68**
TeN	GnLdnM3, LdnM3BS		1		3		4					2762.01	**2763.01**	**2784.99**	**1382.01**	**921.68**
TeN	Ac-BI-Ga		1	1	2		4		1			2762.99	**2764.00**	**2785.98**	**1382.50**	**922.00**
TeN	dF-AG1F		1		1	3	4					2794.02	**2795.03**	**2817.01**	**1398.02**	**932.35**
TeN	dLdnM2	1		2	6		4					2803.03	**2804.04**	**2826.02**	**1402.52**	**935.35**
TeN	mF-MOF		1	1	1	2	4					2810.02	**2811.02**	**2833.00**	**1406.02**	**937.68**
TeN	mF-GnM4F		1	1	1	2	4					2810.02	**2811.02**	**2833.00**	**1406.02**	**937.68**
TeN	mGal-MOF		1	2	1	1	4					2826.01	**2827.02**	**2849.00**	**1414.01**	**943.01**
TeN	LnM4F, GnM5F		1	2	1	1	4					2826.01	**2827.02**	**2849.00**	**1414.01**	**943.01**
TeN	LnM5, GnM6		1	3	1	0	4					2842.01	**2843.01**	**2864.99**	**1422.01**	**948.34**
TeN	mF-AG1BSF		1		2	2	4					2851.04	**2852.05**	**2874.03**	**1426.53**	**951.35**
TeN	mF-AG12F		1		2	2	4					2851.04	**2852.05**	**2874.03**	**1426.53**	**951.35**
TeN	mF-LnM2BSF	1		3	4	2	4					2851.04	**2852.05**	**2874.03**	**1426.53**	**951.35**
TeN	mF-LdnM3F		1		2	2	4					2851.04	**2852.05**	**2874.03**	**1426.53**	**951.35**
TeN	MOBSF		1	1	2	1	4					2867.04	**2868.04**	**2890.03**	**1434.53**	**956.69**
TeN	BIF-Ga		1	1	2	1	4					2867.04	**2868.04**	**2890.03**	**1434.53**	**956.69**
TeN	GnM4BSF		1	1	2	1	4					2867.04	**2868.04**	**2890.03**	**1434.53**	**956.69**
TeN	LdnM4F		1	1	2	1	4					2867.04	**2868.04**	**2890.03**	**1434.53**	**956.69**
TeN	BI		1	2	2	0	4					2883.03	**2884.04**	**2906.02**	**1442.52**	**962.02**
TeN	mGal-MOBS		1	2	2		4					2883.03	**2884.04**	**2906.02**	**1442.52**	**962.02**
TeN	mGal-BI-G		1	2	2		4					2883.03	**2884.04**	**2906.02**	**1442.52**	**962.02**
TeN	LnM4BS		1	2	2	0	4					2883.03	**2884.04**	**2906.02**	**1442.52**	**962.02**
TeN	GnM5BS		1	2	2	0	4					2883.03	**2884.04**	**2906.02**	**1442.52**	**962.02**
TeN	GnLnM4		1	2	2		4					2883.03	**2884.04**	**2906.02**	**1442.52**	**962.02**
TeN	LdnM5		1	2	2		4					2883.03	**2884.04**	**2906.02**	**1442.52**	**962.02**
TeN	mF-LdnBSF	1		2	5	2	4					2892.07	**2893.08**	**2915.06**	**1447.04**	**965.03**
TeN	mF-GnLdnM2F	1		2	5	2	4					2892.07	**2893.08**	**2915.06**	**1447.04**	**965.03**
TeN	AG12BSF		1		3	1	4					2908.06	**2909.07**	**2931.05**	**1455.04**	**970.36**
TeN	AG123F		1		3	1	4					2908.06	**2909.07**	**2931.05**	**1455.04**	**970.36**
TeN	GnLdnM3F, LdnM3BSF		1		3	1	4					2908.06	**2909.07**	**2931.05**	**1455.04**	**970.36**
TeN	BIBS-G		1	1	3	0	4					2924.06	**2925.07**	**2947.05**	**1463.04**	**975.69**
TeN	TR-2G		1	1	3		4					2924.06	**2925.07**	**2947.05**	**1463.04**	**975.69**

TeN	BI(mLdn)		1	1	3		4					2924.06	**2925.07**	**2947.05**	**1463.04**	**975.69**
TeN	LdnM4BS		1	1	3		4					2924.06	**2925.07**	**2947.05**	**1463.04**	**975.69**
TeN	GnLdnM4		1	1	3		4					2924.06	**2925.07**	**2947.05**	**1463.04**	**975.69**
TeN	Ac-BI		1	2	2		4		1			2925.04	**2926.05**	**2948.03**	**1463.53**	**976.02**
TeN	dLdnM2F	1		2	6	1	4					2949.09	**2950.10**	**2972.08**	**1475.55**	**984.04**
TeN	AG123BS		1		4		4					2965.09	**2966.09**	**2988.07**	**1483.55**	**989.37**
TeN	GnLdnM3BS		1		4		4					2965.09	**2966.09**	**2988.07**	**1483.55**	**989.37**
TeN	BI(dLdn)		1		4		4					2965.09	**2966.09**	**2988.07**	**1483.55**	**989.37**
TeN	mGal-mF-MOF		1	2	1	2	4					2972.07	**2973.08**	**2995.06**	**1487.04**	**991.70**
TeN	mF-LnM4F		1	2	1	2	4					2972.07	**2973.08**	**2995.06**	**1487.04**	**991.70**
TeN	mF-GnM5F		1	2	1	2	4					2972.07	**2973.08**	**2995.06**	**1487.04**	**991.70**
TeN	LnM5F		1	3	1	1	4					2988.06	**2989.07**	**3011.05**	**1495.04**	**997.03**
TeN	dF-AG1BSF		1		2	3	4					2997.10	**2998.11**	**3020.09**	**1499.56**	**1000.04**
TeN	dF-AG12F		1		2	3	4					2997.10	**2998.11**	**3020.09**	**1499.56**	**1000.04**
TeN	mG-LnM5		1	4	1	0	4					3004.06	**3005.07**	**3027.05**	**1503.04**	**1002.36**
TeN	mF-MOBSF		1	1	2	2	4					3013.10	**3014.10**	**3036.08**	**1507.55**	**1005.37**
TeN	mF-BIF-G		1	1	2	2	4					3013.10	**3014.10**	**3036.08**	**1507.55**	**1005.37**
TeN	mF-GnM4BSF		1	1	2	2	4					3013.10	**3014.10**	**3036.08**	**1507.55**	**1005.37**
TeN	mF-LdnM4F		1	1	2	2	4					3013.10	**3014.10**	**3036.08**	**1507.55**	**1005.37**
TeN	BIF, mF-BI		1	2	2	1	4					3029.09	**3030.10**	**3052.08**	**1515.55**	**1010.70**
TeN	mGa-MOBSF		1	2	2	1	4					3029.09	**3030.10**	**3052.08**	**1515.55**	**1010.70**
TeN	mGa-BIF-G		1	2	2	1	4					3029.09	**3030.10**	**3052.08**	**1515.55**	**1010.70**
TeN	mGa-mF-MOBS		1	2	2	1	4					3029.09	**3030.10**	**3052.08**	**1515.55**	**1010.70**
TeN	LnM4BSF		1	2	2	1	4					3029.09	**3030.10**	**3052.08**	**1515.55**	**1010.70**
TeN	GnM5BSF		1	2	2	1	4					3029.09	**3030.10**	**3052.08**	**1515.55**	**1010.70**
TeN	GnLnM4F		1	2	2	1	4					3029.09	**3030.10**	**3052.08**	**1515.55**	**1010.70**
TeN	LdnM5F		1	2	2	1	4					3029.09	**3030.10**	**3052.08**	**1515.55**	**1010.70**
TeN	dF-GnLdnM2F	1		2	5	3	4					3038.13	**3039.13**	**3061.12**	**1520.07**	**1013.72**
TeN	mGa-BI		1	3	2		4					3045.08	**3046.09**	**3068.07**	**1523.55**	**1016.04**
TeN	LnM5BS		1	3	2		4					3045.08	**3046.09**	**3068.07**	**1523.55**	**1016.04**
TeN	GnLnM5		1	3	2		4					3045.08	**3046.09**	**3068.07**	**1523.55**	**1016.04**
TeN	dLnM4		1	3	2		4					3045.08	**3046.09**	**3068.07**	**1523.55**	**1016.04**
TeN	mF-AG12BSF		1		3	2	4					3054.12	**3055.13**	**3077.11**	**1528.07**	**1019.05**
TeN	mF-AG123F		1		3	2	4					3054.12	**3055.13**	**3077.11**	**1528.07**	**1019.05**
TeN	mF-LdnM3BSF		1		3	2	4					3054.12	**3055.13**	**3077.11**	**1528.07**	**1019.05**
TeN	mF-LdnAG1F		1		3	2	4					3054.12	**3055.13**	**3077.11**	**1528.07**	**1019.05**
TeN	BIBSF-Ga		1	1	3	1	4					3070.12	**3071.12**	**3093.11**	**1536.07**	**1024.38**
TeN	TRF-2Ga		1	1	3	1	4					3070.12	**3071.12**	**3093.11**	**1536.07**	**1024.38**
TeN	BI(mLdn)F		1	1	3	1	4					3070.12	**3071.12**	**3093.11**	**1536.07**	**1024.38**
TeN	LdnM4BSF		1	1	3	1	4					3070.12	**3071.12**	**3093.11**	**1536.07**	**1024.38**
TeN	GnLdnM4F		1	1	3	1	4					3070.12	**3071.12**	**3093.11**	**1536.07**	**1024.38**
TeN	BIBS		1	2	3	0	4					3086.11	**3087.12**	**3109.10**	**1544.06**	**1029.71**
TeN	TR-Ga		1	2	3		4					3086.11	**3087.12**	**3109.10**	**1544.06**	**1029.71**
TeN	mGal-BIBS-Ga		1	2	3		4					3086.11	**3087.12**	**3109.10**	**1544.06**	**1029.71**
TeN	GnLnM4BS		1	2	3		4					3086.11	**3087.12**	**3109.10**	**1544.06**	**1029.71**
TeN	LdnM5BS		1	2	3		4					3086.11	**3087.12**	**3109.10**	**1544.06**	**1029.71**
TeN	GnLdnM5		1	2	3		4					3086.11	**3087.12**	**3109.10**	**1544.06**	**1029.71**
TeN	mF-dLdnM2F	1		2	6	2	4					3095.15	**3096.16**	**3118.14**	**1548.58**	**1032.72**
TeN	AG123BSF		1		4	1	4					3111.14	**3112.15**	**3134.13**	**1556.58**	**1038.05**
TeN	LdnAG1BSF		1		4	1	4					3111.14	**3112.15**	**3134.13**	**1556.58**	**1038.05**
TeN	BI(dLdn)F		1		4	1	4					3111.14	**3112.15**	**3134.13**	**1556.58**	**1038.05**
TeN	TRBS-2Ga		1	1	4		4					3127.14	**3128.15**	**3150.13**	**1564.58**	**1043.39**
TeN	BI(mLdn)BS		1	1	4		4					3127.14	**3128.15**	**3150.13**	**1564.58**	**1043.39**
TeN	GnLdnM4BS		1	1	4		4					3127.14	**3128.15**	**3150.13**	**1564.58**	**1043.39**
TeN	mF-LnM5F		1	3	1	2	4					3134.12	**3135.13**	**3157.11**	**1568.07**	**1045.71**
TeN	trF-AG12F		1		2	4	4					3143.16	**3144.17**	**3166.15**	**1572.59**	**1048.73**
TeN	mG-LnM5F		1	4	1	1	4					3150.12	**3151.12**	**3173.11**	**1576.07**	**1051.05**
TeN	dF-BIF-Ga		1	1	2	3	4					3159.15	**3160.16**	**3182.14**	**1580.58**	**1054.06**
TeN	AG1234BS		1		5		4					3168.16	**3169.17**	**3191.15**	**1585.09**	**1057.06**
TeN	BI(dLdn)BS		1		5		4					3168.16	**3169.17**	**3191.15**	**1585.09**	**1057.06**
TeN	mF-BIF, diF-BI		1	2	2	2	4					3175.15	**3176.16**	**3198.14**	**1588.58**	**1059.39**
TeN	mGa-mF-MOBSF		1	2	2	2	4					3175.15	**3176.16**	**3198.14**	**1588.58**	**1059.39**
TeN	mGa-mF-BIF-Ga		1	2	2	2	4					3175.15	**3176.16**	**3198.14**	**1588.58**	**1059.39**
TeN	mF-LnM4BSF		1	2	2	2	4					3175.15	**3176.16**	**3198.14**	**1588.58**	**1059.39**
TeN	mF-GnM5BSF		1	2	2	2	4					3175.15	**3176.16**	**3198.14**	**1588.58**	**1059.39**
TeN	mF-GnLnM4F		1	2	2	2	4					3175.15	**3176.16**	**3198.14**	**1588.58**	**1059.39**
TeN	mF-LdnM5F		1	2	2	2	4					3175.15	**3176.16**	**3198.14**	**1588.58**	**1059.39**
TeN	trF-GnLdnM2F	1		2	5	4	4					3184.18	**3185.19**	**3207.17**	**1593.10**	**1062.40**
TeN	mGa-BIF		1	3	2	1	4					3191.14	**3192.15**	**3214.13**	**1596.58**	**1064.72**
TeN	mGa-mF-BI		1	3	2	1	4					3191.14	**3192.15**	**3214.13**	**1596.58**	**1064.72**
TeN	LnM5BSF		1	3	2	1	4					3191.14	**3192.15**	**3214.13**	**1596.58**	**1064.72**
TeN	GnLnM5F		1	3	2	1	4					3191.14	**3192.15**	**3214.13**	**1596.58**	**1064.72**
TeN	dLnM4F		1	3	2	1	4					3191.14	**3192.15**	**3214.13**	**1596.58**	**1064.72**

TeN	dF-AG12BSF		1		3	3	4					3200.18	**3201.19**	**3223.17**	**1601.10**	**1067.73**
TeN	dF-AG123F		1		3	3	4					3200.18	**3201.19**	**3223.17**	**1601.10**	**1067.73**
TeN	dF-GnLdnM3F		1		3	3	4					3200.18	**3201.19**	**3223.17**	**1601.10**	**1067.73**
TeN	dGa-BI		1	4	2		4					3207.14	**3208.14**	**3230.13**	**1604.58**	**1070.05**
TeN	mG-LnM5BS		1	4	2	0	4					3207.14	**3208.14**	**3230.13**	**1604.58**	**1070.05**
TeN	dLnM5		1	4	2		4					3207.14	**3208.14**	**3230.13**	**1604.58**	**1070.05**
TeN	mF-BIBSF-G		1	1	3	2	4					3216.17	**3217.18**	**3239.16**	**1609.09**	**1073.07**
TeN	mF-TRF-2G		1	1	3	2	4					3216.17	**3217.18**	**3239.16**	**1609.09**	**1073.07**
TeN	mF-BI(mLdn)F		1	1	3	2	4					3216.17	**3217.18**	**3239.16**	**1609.09**	**1073.07**
TeN	mF-LdnM4BSF		1	1	3	2	4					3216.17	**3217.18**	**3239.16**	**1609.09**	**1073.07**
TeN	mF-GnLdnM4F		1	1	3	2	4					3216.17	**3217.18**	**3239.16**	**1609.09**	**1073.07**
TeN	BIBSF		1	2	3	1	4					3232.17	**3233.18**	**3255.16**	**1617.09**	**1078.40**
TeN	TRF-Ga		1	2	3	1	4					3232.17	**3233.18**	**3255.16**	**1617.09**	**1078.40**
TeN	mGa-BIBSF-Ga		1	2	3	1	4					3232.17	**3233.18**	**3255.16**	**1617.09**	**1078.40**
TeN	mGa-mF-BIBS-Ga		1	2	3	1	4					3232.17	**3233.18**	**3255.16**	**1617.09**	**1078.40**
TeN	mF-BIBS		1	2	3	1	4					3232.17	**3233.18**	**3255.16**	**1617.09**	**1078.40**
TeN	GnLnM4BSF		1	2	3	1	4					3232.17	**3233.18**	**3255.16**	**1617.09**	**1078.40**
TeN	LdnM5BSF		1	2	3	1	4					3232.17	**3233.18**	**3255.16**	**1617.09**	**1078.40**
TeN	GnLdnM5F		1	2	3	1	4					3232.17	**3233.18**	**3255.16**	**1617.09**	**1078.40**
TeN	dF-dLdnM2F	1		2	6	3	4					3241.21	**3242.21**	**3264.20**	**1621.61**	**1081.41**
TeN	TR		1	3	3	0	4					3248.16	**3249.17**	**3271.15**	**1625.09**	**1083.73**
TeN	mGa-BIBS		1	3	3		4					3248.16	**3249.17**	**3271.15**	**1625.09**	**1083.73**
TeN	GnLnM5BS		1	3	3		4					3248.16	**3249.17**	**3271.15**	**1625.09**	**1083.73**
TeN	dLnM4BS		1	3	3		4					3248.16	**3249.17**	**3271.15**	**1625.09**	**1083.73**
TeN	mF-AG123BSF		1		4	2	4					3257.20	**3258.21**	**3280.19**	**1629.61**	**1086.74**
TeN	mF-LdnAG1BSF		1		4	2	4					3257.20	**3258.21**	**3280.19**	**1629.61**	**1086.74**
TeN	mF-dLdnM3F		1		4	2	4					3257.20	**3258.21**	**3280.19**	**1629.61**	**1086.74**
TeN	TRBSF-2G		1	1	4	1	4					3273.20	**3274.20**	**3296.19**	**1637.61**	**1092.07**
TeN	BI(mLdn)BSF		1	1	4	1	4					3273.20	**3274.20**	**3296.19**	**1637.61**	**1092.07**
TeN	GnLdnM4BSF		1	1	4	1	4					3273.20	**3274.20**	**3296.19**	**1637.61**	**1092.07**
TeN	TRBS-G		1	2	4	0	4					3289.19	**3290.20**	**3312.18**	**1645.60**	**1097.40**
TeN	TR(mLdn)		1	2	4		4					3289.19	**3290.20**	**3312.18**	**1645.60**	**1097.40**
TeN	GnLdnM5BS		1	2	4		4					3289.19	**3290.20**	**3312.18**	**1645.60**	**1097.40**
TeN	AG1234BSF		1		5	1	4					3314.22	**3315.23**	**3337.21**	**1658.12**	**1105.75**
TeN	BI(dLdn)BSF		1		5	1	4					3314.22	**3315.23**	**3337.21**	**1658.12**	**1105.75**
TeN	dF-BIF		1	2	2	3	4					3321.21	**3322.21**	**3344.19**	**1661.61**	**1108.08**
TeN	TR(dLdn)		1	1	5		4					3330.22	**3331.22**	**3353.21**	**1666.12**	**1111.08**
TeN	mGa-dF-BI		1	3	2	2	4					3337.20	**3338.21**	**3360.19**	**1669.61**	**1113.41**
TeN	mGa-mF-BIF		1	3	2	2	4					3337.20	**3338.21**	**3360.19**	**1669.61**	**1113.41**
TeN	mF-LnM5BSF		1	3	2	2	4					3337.20	**3338.21**	**3360.19**	**1669.61**	**1113.41**
TeN	mF-GnLnM5F		1	3	2	2	4					3337.20	**3338.21**	**3360.19**	**1669.61**	**1113.41**
TeN	mF-dLnM4F		1	3	2	2	4					3337.20	**3338.21**	**3360.19**	**1669.61**	**1113.41**
TeN	trF-AG12BSF		1		3	4	4					3346.24	**3347.24**	**3369.23**	**1674.13**	**1116.42**
TeN	trF-AG123F		1		3	4	4					3346.24	**3347.24**	**3369.23**	**1674.13**	**1116.42**
TeN	trF-LdnAG1F		1		3	4	4					3346.24	**3347.24**	**3369.23**	**1674.13**	**1116.42**
TeN	dGa-BIF, dGa-mF-BI		1	4	2	1	4					3353.20	**3354.20**	**3376.18**	**1677.61**	**1118.74**
TeN	mGa-LnM5BSF		1	4	2	1	4					3353.20	**3354.20**	**3376.18**	**1677.61**	**1118.74**
TeN	dLnM5F		1	4	2	1	4					3353.20	**3354.20**	**3376.18**	**1677.61**	**1118.74**
TeN	dF-BIBSF-G		1	1	3	3	4					3362.23	**3363.24**	**3385.22**	**1682.12**	**1121.75**
TeN	dF-TRF-2G		1	1	3	3	4					3362.23	**3363.24**	**3385.22**	**1682.12**	**1121.75**
TeN	dF-BI(mLdn)F		1	1	3	3	4					3362.23	**3363.24**	**3385.22**	**1682.12**	**1121.75**
TeN	TR(trLdn)		1		6		4					3371.24	**3372.25**	**3394.23**	**1686.63**	**1124.76**
TeN	mF-BIBSF		1	2	3	2	4					3378.23	**3379.23**	**3401.22**	**1690.12**	**1127.08**
TeN	mF-TRF-G		1	2	3	2	4					3378.23	**3379.23**	**3401.22**	**1690.12**	**1127.08**
TeN	mGa-mF-BIBSF-G		1	2	3	2	4					3378.23	**3379.23**	**3401.22**	**1690.12**	**1127.08**
TeN	dF-BIBS, mF-BIBSF		1	2	3	2	4					3378.23	**3379.23**	**3401.22**	**1690.12**	**1127.08**
TeN	mF-GnLnM4BSF		1	2	3	2	4					3378.23	**3379.23**	**3401.22**	**1690.12**	**1127.08**
TeN	mF-LdnM5BSF		1	2	3	2	4					3378.23	**3379.23**	**3401.22**	**1690.12**	**1127.08**
TeN	mF-GnLdnM5F		1	2	3	2	4					3378.23	**3379.23**	**3401.22**	**1690.12**	**1127.08**
TeN	trF-dLdnM2F	1		2	6	4	4					3387.26	**3388.27**	**3410.25**	**1694.64**	**1130.10**
TeN	TRF, mF-TR		1	3	3	1	4					3394.22	**3395.23**	**3417.21**	**1698.12**	**1132.41**
TeN	mGa-BIBSF		1	3	3	1	4					3394.22	**3395.23**	**3417.21**	**1698.12**	**1132.41**
TeN	mGal-mF-BIBS		1	3	3	1	4					3394.22	**3395.23**	**3417.21**	**1698.12**	**1132.41**
TeN	GnLnM5BSF		1	3	3	1	4					3394.22	**3395.23**	**3417.21**	**1698.12**	**1132.41**
TeN	diLnM4BSF		1	3	3	1	4					3394.22	**3395.23**	**3417.21**	**1698.12**	**1132.41**
TeN	dF-AG123BSF		1		4	3	4					3403.26	**3404.27**	**3426.25**	**1702.64**	**1135.43**
TeN	dF-LdnAG1BSF		1		4	3	4					3403.26	**3404.27**	**3426.25**	**1702.64**	**1135.43**
TeN	dF-dLdnM3F		1		4	3	4					3403.26	**3404.27**	**3426.25**	**1702.64**	**1135.43**
TeN	mGa-TR, dGa-BIBS		1	4	3		4					3410.22	**3411.22**	**3433.21**	**1706.12**	**1137.75**
TeN	dLnM5BS		1	4	3		4					3410.22	**3411.22**	**3433.21**	**1706.12**	**1137.75**
TeN	mF-TRBSF-2G		1	1	4	2	4					3419.25	**3420.26**	**3442.24**	**1710.63**	**1140.76**
TeN	mF-LdnMOBSF		1	1	4	2	4					3419.25	**3420.26**	**3442.24**	**1710.63**	**1140.76**
TeN	mF-GnLdnM4BSF		1	1	4	2	4					3419.25	**3420.26**	**3442.24**	**1710.63**	**1140.76**

TeN	TRBSF-G		1	2	4	1	4					3435.25	**3436.26**	**3458.24**	**1718.63**	**1146.09**
TeN	TR(mLdn)F		1	2	4	1	4					3435.25	**3436.26**	**3458.24**	**1718.63**	**1146.09**
TeN	GnLdnM5BSF		1	2	4	1	4					3435.25	**3436.26**	**3458.24**	**1718.63**	**1146.09**
TeN	TRBS		1	3	4	0	4					3451.24	**3452.25**	**3474.23**	**1726.63**	**1151.42**
TeN	mF-AG1234BSF		1		5	2	4					3460.28	**3461.29**	**3483.27**	**1731.15**	**1154.43**
TeN	mF-dLdnM3BSF		1		5	2	4					3460.28	**3461.29**	**3483.27**	**1731.15**	**1154.43**
TeN	TR(dLdn)F		1	1	5	1	4					3476.28	**3477.28**	**3499.26**	**1739.14**	**1159.77**
TeN	mGa-dF-BIF		1	3	2	3	4					3483.26	**3484.27**	**3506.25**	**1742.64**	**1162.09**
TeN	dGa-dF-BI		1	4	2	2	4					3499.25	**3500.26**	**3522.24**	**1750.63**	**1167.43**
TeN	dGa-mF-BIF		1	4	2	2	4					3499.25	**3500.26**	**3522.24**	**1750.63**	**1167.43**
TeN	mF-diLnM5F		1	4	2	2	4					3499.25	**3500.26**	**3522.24**	**1750.63**	**1167.43**
TeN	trF-TRF-2G		1	1	3	4	4					3508.29	**3509.30**	**3531.28**	**1755.15**	**1170.44**
TeN	trF-LdnMOF		1	1	3	4	4					3508.29	**3509.30**	**3531.28**	**1755.15**	**1170.44**
TeN	TR(trLdn)F		1		6	1	4					3517.30	**3518.31**	**3540.29**	**1759.66**	**1173.44**
TeN	dF-BIBSF		1	2	3	3	4					3524.29	**3525.29**	**3547.27**	**1763.15**	**1175.77**
TeN	dF-TRF-Ga		1	2	3	3	4					3524.29	**3525.29**	**3547.27**	**1763.15**	**1175.77**
TeN	dF-TR		1	3	3	2	4					3540.28	**3541.29**	**3563.27**	**1771.15**	**1181.10**
TeN	mF-TRF		1	3	3	2	4					3540.28	**3541.29**	**3563.27**	**1771.15**	**1181.10**
TeN	mGa-dF-BIBS		1	3	3	2	4					3540.28	**3541.29**	**3563.27**	**1771.15**	**1181.10**
TeN	mGa-mF-BIBSF		1	3	3	2	4					3540.28	**3541.29**	**3563.27**	**1771.15**	**1181.10**
TeN	mF-GnLnM5BSF		1	3	3	2	4					3540.28	**3541.29**	**3563.27**	**1771.15**	**1181.10**
TeN	mF-dLnM4BSF		1	3	3	2	4					3540.28	**3541.29**	**3563.27**	**1771.15**	**1181.10**
TeN	Ac-dG-dF-BI		1	4	2	2	4		1			3541.26	**3542.27**	**3564.25**	**1771.64**	**1181.43**
TeN	trF-AG123BSF		1		4	4	4					3549.32	**3550.32**	**3572.31**	**1775.67**	**1184.11**
TeN	trF-GnLdnM3BSF		1		4	4	4					3549.32	**3550.32**	**3572.31**	**1775.67**	**1184.11**
TeN	trF-BI(dLdn)F		1		4	4	4					3549.32	**3550.32**	**3572.31**	**1775.67**	**1184.11**
TeN	mGa-TRF		1	4	3	1	4					3556.27	**3557.28**	**3579.26**	**1779.14**	**1186.43**
TeN	dGa-BIBSF		1	4	3	1	4					3556.27	**3557.28**	**3579.26**	**1779.14**	**1186.43**
TeN	mGa-mF-TR		1	4	3	1	4					3556.27	**3557.28**	**3579.26**	**1779.14**	**1186.43**
TeN	dGa-mF-BIBS		1	4	3	1	4					3556.27	**3557.28**	**3579.26**	**1779.14**	**1186.43**
TeN	dLnM5BSF		1	4	3	1	4					3556.27	**3557.28**	**3579.26**	**1779.14**	**1186.43**
TeN	dF-TRBSF-2G		1	1	4	3	4					3565.31	**3566.32**	**3588.30**	**1783.66**	**1189.44**
TeN	dF-BI(mLdn)BSF		1	1	4	3	4					3565.31	**3566.32**	**3588.30**	**1783.66**	**1189.44**
TeN	dGa-TR		1	5	3		4					3572.27	**3573.28**	**3595.26**	**1787.14**	**1191.76**
TeN	TR(trLdn)BS		1		7		4					3574.32	**3575.33**	**3597.31**	**1788.17**	**1192.45**
TeN	mF-TRBSF-Ga		1	2	4	2	4					3581.31	**3582.31**	**3604.30**	**1791.66**	**1194.78**
TeN	mF-LdnBIF		1	2	4	2	4					3581.31	**3582.31**	**3604.30**	**1791.66**	**1194.78**
TeN	mF-GnLdnM5BSF		1	2	4	2	4					3581.31	**3582.31**	**3604.30**	**1791.66**	**1194.78**
TeN	TRBSF		1	3	4	1	4					3597.30	**3598.31**	**3620.29**	**1799.66**	**1200.11**
TeN	mF-TRBS		1	3	4	1	4					3597.30	**3598.31**	**3620.29**	**1799.66**	**1200.11**
TeN	dF-AG1234BSF		1		5	3	4					3606.34	**3607.35**	**3629.33**	**1804.18**	**1203.12**
TeN	dF-dLdnM3BSF		1		5	3	4					3606.34	**3607.35**	**3629.33**	**1804.18**	**1203.12**
TeN	TE		1	4	4	0	4					3613.30	**3614.30**	**3636.29**	**1807.66**	**1205.44**
TeN	mGal-TRBS		1	4	4		4					3613.30	**3614.30**	**3636.29**	**1807.66**	**1205.44**
TeN	dLn-LdnM5A		1	4	4		4					3613.30	**3614.30**	**3636.29**	**1807.66**	**1205.44**
TeN	mF-TR(dLdn)F		1	1	5	2	4					3622.33	**3623.34**	**3645.32**	**1812.17**	**1208.45**
TeN	diGal-diF-BIF		1	4	2	3	4					3645.31	**3646.32**	**3668.30**	**1823.66**	**1216.11**
TeN	mF-trLdnM3F		1		6	2	4					3663.36	**3664.37**	**3686.35**	**1832.69**	**1222.13**
TeN	trF-TRF-Ga		1	2	3	4	4					3670.34	**3671.35**	**3693.33**	**1836.18**	**1224.45**
TeN	dF-TRF		1	3	3	3	4					3686.34	**3687.35**	**3709.33**	**1844.18**	**1229.79**
TeN	trF-TR		1	3	3	3	4					3686.34	**3687.35**	**3709.33**	**1844.18**	**1229.79**
TeN	mGa-dF-BIBSF		1	3	3	3	4					3686.34	**3687.35**	**3709.33**	**1844.18**	**1229.79**
TeN	mGa-dF-TR		1	4	3	2	4					3702.33	**3703.34**	**3725.32**	**1852.17**	**1235.12**
TeN	mGa-mF-TRF		1	4	3	2	4					3702.33	**3703.34**	**3725.32**	**1852.17**	**1235.12**
TeN	dGa-dF-BIBS		1	4	3	2	4					3702.33	**3703.34**	**3725.32**	**1852.17**	**1235.12**
TeN	dGa-mF-BIBSF		1	4	3	2	4					3702.33	**3703.34**	**3725.32**	**1852.17**	**1235.12**
TeN	mF-dLnM5BSF		1	4	3	2	4					3702.33	**3703.34**	**3725.32**	**1852.17**	**1235.12**
TeN	trF-TRBSF-2G		1	1	4	4	4					3711.37	**3712.38**	**3734.36**	**1856.69**	**1238.13**
TeN	trF-BI(mLdn)BSF		1	1	4	4	4					3711.37	**3712.38**	**3734.36**	**1856.69**	**1238.13**
TeN	dGa-TRF		1	5	3	1	4					3718.33	**3719.34**	**3741.32**	**1860.17**	**1240.45**
TeN	dGa-mF-TR		1	5	3	1	4					3718.33	**3719.34**	**3741.32**	**1860.17**	**1240.45**
TeN	trLdnM3BSF		1		7	1	4					3720.38	**3721.39**	**3743.37**	**1861.20**	**1241.13**
TeN	dF-TRBSF-G		1	2	4	3	4					3727.36	**3728.37**	**3750.35**	**1864.69**	**1243.46**
TeN	dF-LdnBIF		1	2	4	3	4					3727.36	**3728.37**	**3750.35**	**1864.69**	**1243.46**
TeN	trGa-TR		1	6	3		4					3734.32	**3735.33**	**3757.31**	**1868.17**	**1245.78**
TeN	mF-TRBSF, diF-TRBS		1	3	4	2	4					3743.36	**3744.37**	**3766.35**	**1872.69**	**1248.79**
TeN	trF-AG1234BSF		1		5	4	4					3752.40	**3753.40**	**3775.39**	**1877.21**	**1251.81**
TeN	trF-BI(dLdn)BSF		1		5	4	4					3752.40	**3753.40**	**3775.39**	**1877.21**	**1251.81**
TeN	TEF		1	4	4	1	4					3759.35	**3760.36**	**3782.34**	**1880.68**	**1254.13**
TeN	mGa-TRBSF		1	4	4	1	4					3759.35	**3760.36**	**3782.34**	**1880.68**	**1254.13**
TeN	mGa-mF-TRBS		1	4	4	1	4					3759.35	**3760.36**	**3782.34**	**1880.68**	**1254.13**
TeN	mF-dLn-LdnM5A		1	4	4	1	4					3759.35	**3760.36**	**3782.34**	**1880.68**	**1254.13**
TeN	dF-TR(dLdn)F		1	1	5	3	4					3768.39	**3769.40**	**3791.38**	**1885.20**	**1257.14**

TeN	dGa-TRBS		1	5	4		4					3775.35	**3776.36**	**3798.34**	**1888.68**	**1259.46**
TeN	TE(teLdn)		1		8		4					3777.40	**3778.41**	**3800.39**	**1889.71**	**1260.14**
TeN	dF-TR(trLdn)F		1		6	3	4					3809.42	**3810.42**	**3832.41**	**1905.72**	**1270.81**
TeN	TEBS		1	4	5	0	4					3816.38	**3817.38**	**3839.37**	**1909.20**	**1273.13**
TeN	trF-TRF		1	3	3	4	4					3832.40	**3833.40**	**3855.39**	**1917.21**	**1278.47**
TeN	mGa-trF-TR		1	4	3	3	4					3848.39	**3849.40**	**3871.38**	**1925.20**	**1283.80**
TeN	mGa-dF-TRF		1	4	3	3	4					3848.39	**3849.40**	**3871.38**	**1925.20**	**1283.80**
TeN	dGa-diF-BIBSF		1	4	3	3	4					3848.39	**3849.40**	**3871.38**	**1925.20**	**1283.80**
TeN	dGa-diF-TR		1	5	3	2	4					3864.39	**3865.39**	**3887.37**	**1933.20**	**1289.14**
TeN	dGa-mF-TRF		1	5	3	2	4					3864.39	**3865.39**	**3887.37**	**1933.20**	**1289.14**
TeN	mF-TR(trLdn)BSF		1		7	2	4					3866.44	**3867.45**	**3889.43**	**1934.23**	**1289.82**
TeN	trF-TRBSF-G		1	2	4	4	4					3873.42	**3874.43**	**3896.41**	**1937.72**	**1292.15**
TeN	trF-TR(mLdn)F		1	2	4	4	4					3873.42	**3874.43**	**3896.41**	**1937.72**	**1292.15**
TeN	trGa-TRF		1	6	3	1	4					3880.38	**3881.39**	**3903.37**	**1941.20**	**1294.47**
TeN	trGa-mF-TR		1	6	3	1	4					3880.38	**3881.39**	**3903.37**	**1941.20**	**1294.47**
TeN	dF-TRBSF		1	3	4	3	4					3889.42	**3890.42**	**3912.41**	**1945.72**	**1297.48**
TeN	trF-TRBS		1	3	4	3	4					3889.42	**3890.42**	**3912.41**	**1945.72**	**1297.48**
TeN	dF-TRBSF		1	3	4	3	4					3889.42	**3890.42**	**3912.41**	**1945.72**	**1297.48**
TeN	teF-AG1234BSF		1		5	5	4					3898.45	**3899.46**	**3921.44**	**1950.23**	**1300.49**
TeN	mF-TEF		1	4	4	2	4					3905.41	**3906.42**	**3928.40**	**1953.71**	**1302.81**
TeN	mGa-dF-TRBS		1	4	4	2	4					3905.41	**3906.42**	**3928.40**	**1953.71**	**1302.81**
TeN	mGa-mF-TRBSF		1	4	4	2	4					3905.41	**3906.42**	**3928.40**	**1953.71**	**1302.81**
TeN	trF-TR(dLdn)F		1	1	5	4	4					3914.45	**3915.46**	**3937.44**	**1958.23**	**1305.82**
TeN	dGa-TRBSF		1	5	4	1	4					3921.41	**3922.41**	**3944.40**	**1961.71**	**1308.14**
TeN	dGa-mF-TRBS		1	5	4	1	4					3921.41	**3922.41**	**3944.40**	**1961.71**	**1308.14**
TeN	(teLdn)F		1		8	1	4					3923.46	**3924.47**	**3946.45**	**1962.74**	**1308.83**
TeN	trGa-TRBS		1	6	4		4					3937.40	**3938.41**	**3960.39**	**1969.71**	**1313.47**
TeN	trF-trLdnM3F		1		6	4	4					3955.48	**3956.48**	**3978.46**	**1978.75**	**1319.50**
TeN	TEBSF		1	4	5	1	4					3962.43	**3963.44**	**3985.42**	**1982.22**	**1321.82**
TeN	TR(teLdn)BS		1		9		4					3980.48	**3981.49**	**4003.47**	**1991.25**	**1327.83**
TeN	mGa-trF-TRF		1	4	3	4	4					3994.45	**3995.46**	**4017.44**	**1998.23**	**1332.49**
TeN	dGa-trF-TR		1	5	3	3	4					4010.44	**4011.45**	**4033.43**	**2006.23**	**1337.82**
TeN	dGa-dF-TRF		1	5	3	3	4					4010.44	**4011.45**	**4033.43**	**2006.23**	**1337.82**
TeN	dF-TR(trLdn)BSF		1		7	3	4					4012.50	**4013.50**	**4035.49**	**2007.26**	**1338.51**
TeN	trGa-dF-TR		1	6	3	2	4					4026.44	**4027.45**	**4049.43**	**2014.23**	**1343.15**
TeN	trGa-mF-TRF		1	6	3	2	4					4026.44	**4027.45**	**4049.43**	**2014.23**	**1343.15**
TeN	trF-TRBSF		1	3	4	4	4					4035.48	**4036.48**	**4058.46**	**2018.74**	**1346.17**
TeN	dF-TEF		1	4	4	3	4					4051.47	**4052.48**	**4074.46**	**2026.74**	**1351.50**
TeN	mGa-trF-TRBS		1	4	4	3	4					4051.47	**4052.48**	**4074.46**	**2026.74**	**1351.50**
TeN	mGa-dF-TRBSF		1	4	4	3	4					4051.47	**4052.48**	**4074.46**	**2026.74**	**1351.50**
TeN	dGa-dF-TRBS		1	5	4	2	4					4067.47	**4068.47**	**4090.45**	**2034.74**	**1356.83**
TeN	dGa-mF-TRBSF		1	5	4	2	4					4067.47	**4068.47**	**4090.45**	**2034.74**	**1356.83**
TeN	mF-teLdnM3F		1		8	2	4					4069.52	**4070.53**	**4092.51**	**2035.77**	**1357.51**
TeN	trGa-TRBSF		1	6	4	1	4					4083.46	**4084.47**	**4106.45**	**2042.74**	**1362.16**
TeN	trGa-mF-TRBS		1	6	4	1	4					4083.46	**4084.47**	**4106.45**	**2042.74**	**1362.16**
TeN	mF-TEBSF		1	4	5	2	4					4108.49	**4109.50**	**4131.48**	**2055.25**	**1370.50**
TeN	TE(teLdn)BSF		1		9	1	4					4126.54	**4127.55**	**4149.53**	**2064.28**	**1376.52**
TeN	dGa-trF-TRF		1	5	3	4	4					4156.50	**4157.51**	**4179.49**	**2079.26**	**1386.51**
TeN	trF-TR(trLdn)BSF		1		7	4	4					4158.55	**4159.56**	**4181.54**	**2080.28**	**1387.19**
TeN	trGa-trF-TR		1	6	3	3	4					4172.50	**4173.50**	**4195.49**	**2087.26**	**1391.84**
TeN	trGa-dF-TRF		1	6	3	3	4					4172.50	**4173.50**	**4195.49**	**2087.26**	**1391.84**
TeN	mGa-trF-TRBSF		1	4	4	4	4					4197.53	**4198.54**	**4220.52**	**2099.77**	**1400.18**
TeN	dGa-trF-TRBS		1	5	4	3	4					4213.52	**4214.53**	**4236.51**	**2107.77**	**1405.51**
TeN	dGa-dF-TRBSF		1	5	4	3	4					4213.52	**4214.53**	**4236.51**	**2107.77**	**1405.51**
TeN	dF-TE(teLdn)F		1		8	3	4					4215.58	**4216.58**	**4238.57**	**2108.80**	**1406.20**
TeN	trGa-dF-TRBS		1	6	4	2	4					4229.52	**4230.53**	**4252.51**	**2115.77**	**1410.85**
TeN	trGa-mF-TRBSF		1	6	4	2	4					4229.52	**4230.53**	**4252.51**	**2115.77**	**1410.85**
TeN	dF-TEBSF		1	4	5	3	4					4254.55	**4255.56**	**4277.54**	**2128.28**	**1419.19**
TeN	mF-TE(teLdn)BSF		1		9	2	4					4272.60	**4273.61**	**4295.59**	**2137.31**	**1425.21**
TeN	dF-dLn-dLdnM5A		1	4	6	2	4					4311.57	**4312.58**	**4334.56**	**2156.79**	**1438.20**
TeN	trGa-trF-TRF		1	6	3	4	4					4318.55	**4319.56**	**4341.54**	**2160.28**	**1440.53**
TeN	dGa-trF-TRBSF		1	5	4	4	4					4359.58	**4360.59**	**4382.57**	**2180.80**	**1454.20**
TeN	trF-(teLdn)F		1		8	4	4					4361.63	**4362.64**	**4384.62**	**2181.82**	**1454.89**
TeN	trGa-trF-TRBS		1	6	4	3	4					4375.58	**4376.58**	**4398.57**	**2188.80**	**1459.53**
TeN	trGa-dF-TRBSF		1	6	4	3	4					4375.58	**4376.58**	**4398.57**	**2188.80**	**1459.53**
TeN	dF-(teLdn)BSF		1		9	3	4					4418.66	**4419.66**	**4441.64**	**2210.34**	**1473.89**
TeN	triGal-triF-TRBSF		1	6	4	4	4					4521.63	**4522.64**	**4544.62**	**2261.82**	**1508.22**
TeN	trF-teLdnM3BSF		1		9	4	4					4564.71	**4565.72**	**4587.70**	**2283.36**	**1522.58**
TeN	teGa-teF-TE		1	8	4	4	4					4845.74	**4846.75**	**4868.73**	**2423.88**	**1616.25**

glycan fragments (B-type)		non-reducing end					modification					*m/z*			
		Hex	HexNAc	dHex	Pen	NANA	Me	Ac	HPO_3	SO_3	MS number	+H	+Na	+2H	3H+
	Xyl				1						132.04	**133.05**	**155.03**	**67.03**	**45.02**
	Fuc			1							146.06	**147.07**	**169.05**	**74.04**	**49.69**
	Me-Pen				1		1				146.06	**147.07**	**169.05**	**74.04**	**49.69**
	Me-Fuc			1			1				160.07	**161.08**	**183.06**	**81.04**	**54.37**
	Hex	1									162.05	**163.06**	**185.04**	**82.03**	**55.02**
	Me-Hex	1					1				176.07	**177.08**	**199.06**	**89.04**	**59.70**
	Me2-Hex	1					2				190.08	**191.09**	**213.07**	**96.05**	**64.37**
	HexNAc		1								203.08	**204.09**	**226.07**	**102.55**	**68.70**
	Ac-Hex	1						1			204.06	**205.07**	**227.05**	**103.04**	**69.03**
	S-Hex	1								1	242.01	**243.02**	**265.00**	**122.01**	**81.68**
	P-Hex	1							1		242.02	**243.03**	**265.01**	**122.02**	**81.68**
	Ac-HexNAc		1					1			245.09	**246.10**	**268.08**	**123.55**	**82.70**
	S-HexNAc		1							1	283.04	**284.04**	**306.03**	**142.53**	**95.35**
	P-HexNAc		1						1		283.05	**284.05**	**306.03**	**142.53**	**95.36**
	Neu5Ac					1					291.10	**292.10**	**314.08**	**146.55**	**98.04**
	Fuc2			2							292.12	**293.12**	**315.11**	**147.07**	**98.38**
	Pen1Hex1	1			1						294.10	**295.10**	**317.08**	**148.05**	**99.04**
	Me-NeuAc					1	1				305.11	**306.12**	**328.10**	**153.56**	**102.71**
	Hex1Fuc1	1		1							308.11	**309.12**	**331.10**	**155.06**	**103.71**
	Hex2	2									324.11	**325.11**	**347.09**	**163.06**	**109.04**
	Pen1HexNAc1		1		1						335.12	**336.13**	**358.11**	**168.57**	**112.71**
	Me-Hex2	2					1				338.12	**339.13**	**361.11**	**170.07**	**113.71**
	HexNAc1Fuc1		1	1							349.14	**350.14**	**372.13**	**175.58**	**117.39**
	Hex1HexNAc1	1	1								365.13	**366.14**	**388.12**	**183.57**	**122.72**
	Ac-Hex2	2						1			366.12	**367.12**	**389.11**	**184.07**	**123.05**
	Me-Hex1HexNAc1	1	1				1				379.15	**380.16**	**402.14**	**190.58**	**127.39**
	S-Hex2	2								1	404.06	**405.07**	**427.05**	**203.04**	**135.69**
	P-Hex2	2							1		404.07	**405.08**	**427.06**	**203.04**	**135.70**
	HexNAc2		2								406.16	**407.17**	**429.15**	**204.09**	**136.39**
	Ac-Hex1HexNAc1	1	1					1			407.14	**408.15**	**430.13**	**204.58**	**136.72**
	S-Hex1HexNAc	1	1							1	445.09	**446.10**	**468.08**	**223.55**	**149.37**
	P-Hex1HexNAc	1	1						1		445.10	**446.11**	**468.09**	**223.56**	**149.37**
	Ac-HexNAc2		2					1			448.17	**449.18**	**471.16**	**225.09**	**150.40**
	Hex1NeuAc1	1				1					453.15	**454.16**	**476.14**	**227.58**	**152.06**
	Hex1Fuc2	1		2							454.17	**455.18**	**477.16**	**228.09**	**152.40**
	Hex2Fuc1	2		1							470.16	**471.17**	**493.15**	**236.09**	**157.73**
	S-HexNAc2		2							1	486.12	**487.12**	**509.10**	**244.07**	**163.05**
	P-HexNAc2		2						1		486.13	**487.13**	**509.11**	**244.07**	**163.05**
	Hex3	3									486.16	**487.17**	**509.15**	**244.09**	**163.06**
	HexNAc1NeuAc1		1			1					494.17	**495.18**	**517.16**	**248.09**	**165.73**
	HexNAc1Fuc2		1	2							495.20	**496.20**	**518.18**	**248.60**	**166.07**
	Hex1HexNAc1Fuc1	1	1	1							511.19	**512.20**	**534.18**	**256.60**	**171.40**
	Hex2HexNAc1	2	1								527.19	**528.19**	**550.17**	**264.60**	**176.74**
	Ac-Hex3	3						1			528.17	**529.18**	**551.16**	**265.09**	**177.06**
	HexNAc2Fuc1		2	1							552.22	**553.22**	**575.21**	**277.12**	**185.08**
	S-Hex3	3								1	566.12	**567.12**	**589.10**	**284.06**	**189.71**
	P-Hex3	3							1		566.12	**567.13**	**589.11**	**284.07**	**189.72**
	Hex1HexNAc2	1	2								568.21	**569.22**	**591.20**	**285.11**	**190.41**
	Ac-Hex2HexNAc1	2	1					1			569.20	**570.20**	**592.18**	**285.61**	**190.74**
	NeuAc2					2					582.19	**583.20**	**605.18**	**292.10**	**195.07**
	Hex1Fuc1NeuAc1	1		1		1					599.21	**600.21**	**622.20**	**300.61**	**200.74**
	S-Hex2HexNAc1	2	1							1	607.14	**608.15**	**630.13**	**304.58**	**203.39**
	P-Hex2HexNAc1	2	1						1		607.15	**608.16**	**630.14**	**304.58**	**203.39**
	HexNAc3		3								609.24	**610.25**	**632.23**	**305.63**	**204.09**
	Ac-Hex1HexNAc2	1	2					1			610.22	**611.23**	**633.21**	**306.12**	**204.41**
	Hex2NeuAc1	2				1					615.20	**616.21**	**638.19**	**308.61**	**206.07**
	Hex2Fuc2	2		2							616.22	**617.23**	**639.21**	**309.12**	**206.41**
	Me-Hex2NA1	2				1	1				629.22	**630.22**	**652.21**	**315.62**	**210.75**
	HexNAc1Fuc1NeuAc1		1	1		1					640.23	**641.24**	**663.22**	**321.12**	**214.42**
	S-Hex1HexNAc2	1	2							1	648.17	**649.18**	**671.16**	**325.09**	**217.06**
	P-Hex1HexNAc2	1	2						1		648.18	**649.19**	**671.17**	**325.10**	**217.07**
	Hex4	4									648.21	**649.22**	**671.20**	**325.11**	**217.08**
	Hex1HexNAc1NeuAc1	1	1			1					656.23	**657.23**	**679.22**	**329.12**	**219.75**
	Hex1HexNAc1Fuc2	1	1	2							657.25	**658.26**	**680.24**	**329.63**	**220.09**
	Me-Hex1HexNAc1NA1	1	1			1	1				670.24	**671.25**	**693.23**	**336.13**	**224.42**
	Hex2HexNAc1Fuc1	2	1	1							673.24	**674.25**	**696.23**	**337.63**	**225.42**
	Hex3HexNAc1	3	1								689.24	**690.25**	**712.23**	**345.63**	**230.75**
	HexNAc2NeuAc1		2			1					697.25	**698.26**	**720.24**	**349.63**	**233.43**

	Ac-Hex1HexNAc1NeuAc1	1	1			1		1			698.24	**699.25**	**721.23**	**350.13**	**233.75**
	HexNAc2Fuc2		2	2							698.27	**699.28**	**721.26**	**350.14**	**233.77**
	Me-HexNAc2NA1		2			1	1				711.27	**712.28**	**734.26**	**356.64**	**238.10**
	Hex1HexNAc2Fuc1	1	2	1							714.27	**715.28**	**737.26**	**358.14**	**239.10**
	Hex2HexNAc2	2	2								730.26	**731.27**	**753.25**	**366.14**	**244.43**
	Ac-HexNAc2NeuAc1		2			1		1			739.26	**740.27**	**762.25**	**370.64**	**247.43**
	Hex1NeuAc2	1				2					744.24	**745.25**	**767.23**	**373.13**	**249.09**
	Hex2Fuc1NeuAc1	2		1		1					761.26	**762.27**	**784.25**	**381.64**	**254.76**
	Hex1HexNAc3	1	3								771.29	**772.30**	**794.28**	**386.65**	**258.10**
	Hex3Fuc2	3		2							778.27	**779.28**	**801.26**	**390.14**	**260.43**
	HexNAc1NeuAc2		1			2					785.27	**786.28**	**808.26**	**393.64**	**262.76**
	Hex1HexNAc1Fuc1NeuAc1	1	1	1		1					802.29	**803.29**	**825.27**	**402.15**	**268.44**
	Hex5	5									810.26	**811.27**	**833.25**	**406.14**	**271.10**
	HexNAc4		4								812.32	**813.32**	**835.31**	**407.17**	**271.78**
	Me-Hex1HexNAc1Fuc1NA1	1	1	1		1	1				816.30	**817.31**	**839.29**	**409.16**	**273.11**
	Hex2HexNAc1NeuAc1	2	1			1					818.28	**819.29**	**841.27**	**410.15**	**273.77**
	Hex2HexNAc1Fuc2	2	1	2							819.30	**820.31**	**842.29**	**410.66**	**274.11**
	Hex3HexNAc1Fuc1	3	1	1							835.30	**836.30**	**858.28**	**418.66**	**279.44**
	HexNAc2Fuc1NeuAc1		2	1		1					843.31	**844.32**	**866.30**	**422.66**	**282.11**
	Hex4HexNAc1	4	1								851.29	**852.30**	**874.28**	**426.65**	**284.77**
	Me-HexNAc2Fuc1NA1		2	1		1	1				857.33	**858.33**	**880.32**	**429.67**	**286.78**
	Hex1HexNAc2NeuAc1	1	2			1					859.31	**860.31**	**882.30**	**430.66**	**287.44**
	Hex1HexNAc2Fuc2	1	2	2							860.33	**861.33**	**883.32**	**431.17**	**287.78**
	Hex2HexNAc2Fuc1	2	2	1							876.32	**877.33**	**899.31**	**439.17**	**293.11**
	Hex3HexNAc2	3	2								892.32	**893.32**	**915.31**	**447.17**	**298.45**
	HexNAc3Fuc2		3	2							901.35	**902.36**	**924.34**	**451.68**	**301.46**
	HexNAc3Fuc2		3	2							901.35	**902.36**	**924.34**	**451.68**	**301.46**
	Hex2NeuAc2	2				2					906.30	**907.30**	**929.29**	**454.16**	**303.11**
	Hex2Fuc2NeuAc1	2		2		1					907.32	**908.32**	**930.31**	**454.67**	**303.45**
	Hex1HexNAc3Fuc1	1	3	1							917.35	**918.36**	**940.34**	**459.68**	**306.79**
	Hex3Fuc1NeuAc1	3		1		1					923.31	**924.32**	**946.30**	**462.66**	**308.78**
	Hex2HexNAc3	2	3								933.34	**934.35**	**956.33**	**467.68**	**312.12**
	Hex1HexNAc1NeuAc2	1	1			2					947.32	**948.33**	**970.31**	**474.67**	**316.78**
	Hex1HexNAc1Fuc2NeuAc1	1	1	2		1					948.34	**949.35**	**971.33**	**475.18**	**317.12**
	HexNAc4Fuc1		4	1							958.38	**959.38**	**981.36**	**480.19**	**320.47**
	Hex2HexNAc1Fuc1NeuAc1	2	1	1		1					964.34	**965.35**	**987.33**	**483.18**	**322.45**
	Hex6	6									972.32	**973.32**	**995.31**	**487.17**	**325.11**
	Hex1HexNAc4	1	4								974.37	**975.38**	**997.36**	**488.19**	**325.80**
	Hex3HexNAc1NeuAc1	3	1			1					980.33	**981.34**	**1003.32**	**491.17**	**327.79**
	HexNAc2NeuAc2		2			2					988.35	**989.36**	**1011.34**	**495.18**	**330.46**
	HexNAc2Fuc2NeuAc1		2	2		1					989.37	**990.38**	**1012.36**	**495.69**	**330.80**
	Hex4HexNAc1Fuc1	4	1	1							997.35	**998.36**	**1020.34**	**499.68**	**333.46**
	Hex1HexNAc2Fuc1NeuAc1	1	2	1		1					1005.36	**1006.37**	**1028.35**	**503.69**	**336.13**
	Hex5HexNAc1	5	1								1013.34	**1014.35**	**1036.33**	**507.68**	**338.79**
	HexNac5		5								1015.40	**1016.40**	**1038.39**	**508.71**	**339.47**
	Hex2HexNAc2NeuAc1	2	2			1					1021.36	**1022.37**	**1044.35**	**511.69**	**341.46**
	Hex2HexNAc2Fuc2	2	2	2							1022.38	**1023.39**	**1045.37**	**512.20**	**341.80**
	Hex3HexNAc2Fuc1	3	2	1							1038.38	**1039.38**	**1061.36**	**520.19**	**347.13**
	Hex4HexNAc2	4	2								1054.37	**1055.38**	**1077.36**	**528.19**	**352.46**
	Hex1HexNAc3NeuAc1	1	3			1					1062.39	**1063.39**	**1085.38**	**532.20**	**355.14**
	Hex1HexNAc3Fuc2	1	3	2							1063.41	**1064.41**	**1086.40**	**532.71**	**355.48**
	Hex2HexNAc3Fuc1	2	3	1							1079.40	**1080.41**	**1102.39**	**540.71**	**360.81**
	Hex3HexNAc3	3	3								1095.40	**1096.40**	**1118.39**	**548.71**	**366.14**
	Hex2HexNAc1NeuAc2	2	1			2					1109.38	**1110.38**	**1132.37**	**555.70**	**370.80**
	Hex2HexNAc1Fuc2NeuAc1	2	1	2		1					1110.40	**1111.40**	**1133.39**	**556.21**	**371.14**
	Hex1HexNAc4Fuc1	1	4	1							1120.43	**1121.44**	**1143.42**	**561.22**	**374.48**
	Hex3HexNAc1Fuc1NeuAc1	3	1	1		1					1126.39	**1127.40**	**1149.38**	**564.20**	**376.47**
	Hex7	7									1134.37	**1135.38**	**1157.36**	**568.19**	**379.13**
	Hex2HexNAc4	2	4								1136.42	**1137.43**	**1159.41**	**569.22**	**379.81**
	Hex1HexNAc2Fuc2NeuAc1	1	2	2		1					1151.42	**1152.43**	**1174.41**	**576.72**	**384.81**
	Hex5HexNAc1Fuc1	5	1	1							1159.40	**1160.41**	**1182.39**	**580.71**	**387.47**
	Hex2HexNAc2Fuc1NeuAc1	2	2	1		1					1167.42	**1168.42**	**1190.41**	**584.72**	**390.15**
	Hex6HexNAc1	6	1								1175.40	**1176.40**	**1198.39**	**588.71**	**392.81**
	Hex1HexNAc5	1	5								1177.45	**1178.46**	**1200.44**	**589.73**	**393.49**
	Hex3HexNAc2NeuAc1	3	2			1					1183.41	**1184.42**	**1206.40**	**592.71**	**395.48**
	Hex3HexNAc2Fuc2	3	2	2							1184.43	**1185.44**	**1207.42**	**593.22**	**395.82**
	HexNAc3Fuc2NeuAc1		3	2		1					1192.45	**1193.46**	**1215.44**	**597.23**	**398.49**
	Hex4HexNAc2Fuc1	4	2	1							1200.43	**1201.44**	**1223.42**	**601.22**	**401.15**
	Hex1HexNAc3Fuc1NeuAc1	1	3	1		1					1208.44	**1209.45**	**1231.43**	**605.23**	**403.82**
	Hex1HexNAc3Fuc1NeuAc1	1	3	1		1					1208.44	**1209.45**	**1231.43**	**605.23**	**403.82**

	Hex5HexNAc2	5	2								1216.42	1217.43	1239.41	609.22	406.48
	Hex2HexNAc3NeuAc1	2	3			1					1224.44	1225.45	1247.43	613.23	409.15
	Hex2HexNAc3Fuc2	2	3	2							1225.46	1226.47	1248.45	613.74	409.49
	Hex3HexNAc3Fuc1	3	3	1							1241.45	1242.46	1264.44	621.73	414.83
	Hex2HexNAc1Fuc1NeuAc2	2	1	1		2					1255.43	1256.44	1278.42	628.72	419.49
	Hex4HexNAc3	4	3								1257.45	1258.46	1280.44	629.73	420.16
	Hex1HexNAc4Fuc2	1	4	2							1266.49	1267.49	1289.48	634.25	423.17
	Hex2HexNAc4Fuc1	2	4	1							1282.48	1283.49	1305.47	642.25	428.50
	Hex4HexNAc1Fuc1NeuAc1	4	1	1		1					1288.44	1289.45	1311.43	645.23	430.49
	Hex8	8									1296.42	1297.43	1319.41	649.22	433.15
	Hex3HexNAc4	3	4								1298.48	1299.48	1321.47	650.25	433.83
	Hex5HexNAc1NeuAc1	5	1			1					1304.44	1305.45	1327.43	653.23	435.82
	Hex5HexNAc1Fuc2	5	1	2							1305.46	1306.47	1328.45	653.74	436.16
	Hex1HexNAc5Fuc1	1	5	1							1323.51	1324.51	1346.50	662.76	442.18
	Hex3HexNAc2Fuc1NeuAc1	3	2	1		1					1329.47	1330.48	1352.46	665.74	444.16
	Hex7HexNAc1	7	1								1337.45	1338.46	1360.44	669.73	446.82
	Hex2HexNAc5	2	5								1339.50	1340.51	1362.49	670.76	447.51
	Hex4HexNAc2NeuAc1	4	2			1					1345.47	1346.47	1368.45	673.74	449.50
	Hex4HexNAc2Fuc2	4	2	2							1346.49	1347.49	1369.48	674.25	449.84
	Hex5HexNAc2Fuc1	5	2	1							1362.48	1363.49	1385.47	682.25	455.17
	Hex2HexNAc3Fuc1NeuAc1	2	3	1		1					1370.50	1371.50	1393.49	686.26	457.84
	Hex6HexNAc2	6	2								1378.48	1379.48	1401.46	690.25	460.50
	Hex1HexNAc6	1	6								1380.53	1381.54	1403.52	691.27	461.18
	Hex3HexNAc3NeuAc1	3	3			1					1386.49	1387.50	1409.48	694.25	463.17
	Hex3HexNAc3Fuc2	3	3	2							1387.51	1388.52	1410.50	694.76	463.51
	Hex4HexNAc3Fuc1	4	3	1							1403.51	1404.51	1426.50	702.76	468.84
	Hex1HexNAc4Fuc1NeuAc1	1	4	1		1					1411.52	1412.53	1434.51	706.77	471.52
	Hex5HexNAc3	5	3								1419.50	1420.51	1442.49	710.76	474.17
	Hex2HexNAc4NeuAc1	2	4			1					1427.52	1428.53	1450.51	714.77	476.85
	Hex2HexNAc4Fuc2	2	4	2							1428.54	1429.55	1451.53	715.28	477.19
	Hex3HexNAc4Fuc1	3	4	1							1444.53	1445.54	1467.52	723.27	482.52
	Hex5HexNAc1Fuc1NeuAc1	5	1	1		1					1450.50	1451.50	1473.49	726.26	484.51
	Hex4HexNAc4	4	4								1460.53	1461.54	1483.52	731.27	487.85
	Hex6HexNAc1NeuAc1	6	1			1					1466.49	1467.50	1489.48	734.25	489.84
	Hex1HexNAc5NeuAc1	1	5			1					1468.55	1469.55	1491.53	735.28	490.52
	Hex1HexNAc5Fuc2	1	5	2							1469.57	1470.57	1492.55	735.79	490.86
	Hex3HexNAc2Fuc2NeuAc1	3	2	2		1					1475.53	1476.54	1498.52	738.77	492.85
	Hex2HexNAc5Fuc1	2	5	1							1485.56	1486.57	1508.55	743.79	496.19
	Hex4HexNAc2Fuc1NeuAc1	4	2	1		1					1491.52	1492.53	1514.51	746.77	498.18
	Hex3HexNAc5	3	5								1501.56	1502.56	1524.54	751.78	501.53
	Hex5HexNAc2NeuAc1	5	2			1					1507.52	1508.53	1530.51	754.77	503.51
	Hex2HexNAc3Fuc2NeuAc1	2	3	2		1					1516.55	1517.56	1539.54	759.28	506.53
	Hex1HexNAc6Fuc1	1	6	1							1526.59	1527.59	1549.58	764.30	509.87
	Hex3HexNAc3Fuc1NeuAc1	3	3	1		1					1532.55	1533.56	1555.54	767.28	511.86
	Hex2HexNAc6	2	6								1542.58	1543.59	1565.57	772.30	515.20
	Hex4HexNAc3NeuAc1	4	3			1					1548.54	1549.55	1571.53	775.28	517.19
	Hex1HexNAc4Fuc2NeuAc1	1	4	2		1					1557.58	1558.59	1580.57	779.80	520.20
	Hex2HexNAc4Fuc1NeuAc1	2	4	1		1					1573.58	1574.58	1596.57	787.80	525.53
	Hex3HexNAc4NeuAc1	3	4			1					1589.57	1590.58	1612.56	795.79	530.86
	Hex1HexNAc5Fuc1NeuAc1	1	5	1		1					1614.60	1615.61	1637.59	808.31	539.21
	Hex2HexNAc5NeuAc1	2	5			1					1630.60	1631.61	1653.59	816.31	544.54
	Hex1HexNAc6NeuAc1	1	6			1					1671.62	1672.63	1694.61	836.82	558.22

PA, pyridylaminated; M3PA, Man3GlcNAc2-PA; Hex, hexose; HexNAc, N-acetylhexosamin; dHex, deoxy-hexose; Pen, pentose; NANA, N-acetylneuraminic acid
Me, methylation; Ac, acetylation; HPO_3, phosphorylation; SO_3, sulfation

Supplementary Table S8

LC-ESI-MS and MS^2 measurement

・HPLC: Dionex Ultimate 3000RS UHPLC (Thermo Scientific, San Jose, CA)

Column: Hypercarb Guard Cartridge (5 µm, 1 mm × 10 mm) (Thermo Scientific)

Column Temp: 25 ℃

Flow rate: 0.050 ml/min

Eluent A: 0.1% Formic acid in water

Eluent B: 0.5% Formic acid in acetonitrile

・MS: LTQ XL linear ion trap mass spectrometer (Thermo Scientific)

Ion source: Ion Max ion source (Thermo Scientific)

ESI probe: HESI-II probe (Thermo Scientific)

・ESI-MS settings

Ion mode: Positive ion mode

Scan range: *m/z* 500–2000

Scan rate: Enhanced mode

Electrospray voltage: 4.0 kV

Tube lens voltage: 80 V

Capillary Temp: 220 °C

Capillary voltage: 40 V

Source Heater Temp: 100 °C

Sheath Gas Flow: 20 (arbitrary units)

Auxiliary Gas Flow: 2 (arbitrary units)

Full Micro Scans: 1

Full Max Ion Time: 50 ms

Full AGC Target: 100000

・Data dependent MS^2 (Top 3 Method) settings

Scan rate: Normal mode

Activation Type: collision-induced dissociation

Minimum Signal threshold of MS peak: 600

Isolation Mass Width: 4.0 *m/z*

Normalized Collision Energy: 35.0

Activation Q: 0.25

Activation Time: 30.0

MS^n Micro Scans: 1

MS^n Max Ion Time: 400 ms

MS^n AGC Target: 10000

Supplementary Table S9

MALDI-QIT-TOF MS measurement

・MS: AXIMA-Resonance mass spectrometer (Shimadzu/Kratos, Manchester, UK)

Matrix solution for positive-ion mode analysis: 1% 1,1,3,3-tetramethylguanidium salt of *p*-coumaric acid (G_3CA) liquid matrix in 50% acetonitrile[1]

Matrix solution for negative-ion mode analysis: 100 mM 3-Aminoquinoline/ *p*-coumaric acid (3-AQ/CA) with 2 mM ammonium dihydrogen phosphate in 50% acetonitrile[2]

Laser for ionization: nitrogen UV laser (337 nm)

Sample stage voltage for ion extraction: 30 V

Ion acceleration voltage after ion trap to TOF: 10 kV

・MS Settings (manual measurement)

Scan mode: high mass mode (range: *m/z* 2000~)

Laser power: just above threshold for ion production to avoid in-source decay

Ion cooling gas: He

・MS^n settings (manual measurement)

Activation type: collision-induced dissociation

Activation gas: Ar

Isolation width: Standard (resolution; 250)

Activation energy: 170~200 (arbitrary units)

References

1. Fukuyama, Y., Nakaya, S., Yamazaki, Y. & Tanaka, K. Ionic liquid matrixes optimized for MALDI-MS of sulfated/sialylated/neutral oligosaccharides and glycopeptides. *Anal Chem*. **80**, 2171-2179 (2008).
2. Fukuyama, Y. *et al.* 3-Aminoquinoline/p-coumaric acid as a MALDI matrix for glycopeptides, carbohydrates, and phosphopeptides. *Anal Chem*. **86**, 1937-1942 (2014).
